# Supplementary material for: Hemodynamic, Surgical and Oncological Outcomes of 40 Distal Pancreatectomies with Celiac and Left Gastric Arteries Resection (DP CAR) without Arterial Reconstructions and Preoperative Embolization
Source: Cancers (Basel). 2022 Feb 28;14(5):1254. doi: 10.3390/cancers14051254 (PMC8909059; doi:10.3390/cancers14051254)
Supplement: Supplementary file 1 [file cancers-14-01254-s001.zip › cancers-1582412-supplementary/Supplement/Figure 1S.pdf]

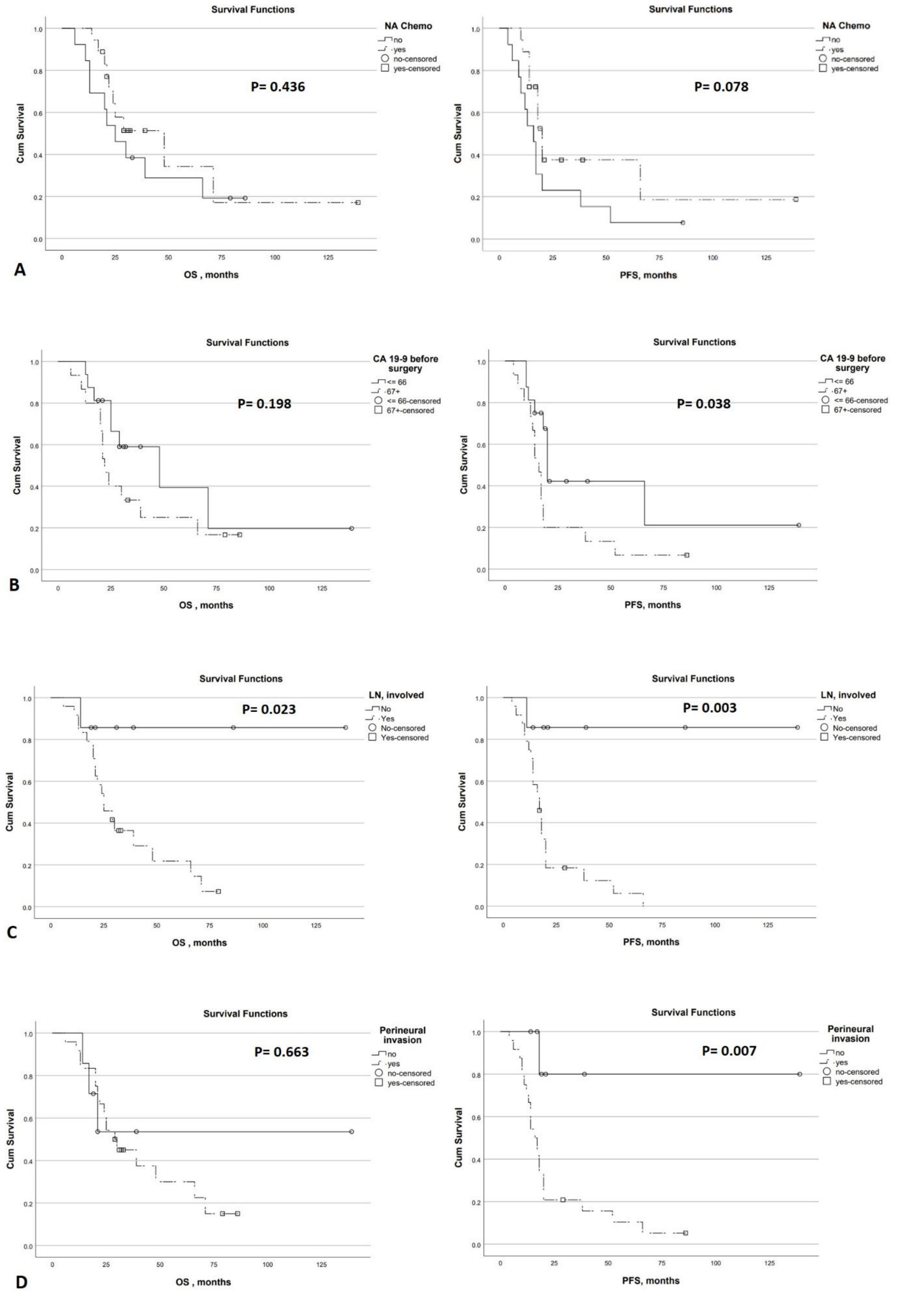
Survival Functions
NA Chemo
no
yes
no-censored
yes-censored
P= 0.436
Cum Survival
OS , months
A
Survival Functions
NA Chemo
no
yes
no-censored
yes-censored
P= 0.078
Cum Survival
PFS, months
Survival Functions
CA 19-9 before surgery
<= 66
67+
<= 66-censored
67+-censored
P= 0.198
Cum Survival
OS , months
B
Survival Functions
CA 19-9 before surgery
<= 66
67+
<= 66-censored
67+-censored
P= 0.038
Cum Survival
PFS, months
Survival Functions
LN, involved
No
Yes
No-censored
Yes-censored
P= 0.023
Cum Survival
OS , months
C
Survival Functions
LN, involved
No
Yes
No-censored
Yes-censored
P= 0.003
Cum Survival
PFS, months
Survival Functions
Perineural invasion
no
yes
no-censored
yes-censored
P= 0.663
Cum Survival
OS , months
D
Survival Functions
Perineural invasion
no
yes
no-censored
yes-censored
P= 0.007
Cum Survival
PFS, months

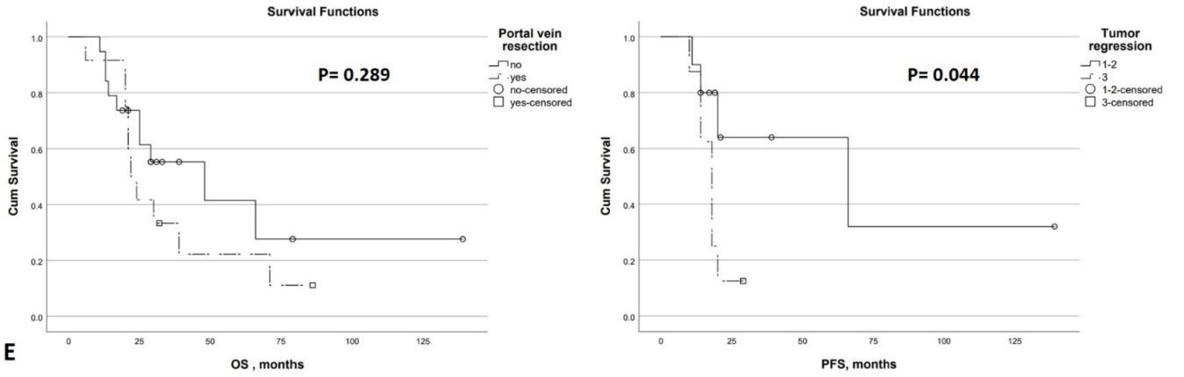

Figure S1. Significant and possibly significant predictors of progression-free and overall survival for PDAC. A. RFS and OS for neoadjuvant chemotherapy. B, RFS and OS for preoperative CA19-9. C, RFS and OS for lymph node involvement, D, RFS and OS for perineural invasion, E, PFS for tumor regression score and OS for concomitant PV/SMV resection.

Gender, body mass index, age, tumor size, morbidity, concomitant resection of one hepatic artery and portal vein did not influence overall and progression-free survival.

In the case of neoadjuvant chemotherapy, overall and progression-free survival was 48 [95% CI:17; 42] and 20 [95% CI:16; 24] months, respectively. If neoadjuvant chemotherapy was not used, these values were 25 [95% CI:13; 37] and 16 [95% CI:12; 20] months, respectively. Neoadjuvant chemotherapy was associated with increased overall and progression-free survival, although the improvement was insignificant (Fig. 1Sa)

Preoperative CA 19-9 level <66 U/mL was accompanied by higher PFS and OS:(20 [95% CI: 18; 22] and16 [95% CI: 13; 19] months, and 48 [95% CI: 13; 83] and 22 [95% CI: 18; 26] months) respectively (Fig. 1Sb).

Lymph node invasion significantly reduced overall and progression-free survival (25 [95% CI: 17;34] and 17 [95% CI: 14;20] months, respectively), in N_0-patients median survival was not achieved for both overall and progression-free survival (Fig. 1Sc).

Perineural invasion significantly reduced progression-free (16 [95% CI: 13; 19] months) and overall survival (29 [95% CI: 22; 36] months). Median survival was not achieved for OS or PFS if the perineural invasion was absent (Fig. 1Sd)

The association of portal vein resection and tumor regression score is presented on Figure 1Se.
