# Supplementary material for: Hemodynamic, Surgical and Oncological Outcomes of 40 Distal Pancreatectomies with Celiac and Left Gastric Arteries Resection (DP CAR) without Arterial Reconstructions and Preoperative Embolization
Source: Cancers (Basel). 2022 Feb 28;14(5):1254. doi: 10.3390/cancers14051254 (PMC8909059; doi:10.3390/cancers14051254)
Supplement: Supplementary file 1 [file cancers-14-01254-s001.zip › cancers-1582412-supplementary/Supplement/File S1. Statistics 1 for PDAC survival.pdf]

File S1. Statistics. Part 1. Pancreatic ductal adenocarcinoma patients' survival. Frequencies

Gender

		Frequency	Percent	Valid Percent	Cumulative Percent
Valid	f	16	51.6	51.6	51.6
	m	15	48.4	48.4	100.0
	Total	31	100.0	100.0	

Additional artery resection

		Frequency	Percent	Valid Percent	Cumulative Percent
Valid	no	23	74.2	74.2	74.2
	yes	8	25.8	25.8	100.0
	Total	31	100.0	100.0	

NA Chemo

		Frequency	Percent	Valid Percent	Cumulative Percent
Valid	no	13	41.9	41.9	41.9
	yes	18	58.1	58.1	100.0
	Total	31	100.0	100.0	

Adj Chemo

		Frequency	Percent	Valid Percent	Cumulative Percent
Valid	no	2	6.5	6.5	6.5
	yes	29	93.5	93.5	100.0
	Total	31	100.0	100.0	

Portal vein resection

		Frequency	Percent	Valid Percent	Cumulative Percent
Valid	no	19	61.3	61.3	61.3
	yes	12	38.7	38.7	100.0
	Total	31	100.0	100.0	

Histologic type. G

		Frequency	Percent	Valid Percent	Cumulative Percent
Valid	1	5	16.1	16.1	16.1
	2	21	67.7	67.7	83.9

		Frequency	Percent	Valid Percent	Cumulative Percent
	3	5	16.1	16.1	100.0
	Total	31	100.0	100.0	

LN. involved

		Frequency	Percent	Valid Percent	Cumulative Percent
Valid	0	7	22.6	22.6	22.6
	1	6	19.4	19.4	41.9
	2	4	12.9	12.9	54.8
	3	7	22.6	22.6	77.4
	4	3	9.7	9.7	87.1
	5	2	6.5	6.5	93.5
	6	2	6.5	6.5	100.0
	Total	31	100.0	100.0	

Perineural invasion

		Frequency	Percent	Valid Percent	Cumulative Percent
Valid	no	7	22.6	22.6	22.6
	yes	24	77.4	77.4	100.0
	Total	31	100.0	100.0	

R-status

		Frequency	Percent	Valid Percent	Cumulative Percent
Valid	no	28	90.3	90.3	90.3
	yes	3	9.7	9.7	100.0
	Total	31	100.0	100.0	

Alive

		Frequency	Percent	Valid Percent	Cumulative Percent
Valid	dead	20	64.5	64.5	64.5
	alive	11	35.5	35.5	100.0
	Total	31	100.0	100.0	

Progression

		Frequency	Percent	Valid Percent	Cumulative Percent
Valid	no	8	25.8	25.8	25.8

	yes	23	74.2	74.2	100.0
	Total	31	100.0	100.0	

Regression grade

		Frequency	Percent	Valid Percent	Cumulative Percent
Valid	0	1	3.2	5.0	5.0
	1	1	3.2	5.0	10.0
	2	9	29.0	45.0	55.0
	3	9	29.0	45.0	100.0
	Total	20	64.5	100.0	
Missing	System	11	35.5		
Total		31	100.0		

Dindo-C

		Frequency	Percent	Valid Percent	Cumulative Percent
Valid	0	22	71.0	71.0	71.0
	1	3	9.7	9.7	80.6
	2	5	16.1	16.1	96.8
	3	1	3.2	3.2	100.0
	Total	31	100.0	100.0	

Frequencies

BMI 22.016 ±3.0591
CA 19-9 before CT 292 [112;551]
CA 19-9 after CT (18) 45[19;67.5]
CA 19-9 before CT/after CT (18) 8.94 [5.04:15.9]
LN all 30.87±4.463
Time of follow up. months 27 [22;39]

Statistics

	N						Percentiles		
	Valid	Missing	Mean	Std. Deviation	Minimum	Maximum	25	50	75
BMI	31	0	22.016	3.0591	18.0	29.5	19.500	21.000	25.000
Age	31	0	61.55	7.924	39	73	59.00	64.00	66.00
CA 19-9 before CT	31	0	352.497	266.6493	.4	1145.0	112.000	292.000	551.000
CA 19-9 after CT (18)	18	13	50.94	40.473	14	144	19.00	45.00	67.50

CA 19-9 before CT/after CT	18	13	11.4550	7.35294	1.80	29.32	5.0475	8.9400	15.9050
LN all	31	0	30.87	4.463	22	43	28.00	31.00	33.00
~~OS . months~~	~~31~~	~~0~~	~~34.58~~	~~27.629~~	~~6~~	~~139~~	~~20.00~~	~~25.00~~	~~39.00~~
~~PFS. months~~	~~31~~	~~0~~	~~25.84~~	~~27.407~~	~~4~~	~~139~~	~~13.00~~	~~17.00~~	~~21.00~~
Time of follow up. months	31	0	35.97	27.384	11	140	22.00	27.00	39.00

Mean±SD 62±8 лет

Median [Q1;Q3] 292 [112;551]

Histogram

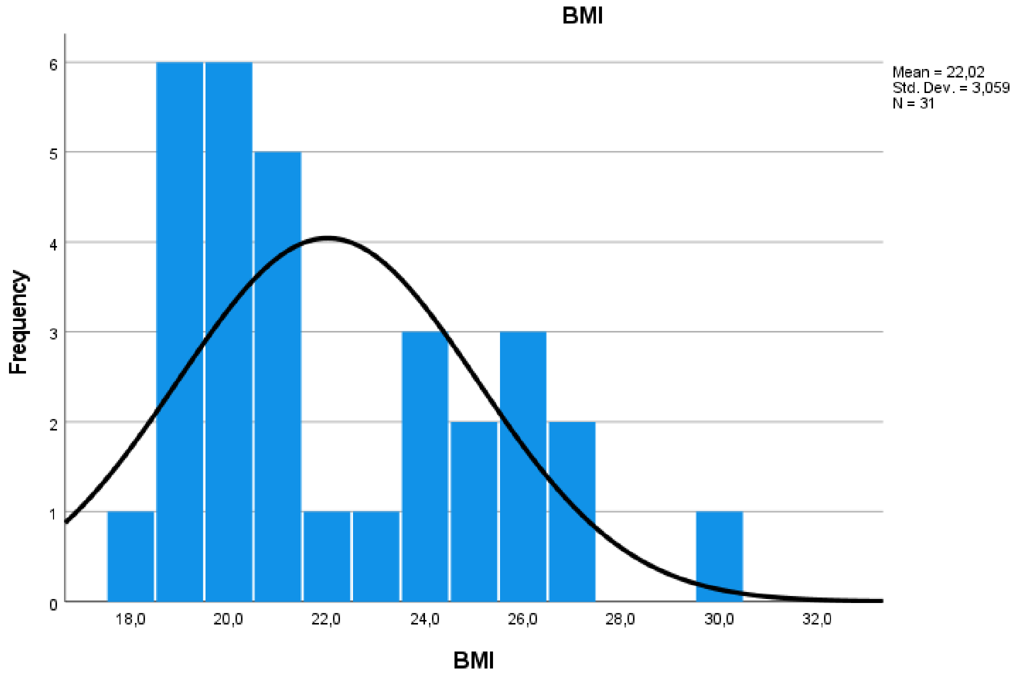

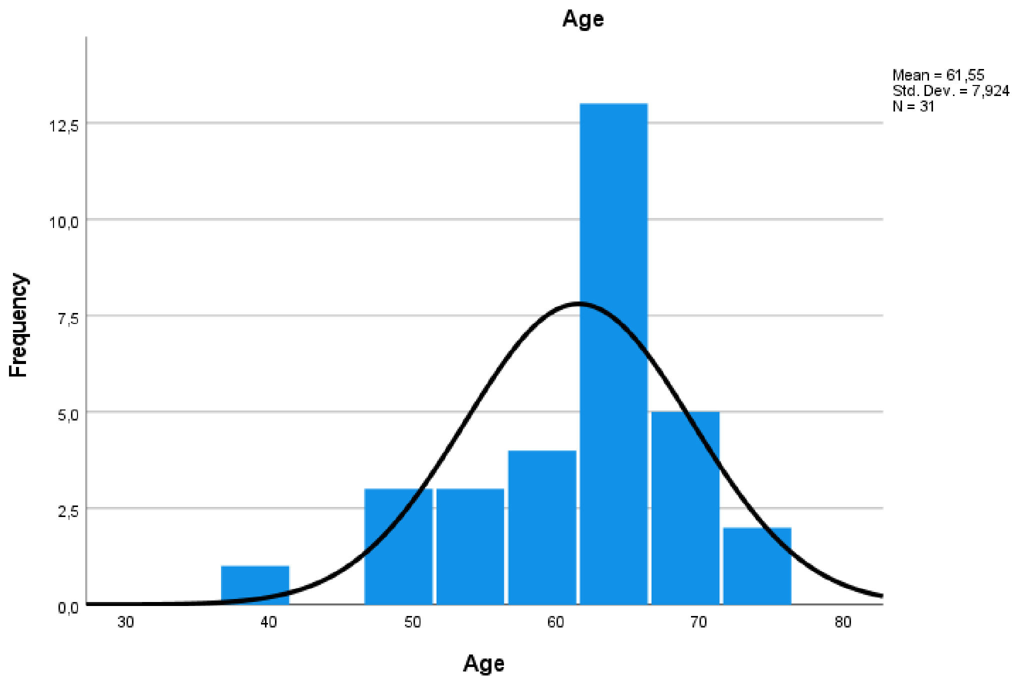

Age
Mean = 61,55
Std. Dev. = 7,924
N = 31
Frequency
12,5
10,0
7,5
5,0
2,5
0,0
30
40
50
60
70
80
Age

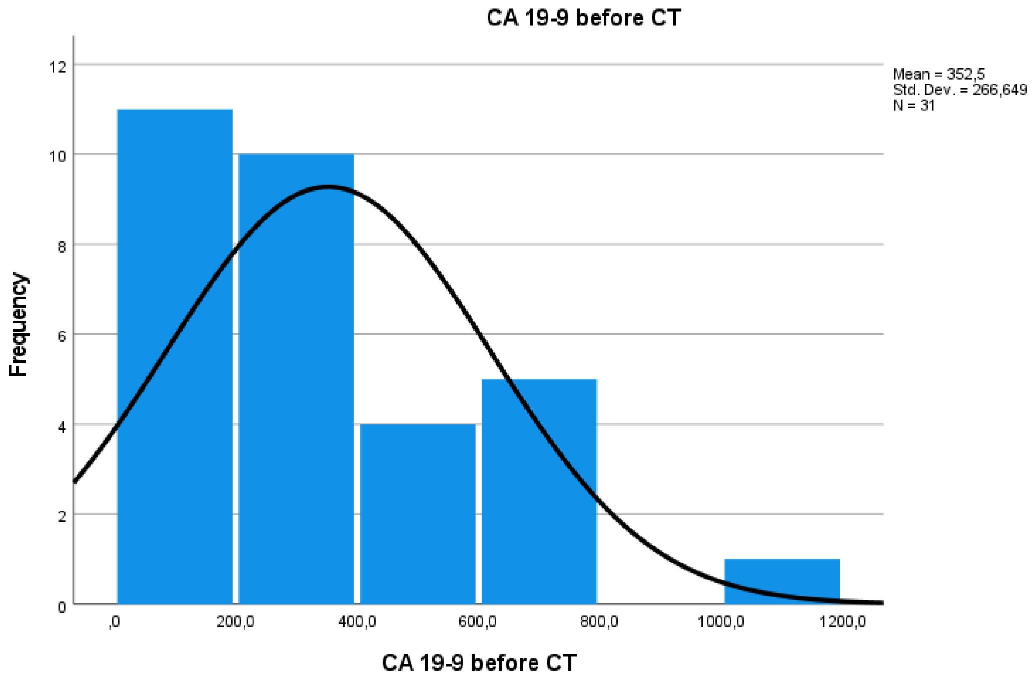

CA 19-9 before CT
Mean = 352,5
Std. Dev. = 266,649
N = 31
Frequency
12
10
8
6
4
2
0
,0
200,0
400,0
600,0
800,0
1000,0
1200,0
CA 19-9 before CT

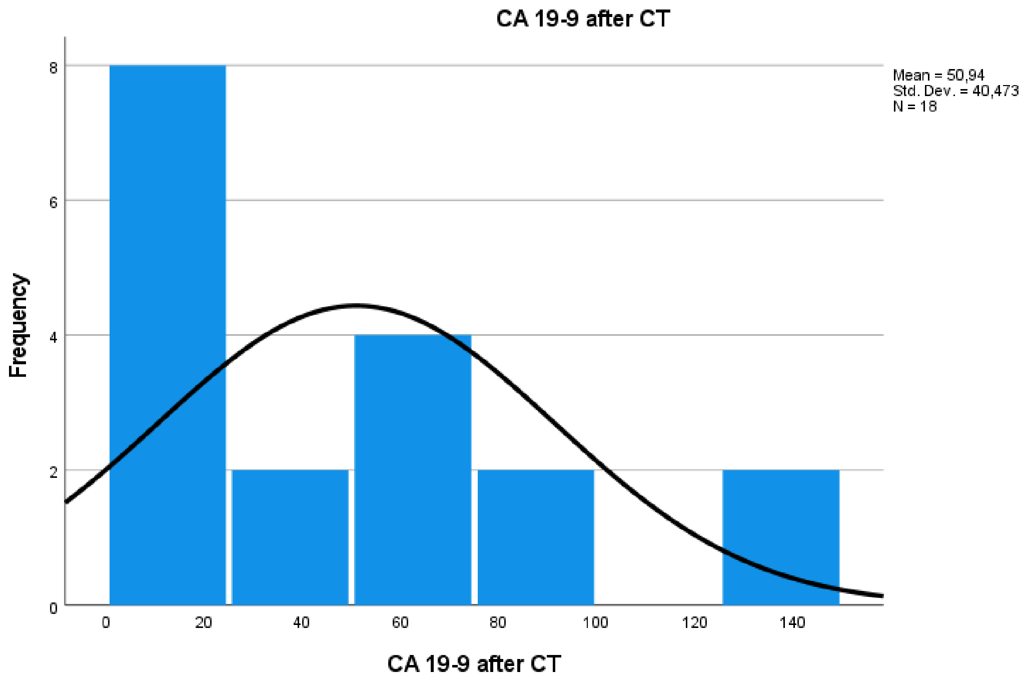
CA 19-9 after CT
Mean = 50,94
Std. Dev. = 40,473
N = 18
Frequency
0
2
4
6
8
0
20
40
60
80
100
120
140
CA 19-9 after CT

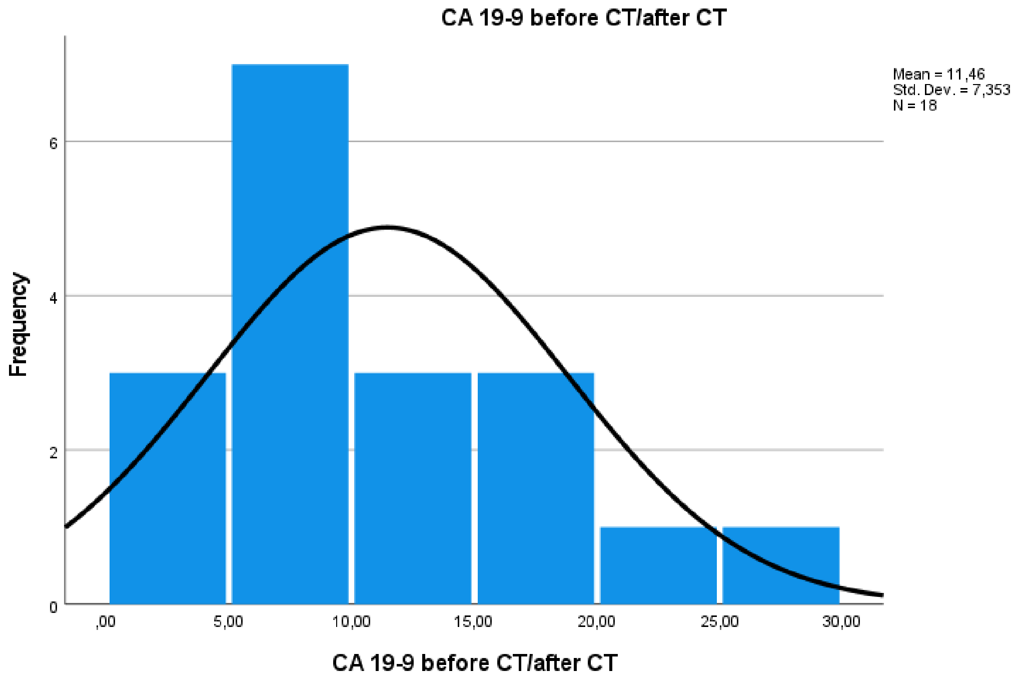
CA 19-9 before CT/after CT
Mean = 11,46
Std. Dev. = 7,353
N = 18
Frequency
0
2
4
6
,00
5,00
10,00
15,00
20,00
25,00
30,00
CA 19-9 before CT/after CT

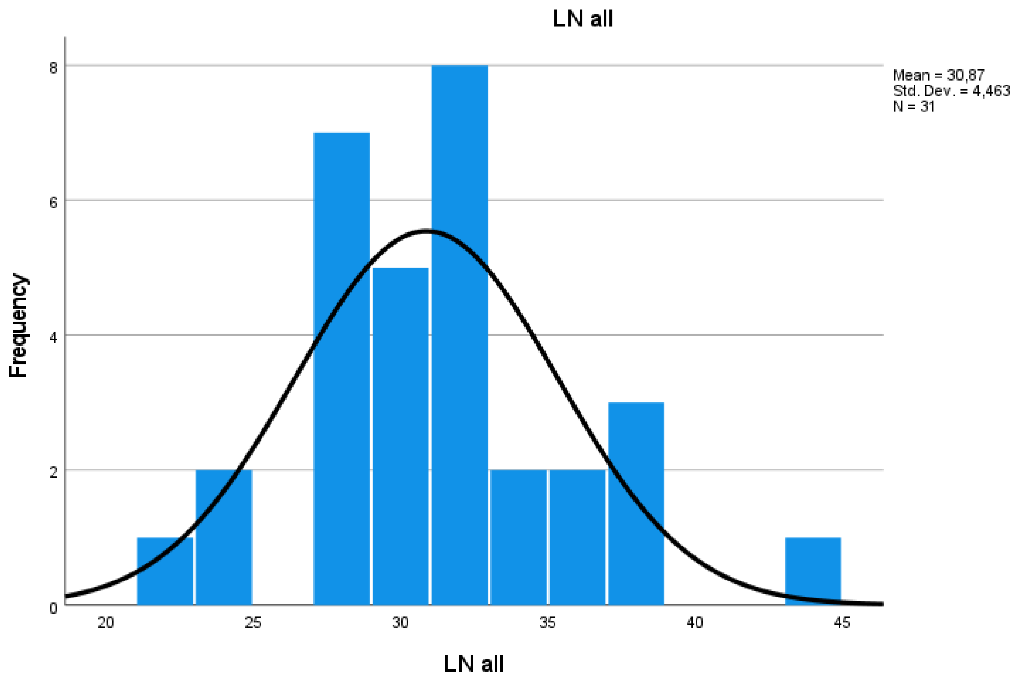
LN all
Mean = 30,87
Std. Dev. = 4,463
N = 31
Frequency
0
2
4
6
8
20
25
30
35
40
45
LN all

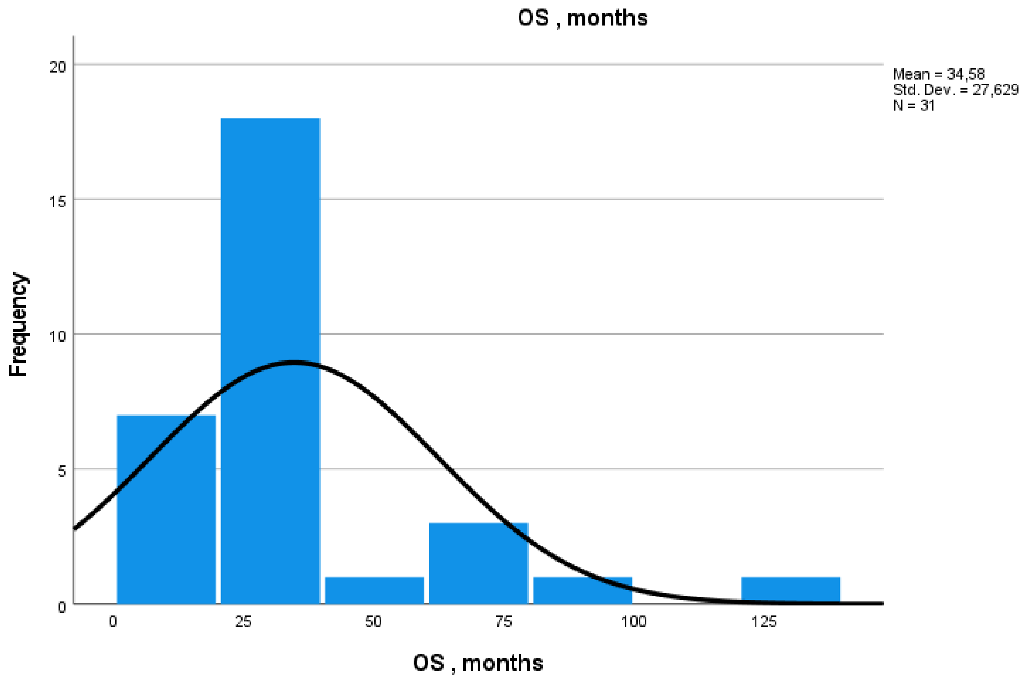
OS , months
Mean = 34,58
Std. Dev. = 27,629
N = 31
Frequency
0
5
10
15
20
0
25
50
75
100
125
OS , months

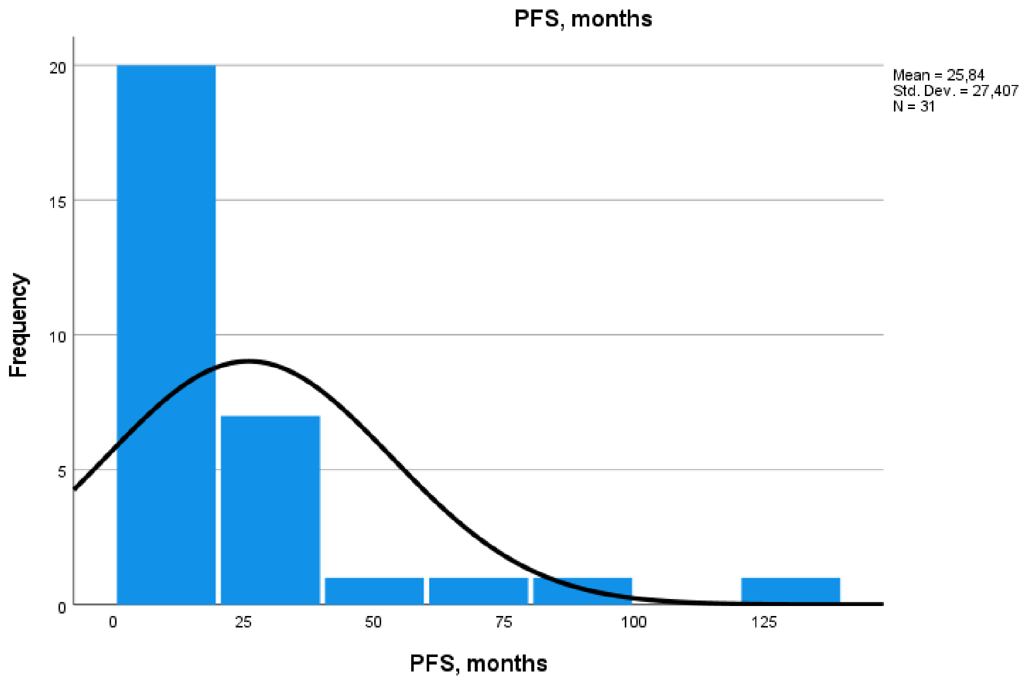

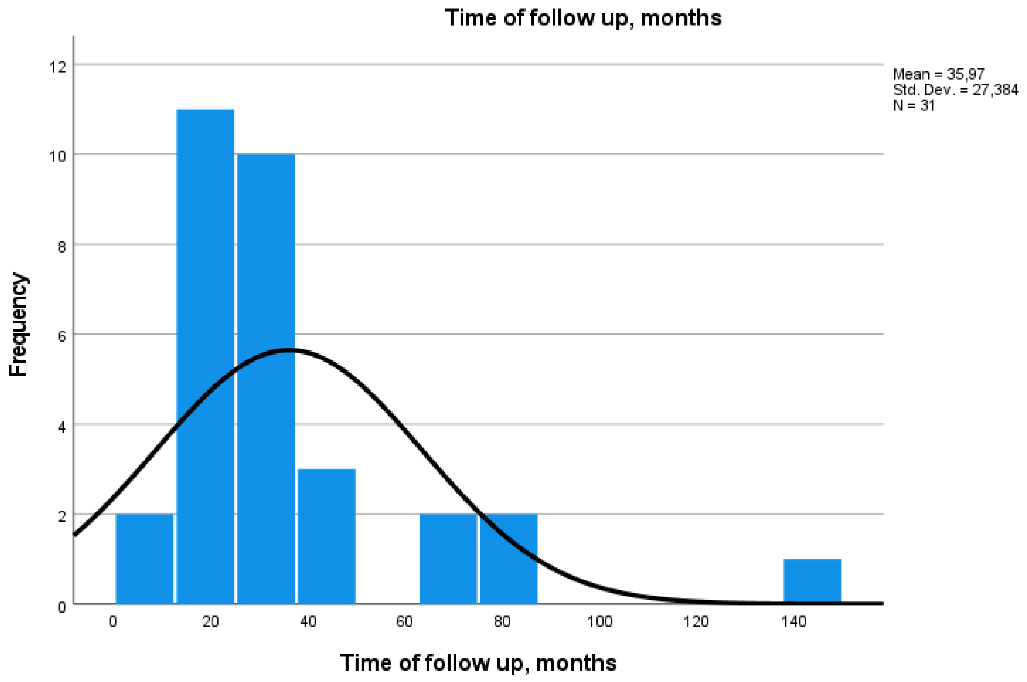

Overall survival and analysis of factors influencing it

Kaplan-Meier

Case Processing Summary

Total N	N of Events	Censored	
		N	Percent
31	20	11	35.5%

Survival Table

	Time	Status	Cumulative Proportion Surviving at the Time		N of Cumulative Events	N of Remaining Cases
			Estimate	Std. Error		
1	6.000	dead	.968	.032	1	30
2	11.000	dead	.935	.044	2	29
3	13.000	dead	.	.	3	28
4	13.000	dead	.871	.060	4	27
5	14.000	dead	.839	.066	5	26
6	17.000	dead	.806	.071	6	25
7	19.000	alive	.	.	6	24
8	20.000	dead	.	.	7	23
9	20.000	dead	.739	.079	8	22
10	21.000	dead	.	.	9	21
11	21.000	dead	.672	.085	10	20
12	21.000	alive	.	.	10	19
13	22.000	dead	.637	.088	11	18
14	24.000	dead	.601	.090	12	17
15	25.000	dead	.	.	13	16
16	25.000	dead	.531	.092	14	15
17	29.000	dead	.495	.092	15	14
18	29.000	alive	.	.	15	13
19	29.000	alive	.	.	15	12
20	30.000	dead	.454	.094	16	11
21	31.000	alive	.	.	16	10
22	32.000	alive	.	.	16	9
23	33.000	alive	.	.	16	8
24	39.000	dead	.397	.098	17	7
25	39.000	alive	.	.	17	6
26	48.000	dead	.331	.101	18	5
27	66.000	dead	.265	.100	19	4
28	71.000	dead	.199	.095	20	3
29	79.000	alive	.	.	20	2
30	86.000	alive	.	.	20	1
31	139.000	alive	.	.	20	0

Means and Medians for Overall Survival Time

29.000±6.795	[95%CI:15.681;42.319]		

Means and Medians for Survival Time

Mean	Median

Estimate	Std. Error	95% Confidence Interval		Estimate	Std. Error	95% Confidence Interval	
		Lower Bound	Upper Bound			Lower Bound	Upper Bound
52.865	9.885	33.490	~~72.240~~	29.000	6.795	15.681	42.319

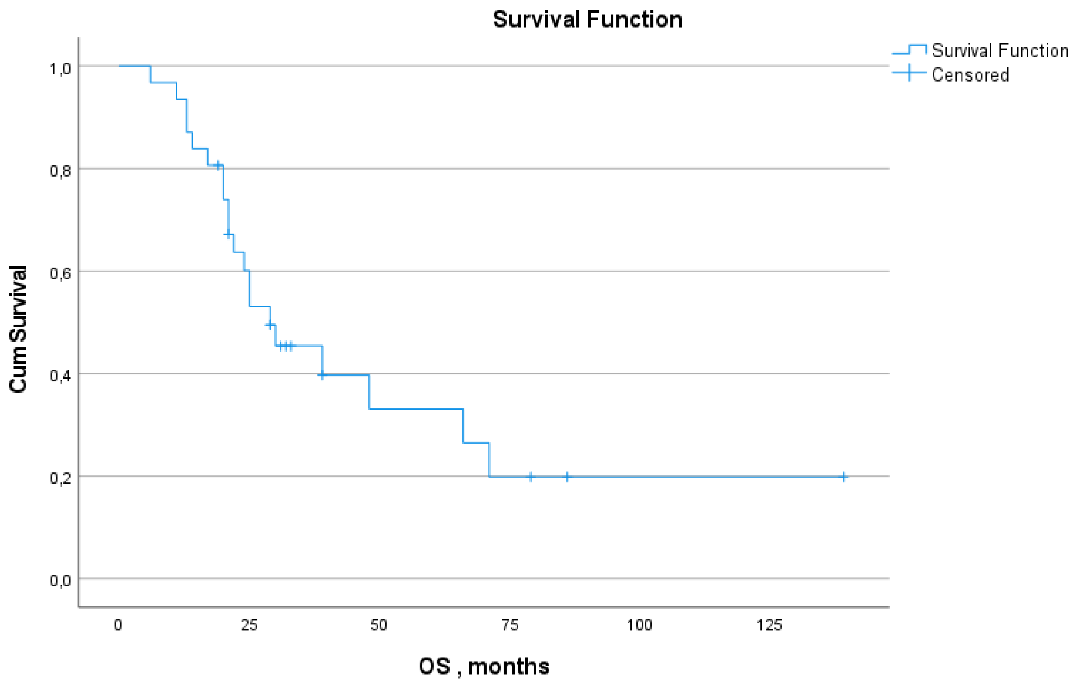

Kaplan-Meier

Case Processing Summary

Gender	Total N	N of Events	Censored	
			N	Percent
f	16	12	4	25.0%
m	15	8	7	46.7%
Overall	31	20	11	35.5%

Survival Table

Gender		Time	Status	Cumulative Proportion Surviving at the Time		N of Cumulative Events	N of Remaining Cases
				Estimate	Std. Error		
f	1	6.000	dead	.938	.061	1	15
	2	13.000	dead	.875	.083	2	14
	3	14.000	dead	.813	.098	3	13
	4	19.000	alive	.	.	3	12
	5	22.000	dead	.745	.110	4	11
	6	25.000	dead	.	.	5	10
	7	25.000	dead	.609	.125	6	9
	8	29.000	dead	.542	.128	7	8

	9	29.000	alive	.	.	7	7
	10	30.000	dead	.464	.131	8	6
	11	32.000	alive	.	.	8	5
	12	39.000	dead	.371	.134	9	4
	13	48.000	dead	.279	.129	10	3
	14	66.000	dead	.186	.114	11	2
	15	71.000	dead	.093	.087	12	1
	16	139.000	alive	.	.	12	0
m	1	11.000	dead	.933	.064	1	14
	2	13.000	dead	.867	.088	2	13
	3	17.000	dead	.800	.103	3	12
	4	20.000	dead	.	.	4	11
	5	20.000	dead	.667	.122	5	10
	6	21.000	dead	.	.	6	9
	7	21.000	dead	.533	.129	7	8
	8	21.000	alive	.	.	7	7
	9	24.000	dead	.457	.131	8	6
	10	29.000	alive	.	.	8	5
	11	31.000	alive	.	.	8	4
	12	33.000	alive	.	.	8	3
	13	39.000	alive	.	.	8	2
	14	79.000	alive	.	.	8	1
	15	86.000	alive	.	.	8	0

Means and Medians for Survival Time m-f .739

	Mean				Median			
			95% Confidence Interval				95% Confidence Interval	
Gender	Estimate	Std. Error	Lower Bound	Upper Bound	Estimate	Std. Error	Lower Bound	Upper Bound
f	44.930	10.018	25.294	64.565	30.000	7.722	14.866	45.134
m	49.343	8.857	31.982	66.703	24.000	.	.	.
Overall	52.865	9.885	33.490	72.240	29.000	6.795	15.681	42.319

Overall Comparisons

	Chi-Square	df	Sig.
Log Rank (Mantel-Cox)	.111	1	.739

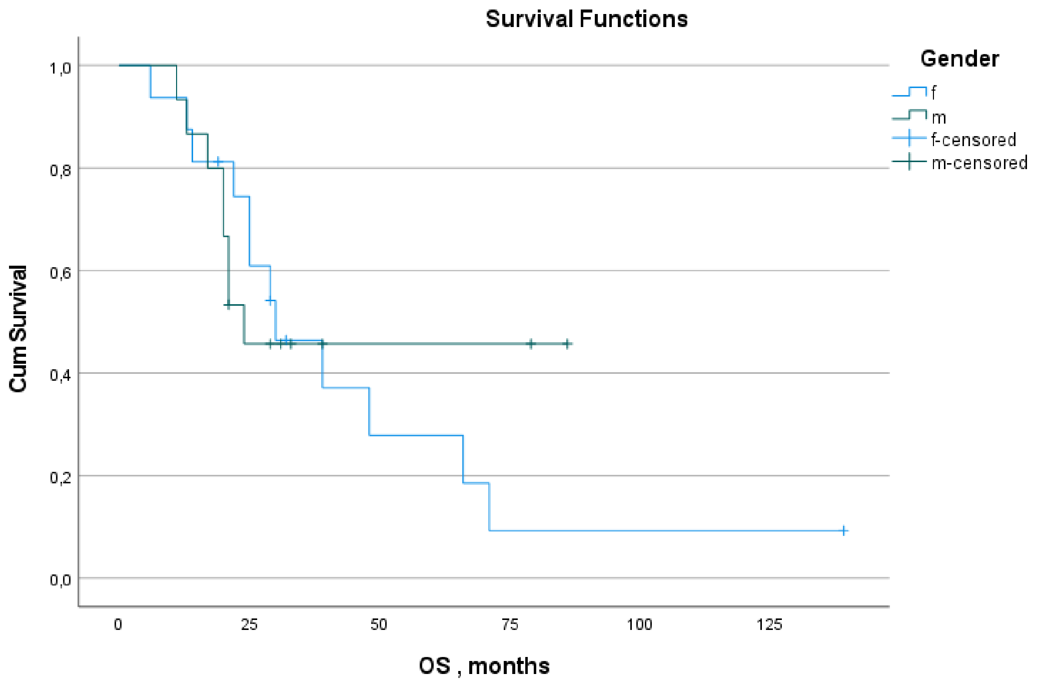

Kaplan-Meier

Case Processing Summary

Additional artery resection	Total N	N of Events	Censored	
			N	Percent
no	23	15	8	34.8%
yes	8	5	3	37.5%
Overall	31	20	11	35.5%

Survival Table

Additional artery resection		Time	Status	Cumulative Proportion Surviving at the Time		N of Cumulative Events	N of Remaining Cases
				Estimate	Std. Error		
no	1	6.000	dead	.957	.043	1	22
	2	11.000	dead	.913	.059	2	21
	3	13.000	dead	.	.	3	20
	4	13.000	dead	.826	.079	4	19
	5	14.000	dead	.783	.086	5	18
	6	19.000	alive	.	.	5	17
	7	20.000	dead	.737	.092	6	16
	8	21.000	dead	.	.	7	15
	9	21.000	dead	.645	.101	8	14
	10	22.000	dead	.598	.104	9	13
	11	25.000	dead	.	.	10	12
	12	25.000	dead	.506	.106	11	11

	13	29.000	alive	.	.	11	10
	14	29.000	alive	.	.	11	9
	15	30.000	dead	.450	.108	12	8
	16	31.000	alive	.	.	12	7
	17	32.000	alive	.	.	12	6
	18	33.000	alive	.	.	12	5
	19	48.000	dead	.360	.118	13	4
	20	66.000	dead	.270	.118	14	3
	21	71.000	dead	.180	.108	15	2
	22	79.000	alive	.	.	15	1
	23	86.000	alive	.	.	15	0
yes	1	17.000	dead	.875	.117	1	7
	2	20.000	dead	.750	.153	2	6
	3	21.000	alive	.	.	2	5
	4	24.000	dead	.600	.182	3	4
	5	29.000	dead	.450	.188	4	3
	6	39.000	dead	.300	.175	5	2
	7	39.000	alive	.	.	5	1
	8	139.000	alive	.	.	5	0

Additional artery resection **Means and Medians for Survival Time**

	Mean				Median			
			95% Confidence Interval				95% Confidence Interval	
Additional artery resection	Estimate	Std. Error	Lower Bound	Upper Bound	Estimate	Std. Error	Lower Bound	Upper Bound
no	42.474	6.404	29.922	55.026	30.000	11.830	6.814	53.186
yes	60.125	19.793	21.331	98.919	29.000	6.275	16.701	41.299
Overall	52.865	9.885	33.490	72.240	29.000	6.795	15.681	42.319

Overall Comparisons

	Chi-Square	df	Sig.
Log Rank (Mantel-Cox)	.028	1	.866

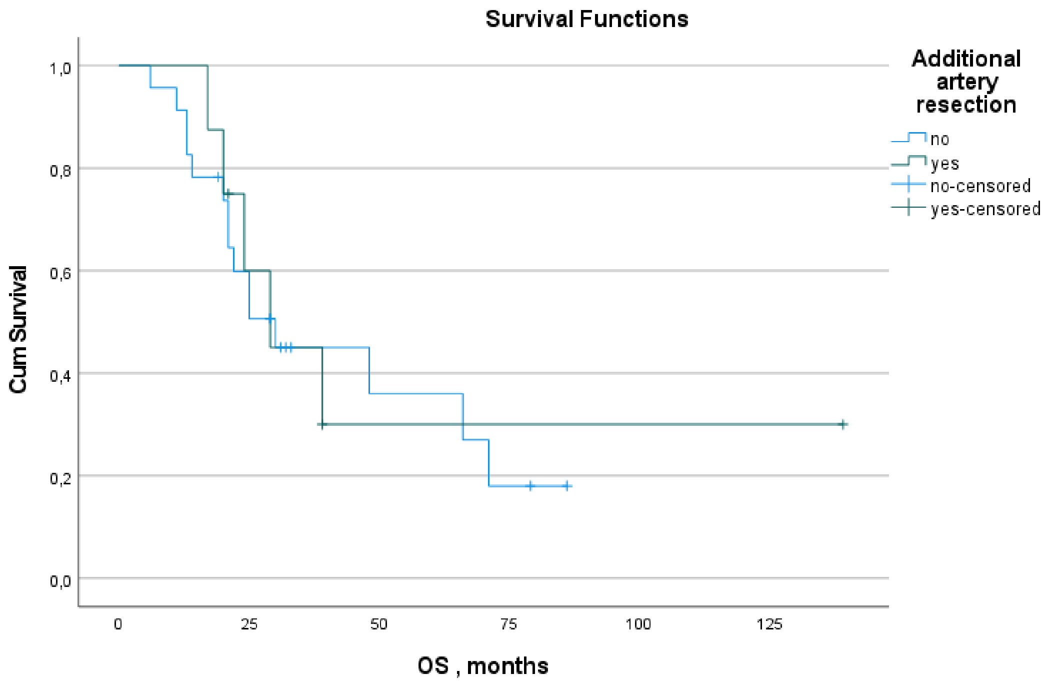

Kaplan-Meier

Case Processing Summary

NA Chemo	Total N	N of Events	Censored	
			N	Percent
no	13	10	3	23.1%
yes	18	10	8	44.4%
Overall	31	20	11	35.5%

Survival Table

NA Chemo		Time	Status	Cumulative Proportion Surviving at the Time		N of Cumulative Events	N of Remaining Cases
				Estimate	Std. Error		
no	1	6.000	dead	.923	.074	1	12
	2	11.000	dead	.846	.100	2	11
	3	13.000	dead	.	.	3	10
	4	13.000	dead	.692	.128	4	9
	5	20.000	dead	.615	.135	5	8
	6	21.000	dead	.538	.138	6	7
	7	25.000	dead	.462	.138	7	6
	8	30.000	dead	.385	.135	8	5
	9	33.000	alive	.	.	8	4
	10	39.000	dead	.288	.131	9	3
	11	66.000	dead	.192	.117	10	2
	12	79.000	alive	.	.	10	1
	13	86.000	alive	.	.	10	0

yes	1	14.000	dead	.944	.054	1	17
	2	17.000	dead	.889	.074	2	16
	3	19.000	alive	.	.	2	15
	4	20.000	dead	.830	.090	3	14
	5	21.000	dead	.770	.101	4	13
	6	21.000	alive	.	.	4	12
	7	22.000	dead	.706	.111	5	11
	8	24.000	dead	.642	.118	6	10
	9	25.000	dead	.578	.123	7	9
	10	29.000	dead	.514	.125	8	8
	11	29.000	alive	.	.	8	7
	12	29.000	alive	.	.	8	6
	13	31.000	alive	.	.	8	5
	14	32.000	alive	.	.	8	4
	15	39.000	alive	.	.	8	3
	16	48.000	dead	.342	.163	9	2
	17	71.000	dead	.171	.146	10	1
	18	139.000	alive	.	.	10	0

Yes and NO NA chemo Means and Medians for Survival Time .436 although for No NACHT it was 25 [95%CI:13.26;36.7] and for YES – 48 [95%CI:16.9;42.32]

	Mean				Median			
			95% Confidence Interval				95% Confidence Interval	
NA Chemo	Estimate	Std. Error	Lower Bound	Upper Bound	Estimate	Std. Error	Lower Bound	Upper Bound
no	37.327	8.086	21.479	53.175	25.000	5.991	13.257	36.743
yes	54.740	13.977	27.344	82.135	48.000	15.888	16.860	79.140
Overall	52.865	9.885	33.490	72.240	29.000	6.795	15.681	42.319

Overall Comparisons

	Chi-Square	df	Sig.
Log Rank (Mantel-Cox)	.606	1	.436

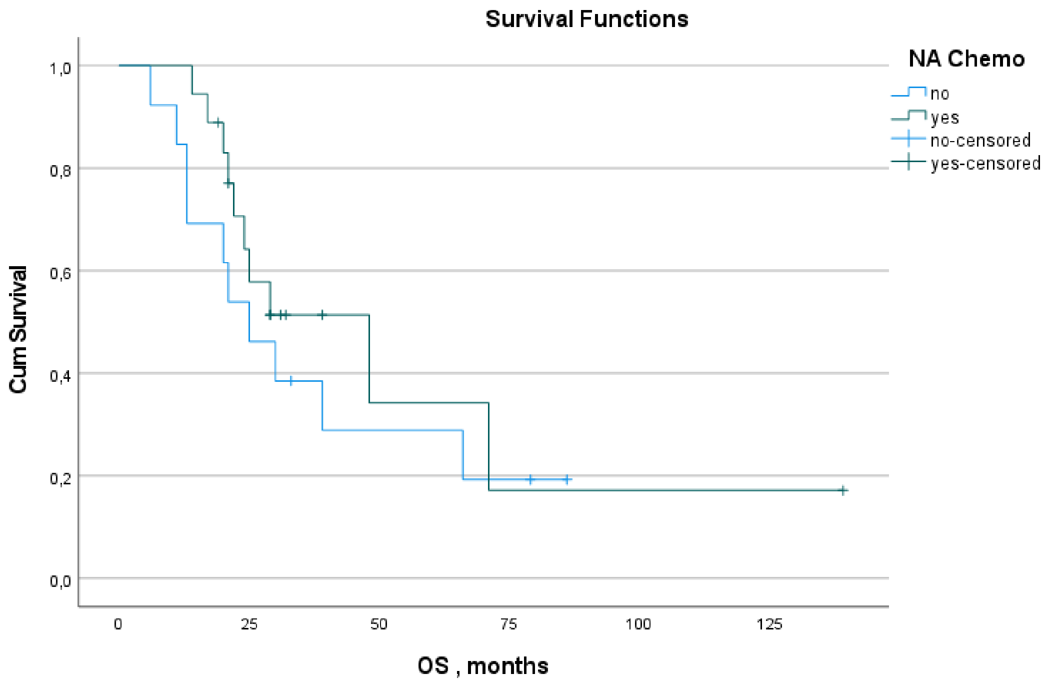

Kaplan-Meier

Case Processing Summary

Adj Chemo	Total N	N of Events	Censored N	Censored Percent
no	2	1	1	50.0%
yes	29	19	10	34.5%
Overall	31	20	11	35.5%

Survival Table

Adj Chemo		Time	Status	Cumulative Proportion Surviving at the Time: Estimate	Cumulative Proportion Surviving at the Time: Std. Error	N of Cumulative Events	N of Remaining Cases
no	1	6.000	dead	.500	.354	1	1
	2	139.000	alive	.	.	1	0
yes	1	11.000	dead	.966	.034	1	28
	2	13.000	dead	.	.	2	27
	3	13.000	dead	.897	.057	3	26
	4	14.000	dead	.862	.064	4	25
	5	17.000	dead	.828	.070	5	24
	6	19.000	alive	.	.	5	23
	7	20.000	dead	.	.	6	22
	8	20.000	dead	.756	.080	7	21
	9	21.000	dead	.	.	8	20
	10	21.000	dead	.684	.087	9	19

11	21.000	alive	.	.	9	18
12	22.000	dead	.646	.090	10	17
13	24.000	dead	.608	.093	11	16
14	25.000	dead	.	.	12	15
15	25.000	dead	.532	.095	13	14
16	29.000	dead	.494	.096	14	13
17	29.000	alive	.	.	14	12
18	29.000	alive	.	.	14	11
19	30.000	dead	.449	.097	15	10
20	31.000	alive	.	.	15	9
21	32.000	alive	.	.	15	8
22	33.000	alive	.	.	15	7
23	39.000	dead	.385	.102	16	6
24	39.000	alive	.	.	16	5
25	48.000	dead	.308	.107	17	4
26	66.000	dead	.231	.104	18	3
27	71.000	dead	.154	.094	19	2
28	79.000	alive	.	.	19	1
29	86.000	alive	.	.	19	0

Adj Chemo Means and Medians for Survival Time .643

Adj Chemo	Mean				Median			
			95% Confidence Interval				95% Confidence Interval	
	Estimate	Std. Error	Lower Bound	Upper Bound	Estimate	Std. Error	Lower Bound	Upper Bound
no	72.500	47.023	.000	164.664	6.000	.	.	.
yes	41.361	5.464	30.651	52.071	29.000	3.622	21.901	36.099
Overall	52.865	9.885	33.490	72.240	29.000	6.795	15.681	42.319

Overall Comparisons

	Chi-Square	df	Sig.
Log Rank (Mantel-Cox)	.215	1	.643

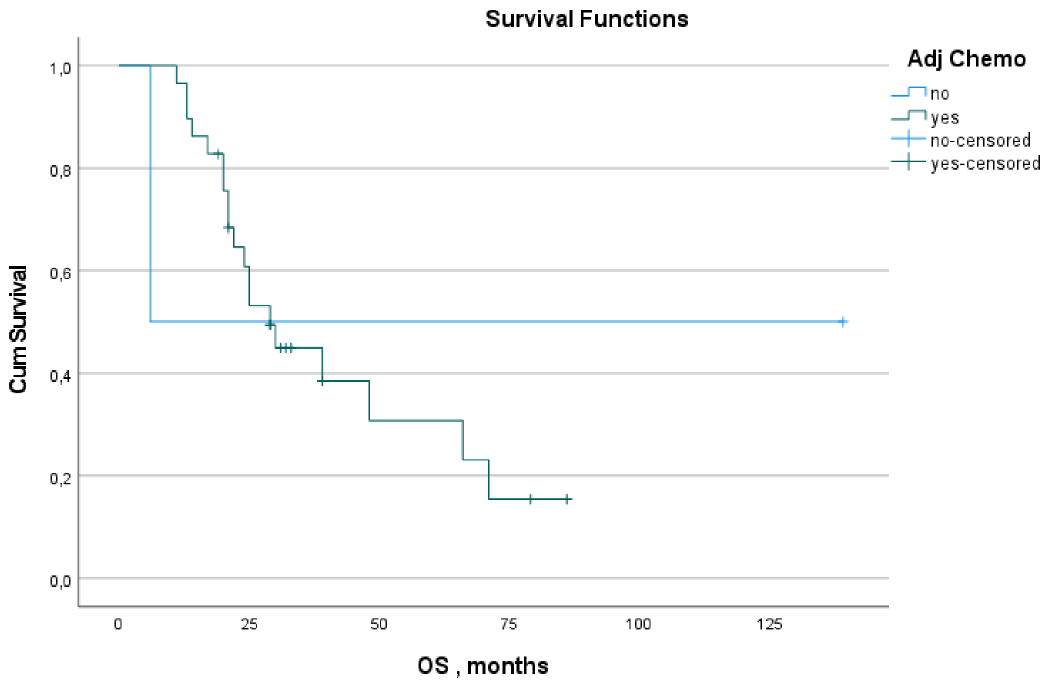

Kaplan-Meier

Case Processing Summary

Portal vein resection	Total N	N of Events	Censored N	Censored Percent
no	19	10	9	47.4%
yes	12	10	2	16.7%
Overall	31	20	11	35.5%

Survival Table

Portal vein resection		Time	Status	Cumulative Proportion Surviving at the Time Estimate	Cumulative Proportion Surviving at the Time Std. Error	N of Cumulative Events	N of Remaining Cases
no	1	11.000	dead	.947	.051	1	18
	2	13.000	dead	.	.	2	17
	3	13.000	dead	.842	.084	3	16
	4	14.000	dead	.789	.094	4	15
	5	17.000	dead	.737	.101	5	14
	6	19.000	alive	.	.	5	13
	7	21.000	alive	.	.	5	12
	8	25.000	dead	.	.	6	11
	9	25.000	dead	.614	.116	7	10
	10	29.000	dead	.553	.119	8	9
	11	29.000	alive	.	.	8	8
	12	29.000	alive	.	.	8	7

	13	31.000	alive	.	.	8	6
	14	33.000	alive	.	.	8	5
	15	39.000	alive	.	.	8	4
	16	48.000	dead	.414	.149	9	3
	17	66.000	dead	.276	.150	10	2
	18	79.000	alive	.	.	10	1
	19	139.000	alive	.	.	10	0
yes	1	6.000	dead	.917	.080	1	11
	2	20.000	dead	.	.	2	10
	3	20.000	dead	.750	.125	3	9
	4	21.000	dead	.	.	4	8
	5	21.000	dead	.583	.142	5	7
	6	22.000	dead	.500	.144	6	6
	7	24.000	dead	.417	.142	7	5
	8	30.000	dead	.333	.136	8	4
	9	32.000	alive	.	.	8	3
	10	39.000	dead	.222	.128	9	2
	11	71.000	dead	.111	.101	10	1
	12	86.000	alive	.	.	10	0

PV resection Means and Medians for Survival Time .289

no	48 [95%CI:7.733;88.267
yes	22{95%CI:16.9; 27.12

Portal vein resection	Mean			Median			
		95% Confidence Interval				95% Confidence Interval	
	Estimate	Lower Bound	Upper Bound	Estimate	Std. Error	Lower Bound	Upper Bound
no	62.588	33.502	91.673	48.000	20.544	7.733	88.267

yes	35.444	21.089	49.800	22.000	2.598	16.908	27.092
Overall	52.865	33.490	72.240	29.000	6.795	15.681	42.319

Overall Comparisons

	Chi-Square	df	Sig.
Log Rank (Mantel-Cox)	1.124	1	.289

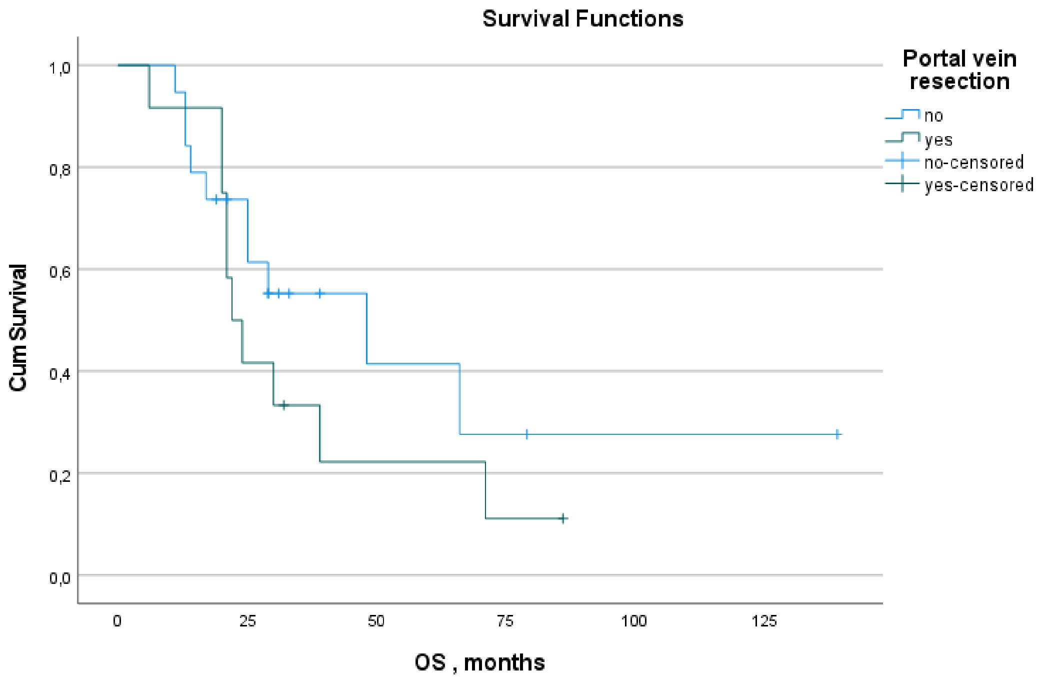

Kaplan-Meier

Case Processing Summary

Perineural invasion	Total N	N of Events	Censored	
			N	Percent
no	7	3	4	57.1%
yes	24	17	7	29.2%
Overall	31	20	11	35.5%

Survival Table

Perineural invasion		Time	Status	Cumulative Proportion Surviving at the Time Estimate	Cumulative Proportion Surviving at the Time Std. Error	N of Cumulative Events	N of Remaining Cases
no	1	14.000	dead	.857	.132	1	6
	2	17.000	dead	.714	.171	2	5
	3	19.000	alive	.	.	2	4
	4	21.000	dead	.536	.201	3	3
	5	21.000	alive	.	.	3	2
	6	39.000	alive	.	.	3	1
	7	139.000	alive	.	.	3	0
yes	1	6.000	dead	.958	.041	1	23
	2	11.000	dead	.917	.056	2	22
	3	13.000	dead	.	.	3	21
	4	13.000	dead	.833	.076	4	20
	5	20.000	dead	.	.	5	19
	6	20.000	dead	.750	.088	6	18
	7	21.000	dead	.708	.093	7	17
	8	22.000	dead	.667	.096	8	16
	9	24.000	dead	.625	.099	9	15
	10	25.000	dead	.	.	10	14
	11	25.000	dead	.542	.102	11	13
	12	29.000	dead	.500	.102	12	12
	13	29.000	alive	.	.	12	11
	14	29.000	alive	.	.	12	10
	15	30.000	dead	.450	.103	13	9
	16	31.000	alive	.	.	13	8
	17	32.000	alive	.	.	13	7
	18	33.000	alive	.	.	13	6
	19	39.000	dead	.375	.110	14	5
	20	48.000	dead	.300	.111	15	4
	21	66.000	dead	.225	.105	16	3
	22	71.000	dead	.150	.093	17	2
	23	79.000	alive	.	.	17	1
	24	86.000	alive	.	.	17	0

Perineural invasion Means and Medians for Survival Time .663

Perineural invasion	Mean				Median			
			95% Confidence Interval				95% Confidence Interval	
	Estimate	Std. Error	Lower Bound	Upper Bound	Estimate	Std. Error	Lower Bound	Upper Bound
no	82.643	24.304	35.006	130.280	.	.	.	.

yes	40.742	5.824	29.326	52.157	29.000	3.499	22.141	35.859
Overall	52.865	9.885	33.490	72.240	29.000	6.795	15.681	42.319

Overall Comparisons

	Chi-Square	df	Sig.
Log Rank (Mantel-Cox)	.190	1	.663

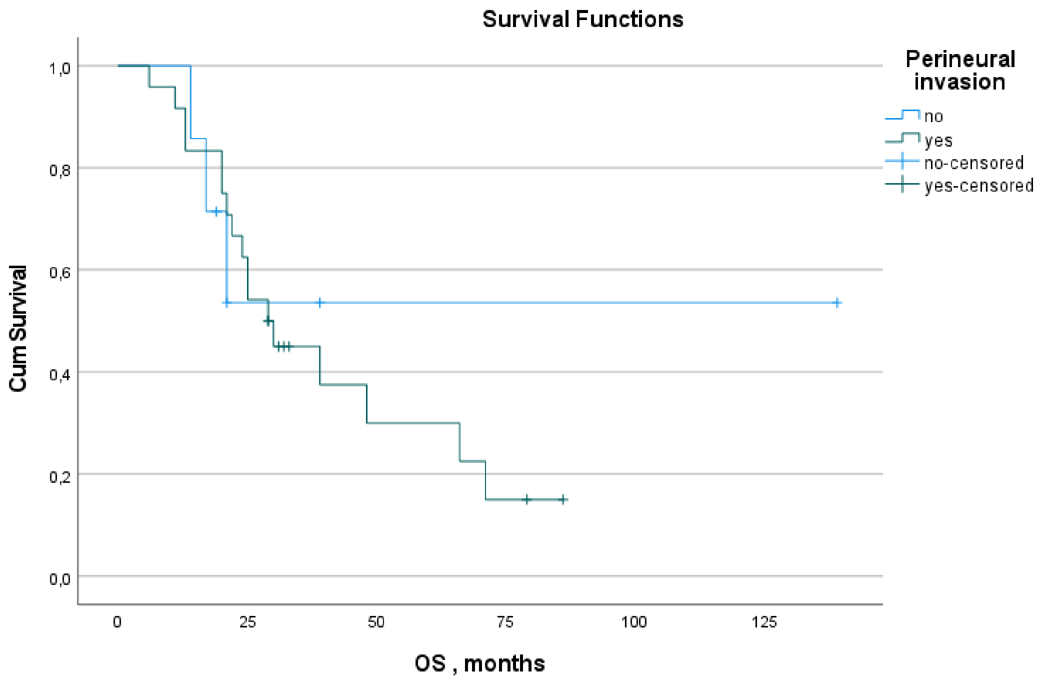

PFS

Kaplan-Meier

Case Processing Summary

Total N	N of Events	Censored	
		N	Percent
31	23	8	25.8%

Survival Table

	Time	Status	Cumulative Proportion Surviving at the Time		N of Cumulative Events	N of Remaining Cases
			Estimate	Std. Error		
1	4.000	yes	.968	.032	1	30
2	6.000	yes	.935	.044	2	29
3	9.000	yes	.903	.053	3	28
4	10.000	yes	.	.	4	27
5	10.000	yes	.839	.066	5	26
6	11.000	yes	.806	.071	6	25
7	12.000	yes	.774	.075	7	24

8	13.000	yes	.742	.079	8	23
9	14.000	yes	.	.	9	22
10	14.000	yes	.	.	10	21
11	14.000	yes	.645	.086	11	20
12	14.000	no	.	.	11	19
13	16.000	yes	.611	.088	12	18
14	17.000	yes	.	.	13	17
15	17.000	yes	.543	.090	14	16
16	17.000	no	.	.	14	15
17	18.000	yes	.	.	15	14
18	18.000	yes	.	.	16	13
19	18.000	yes	.435	.091	17	12
20	19.000	no	.	.	17	11
21	20.000	yes	.	.	18	10
22	20.000	yes	.	.	19	9
23	20.000	yes	.316	.088	20	8
24	21.000	no	.	.	20	7
25	29.000	no	.	.	20	6
26	38.000	yes	.263	.088	21	5
27	39.000	no	.	.	21	4
28	52.000	yes	.198	.087	22	3
29	66.000	yes	.132	.079	23	2
30	86.000	no	.	.	23	1
31	139.000	no	.	.	23	0

PFS Means and Medians for Survival Time 18 [95%CI: 15.98;20.03]

Mean				Median			
		95% Confidence Interval				95% Confidence Interval	
Estimate	Std. Error	Lower Bound	Upper Bound	Estimate	Std. Error	Lower Bound	Upper Bound
37.879	8.951	20.334	55.423	18.000	1.036	15.970	20.030

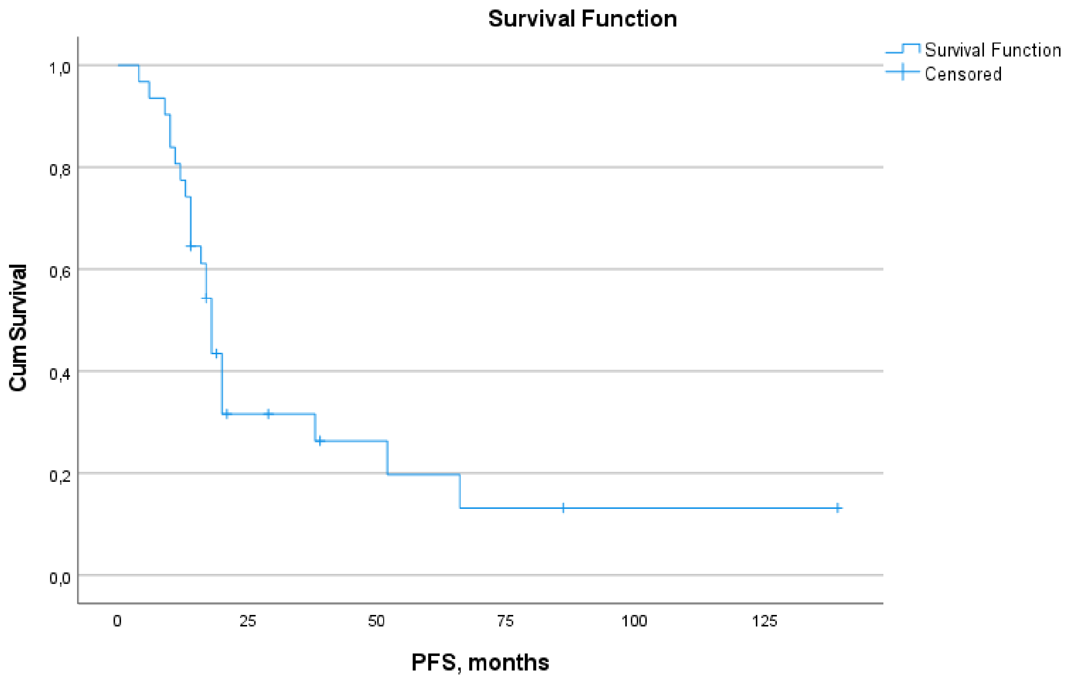

Kaplan-Meier

Case Processing Summary

Gender	Total N	N of Events	Censored N	Censored Percent
f	16	12	4	25.0%
m	15	11	4	26.7%
Overall	31	23	8	25.8%

Survival Table

Gender		Time	Status	Cumulative Proportion Surviving at the Time Estimate	Cumulative Proportion Surviving at the Time Std. Error	N of Cumulative Events	N of Remaining Cases
f	1	4.000	yes	.938	.061	1	15
	2	10.000	yes	.875	.083	2	14
	3	12.000	yes	.813	.098	3	13
	4	14.000	yes	.	.	4	12
	5	14.000	yes	.688	.116	5	11
	6	14.000	no	.	.	5	10
	7	17.000	yes	.619	.123	6	9
	8	18.000	yes	.550	.127	7	8
	9	19.000	no	.	.	7	7
	10	20.000	yes	.	.	8	6
	11	20.000	yes	.	.	9	5
	12	20.000	yes	.314	.126	10	4
	13	29.000	no	.	.	10	3

	14	52.000	yes	.210	.120	11	2
	15	66.000	yes	.105	.095	12	1
	16	139.000	no	.	.	12	0
m	1	6.000	yes	.933	.064	1	14
	2	9.000	yes	.867	.088	2	13
	3	10.000	yes	.800	.103	3	12
	4	11.000	yes	.733	.114	4	11
	5	13.000	yes	.667	.122	5	10
	6	14.000	yes	.600	.126	6	9
	7	16.000	yes	.533	.129	7	8
	8	17.000	yes	.467	.129	8	7
	9	17.000	no	.	.	8	6
	10	18.000	yes	.	.	9	5
	11	18.000	yes	.311	.124	10	4
	12	21.000	no	.	.	10	3
	13	38.000	yes	.207	.118	11	2
	14	39.000	no	.	.	11	1
	15	86.000	no	.	.	11	0

Gender Means and Medians for Survival Time .597

Gender	Mean				Median			
	Estimate	Std. Error	95% Confidence Interval Lower Bound	95% Confidence Interval Upper Bound	Estimate	Std. Error	95% Confidence Interval Lower Bound	95% Confidence Interval Upper Bound
f	37.419	11.227	15.414	59.425	20.000	1.069	17.906	22.094
m	30.978	8.136	15.031	46.925	17.000	1.784	13.504	20.496
Overall	37.879	8.951	20.334	55.423	18.000	1.036	15.970	20.030

Overall Comparisons

	Chi-Square	df	Sig.
Log Rank (Mantel-Cox)	.279	1	.597

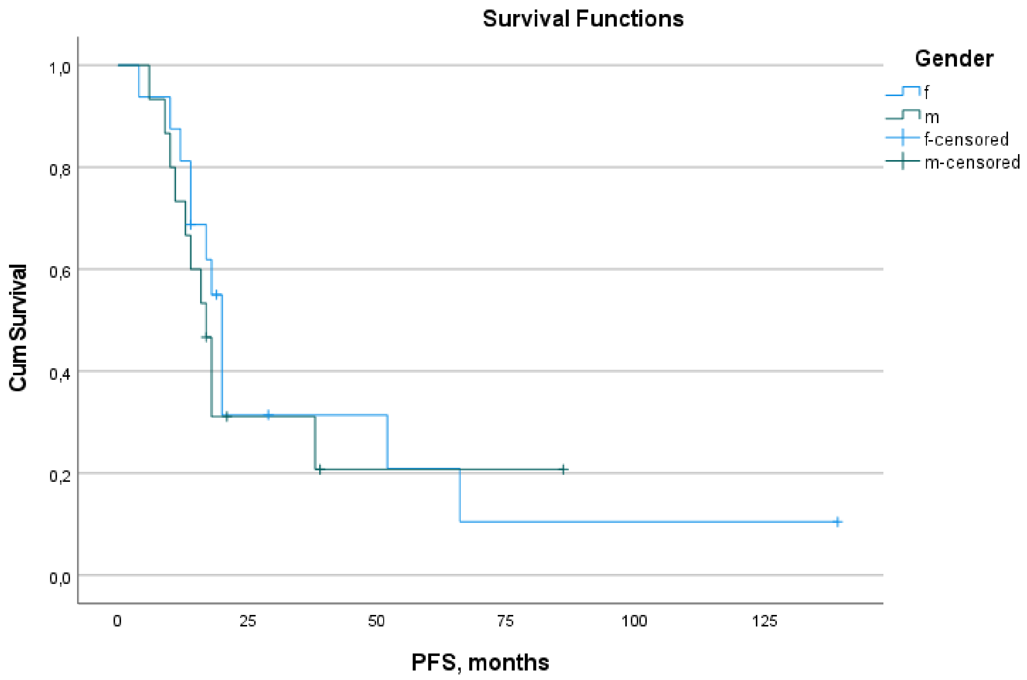

Kaplan-Meier

Case Processing Summary

Additional artery resection	Total N	N of Events	Censored N	Censored Percent
no	23	19	4	17.4%
yes	8	4	4	50.0%
Overall	31	23	8	25.8%

Survival Table

Additional artery resection		Time	Status	Cumulative Proportion Surviving at the Time Estimate	Cumulative Proportion Surviving at the Time Std. Error	N of Cumulative Events	N of Remaining Cases
no	1	4.000	yes	.957	.043	1	22
	2	6.000	yes	.913	.059	2	21
	3	9.000	yes	.870	.070	3	20
	4	10.000	yes	.	.	4	19
	5	10.000	yes	.783	.086	5	18
	6	11.000	yes	.739	.092	6	17
	7	13.000	yes	.696	.096	7	16
	8	14.000	yes	.	.	8	15
	9	14.000	yes	.	.	9	14
	10	14.000	yes	.565	.103	10	13
	11	14.000	no	.	.	10	12
	12	17.000	yes	.	.	11	11
	13	17.000	yes	.471	.105	12	10

	14	18.000	yes	.	.	13	9
	15	18.000	yes	.377	.103	14	8
	16	19.000	no	.	.	14	7
	17	20.000	yes	.	.	15	6
	18	20.000	yes	.269	.098	16	5
	19	29.000	no	.	.	16	4
	20	38.000	yes	.202	.094	17	3
	21	52.000	yes	.135	.083	18	2
	22	66.000	yes	.067	.063	19	1
	23	86.000	no	.	.	19	0
yes	1	12.000	yes	.875	.117	1	7
	2	16.000	yes	.750	.153	2	6
	3	17.000	no	.	.	2	5
	4	18.000	yes	.600	.182	3	4
	5	20.000	yes	.450	.188	4	3
	6	21.000	no	.	.	4	2
	7	39.000	no	.	.	4	1
	8	139.000	no	.	.	4	0

Additional artery resection Means and Medians for Survival Time .130

No – 17[95%:12.6;21.39]. Yes – 20 [95%:15.1;20.03]

	Mean				Median			
			95% Confidence Interval				95% Confidence Interval	
Additional artery resection	Estimate	Std. Error	Lower Bound	Upper Bound	Estimate	Std. Error	Lower Bound	Upper Bound
no	26.299	5.186	16.136	36.463	17.000	2.239	12.612	21.388
yes	71.750	22.977	26.716	116.784	20.000	2.510	15.080	24.920
Overall	37.879	8.951	20.334	55.423	18.000	1.036	15.970	20.030

Overall Comparisons

	Chi-Square	df	Sig.
Log Rank (Mantel-Cox)	2.290	1	.130

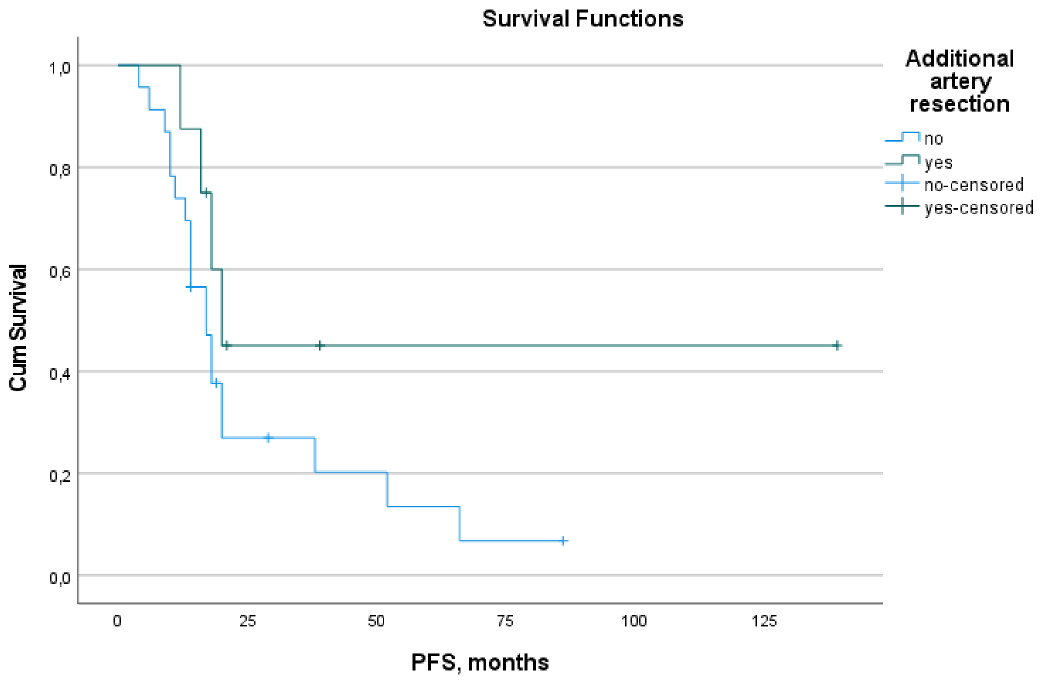

Kaplan-Meier

Case Processing Summary

NA Chemo	Total N	N of Events	Censored N	Censored Percent
no	13	12	1	7.7%
yes	18	11	7	38.9%
Overall	31	23	8	25.8%

Survival Table

NA Chemo		Time	Status	Cumulative Proportion Surviving at the Time Estimate	Cumulative Proportion Surviving at the Time Std. Error	N of Cumulative Events	N of Remaining Cases
no	1	4.000	yes	.923	.074	1	12
	2	6.000	yes	.846	.100	2	11
	3	9.000	yes	.769	.117	3	10
	4	10.000	yes	.692	.128	4	9
	5	12.000	yes	.615	.135	5	8
	6	13.000	yes	.538	.138	6	7
	7	16.000	yes	.462	.138	7	6
	8	17.000	yes	.	.	8	5
	9	17.000	yes	.308	.128	9	4
	10	20.000	yes	.231	.117	10	3
	11	38.000	yes	.154	.100	11	2
	12	52.000	yes	.077	.074	12	1
	13	86.000	no	.	.	12	0

yes	1	10.000	yes	.944	.054	1	17
	2	11.000	yes	.889	.074	2	16
	3	14.000	yes	.	.	3	15
	4	14.000	yes	.	.	4	14
	5	14.000	yes	.722	.106	5	13
	6	14.000	no	.	.	5	12
	7	17.000	no	.	.	5	11
	8	18.000	yes	.	.	6	10
	9	18.000	yes	.	.	7	9
	10	18.000	yes	.525	.124	8	8
	11	19.000	no	.	.	8	7
	12	20.000	yes	.	.	9	6
	13	20.000	yes	.375	.126	10	5
	14	21.000	no	.	.	10	4
	15	29.000	no	.	.	10	3
	16	39.000	no	.	.	10	2
	17	66.000	yes	.188	.147	11	1
	18	139.000	no	.	.	11	0

NA Chemo Means and Medians for Survival Time .078

No – 16[95%CI:11.6;20.4]. Yes – 20 [95%CI:15.7;24.27]

	Mean				Median			
			95% Confidence Interval				95% Confidence Interval	
NA Chemo	Estimate	Std. Error	Lower Bound	Upper Bound	Estimate	Std. Error	Lower Bound	Upper Bound
no	23.077	6.154	11.015	35.139	16.000	2.247	11.596	20.404
yes	48.503	14.526	20.031	76.975	20.000	2.177	15.734	24.266
Overall	37.879	8.951	20.334	55.423	18.000	1.036	15.970	20.030

Overall Comparisons

	Chi-Square	df	Sig.
Log Rank (Mantel-Cox)	3.097	1	.078

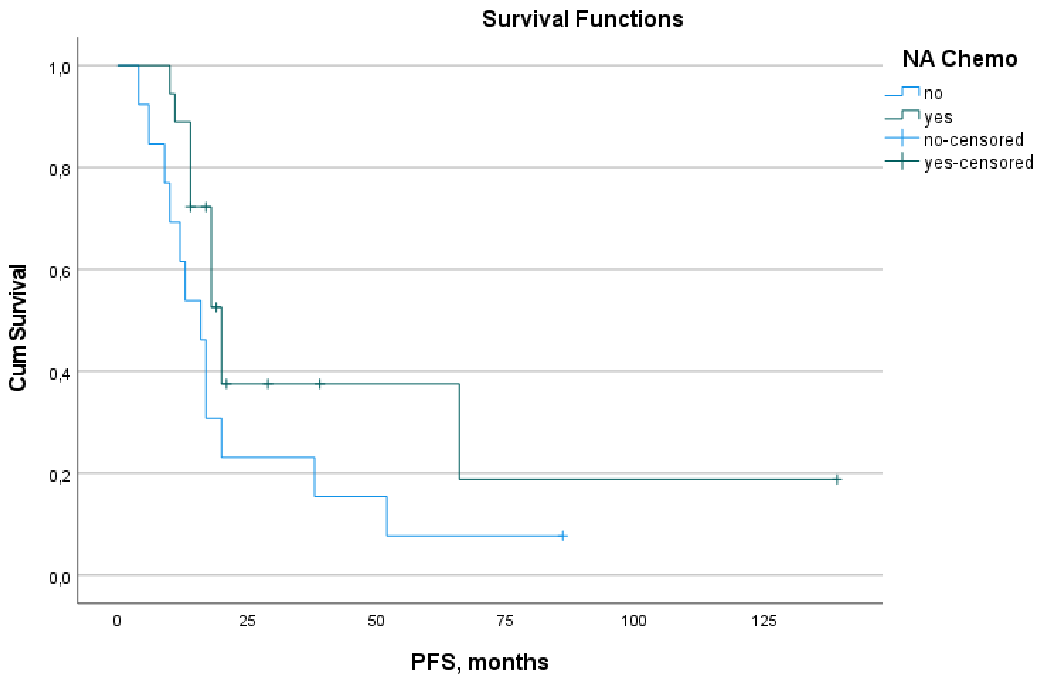

Kaplan-Meier

Case Processing Summary

Adj Chemo	Total N	N of Events	Censored	
			N	Percent
no	2	1	1	50.0%
yes	29	22	7	24.1%
Overall	31	23	8	25.8%

Survival Table

Adj Chemo		Time	Status	Cumulative Proportion Surviving at the Time		N of Cumulative Events	N of Remaining Cases
				Estimate	Std. Error		
no	1	4.000	yes	.500	.354	1	1
	2	139.000	no	.	.	1	0
yes	1	6.000	yes	.966	.034	1	28
	2	9.000	yes	.931	.047	2	27
	3	10.000	yes	.	.	3	26
	4	10.000	yes	.862	.064	4	25
	5	11.000	yes	.828	.070	5	24
	6	12.000	yes	.793	.075	6	23
	7	13.000	yes	.759	.079	7	22
	8	14.000	yes	.	.	8	21
	9	14.000	yes	.	.	9	20
	10	14.000	yes	.655	.088	10	19
	11	14.000	no	.	.	10	18

	12	16.000	yes	.619	.091	11	17
	13	17.000	yes	.	.	12	16
	14	17.000	yes	.546	.093	13	15
	15	17.000	no	.	.	13	14
	16	18.000	yes	.	.	14	13
	17	18.000	yes	.	.	15	12
	18	18.000	yes	.429	.095	16	11
	19	19.000	no	.	.	16	10
	20	20.000	yes	.	.	17	9
	21	20.000	yes	.	.	18	8
	22	20.000	yes	.300	.091	19	7
	23	21.000	no	.	.	19	6
	24	29.000	no	.	.	19	5
	25	38.000	yes	.240	.090	20	4
	26	39.000	no	.	.	20	3
	27	52.000	yes	.160	.089	21	2
	28	66.000	yes	.080	.072	22	1
	29	86.000	no	.	.	22	0

Means and Medians for Survival Time

Adj Chemo	Mean				Median			
			95% Confidence Interval				95% Confidence Interval	
	Estimate	Std. Error	Lower Bound	Upper Bound	Estimate	Std. Error	Lower Bound	Upper Bound
no	71.500	47.730	.000	165.050	4.000	.	.	.
yes	29.014	4.970	19.273	38.756	18.000	.998	16.044	19.956
Overall	37.879	8.951	20.334	55.423	18.000	1.036	15.970	20.030

Overall Comparisons

	Chi-Square	df	Sig.
Log Rank (Mantel-Cox)	.479	1	.489

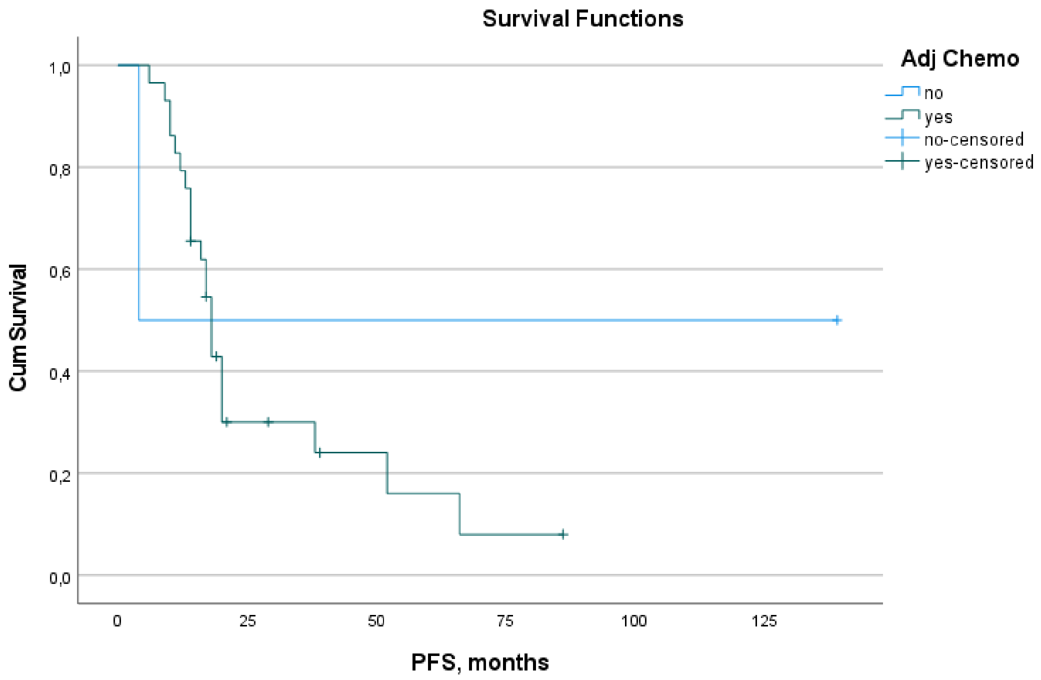

Kaplan-Meier

Case Processing Summary

Portal vein resection	Total N	N of Events	Censored N	Censored Percent
no	19	12	7	36.8%
yes	12	11	1	8.3%
Overall	31	23	8	25.8%

Survival Table

Portal vein resection		Time	Status	Cumulative Proportion Surviving at the Time: Estimate	Cumulative Proportion Surviving at the Time: Std. Error	N of Cumulative Events	N of Remaining Cases
no	1	6.000	yes	.947	.051	1	18
	2	9.000	yes	.895	.070	2	17
	3	10.000	yes	.	.	3	16
	4	10.000	yes	.789	.094	4	15
	5	11.000	yes	.737	.101	5	14
	6	13.000	yes	.684	.107	6	13
	7	14.000	yes	.632	.111	7	12
	8	14.000	no	.	.	7	11
	9	17.000	no	.	.	7	10
	10	19.000	no	.	.	7	9
	11	20.000	yes	.	.	8	8
	12	20.000	yes	.	.	9	7

	13	20.000	yes	.421	.124	10	6
	14	21.000	no	.	.	10	5
	15	29.000	no	.	.	10	4
	16	38.000	yes	.316	.130	11	3
	17	39.000	no	.	.	11	2
	18	52.000	yes	.158	.129	12	1
	19	139.000	no	.	.	12	0
yes	1	4.000	yes	.917	.080	1	11
	2	12.000	yes	.833	.108	2	10
	3	14.000	yes	.	.	3	9
	4	14.000	yes	.667	.136	4	8
	5	16.000	yes	.583	.142	5	7
	6	17.000	yes	.	.	6	6
	7	17.000	yes	.417	.142	7	5
	8	18.000	yes	.	.	8	4
	9	18.000	yes	.	.	9	3
	10	18.000	yes	.167	.108	10	2
	11	66.000	yes	.083	.080	11	1
	12	86.000	no	.	.	11	0

PV resection Means and Medians for Survival Time .308

No – 20[95%CI:13.1;26.91]. Yes – 17[95%CI:15.37;18.68]

	Mean				Median			
			95% Confidence Interval				95% Confidence Interval	
Portal vein resection	Estimate	Std. Error	Lower Bound	Upper Bound	Estimate	Std. Error	Lower Bound	Upper Bound
no	42.211	13.775	15.211	69.210	20.000	3.524	13.092	26.908
yes	25.000	6.775	11.721	38.279	17.000	.854	15.326	18.674
Overall	37.879	8.951	20.334	55.423	18.000	1.036	15.970	20.030

Overall Comparisons

	Chi-Square	df	Sig.
Log Rank (Mantel-Cox)	1.040	1	.308

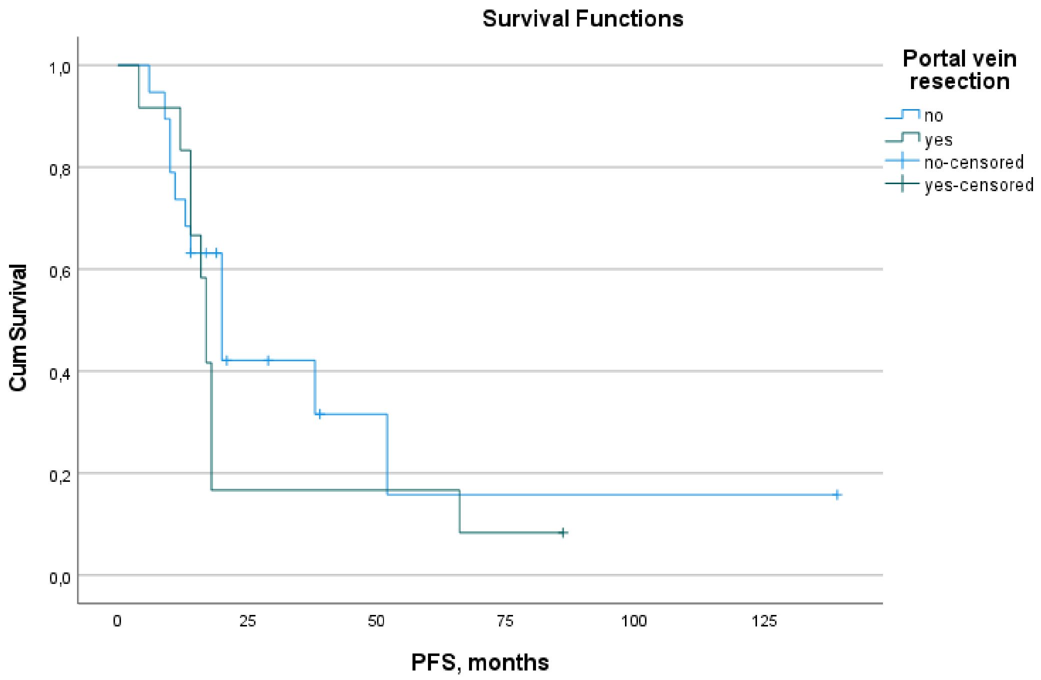

Kaplan-Meier

Case Processing Summary

Perineural invasion	Total N	N of Events	Censored	
			N	Percent
no	7	1	6	85.7%
yes	24	22	2	8.3%
Overall	31	23	8	25.8%

Survival Table

Perineural invasion		Time	Status	Cumulative Proportion Surviving at the Time		N of Cumulative Events	N of Remaining Cases
				Estimate	Std. Error		
no	1	14.000	no	.	.	0	6
	2	17.000	no	.	.	0	5
	3	18.000	yes	.800	.179	1	4
	4	19.000	no	.	.	1	3
	5	21.000	no	.	.	1	2
	6	39.000	no	.	.	1	1
	7	139.000	no	.	.	1	0
yes	1	4.000	yes	.958	.041	1	23
	2	6.000	yes	.917	.056	2	22
	3	9.000	yes	.875	.068	3	21
	4	10.000	yes	.	.	4	20
	5	10.000	yes	.792	.083	5	19
	6	11.000	yes	.750	.088	6	18

7	12.000	yes	.708	.093	7	17
8	13.000	yes	.667	.096	8	16
9	14.000	yes	.	.	9	15
10	14.000	yes	.	.	10	14
11	14.000	yes	.542	.102	11	13
12	16.000	yes	.500	.102	12	12
13	17.000	yes	.	.	13	11
14	17.000	yes	.417	.101	14	10
15	18.000	yes	.	.	15	9
16	18.000	yes	.333	.096	16	8
17	20.000	yes	.	.	17	7
18	20.000	yes	.	.	18	6
19	20.000	yes	.208	.083	19	5
20	29.000	no	.	.	19	4
21	38.000	yes	.156	.077	20	3
22	52.000	yes	.104	.067	21	2
23	66.000	yes	.052	.050	22	1
24	86.000	no	.	.	22	0

Perineural invasion Means and Medians for Survival Time .007

	Mean				Median			
			95% Confidence Interval				95% Confidence Interval	
Perineural invasion	Estimate	Std. Error	Lower Bound	Upper Bound	Estimate	Std. Error	Lower Bound	Upper Bound
no	114.800	21.645	72.376	157.224	.	.	.	.
yes	23.563	4.366	15.005	32.120	16.000	1.633	12.799	19.201
Overall	37.879	8.951	20.334	55.423	18.000	1.036	15.970	20.030

Overall Comparisons

	Chi-Square	df	Sig.
Log Rank (Mantel-Cox)	7.321	1	.007

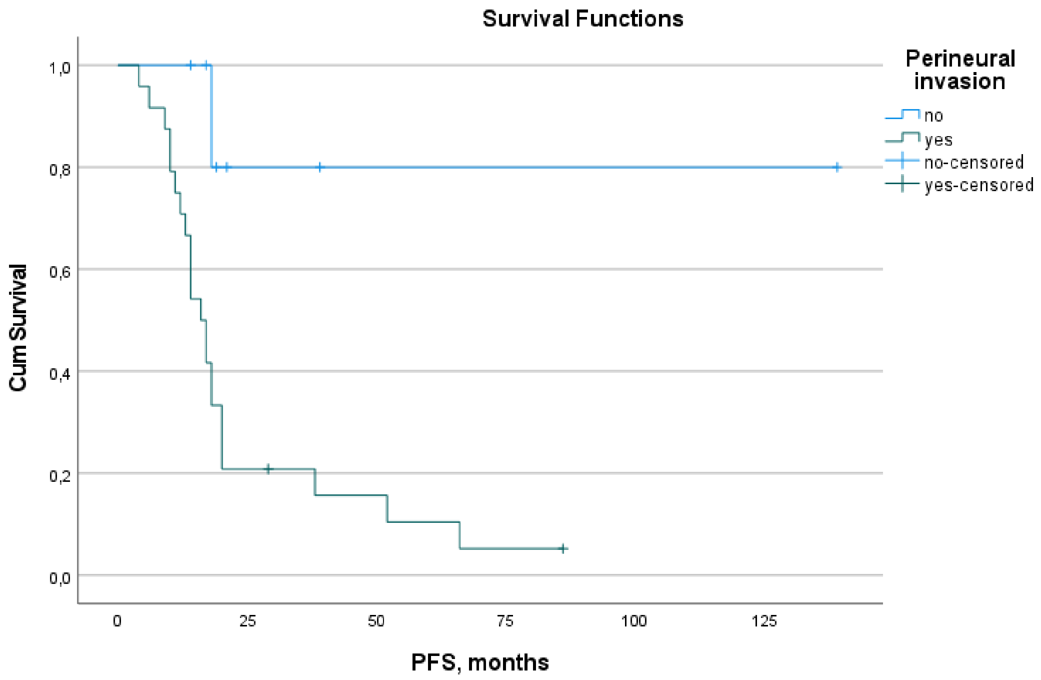

Kaplan-Meier

Case Processing Summary

R-status	Total N	N of Events	Censored	
			N	Percent
no	28	20	8	28.6%
yes	3	3	0	0.0%
Overall	31	23	8	25.8%

Survival Table

R-status		Time	Status	Cumulative Proportion Surviving at the Time		N of Cumulative Events	N of Remaining Cases
				Estimate	Std. Error		
no	1	4.000	yes	.964	.035	1	27
	2	6.000	yes	.929	.049	2	26
	3	9.000	yes	.893	.058	3	25
	4	10.000	yes	.	.	4	24
	5	10.000	yes	.821	.072	5	23
	6	11.000	yes	.786	.078	6	22
	7	12.000	yes	.750	.082	7	21
	8	13.000	yes	.714	.085	8	20
	9	14.000	yes	.	.	9	19
	10	14.000	yes	.	.	10	18
	11	14.000	yes	.607	.092	11	17
	12	14.000	no	.	.	11	16

	13	16.000	yes	.569	.094	12	15
	14	17.000	yes	.531	.095	13	14
	15	17.000	no	.	.	13	13
	16	18.000	yes	.	.	14	12
	17	18.000	yes	.	.	15	11
	18	18.000	yes	.409	.096	16	10
	19	19.000	no	.	.	16	9
	20	20.000	yes	.	.	17	8
	21	20.000	yes	.	.	18	7
	22	20.000	yes	.272	.091	19	6
	23	21.000	no	.	.	19	5
	24	29.000	no	.	.	19	4
	25	39.000	no	.	.	19	3
	26	66.000	yes	.182	.096	20	2
	27	86.000	no	.	.	20	1
	28	139.000	no	.	.	20	0
yes	1	17.000	yes	.667	.272	1	2
	2	38.000	yes	.333	.272	2	1
	3	52.000	yes	.000	.000	3	0

R-status Means and Medians for Survival Time .857

	Mean				Median			
			95% Confidence Interval				95% Confidence Interval	
R-status	Estimate	Std. Error	Lower Bound	Upper Bound	Estimate	Std. Error	Lower Bound	Upper Bound
no	41.601	10.564	20.896	62.306	18.000	1.195	15.657	20.343
yes	35.667	10.171	15.732	55.601	38.000	17.146	4.393	71.607
Overall	37.879	8.951	20.334	55.423	18.000	1.036	15.970	20.030

Overall Comparisons

	Chi-Square	df	Sig.
Log Rank (Mantel-Cox)	.032	1	.857

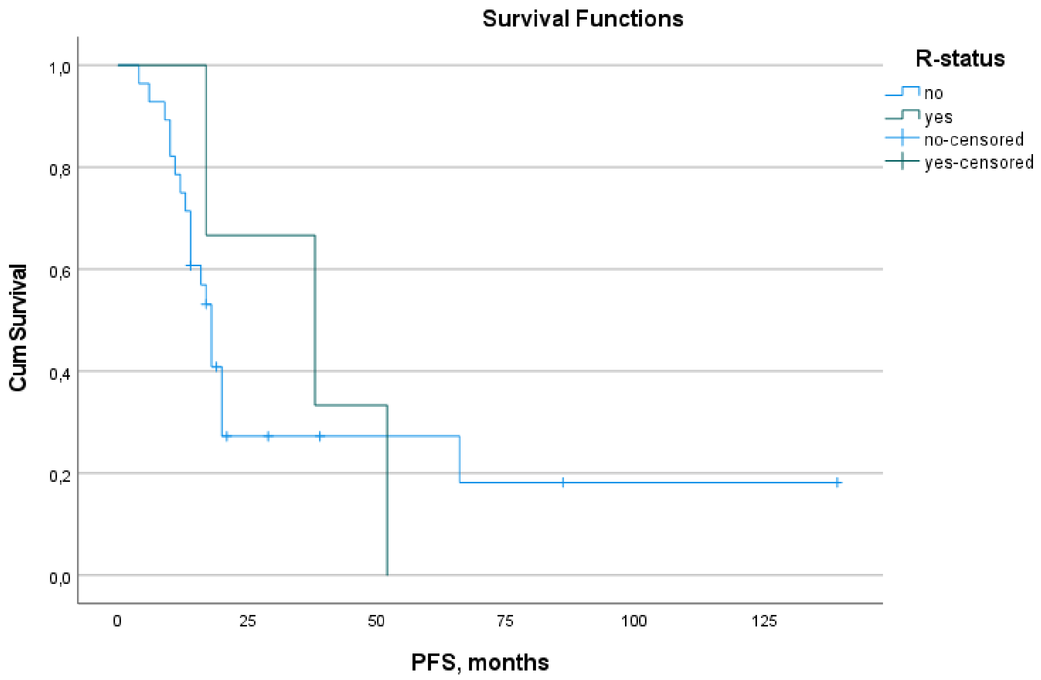

Survival Functions
R-status
no
yes
no-censored
yes-censored
Cum Survival
1,0
0,8
0,6
0,4
0,2
0,0
0
25
50
75
100
125
PFS, months
