# Supplementary material for: Hemodynamic, Surgical and Oncological Outcomes of 40 Distal Pancreatectomies with Celiac and Left Gastric Arteries Resection (DP CAR) without Arterial Reconstructions and Preoperative Embolization
Source: Cancers (Basel). 2022 Feb 28;14(5):1254. doi: 10.3390/cancers14051254 (PMC8909059; doi:10.3390/cancers14051254)
Supplement: Supplementary file 1 [file cancers-14-01254-s001.zip › cancers-1582412-supplementary/Supplement/File S2. Statistics 2 for PDAC survival.pdf]

File S2. Statistics. Part 2. Pancreatic ductal adenocarcinoma patients' survival.

Frequencies

Dindo-C

		Frequency	Per cent	Valid Percent	Cumulative Percent
Valid	0	22	71.0	71.0	71.0
	1	3	9.7	9.7	80.6
	2	6	19.4	19.4	100.0
	Total	31	100.0	100.0	

Frequencies

LN. involved

		Frequency	Per cent	Valid Percent	Cumulative Percent
Valid	0	7	22.6	22.6	22.6
	1	17	54.8	54.8	77.4
	2	7	22.6	22.6	100.0
	Total	31	100.0	100.0	

Frequencies

CA 19-9 before surgery

		Frequency	Per cent	Valid Percent	Cumulative Percent
Valid	<= 66	16	51.6	51.6	51.6
	67+	15	48.4	48.4	100.0
	Total	31	100.0	100.0	

Frequencies

Tumour size. Pathol. mm

		Frequency	Per cent	Valid Percent	Cumulative Percent
Valid	<= 43	16	51.6	51.6	51.6
	44+	15	48.4	48.4	100.0
	Total	31	100.0	100.0	

Kaplan-Meier

Case Processing Summary

CA 19-9 before surgery	Total N	N of Events	Censored	
			N	Per cent
<= 66	16	8	8	50.0%
67+	15	12	3	20.0%
Overall	31	20	11	35.5%

Survival Table

CA 19-9 before surgery		Time	Status	Cumulative Proportion Surviving at the Time Estimate	Std. Error	N of Cumulative Events	N of Remaining Cases
<= 66	1	13.000	dead	.938	.061	1	15
	2	14.000	dead	.875	.083	2	14
	3	17.000	dead	.813	.098	3	13
	4	19.000	alive	.	.	3	12
	5	21.000	alive	.	.	3	11
	6	25.000	dead	.	.	4	10
	7	25.000	dead	.665	.124	5	9
	8	29.000	dead	.591	.130	6	8
	9	29.000	alive	.	.	6	7
	10	29.000	alive	.	.	6	6
	11	31.000	alive	.	.	6	5
	12	32.000	alive	.	.	6	4
	13	39.000	alive	.	.	6	3
	14	48.000	dead	.394	.183	7	2
	15	71.000	dead	.197	.167	8	1
	16	139.000	alive	.	.	8	0
67+	1	6000	dead	.933	.064	1	14
	2	11.000	dead	.867	.088	2	13
	3	13.000	dead	.800	.103	3	12
	4	20.000	dead	.	.	4	11
	5	20.000	dead	.667	.122	5	10
	6	21.000	dead	.	.	6	9
	7	21.000	dead	.533	.129	7	8
	8	22.000	dead	.467	.129	8	7
	9	24.000	dead	.400	.126	9	6
	10	30.000	dead	.333	.122	10	5
	11	33.000	alive	.	.	10	4
	12	39.000	dead	.250	.116	11	3
	13	66.000	dead	.167	.103	12	2
	14	79.000	alive	.	.	12	1
	15	86.000	alive	.	.	12	0

CA 19-9 before surgery Means and Medians for Survival Time .198

> 66 – 48 [95%CI:13.45;82.549]. >66 – 22[95%CI:18.2;25.8]

CA 19-9 before CT	Mean		Median	
		95% Confidence Interval		95% Confidence Interval

	Estimate	Std. Error	Lower Bound	Upper Bound	Estimate	Std. Error	Lower Bound	Upper Bound
<= 66	59.403	15.655	28.720	90.087	48.000	17.627	13.451	82.549
67+	35.617	7068	21.763	49.470	22.000	1932	18.213	25.787
Overall	52.865	9885	33.490	72.240	29.000	6795	15.681	42.319

Overall Comparisons

	Chi-Square	df	Sig.
Log Rank (Mantel-Cox)	1655	1	.198

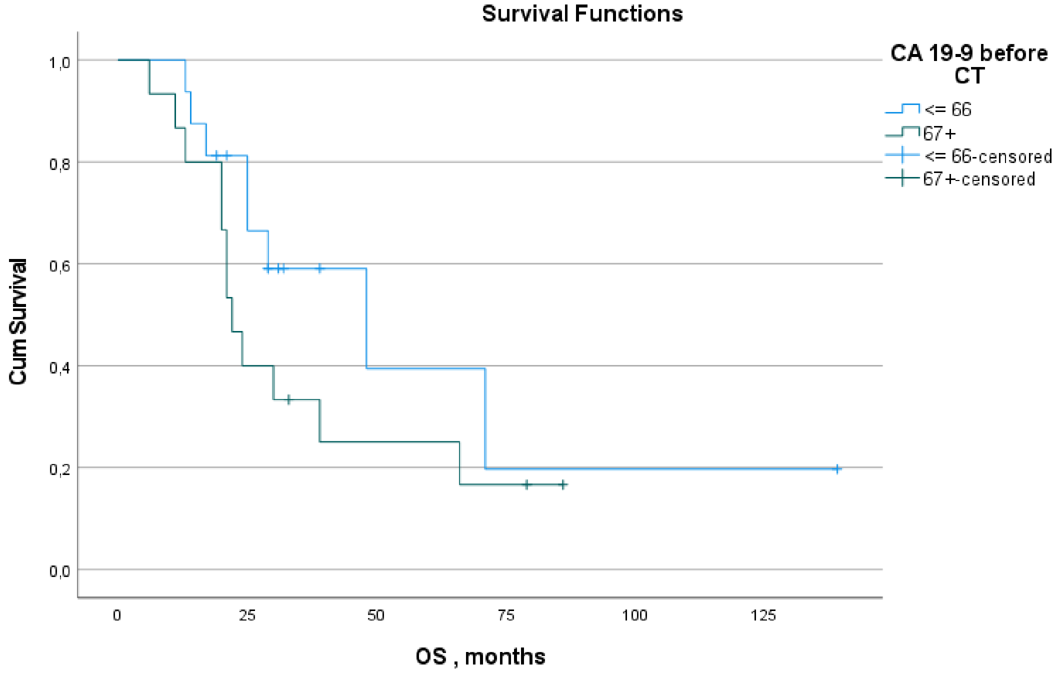

CA 19-9 before surgery= CA 19-9 before CT (mistake in writing)

Kaplan-Meier

Case Processing Summary

Tumour size. Pathol. mm	Total N	N of Events	Censored N	Censored Per cent
<= 43	16	10	6	37.5%
44+	15	10	5	33.3%
Overall	31	20	11	35.5%

Survival Table

Tumour size. Pathol. mm		Time	Status	Cumulative Proportion Surviving at the Time Estimate	Cumulative Proportion Surviving at the Time Std. Error	N of Cumulative Events	N of Remaining Cases
<= 43	1	6000	dead	.938	.061	1	15
	2	13.000	dead	.875	.083	2	14
	3	14.000	dead	.813	.098	3	13

	4	20.000	dead	.	.	4	12
	5	20.000	dead	.688	.116	5	11
	6	21.000	dead	.625	.121	6	10
	7	21.000	alive	.	.	6	9
	8	25.000	dead	.	.	7	8
	9	25.000	dead	.486	.128	8	7
	10	29.000	dead	.417	.127	9	6
	11	29.000	alive	.	.	9	5
	12	31.000	alive	.	.	9	4
	13	32.000	alive	.	.	9	3
	14	39.000	alive	.	.	9	2
	15	48.000	dead	.208	.160	10	1
	16	139.000	alive	.	.	10	0
44+	1	11.000	dead	.933	.064	1	14
	2	13.000	dead	.867	.088	2	13
	3	17.000	dead	.800	.103	3	12
	4	19.000	alive	.	.	3	11
	5	21.000	dead	.727	.117	4	10
	6	22.000	dead	.655	.126	5	9
	7	24.000	dead	.582	.131	6	8
	8	29.000	alive	.	.	6	7
	9	30.000	dead	.499	.136	7	6
	10	33.000	alive	.	.	7	5
	11	39.000	dead	.399	.141	8	4
	12	66.000	dead	.299	.136	9	3
	13	71.000	dead	.199	.122	10	2
	14	79.000	alive	.	.	10	1
	15	86.000	alive	.	.	10	0

Tumor size. pathol **Means and Medians for Survival Time** .721

< 43 – 25 [95%CI:15.37;34.63]. >43 - 30[95%CI;8.1;51.89]

Tumour size. Pathol. mm	Mean				Median			
	Estimate	Std. Error	95% Confidence Interval		Estimate	Std. Error	95% Confidence Interval	
			Lower Bound	Upper Bound			Lower Bound	Upper Bound
<= 43	50.319	16.400	18.175	82.464	25.000	4912	15.372	34.628
44+	44.809	7769	29.583	60.036	30.000	11.171	8105	51.895
Overall	52.865	9885	33.490	72.240	29.000	6795	15.681	42.319

Overall Comparisons

	Chi-Square	df	Sig.
Log Rank (Mantel-Cox)	.127	1	.721

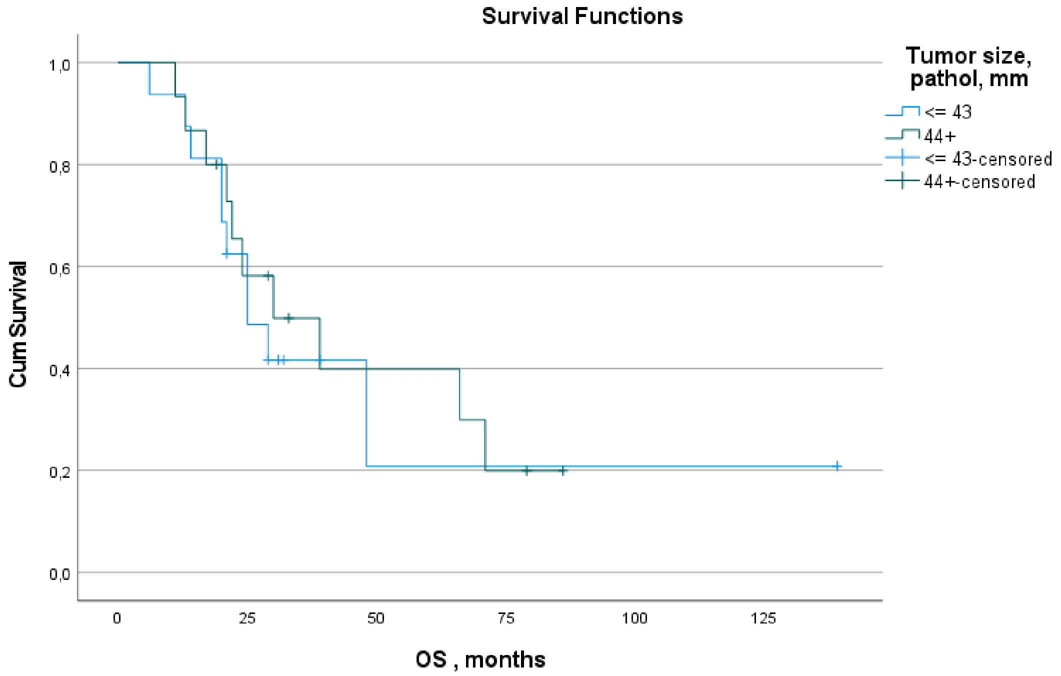

Kaplan-Meier

Case Processing Summary

Dindo-C	Total N	N of Events	Censored N	Censored Per cent
0	22	14	8	36.4%
1	4	2	2	50.0%
2	5	4	1	20.0%
Overall	31	20	11	35.5%

Survival Table

Dindo-C		Time	Status	Cumulative Proportion Surviving at the Time: Estimate	Cumulative Proportion Surviving at the Time: Std. Error	N of Cumulative Events	N of Remaining Cases
0	1	11.000	dead	.955	.044	1	21
	2	13.000	dead	.	.	2	20
	3	13.000	dead	.864	.073	3	19
	4	19.000	alive	.	.	3	18
	5	20.000	dead	.816	.083	4	17
	6	21.000	dead	.	.	5	16
	7	21.000	dead	.720	.097	6	15
	8	21.000	alive	.	.	6	14
	9	24.000	dead	.668	.103	7	13

	10	25.000	dead	.	.	8	12
	11	25.000	dead	.565	.110	9	11
	12	29.000	dead	.514	.111	10	10
	13	29.000	alive	.	.	10	9
	14	29.000	alive	.	.	10	8
	15	30.000	dead	.450	.114	11	7
	16	32.000	alive	.	.	11	6
	17	33.000	alive	.	.	11	5
	18	39.000	dead	.360	.122	12	4
	19	39.000	alive	.	.	12	3
	20	66.000	dead	.240	.127	13	2
	21	71.000	dead	.120	.106	14	1
	22	139.000	alive	.	.	14	0
1	1	17.000	dead	.750	.217	1	3
	2	31.000	alive	.	.	1	2
	3	48.000	dead	.375	.286	2	1
	4	86.000	alive	.	.	2	0
2	1	6000	dead	.800	.179	1	4
	2	14.000	dead	.600	.219	2	3
	3	20.000	dead	.400	.219	3	2
	4	22.000	dead	.200	.179	4	1
	5	79.000	alive	.	.	4	0

Means and Medians for Survival Time.425

	Mean				Median			
			95% Confidence Interval				95% Confidence Interval	
Dindo-C	Estimate	Std. Error	Lower Bound	Upper Bound	Estimate	Std. Error	Lower Bound	Upper Bound
0	48.494	10.613	27.693	69.295	30.000	4947	20.305	39.695
1	54.500	14.789	25.513	83.487	48.000	23.677	1594	94.406
2	28.200	11.629	5407	50.993	20.000	6573	7118	32.882
Overall	52.865	9885	33.490	72.240	29.000	6795	15.681	42.319

Overall Comparisons

	Chi-Square	df	Sig.
Log Rank (Mantel-Cox)	1713	2	.425

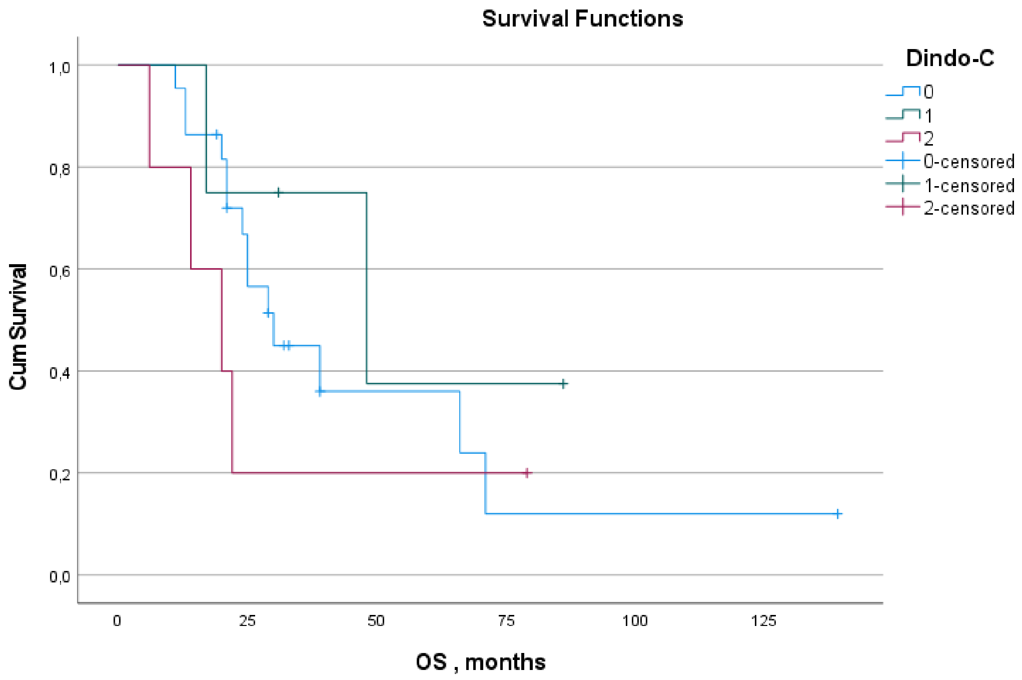

Kaplan-Meier

Case Processing Summary

LN. involved	Total N	N of Events	Censored N	Censored Per cent
0	7	1	6	85.7%
1	17	14	3	17.6%
2	7	5	2	28.6%
Overall	31	20	11	35.5%

Survival Table

LN. involved		Time	Status	Cumulative Proportion Surviving at the Time Estimate	Cumulative Proportion Surviving at the Time Std. Error	N of Cumulative Events	N of Remaining Cases
0	1	14.000	dead	.857	.132	1	6
	2	19.000	alive	.	.	1	5
	3	21.000	alive	.	.	1	4
	4	31.000	alive	.	.	1	3
	5	39.000	alive	.	.	1	2
	6	86.000	alive	.	.	1	1
	7	139.000	alive	.	.	1	0
1	1	11.000	dead	.941	.057	1	16
	2	13.000	dead	.	.	2	15
	3	13.000	dead	.824	.092	3	14
	4	17.000	dead	.765	.103	4	13
	5	20.000	dead	.	.	5	12

	6	20.000	dead	.647	.116	6	11
	7	21.000	dead	.588	.119	7	10
	8	22.000	dead	.529	.121	8	9
	9	25.000	dead	.	.	9	8
	10	25.000	dead	.412	.119	10	7
	11	29.000	dead	.353	.116	11	6
	12	29.000	alive	.	.	11	5
	13	32.000	alive	.	.	11	4
	14	33.000	alive	.	.	11	3
	15	39.000	dead	.235	.123	12	2
	16	48.000	dead	.118	.104	13	1
	17	71.000	dead	.000	.000	14	0
2	1	6000	dead	.857	.132	1	6
	2	21.000	dead	.714	.171	2	5
	3	24.000	dead	.571	.187	3	4
	4	29.000	alive	.	.	3	3
	5	30.000	dead	.381	.199	4	2
	6	66.000	dead	.190	.168	5	1
	7	79.000	alive	.	.	5	0

LN involved **Means and Medians for Survival Time** .047

LN. involved	Mean				Median			
	Estimate	Std. Error	95% Confidence Interval Lower Bound	95% Confidence Interval Upper Bound	Estimate	Std. Error	95% Confidence Interval Lower Bound	95% Confidence Interval Upper Bound
0	121.143	16.533	88.739	153.547	.	.	.	.
1	31.294	5010	21.474	41.114	25.000	2706	19.697	30.303
2	40.619	10.503	20.032	61.206	30.000	6279	17.693	42.307
Overall	52.865	9885	33.490	72.240	29.000	6795	15.681	42.319

Overall Comparisons

	Chi-Square	df	Sig.
Log Rank (Mantel-Cox)	6111	2	.047

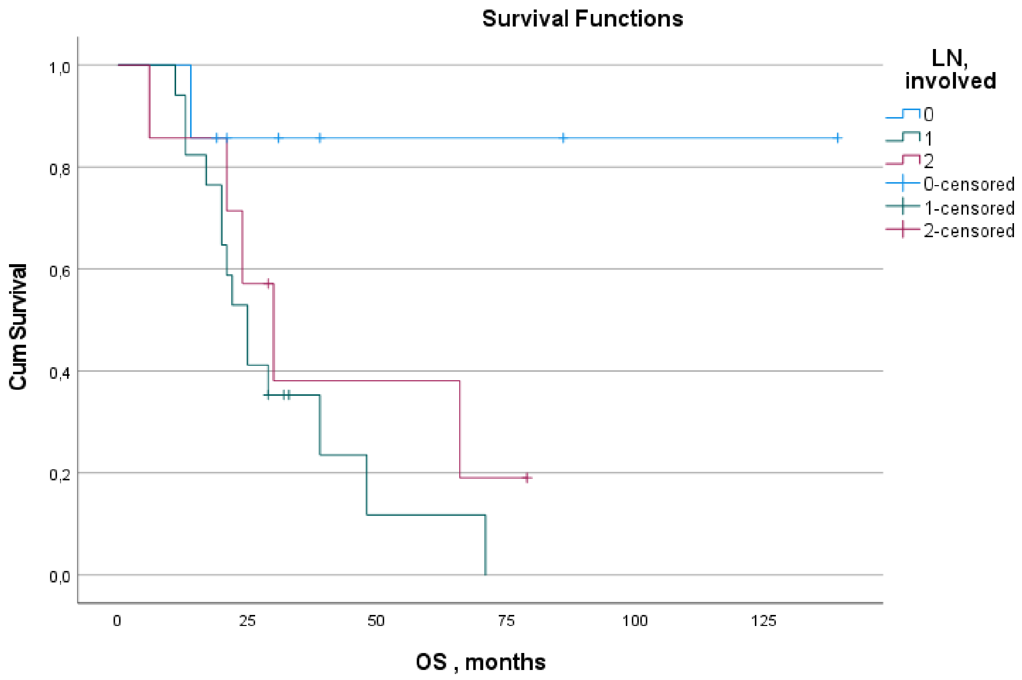

Frequencies

LN. involved

		Frequency	Per cent	Valid Percent	Cumulative Percent
Valid	No	7	22.6	22.6	22.6
	Yes	24	77.4	77.4	100.0
	Total	31	100.0	100.0	

Kaplan-Meier

Case Processing Summary

LN. involved	Total N	N of Events	Censored N	Censored Per cent
No	7	1	6	85.7%
Yes	24	19	5	20.8%
Overall	31	20	11	35.5%

Survival Table

LN. involved		Time	Status	Cumulative Proportion Surviving at the Time Estimate	Cumulative Proportion Surviving at the Time Std. Error	N of Cumulative Events	N of Remaining Cases
No	1	14.000	dead	.857	.132	1	6
	2	19.000	alive	.	.	1	5
	3	21.000	alive	.	.	1	4
	4	31.000	alive	.	.	1	3
	5	39.000	alive	.	.	1	2
	6	86.000	alive	.	.	1	1

	7	139.000	alive	.	.	1	0
Yes	1	6000	dead	.958	.041	1	23
	2	11.000	dead	.917	.056	2	22
	3	13.000	dead	.	.	3	21
	4	13.000	dead	.833	.076	4	20
	5	17.000	dead	.792	.083	5	19
	6	20.000	dead	.	.	6	18
	7	20.000	dead	.708	.093	7	17
	8	21.000	dead	.	.	8	16
	9	21.000	dead	.625	.099	9	15
	10	22.000	dead	.583	.101	10	14
	11	24.000	dead	.542	.102	11	13
	12	25.000	dead	.	.	12	12
	13	25.000	dead	.458	.102	13	11
	14	29.000	dead	.417	.101	14	10
	15	29.000	alive	.	.	14	9
	16	29.000	alive	.	.	14	8
	17	30.000	dead	.365	.101	15	7
	18	32.000	alive	.	.	15	6
	19	33.000	alive	.	.	15	5
	20	39.000	dead	.292	.104	16	4
	21	48.000	dead	.219	.100	17	3
	22	66.000	dead	.146	.089	18	2
	23	71.000	dead	.073	.068	19	1
	24	79.000	alive	.	.	19	0

Means and Medians for Survival Time .023 **N- - не достигнута. N+ - 25 [95%CI:16.6;33.37]**

	Mean				Median			
			95% Confidence Interval				95% Confidence Interval	
LN. involved	Estimate	Std. Error	Lower Bound	Upper Bound	Estimate	Std. Error	Lower Bound	Upper Bound
No	121.143	16.533	88.739	153.547	.	.	.	.
Yes	34.781	4836	25.303	44.259	25.000	4272	16.627	33.373
Overall	52.865	9885	33.490	72.240	29.000	6795	15.681	42.319

Overall Comparisons

	Chi-Square	df	Sig.
Log Rank (Mantel-Cox)	5174	1	.023

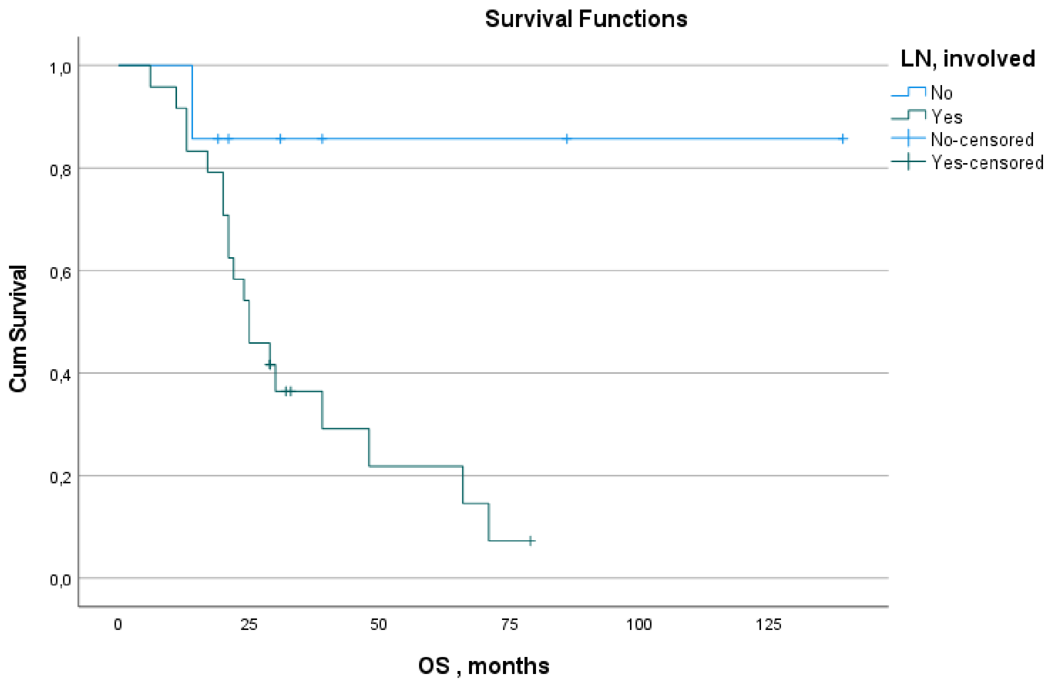

Kaplan-Meier

Case Processing Summary

CA 19-9 before surgery	Total N	N of Events	Censored	
			N	Per cent
<= 66	16	9	7	43.8%
67+	15	14	1	6.7%
Overall	31	23	8	25.8%

Survival Table

CA 19-9 before surgery		Time	Status	Cumulative Proportion Surviving at the Time		N of Cumulative Events	N of Remaining Cases
				Estimate	Std. Error		
<= 66	1	10.000	yes	.	.	1	15
	2	10.000	yes	.875	.083	2	14
	3	11.000	yes	.813	.098	3	13
	4	14.000	yes	.750	.108	4	12
	5	14.000	no	.	.	4	11
	6	17.000	no	.	.	4	10
	7	18.000	yes	.675	.121	5	9
	8	19.000	no	.	.	5	8
	9	20.000	yes	.	.	6	7
	10	20.000	yes	.	.	7	6
	11	20.000	yes	.422	.138	8	5
	12	21.000	no	.	.	8	4

13	29.000	no	.	.	8	3
14	39.000	no	.	.	8	2
15	66.000	yes	.211	.164	9	1
16	139.000	no	.	.	9	0

67+	1	4000	yes	.933	.064	1	14
	2	6000	yes	.867	.088	2	13
	3	9000	yes	.800	.103	3	12
	4	12.000	yes	.733	.114	4	11
	5	13.000	yes	.667	.122	5	10
	6	14.000	yes	.	.	6	9
	7	14.000	yes	.533	.129	7	8
	8	16.000	yes	.467	.129	8	7
	9	17.000	yes	.	.	9	6
	10	17.000	yes	.333	.122	10	5
	11	18.000	yes	.	.	11	4
	12	18.000	yes	.200	.103	12	3
	13	38.000	yes	.133	.088	13	2
	14	52.000	yes	.067	.064	14	1
	15	86.000	no	.	.	14	0

Means and Medians for Survival Time .038

< 66 – 20 [95%CI:17.86;22.14] . >66 – 16 [95%:12.97;19.03]

CA 19-9 before surgery	Mean				Median			
			95% Confidence Interval				95% Confidence Interval	
	Estimate	Std. Error	Lower Bound	Upper Bound	Estimate	Std. Error	Lower Bound	Upper Bound
<= 66	52.467	16.105	20.902	84.033	20.000	1090	17.863	22.137
67+	22.267	5345	11.791	32.742	16.000	1546	12.970	19.030
Overall	37.879	8951	20.334	55.423	18.000	1036	15.970	20.030

Overall Comparisons

	Chi-Square	df	Sig.
Log Rank (Mantel-Cox)	4316	1	.038

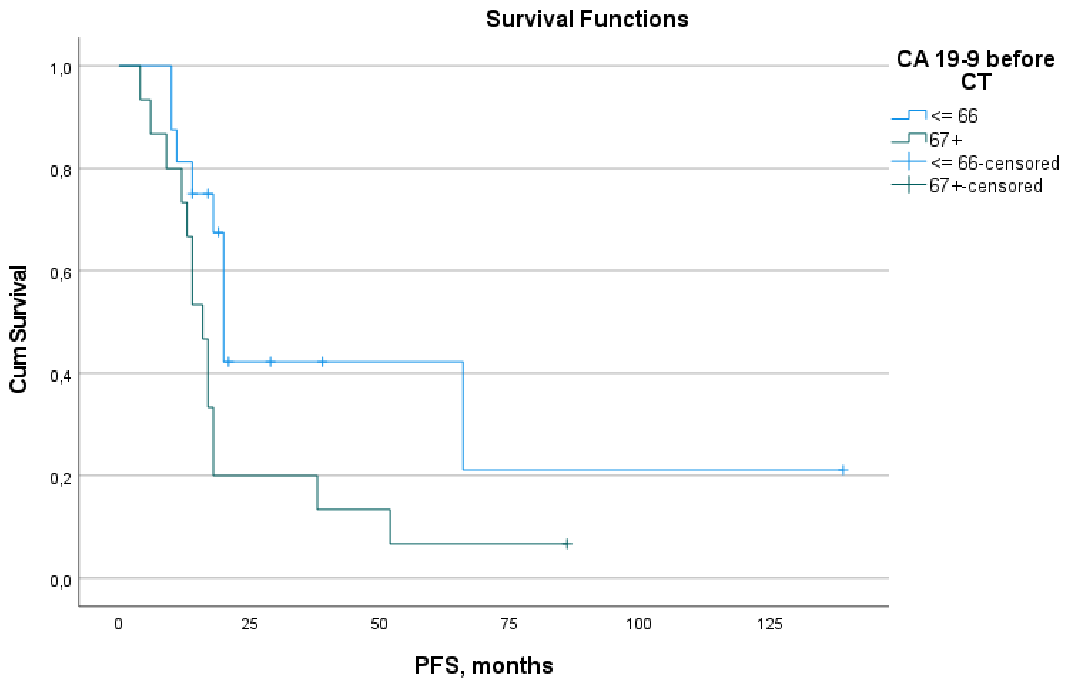

Kaplan-Meier

Case Processing Summary

Tumour size. Pathol. mm	Total N	N of Events	Censored	
			N	Per cent
<= 43	16	12	4	25.0%
44+	15	11	4	26.7%
Overall	31	23	8	25.8%

Survival Table

Tumour size. Pathol. mm		Time	Status	Cumulative Proportion Surviving at the Time		N of Cumulative Events	N of Remaining Cases
				Estimate	Std. Error		
<= 43	1	4000	yes	.938	.061	1	15
	2	10.000	yes	.	.	2	14
	3	10.000	yes	.813	.098	3	13
	4	11.000	yes	.750	.108	4	12
	5	14.000	yes	.	.	5	11
	6	14.000	yes	.625	.121	6	10
	7	14.000	no	.	.	6	9
	8	16.000	yes	.556	.126	7	8
	9	18.000	yes	.	.	8	7
	10	18.000	yes	.417	.127	9	6
	11	20.000	yes	.	.	10	5
	12	20.000	yes	.	.	11	4

	13	20.000	yes	.208	.106	12	3
	14	21.000	no	.	.	12	2
	15	39.000	no	.	.	12	1
	16	139.000	no	.	.	12	0
44+	1	6000	yes	.933	.064	1	14
	2	9000	yes	.867	.088	2	13
	3	12.000	yes	.800	.103	3	12
	4	13.000	yes	.733	.114	4	11
	5	14.000	yes	.667	.122	5	10
	6	17.000	yes	.	.	6	9
	7	17.000	yes	.533	.129	7	8
	8	17.000	no	.	.	7	7
	9	18.000	yes	.457	.131	8	6
	10	19.000	no	.	.	8	5
	11	29.000	no	.	.	8	4
	12	38.000	yes	.343	.139	9	3
	13	52.000	yes	.229	.132	10	2
	14	66.000	yes	.114	.104	11	1
	15	86.000	no	.	.	11	0

Tumor size. pathol. mm **Means and Medians for Survival Time** .715 **<43 – 18 [95%CI:14.41;21.59] . >43 – 18 [95%CI:0.00;37.03]**

Tumour size. Pathol. mm	Mean				Median			
			95% Confidence Interval				95% Confidence Interval	
	Estimate	Std. Error	Lower Bound	Upper Bound	Estimate	Std. Error	Lower Bound	Upper Bound
<= 43	40.674	13.196	14.810	66.538	18.000	1830	14.413	21.587
44+	34.895	7412	20.368	49.423	18.000	9711	.000	37.033
Overall	37.879	8951	20.334	55.423	18.000	1036	15.970	20.030

Overall Comparisons

	Chi-Square	df	Sig.
Log Rank (Mantel-Cox)	.133	1	.715

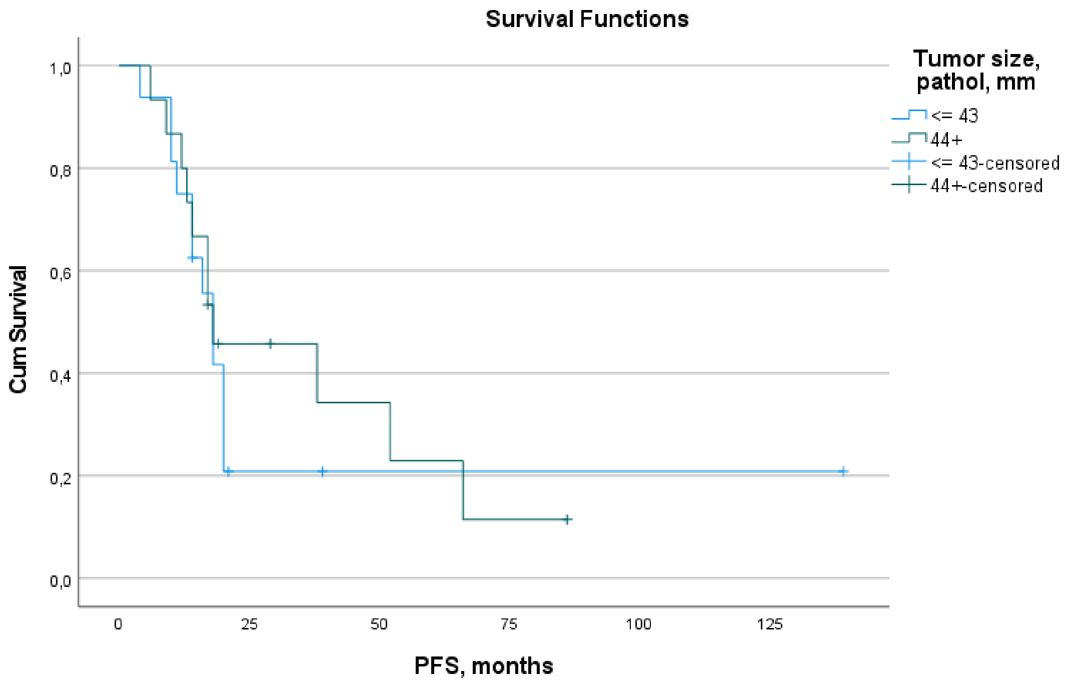

Kaplan-Meier

Case Processing Summary

Dindo-C	Total N	N of Events	Censored N	Censored Per cent
0	22	17	5	22.7%
1	4	2	2	50.0%
2	5	4	1	20.0%
Overall	31	23	8	25.8%

Survival Table

Dindo-C		Time	Status	Cumulative Proportion Surviving at the Time Estimate	Cumulative Proportion Surviving at the Time Std. Error	N of Cumulative Events	N of Remaining Cases
0	1	6000	yes	.955	.044	1	21
	2	9000	yes	.909	.061	2	20
	3	10.000	yes	.	.	3	19
	4	10.000	yes	.818	.082	4	18
	5	12.000	yes	.773	.089	5	17
	6	13.000	yes	.727	.095	6	16
	7	14.000	yes	.682	.099	7	15
	8	17.000	yes	.	.	8	14
	9	17.000	yes	.591	.105	9	13
	10	18.000	yes	.	.	10	12
	11	18.000	yes	.	.	11	11
	12	18.000	yes	.455	.106	12	10

	13	19.000	no	.	.	12	9
	14	20.000	yes	.	.	13	8
	15	20.000	yes	.	.	14	7
	16	20.000	yes	.303	.101	15	6
	17	21.000	no	.	.	15	5
	18	29.000	no	.	.	15	4
	19	39.000	no	.	.	15	3
	20	52.000	yes	.202	.106	16	2
	21	66.000	yes	.101	.089	17	1
	22	139.000	no	.	.	17	0
1	1	11.000	yes	.750	.217	1	3
	2	14.000	yes	.500	.250	2	2
	3	17.000	no	.	.	2	1
	4	86.000	no	.	.	2	0
2	1	4000	yes	.800	.179	1	4
	2	14.000	yes	.600	.219	2	3
	3	14.000	no	.	.	2	2
	4	16.000	yes	.300	.239	3	1
	5	38.000	yes	.000	.000	4	0

Means and Medians for Survival Time .535

	Mean				Median			
			95% Confidence Interval				95% Confidence Interval	
Dindo-C	Estimate	Std. Error	Lower Bound	Upper Bound	Estimate	Std. Error	Lower Bound	Upper Bound
0	36.354	9768	17.209	55.498	18.000	1106	15.832	20.168
1	49.250	18.383	13.220	85.280	14.000	.	.	.
2	19.800	7242	5606	33.994	16.000	1592	12.880	19.120
Overall	37.879	8951	20.334	55.423	18.000	1036	15.970	20.030

Overall Comparisons

	Chi-Square	df	Sig.
Log Rank (Mantel-Cox)	1251	2	.535

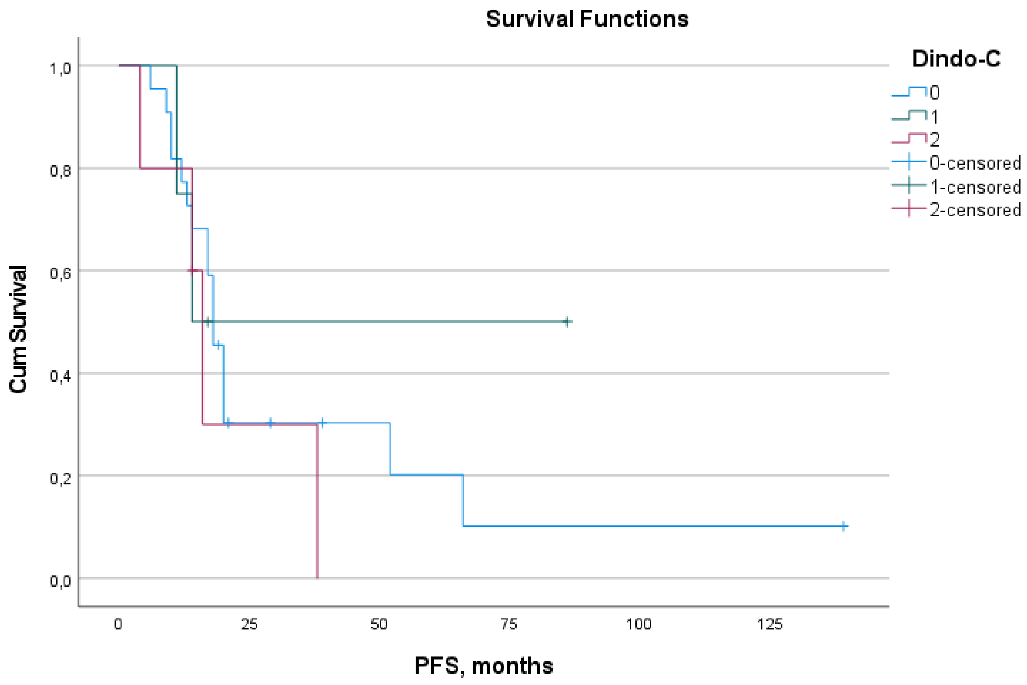

Kaplan-Meier

Case Processing Summary

LN. involved	Total N	N of Events	Censored N	Censored Per cent
No	7	1	6	85.7%
Yes	24	22	2	8.3%
Overall	31	23	8	25.8%

Survival Table

LN. involved		Time	Status	Cumulative Proportion Surviving at the Time Estimate	Cumulative Proportion Surviving at the Time Std. Error	N of Cumulative Events	N of Remaining Cases
No	1	11.000	yes	.857	.132	1	6
	2	14.000	no	.	.	1	5
	3	19.000	no	.	.	1	4
	4	21.000	no	.	.	1	3
	5	39.000	no	.	.	1	2
	6	86.000	no	.	.	1	1
	7	139.000	no	.	.	1	0
Yes	1	4000	yes	.958	.041	1	23
	2	6000	yes	.917	.056	2	22
	3	9000	yes	.875	.068	3	21
	4	10.000	yes	.	.	4	20
	5	10.000	yes	.792	.083	5	19
	6	12.000	yes	.750	.088	6	18

7	13.000	yes	.708	.093	7	17
8	14.000	yes	.	.	8	16
9	14.000	yes	.	.	9	15
10	14.000	yes	.583	.101	10	14
11	16.000	yes	.542	.102	11	13
12	17.000	yes	.	.	12	12
13	17.000	yes	.458	.102	13	11
14	17.000	no	.	.	13	10
15	18.000	yes	.	.	14	9
16	18.000	yes	.	.	15	8
17	18.000	yes	.321	.097	16	7
18	20.000	yes	.	.	17	6
19	20.000	yes	.	.	18	5
20	20.000	yes	.183	.082	19	4
21	29.000	no	.	.	19	3
22	38.000	yes	.122	.074	20	2
23	52.000	yes	.061	.057	21	1
24	66.000	yes	.000	.000	22	0

LN. involved **Means and Medians for Survival Time** .003

N- - не достигнута. N+ - 17 [95%CI:13.96;20.04]

	Mean				Median			
			95% Confidence Interval				95% Confidence Interval	
LN. involved	Estimate	Std. Error	Lower Bound	Upper Bound	Estimate	Std. Error	Lower Bound	Upper Bound
No	120.714	16.929	87.533	153.896	.	.	.	.
Yes	21.258	3475	14.447	28.070	17.000	1550	13.962	20.038
Overall	37.879	8951	20.334	55.423	18.000	1036	15.970	20.030

Overall Comparisons

	Chi-Square	df	Sig.
Log Rank (Mantel-Cox)	9051	1	.003

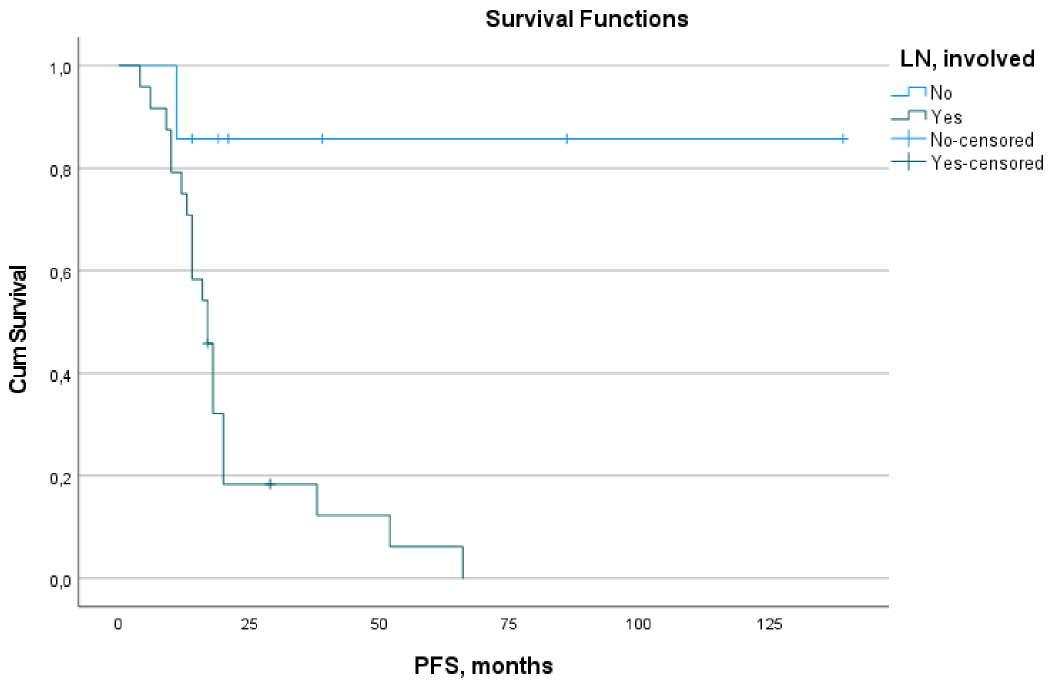

Survival Functions
LN, involved
No
Yes
No-censored
Yes-censored
Cum Survival
1,0
0,8
0,6
0,4
0,2
0,0
0
25
50
75
100
125
PFS, months
