# Supplementary material for: Hemodynamic, Surgical and Oncological Outcomes of 40 Distal Pancreatectomies with Celiac and Left Gastric Arteries Resection (DP CAR) without Arterial Reconstructions and Preoperative Embolization
Source: Cancers (Basel). 2022 Feb 28;14(5):1254. doi: 10.3390/cancers14051254 (PMC8909059; doi:10.3390/cancers14051254)
Supplement: Supplementary file 1 [file cancers-14-01254-s001.zip › cancers-1582412-supplementary/Supplement/File S3. Statistical analysis of hemodynamic changes after DPCAR .pdf]

**File S3.** Statistical analysis of hemodynamic changes in pancreatoduodenal arterial system after DP CAR.

Frequencies

**Patient #**

| | | Frequency | Percent | Valid Percent | Cumulative Percent |
|---|---|---|---|---|---|
| Valid | 1 | 1 | 2.6 | 2.6 | 2.6 |
| | 2 | 1 | 2.6 | 2.6 | 5.3 |
| | 3 | 1 | 2.6 | 2.6 | 7.9 |
| | 4 | 1 | 2.6 | 2.6 | 10.5 |
| | 5 | 1 | 2.6 | 2.6 | 13.2 |
| | 7 | 1 | 2.6 | 2.6 | 15.8 |
| | 8 | 1 | 2.6 | 2.6 | 18.4 |
| | 9 | 1 | 2.6 | 2.6 | 21.1 |
| | 10 | 1 | 2.6 | 2.6 | 23.7 |
| | 12 | 1 | 2.6 | 2.6 | 26.3 |
| | 13 | 1 | 2.6 | 2.6 | 28.9 |
| | 14 | 1 | 2.6 | 2.6 | 31.6 |
| | 15 | 1 | 2.6 | 2.6 | 34.2 |
| | 16 | 1 | 2.6 | 2.6 | 36.8 |
| | 17 | 1 | 2.6 | 2.6 | 39.5 |
| | 18 | 1 | 2.6 | 2.6 | 42.1 |
| | 19 | 1 | 2.6 | 2.6 | 44.7 |
| | 20 | 1 | 2.6 | 2.6 | 47.4 |
| | 21 | 1 | 2.6 | 2.6 | 50.0 |
| | 22 | 1 | 2.6 | 2.6 | 52.6 |
| | 23 | 1 | 2.6 | 2.6 | 55.3 |
| | 24 | 1 | 2.6 | 2.6 | 57.9 |
| | 25 | 1 | 2.6 | 2.6 | 60.5 |
| | 26 | 1 | 2.6 | 2.6 | 63.2 |
| | 27 | 1 | 2.6 | 2.6 | 65.8 |
| | 28 | 1 | 2.6 | 2.6 | 68.4 |
| | 29 | 1 | 2.6 | 2.6 | 71.1 |
| | 30 | 1 | 2.6 | 2.6 | 73.7 |
| | 31 | 1 | 2.6 | 2.6 | 76.3 |
| | 32 | 1 | 2.6 | 2.6 | 78.9 |
| | 33 | 1 | 2.6 | 2.6 | 81.6 |
| | 34 | 1 | 2.6 | 2.6 | 84.2 |
| | 35 | 1 | 2.6 | 2.6 | 86.8 |
| | 36 | 1 | 2.6 | 2.6 | 89.5 |

| | | | | | |
|---|---|---|---|---|---|
| | 37 | 1 | 2.6 | 2.6 | 92.1 |
| | 38 | 1 | 2.6 | 2.6 | 94.7 |
| | 39 | 1 | 2.6 | 2.6 | 97.4 |
| | 40 | 1 | 2.6 | 2.6 | 100.0 |
| | Total | 38 | 100.0 | 100.0 | |

Frequencies

**Type**

| | | Frequency | Percent | Valid Percent | Cumulative Percent |
|---|---|---|---|---|---|
| Valid | classical | 30 | 78.9 | 78.9 | 78.9 |
| | aberrant | 8 | 21.1 | 21.1 | 100.0 |
| | Total | 38 | 100.0 | 100.0 | |

**Puls preservation**

| | | Frequency | Percent | Valid Percent | Cumulative Percent |
|---|---|---|---|---|---|
| Valid | no | 4 | 10.5 | 10.5 | 10.5 |
| | yes | 34 | 89.5 | 89.5 | 100.0 |
| | Total | 38 | 100.0 | 100.0 | |

**Type od parenchimal blood flow**

| | | Frequency | Percent | Valid Percent | Cumulative Percent |
|---|---|---|---|---|---|
| Valid | collateral | 12 | 31.6 | 31.6 | 31.6 |
| | main | 26 | 68.4 | 68.4 | 100.0 |
| | Total | 38 | 100.0 | 100.0 | |

Frequencies

**Statistics**

| | | Linear blood flow velosity decrease.% | GDA diameter before surgery. mm | PDA diameter before surgery. mm | PHA diameter before surgery. mm | RGEA diameter before surgery. mm | Preop CHA/GDA diameters ratio | Preop CHA/PDA diameters ratio | GDA blood flow intencity alteration after/before | PDA blood flow intencity alteration after/before | PHA blood flow intencity alteration after/before | RGEA blood flow intencity alteration after/before | GDA diameters ratio after/before | PDA diameters ratio after/before | PHA diameters ratio after/before | RGEA diameters ratio after/before | GDA diameters difference after/before | PDA diameters difference after/before | PHA diameters difference after/before | RGEA diameters difference after/before |
|---|---|---|---|---|---|---|---|---|---|---|---|---|---|---|---|---|---|---|---|---|
| N | Valid | 38 | 38 | 38 | 38 | 38 | 38 | 38 | 38 | 38 | 38 | 35 | 38 | 38 | 38 | 35 | 38 | 38 | 38 | 35 |

| | | | | | | | | | | | | | | | | | | | | |
|---|---|---|---|---|---|---|---|---|---|---|---|---|---|---|---|---|---|---|---|---|
| | Missing | 0 | 0 | 0 | 0 | 0 | 0 | 0 | 0 | 0 | 0 | 3 | 0 | 0 | 0 | 3 | 0 | 0 | 0 | 3 |
| Mean | | -21.26 | 3.7255 | 2.5095 | 3.8621 | 2.5266 | 1.4134 | 2.1099 | 2.3731 | 7.3252 | 1.1849 | 3.5514 | 1.1086 | 1.4655 | 1.0002 | 1.2689 | .2324 | .9795 | -.0142 | .5520 |
| Std. Deviation | | 19.339 | 1.25049 | .82945 | .98164 | .77758 | .53004 | .82121 | 4.07544 | 14.52274 | .77377 | 4.00575 | .30400 | .38348 | .13250 | .29292 | .93073 | .56176 | .49601 | .54246 |
| Minimum | | -66 | 1.82 | 1.10 | 2.00 | 1.20 | .80 | .83 | .16 | .91 | .27 | .40 | .63 | .98 | .66 | .80 | -2.30 | -.07 | -1.61 | -.74 |
| Maximum | | 0 | 6.67 | 5.76 | 6.30 | 3.91 | 3.08 | 4.45 | 25.01 | 90.20 | 5.13 | 18.84 | 2.24 | 3.08 | 1.38 | 2.08 | 2.25 | 2.29 | 1.30 | 1.82 |
| Percentiles | 25 | -32.50 | 2.7000 | 1.9475 | 2.9000 | 1.9250 | 1.0691 | 1.3768 | .7839 | 2.1703 | .8394 | 1.2439 | .9409 | 1.2137 | .9359 | 1.0561 | -.2625 | .5800 | -.2500 | .1600 |
| | 50 | -16.00 | 3.6500 | 2.3050 | 4.2000 | 2.5500 | 1.2609 | 1.9696 | 1.4808 | 4.1565 | 1.1105 | 2.3497 | 1.1023 | 1.4278 | .9741 | 1.2381 | .3450 | .9500 | -.1100 | .5000 |
| | 75 | -7.00 | 4.4225 | 2.9100 | 4.4625 | 3.1925 | 1.7870 | 2.6785 | 2.4414 | 6.6945 | 1.3036 | 3.5022 | 1.2500 | 1.6083 | 1.0447 | 1.3680 | .8125 | 1.4425 | .1500 | .9100 |

Histogram

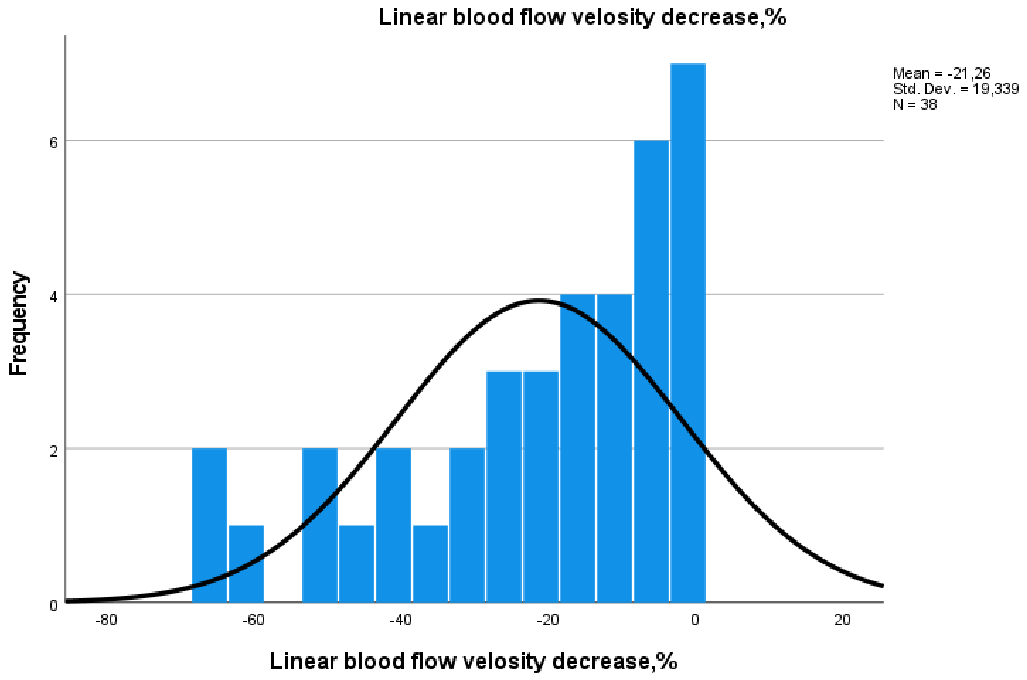

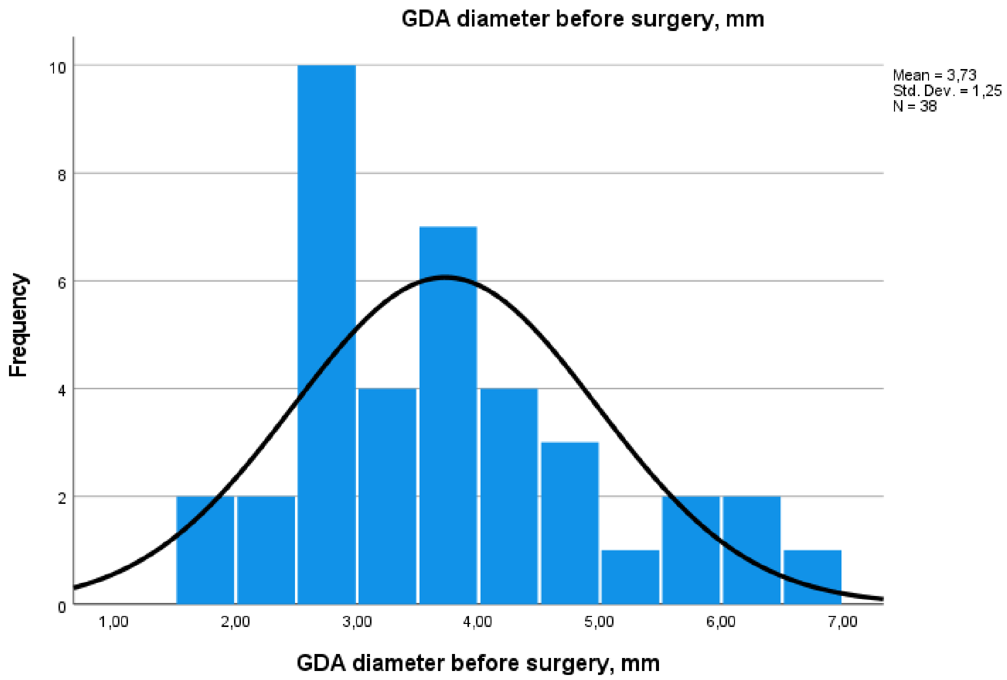

GDA diameter before surgery, mm
Mean = 3,73
Std. Dev. = 1,25
N = 38
Frequency
0
2
4
6
8
10
1,00
2,00
3,00
4,00
5,00
6,00
7,00
GDA diameter before surgery, mm


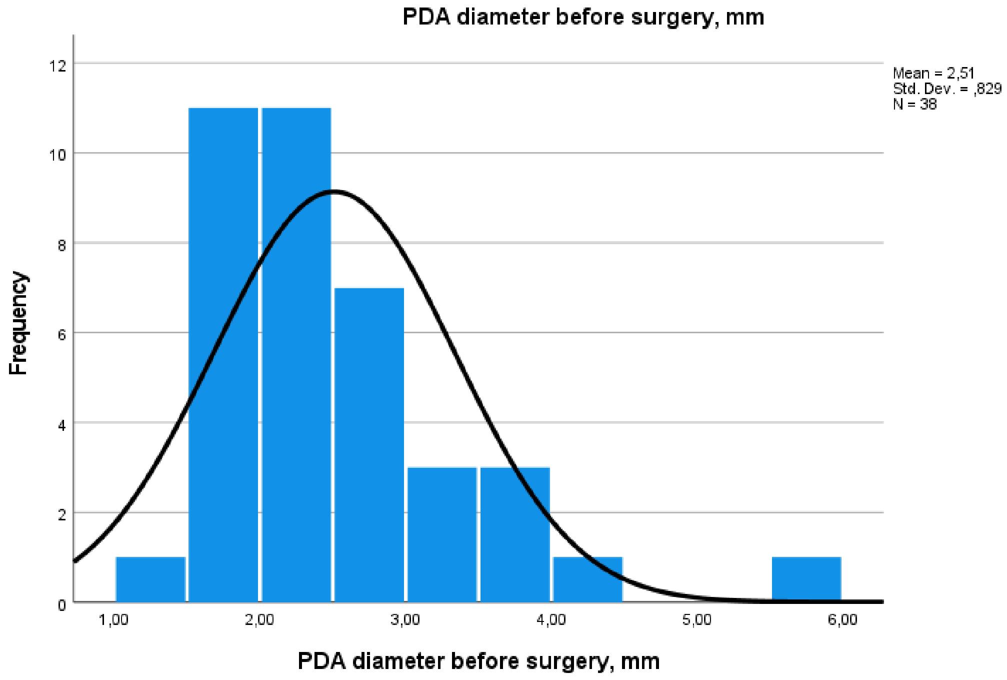

PDA diameter before surgery, mm
Mean = 2,51
Std. Dev. = ,829
N = 38
Frequency
0
2
4
6
8
10
12
1,00
2,00
3,00
4,00
5,00
6,00
PDA diameter before surgery, mm

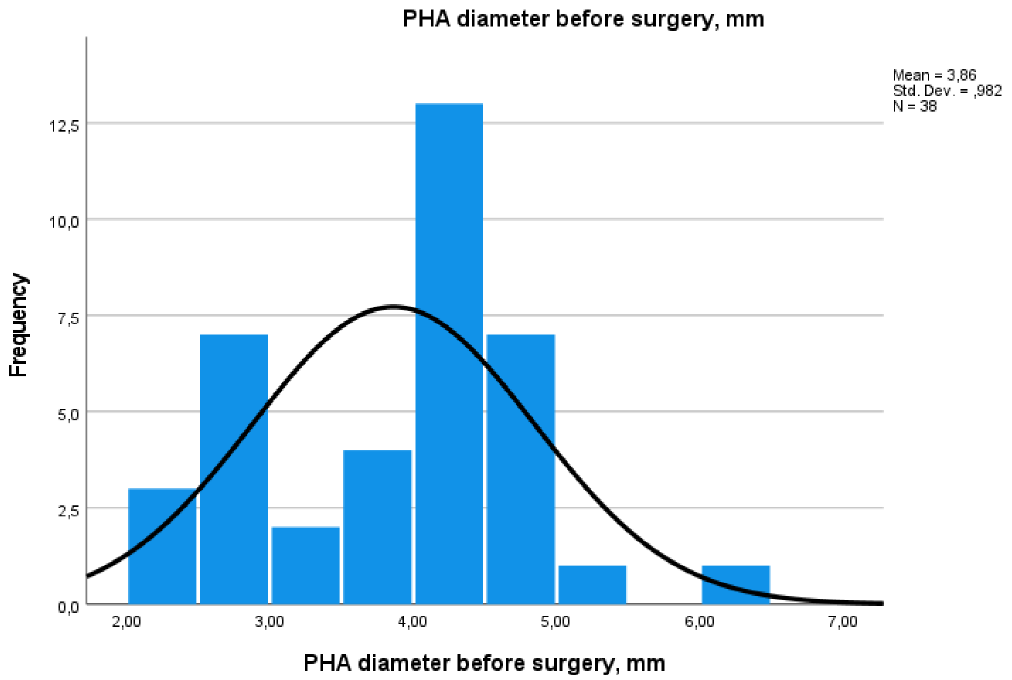
PHA diameter before surgery, mm
Mean = 3,86
Std. Dev. = ,982
N = 38
Frequency
12,5
10,0
7,5
5,0
2,5
0,0
2,00
3,00
4,00
5,00
6,00
7,00
PHA diameter before surgery, mm

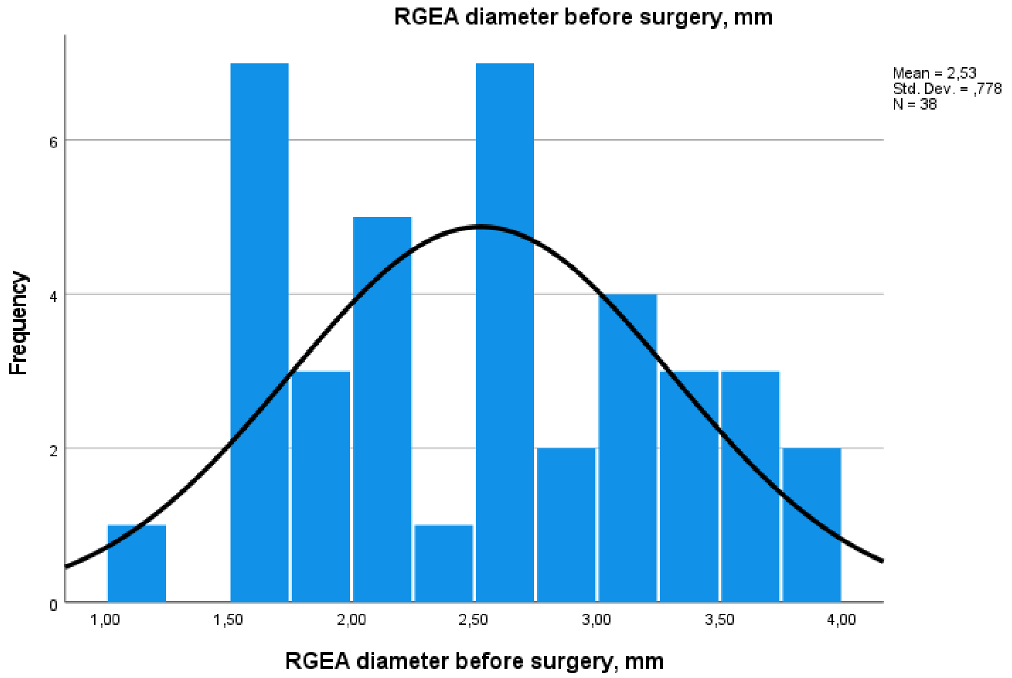
RGEA diameter before surgery, mm
Mean = 2,53
Std. Dev. = ,778
N = 38
Frequency
6
4
2
0
1,00
1,50
2,00
2,50
3,00
3,50
4,00
RGEA diameter before surgery, mm

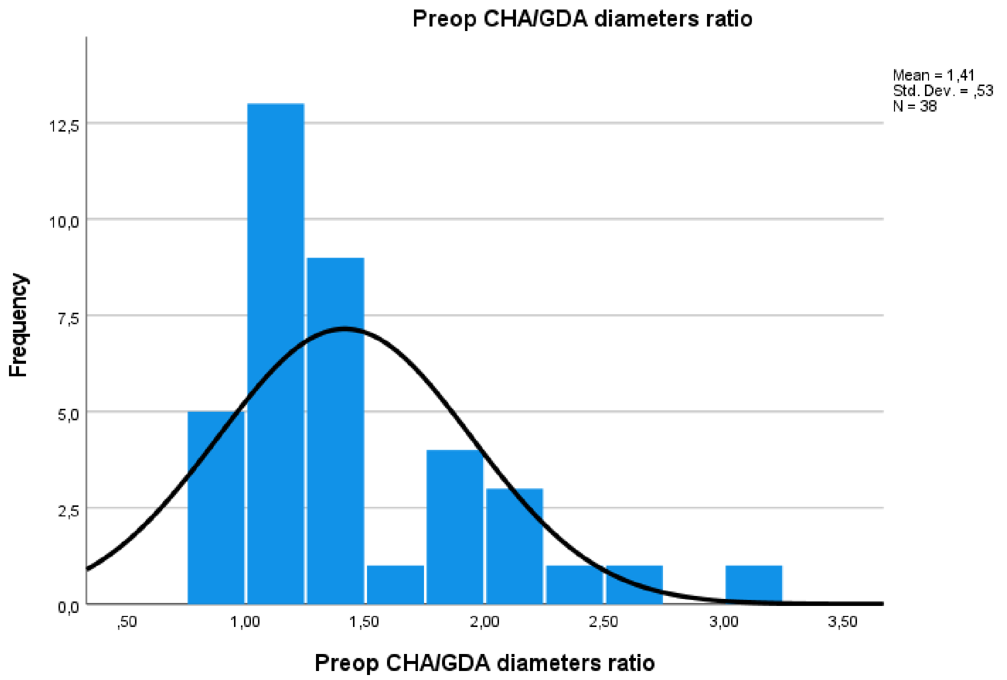
Preop CHA/GDA diameters ratio
Mean = 1,41
Std. Dev. = ,53
N = 38
Frequency
12,5
10,0
7,5
5,0
2,5
0,0
,50
1,00
1,50
2,00
2,50
3,00
3,50
Preop CHA/GDA diameters ratio

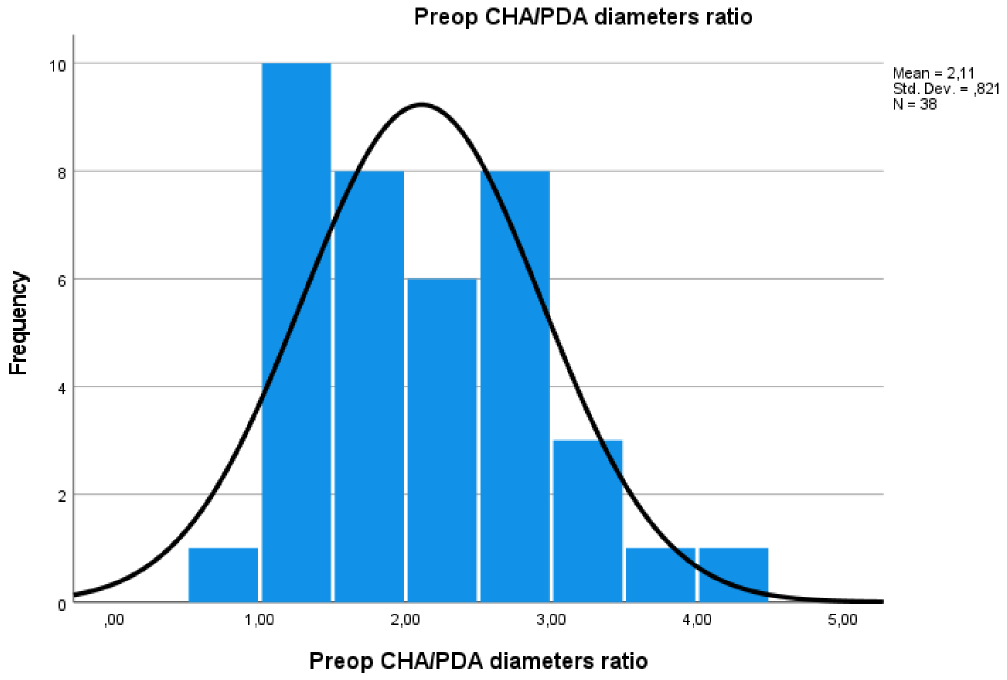
Preop CHA/PDA diameters ratio
Mean = 2,11
Std. Dev. = ,821
N = 38
Frequency
10
8
6
4
2
0
,00
1,00
2,00
3,00
4,00
5,00
Preop CHA/PDA diameters ratio

GDA blood flow intencity alteration after/before

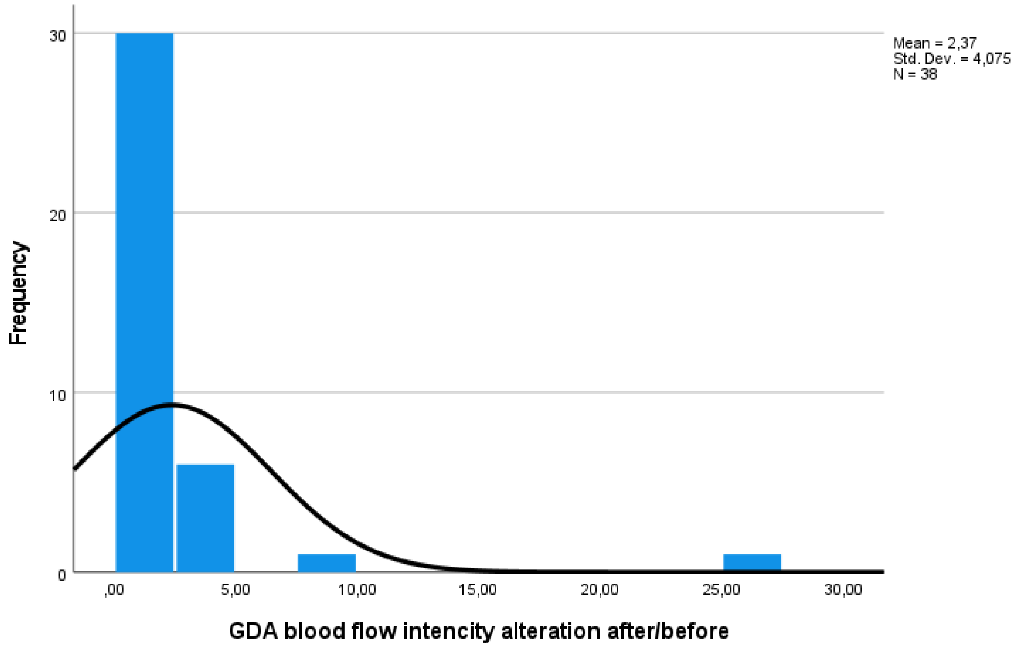


PDA blood flow intencity alteration after/before

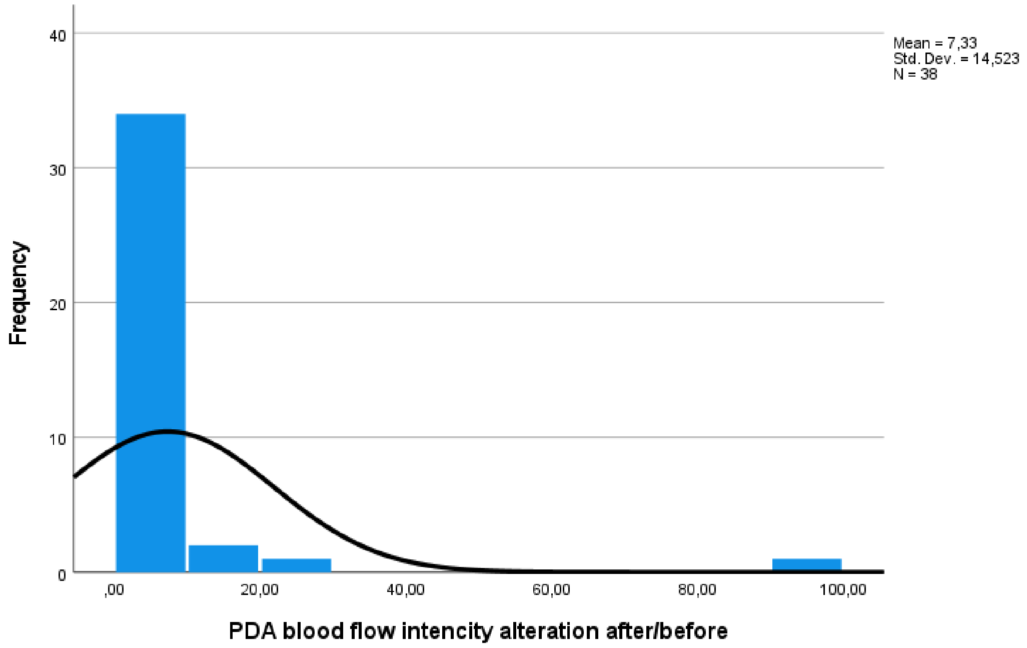

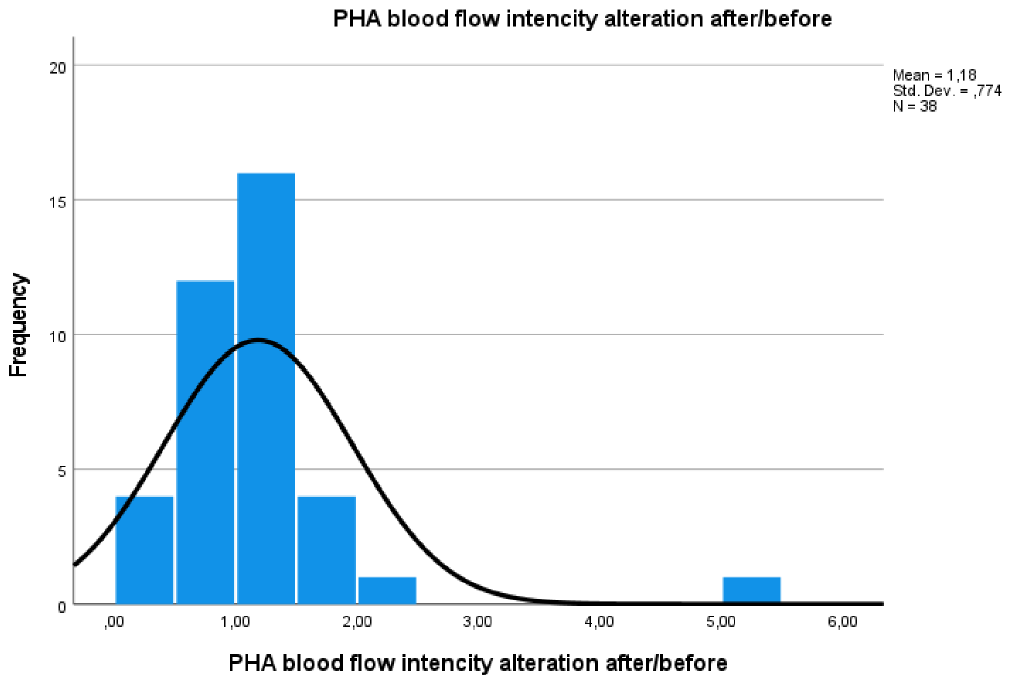
PHA blood flow intencity alteration after/before
Mean = 1,18
Std. Dev. = ,774
N = 38
Frequency
0
5
10
15
20
,00
1,00
2,00
3,00
4,00
5,00
6,00
PHA blood flow intencity alteration after/before

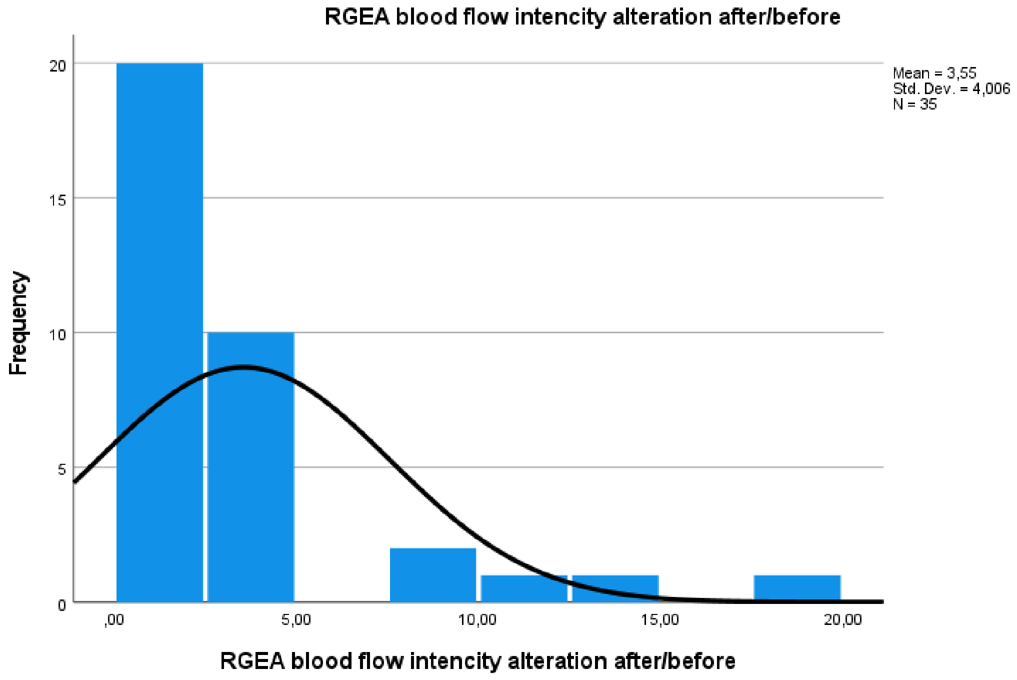
RGEA blood flow intencity alteration after/before
Mean = 3,55
Std. Dev. = 4,006
N = 35
Frequency
0
5
10
15
20
,00
5,00
10,00
15,00
20,00
RGEA blood flow intencity alteration after/before

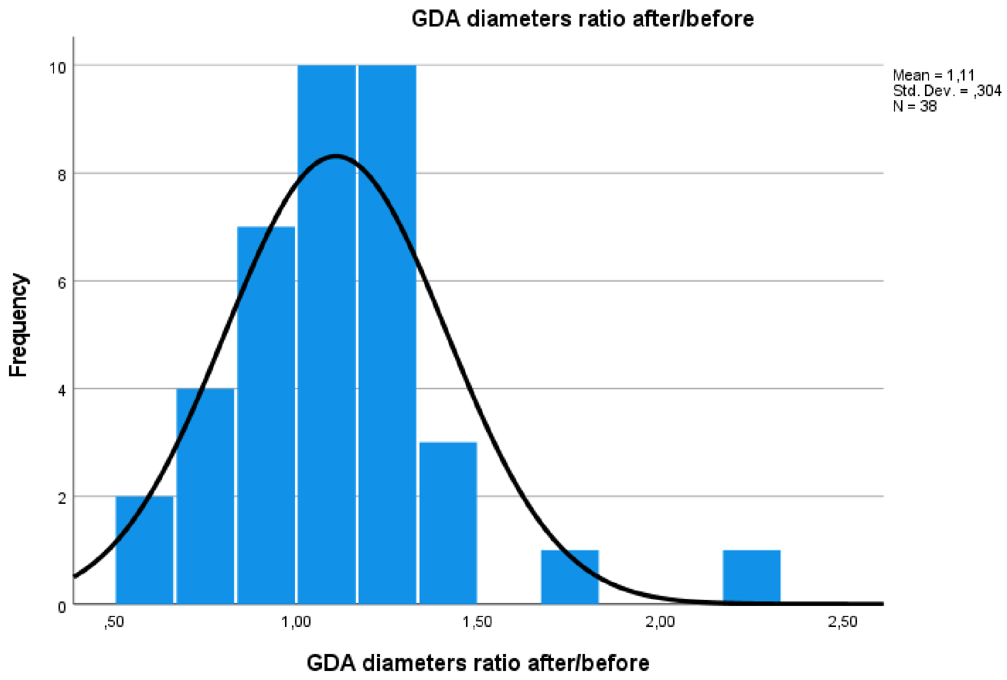
GDA diameters ratio after/before
Mean = 1,11
Std. Dev. = ,304
N = 38
Frequency
10
8
6
4
2
0
,50
1,00
1,50
2,00
2,50
GDA diameters ratio after/before

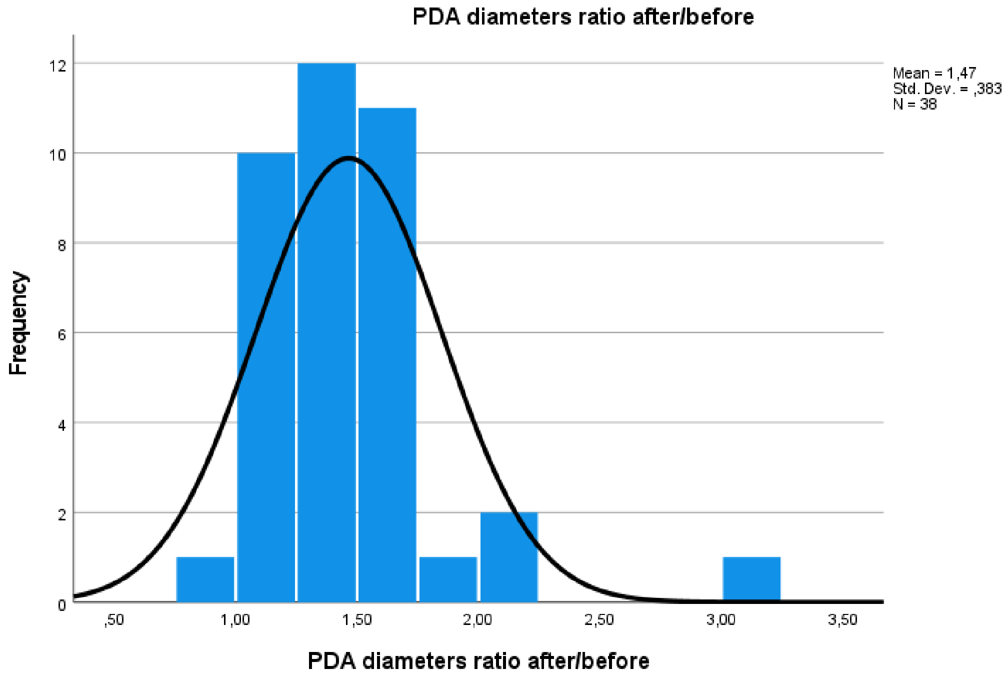
PDA diameters ratio after/before
Mean = 1,47
Std. Dev. = ,383
N = 38
Frequency
12
10
8
6
4
2
0
,50
1,00
1,50
2,00
2,50
3,00
3,50
PDA diameters ratio after/before

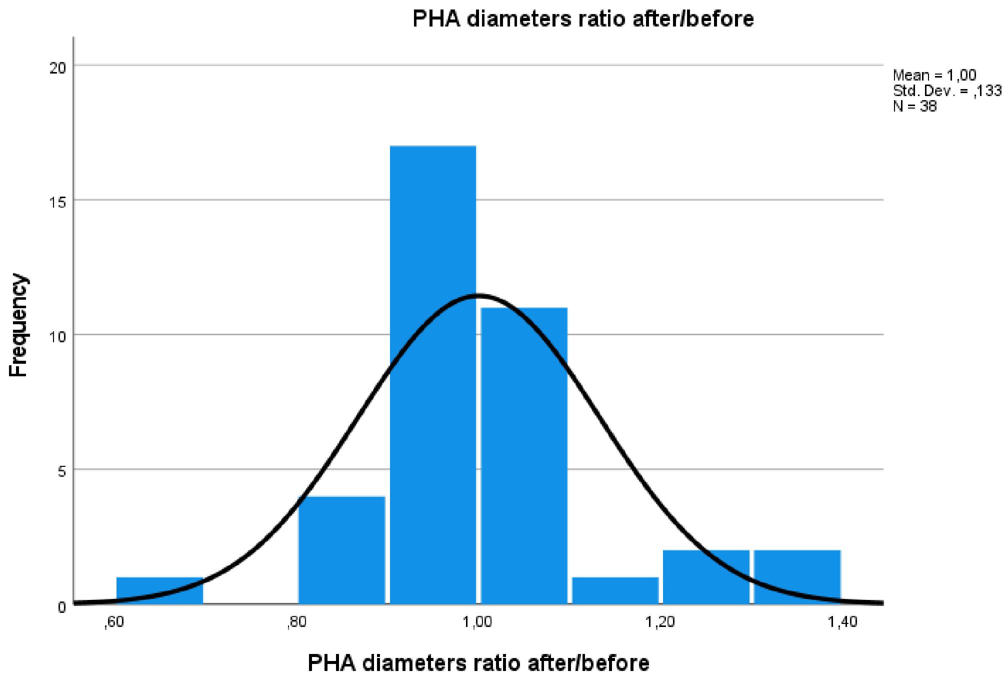
PHA diameters ratio after/before
Mean = 1,00
Std. Dev. = ,133
N = 38
Frequency
20
15
10
5
0
,60
,80
1,00
1,20
1,40
PHA diameters ratio after/before

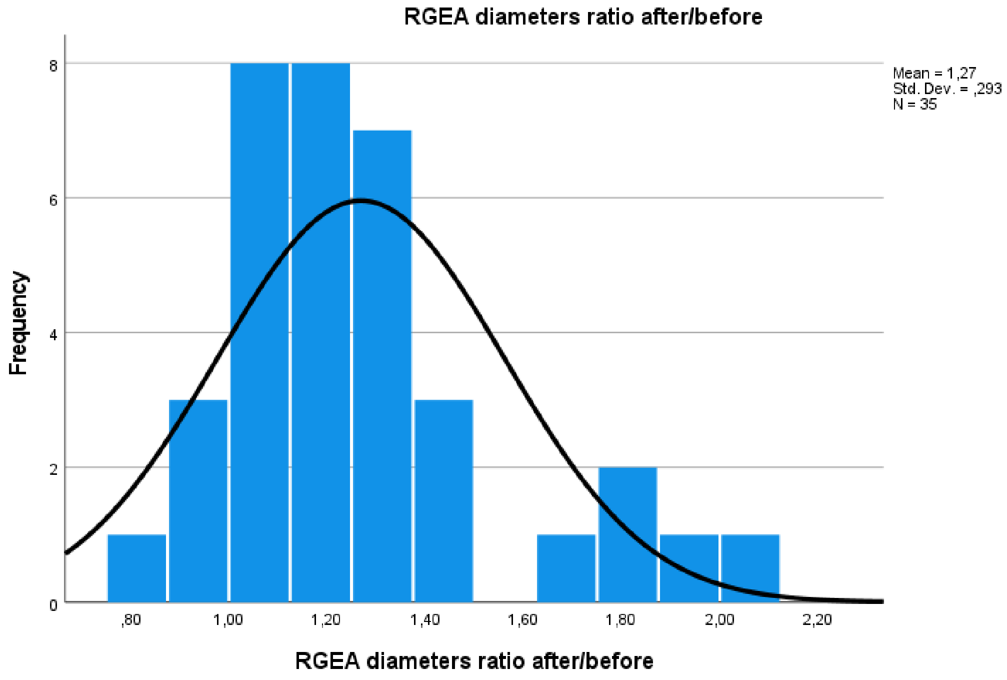
RGEA diameters ratio after/before
Mean = 1,27
Std. Dev. = ,293
N = 35
Frequency
8
6
4
2
0
,80
1,00
1,20
1,40
1,60
1,80
2,00
2,20
RGEA diameters ratio after/before

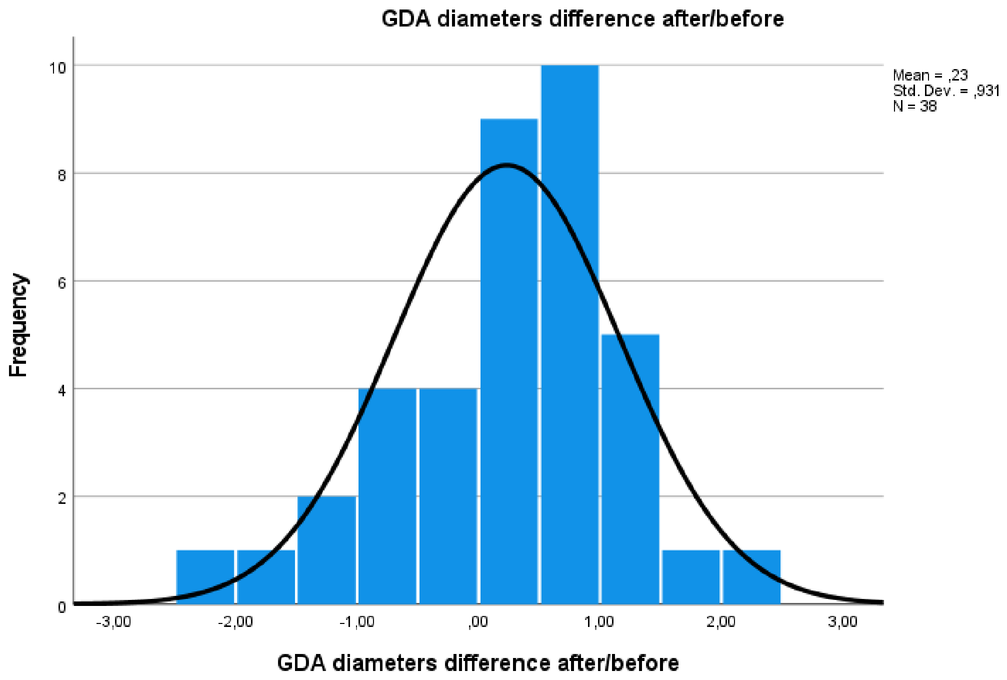
GDA diameters difference after/before
Mean = ,23
Std. Dev. = ,931
N = 38
Frequency
10
8
6
4
2
0
-3,00
-2,00
-1,00
,00
1,00
2,00
3,00
GDA diameters difference after/before

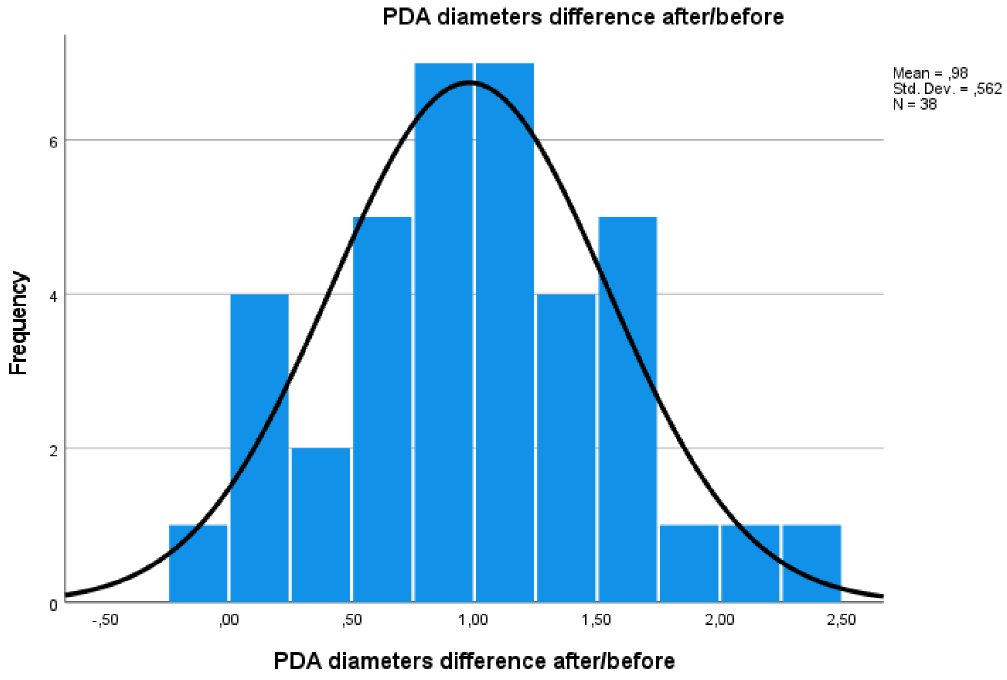
PDA diameters difference after/before
Mean = ,98
Std. Dev. = ,562
N = 38
Frequency
6
4
2
0
-,50
,00
,50
1,00
1,50
2,00
2,50
PDA diameters difference after/before

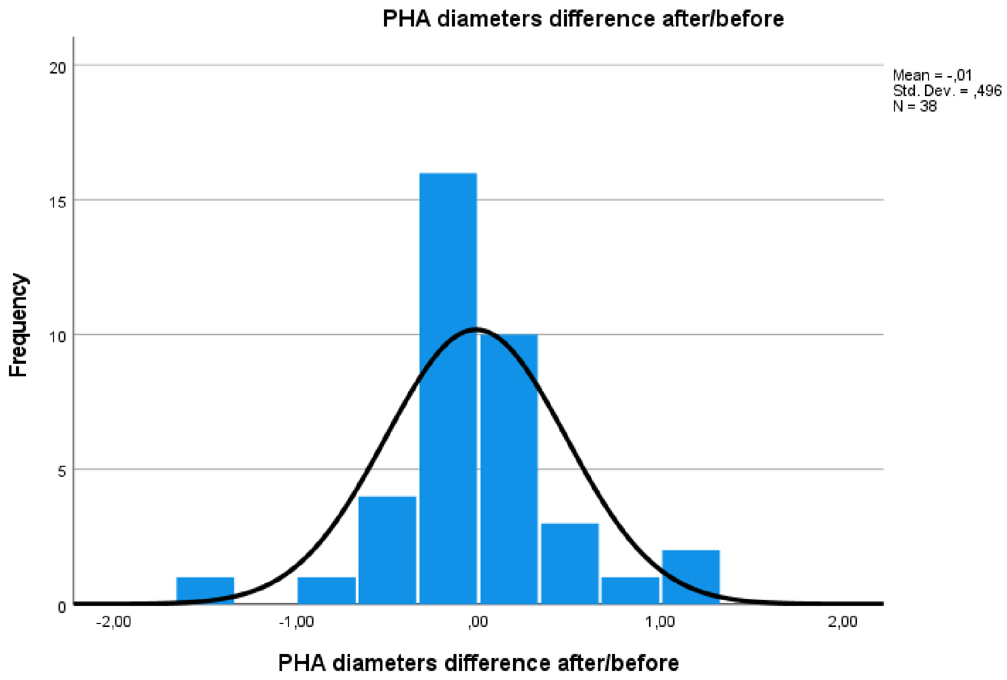

PHA diameters difference after/before
Mean = -,01
Std. Dev. = ,496
N = 38
Frequency
20
15
10
5
0
-2,00
-1,00
,00
1,00
2,00
PHA diameters difference after/before


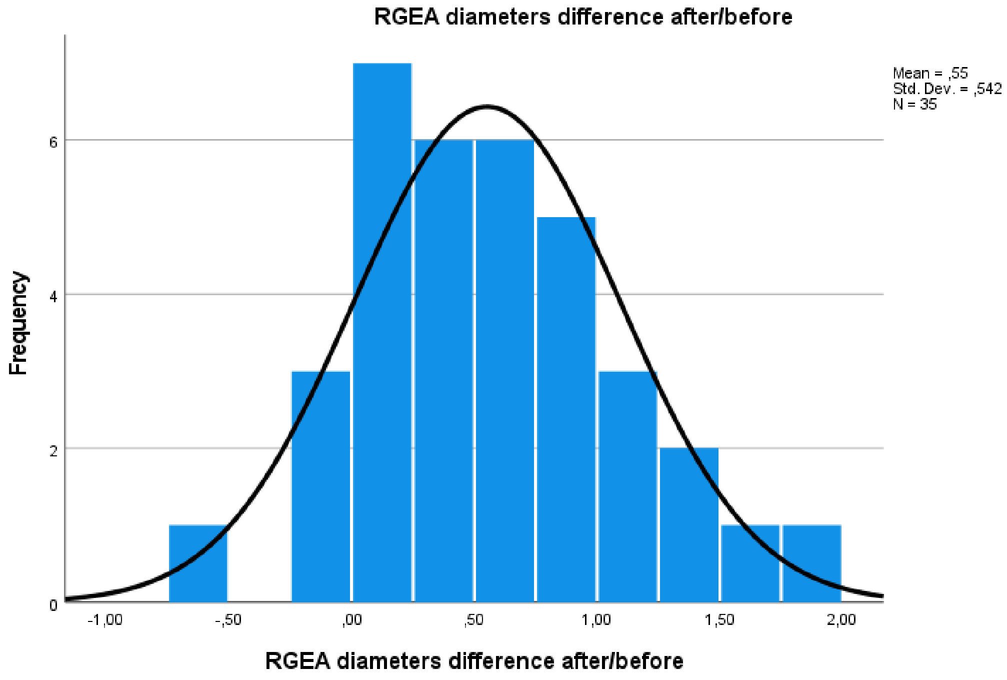

RGEA diameters difference after/before
Mean = ,55
Std. Dev. = ,542
N = 35
Frequency
6
4
2
0
-1,00
-,50
,00
,50
1,00
1,50
2,00
RGEA diameters difference after/before


T-Test

**Group Statistics**

| | Puls preservation | N | Mean | Std. Deviation | Std. Error Mean |
|---|---|---|---|---|---|
| Preop CHA/GDA diameters ratio | no | 4 | 1.2520 | .50953 | .25476 |
| | yes | 34 | 1.4324 | .53653 | .09201 |
| Preop CHA/PDA diameters ratio | no | 4 | 2.0719 | .96106 | .48053 |
| | yes | 34 | 2.1144 | .81974 | .14058 |

**Independent Samples Test**

| | | Levene's Test for Equality of Variances | | t-test for Equality of Means | | | | | | |
|---|---|---|---|---|---|---|---|---|---|---|
| | | | | | | | | | 95% Confidence Interval of the Difference | |
| | | F | Sig. | t | df | Sig. (2-tailed) | Mean Difference | Std. Error Difference | Lower | Upper |
| Preop CHA/GDA diameters ratio | Equal variances assumed | .056 | .814 | -.639 | 36 | .527 | -.18036 | .28244 | -.75318 | .39246 |
| | Equal variances not assumed | | | -.666 | 3.828 | .543 | -.18036 | .27087 | -.94595 | .58523 |
| Preop CHA/PDA diameters ratio | Equal variances assumed | .403 | .529 | -.097 | 36 | .924 | -.04253 | .44002 | -.93493 | .84987 |
| | Equal variances not assumed | | | -.085 | 3.533 | .937 | -.04253 | .50067 | -1.50814 | 1.42308 |

NPar Tests

Mann-Whitney Test

**Ranks**

| | Puls preservation | N | Mean Rank | Sum of Ranks |
|---|---|---|---|---|
| Preop CHA/GDA diameters ratio | no | 4 | 15.00 | 60.00 |
| | yes | 34 | 20.03 | 681.00 |
| | Total | 38 | | |
| Preop CHA/PDA diameters ratio | no | 4 | 18.75 | 75.00 |
| | yes | 34 | 19.59 | 666.00 |
| | Total | 38 | | |

**Test Statistics**

| | Preop CHA/GDA diameters ratio | Preop CHA/PDA diameters ratio |
|---|---|---|
| Mann-Whitney U | 50.000 | 65.000 |
| Wilcoxon W | 60.000 | 75.000 |
| Z | -.856 | -.143 |
| Asymp. Sig. (2-tailed) | .392 | .887 |
| Exact Sig. [2*(1-tailed Sig.)] | .420 | .909 |

Graph

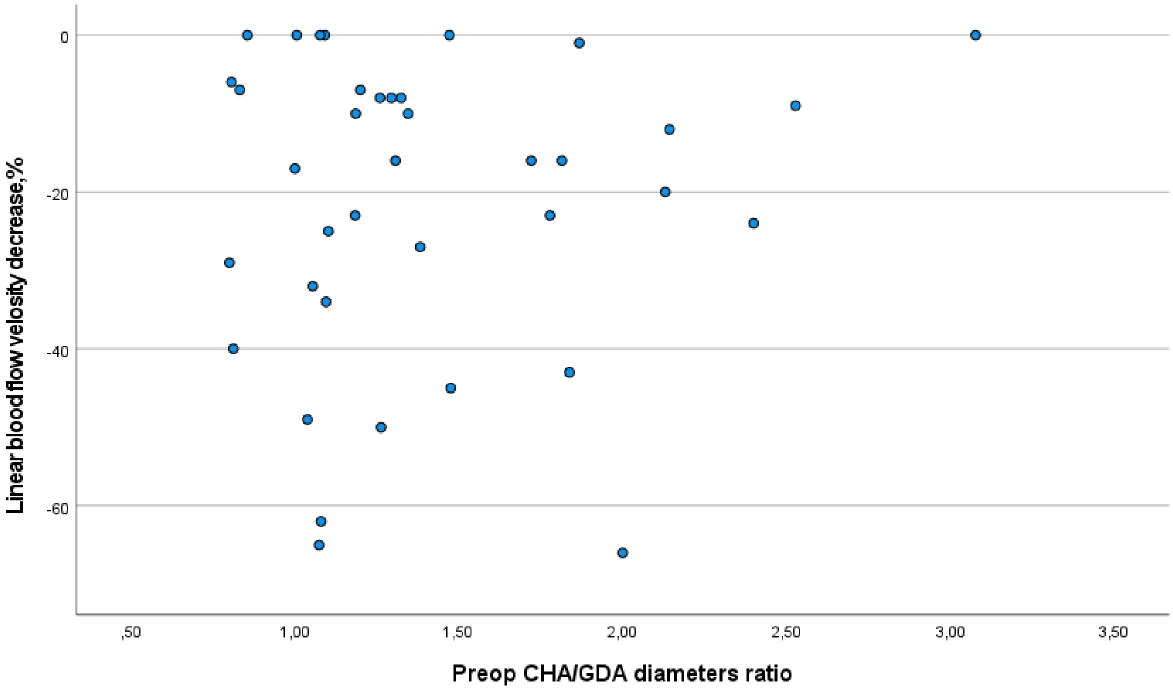


Graph

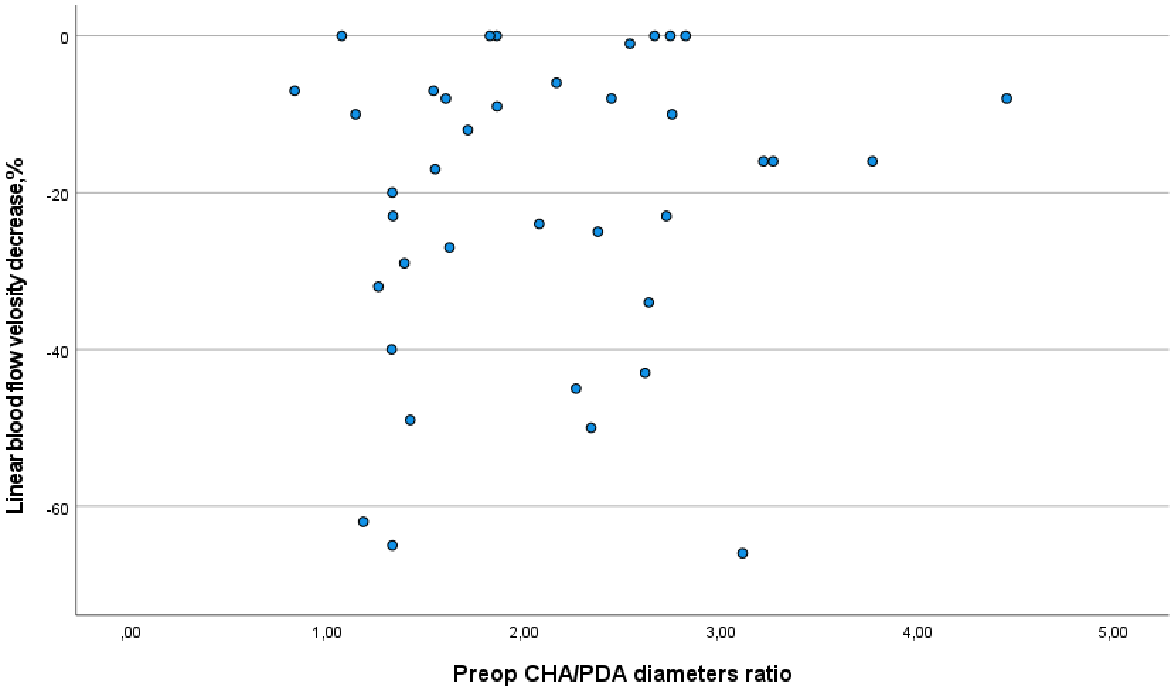


Correlations

**Correlations**

| | | Preop CHA/GDA diameters ratio | Preop CHA/PDA diameters ratio |
|---|---|---|---|
| Preop CHA/GDA diameters ratio | Pearson Correlation | 1 | .142 |
| | Sig. (2-tailed) | | .395 |
| | N | 38 | 38 |
| Preop CHA/PDA diameters ratio | Pearson Correlation | .142 | 1 |
| | Sig. (2-tailed) | .395 | |
| | N | 38 | 38 |

Nonparametric Correlations

**Correlations**

| | | | Preop CHA/GDA diameters ratio | Preop CHA/PDA diameters ratio |
|---|---|---|---|---|
| Spearman's rho | Preop CHA/GDA diameters ratio | Correlation Coefficient | 1.000 | .283 |
| | | Sig. (2-tailed) | . | .085 |
| | | N | 38 | 38 |
| | Preop CHA/PDA diameters ratio | Correlation Coefficient | .283 | 1.000 |
| | | Sig. (2-tailed) | .085 | . |
| | | N | 38 | 38 |

Graph

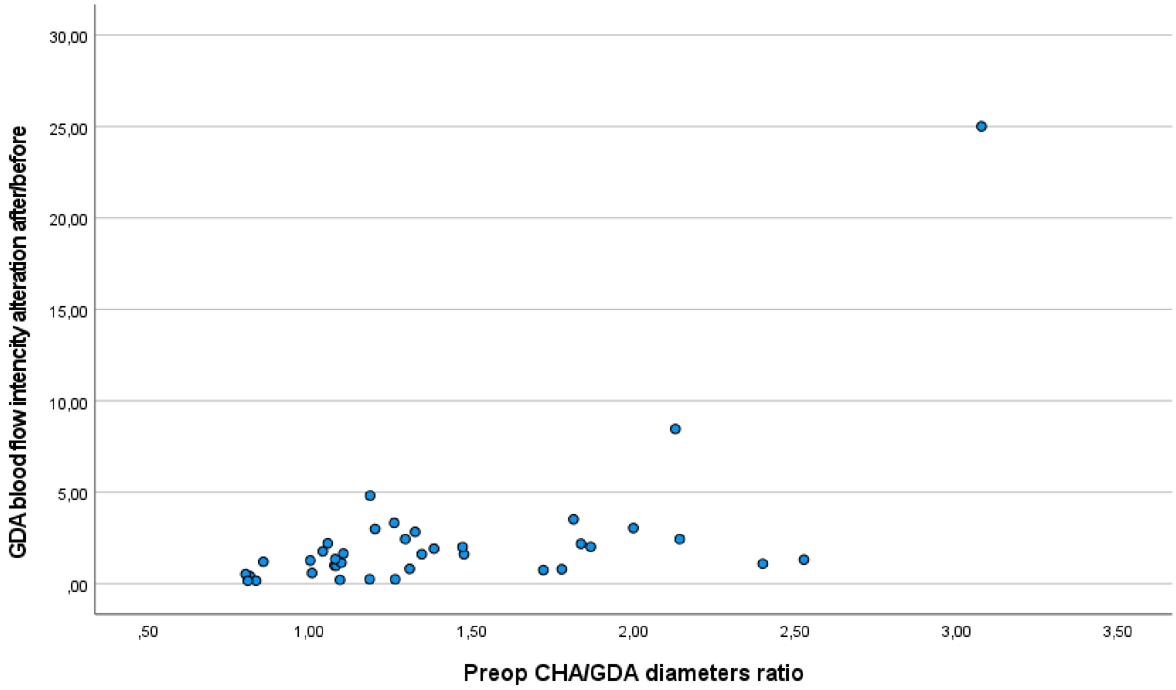


Graph

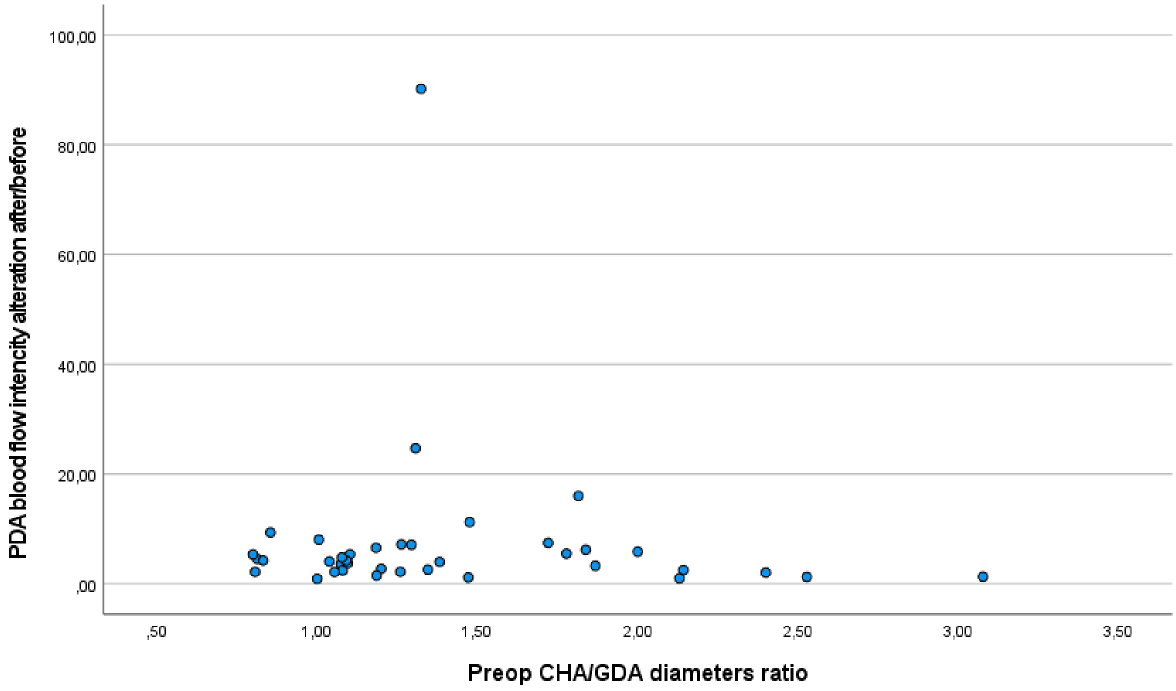


Graph

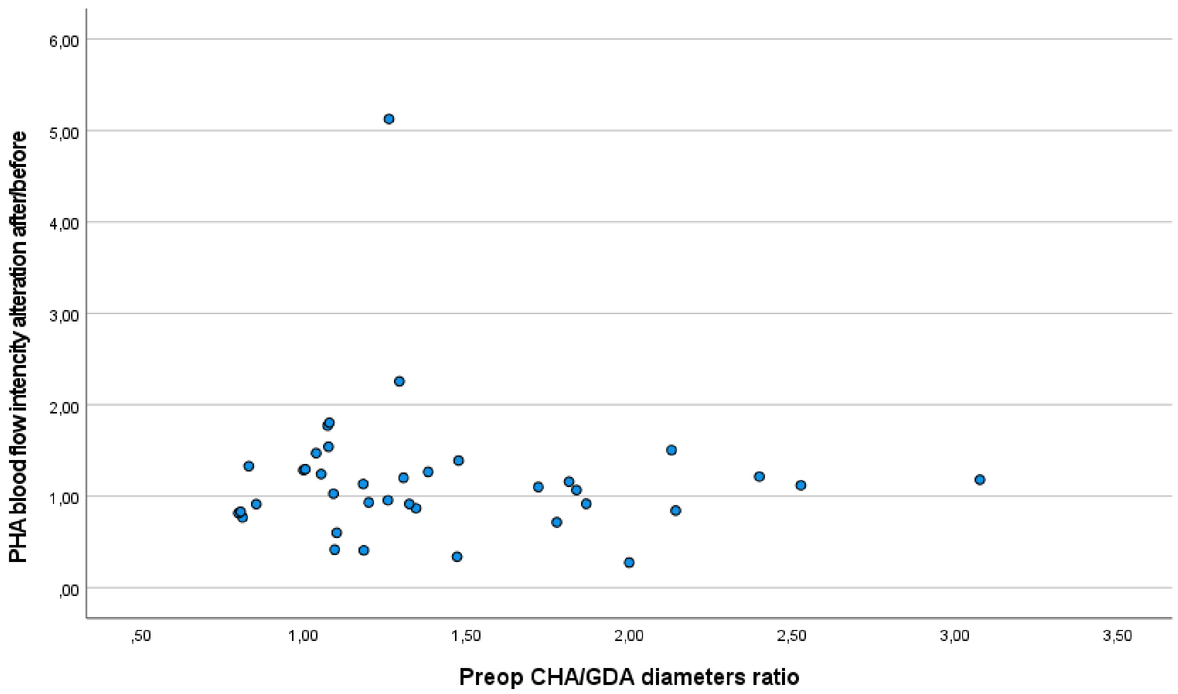


Graph

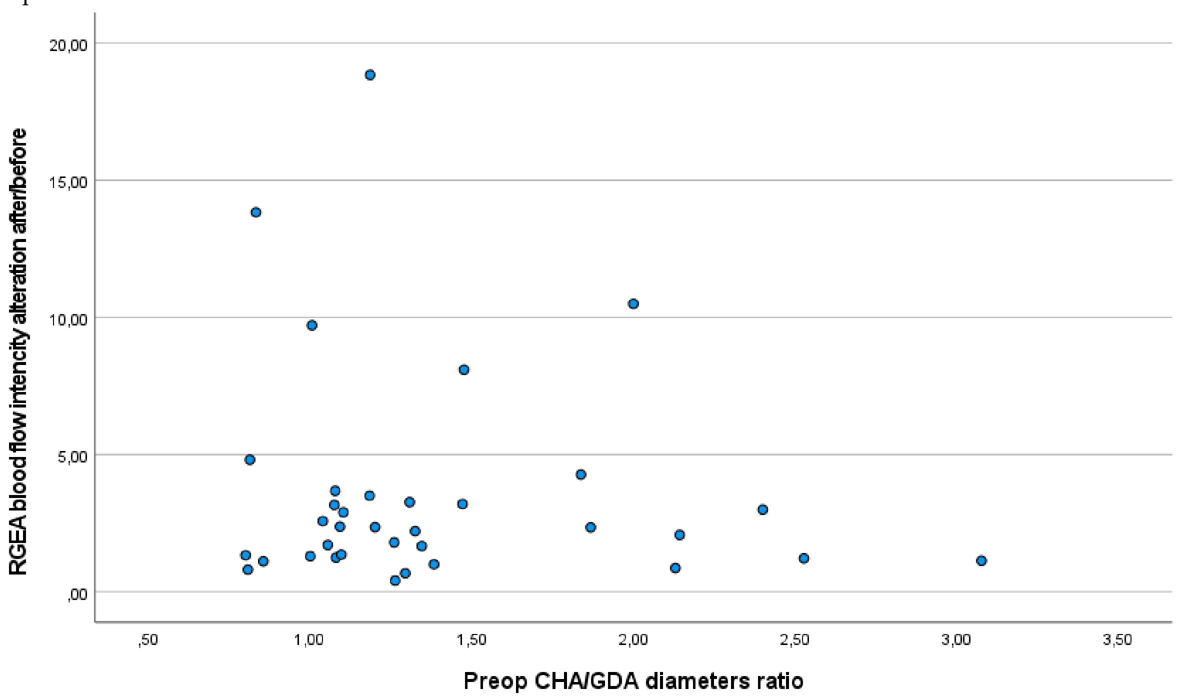


Correlations

**Correlations**

| | Preop CHA/GDA diameters ratio | GDA blood flow intencity alteration after/before | PDA blood flow intencity alteration after/before | PHA blood flow intencity alteration after/before | RGEA blood flow intencity alteration after/before |
|---|---|---|---|---|---|

| | | | | | | |
|---|---|---|---|---|---|---|
| Preop CHA/GDA diameters ratio | Pearson Correlation | 1 | .612 | -.061 | -.061 | -.117 |
| | Sig. (2-tailed) | | .000 | .716 | .716 | .504 |
| | N | 38 | 38 | 38 | 38 | 35 |
| GDA blood flow intencity alteration after/before | Pearson Correlation | .612 | 1 | -.042 | -.057 | -.057 |
| | Sig. (2-tailed) | .000 | | .800 | .735 | .744 |
| | N | 38 | 38 | 38 | 38 | 35 |
| PDA blood flow intencity alteration after/before | Pearson Correlation | -.061 | -.042 | 1 | -.015 | -.033 |
| | Sig. (2-tailed) | .716 | .800 | | .926 | .851 |
| | N | 38 | 38 | 38 | 38 | 35 |
| PHA blood flow intencity alteration after/before | Pearson Correlation | -.061 | -.057 | -.015 | 1 | -.246 |
| | Sig. (2-tailed) | .716 | .735 | .926 | | .155 |
| | N | 38 | 38 | 38 | 38 | 35 |
| RGEA blood flow intencity alteration after/before | Pearson Correlation | -.117 | -.057 | -.033 | -.246 | 1 |
| | Sig. (2-tailed) | .504 | .744 | .851 | .155 | |
| | N | 35 | 35 | 35 | 35 | 35 |

Nonparametric Correlations

**Correlations**

| | | | Preop CHA/GDA diameters ratio | GDA blood flow intencity alteration after/before | PDA blood flow intencity alteration after/before | PHA blood flow intencity alteration after/before | RGEA blood flow intencity alteration after/before |
|---|---|---|---|---|---|---|---|
| Spearman's rho | Preop CHA/GDA diameters ratio | Correlation Coefficient | 1.000 | .535 | -.094 | -.068 | -.066 |
| | | Sig. (2-tailed) | . | .001 | .574 | .686 | .706 |
| | | N | 38 | 38 | 38 | 38 | 35 |
| | GDA blood flow intencity alteration after/before | Correlation Coefficient | .535 | 1.000 | -.246 | -.109 | -.057 |
| | | Sig. (2-tailed) | .001 | . | .137 | .515 | .746 |
| | | N | 38 | 38 | 38 | 38 | 35 |
| | PDA blood flow intencity alteration after/before | Correlation Coefficient | -.094 | -.246 | 1.000 | .072 | .268 |
| | | Sig. (2-tailed) | .574 | .137 | . | .669 | .120 |
| | | N | 38 | 38 | 38 | 38 | 35 |

| | | | | | | | |
|---|---|---|---|---|---|---|---|
| | PHA blood flow intencity alteration after/before | Correlation Coefficient | -.068 | -.109 | .072 | 1.000 | -.198 |
| | | Sig. (2-tailed) | .686 | .515 | .669 | . | .254 |
| | | N | 38 | 38 | 38 | 38 | 35 |
| | RGEA blood flow intencity alteration after/before | Correlation Coefficient | -.066 | -.057 | .268 | -.198 | 1.000 |
| | | Sig. (2-tailed) | .706 | .746 | .120 | .254 | . |
| | | N | 35 | 35 | 35 | 35 | 35 |

Graph

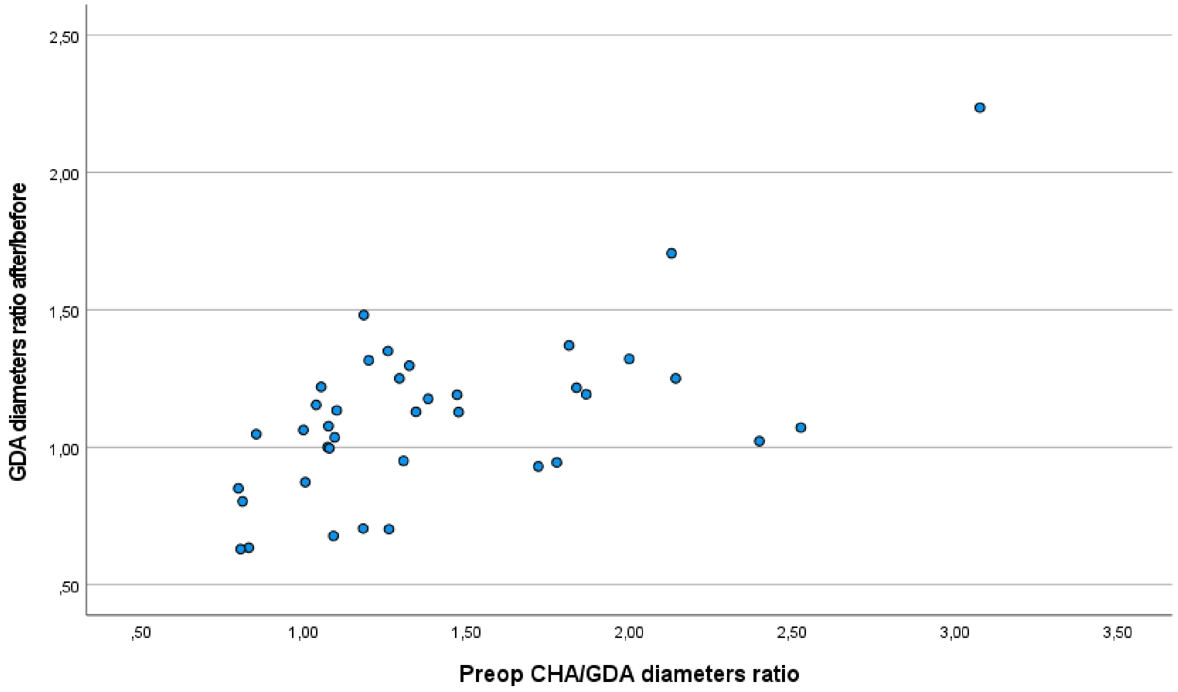


Graph

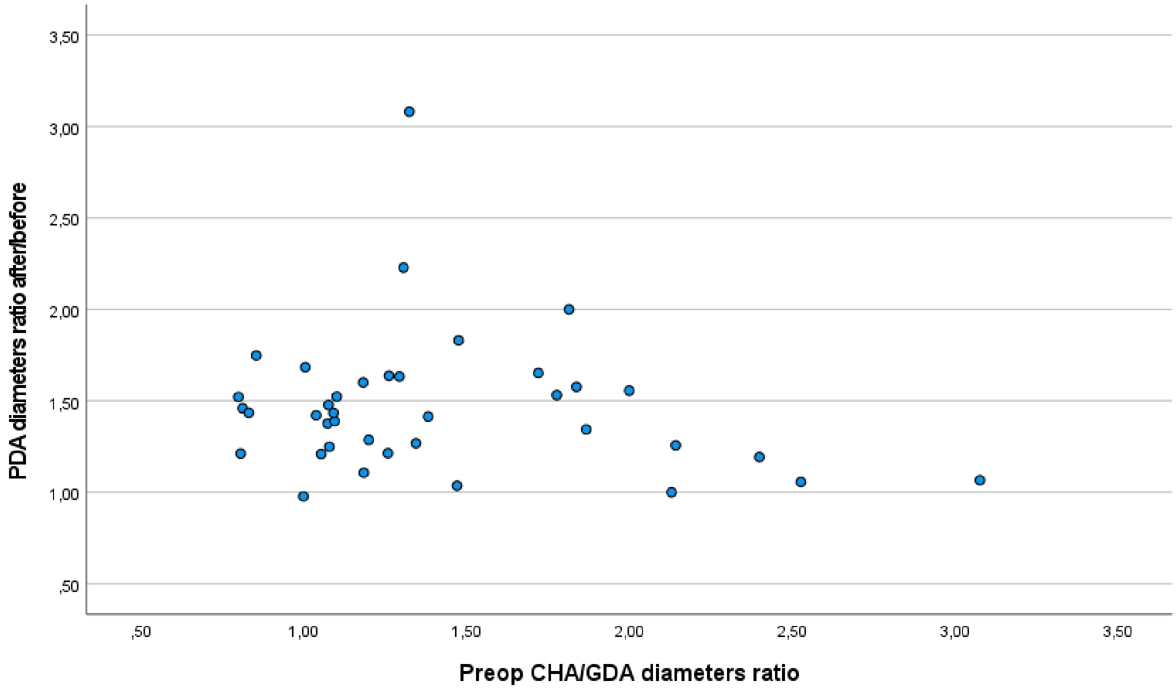
PDA diameters ratio after/before
3,50
3,00
2,50
2,00
1,50
1,00
,50
,50
1,00
1,50
2,00
2,50
3,00
3,50
Preop CHA/GDA diameters ratio

Graph

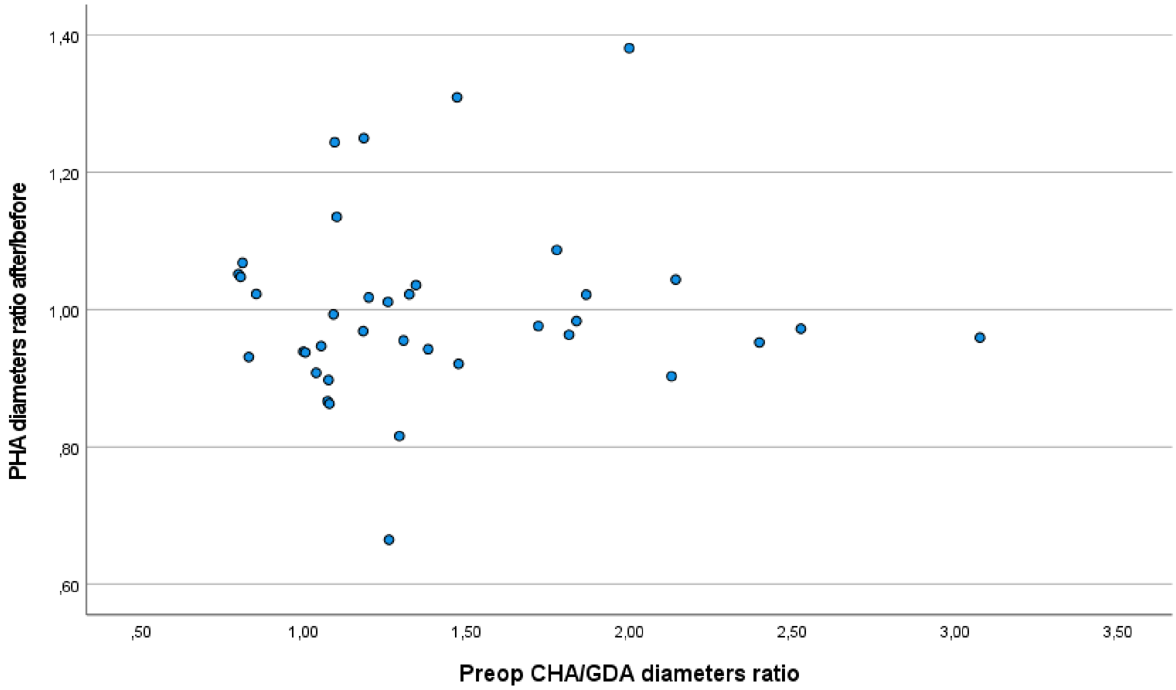
PHA diameters ratio after/before
1,40
1,20
1,00
,80
,60
,50
1,00
1,50
2,00
2,50
3,00
3,50
Preop CHA/GDA diameters ratio

Graph

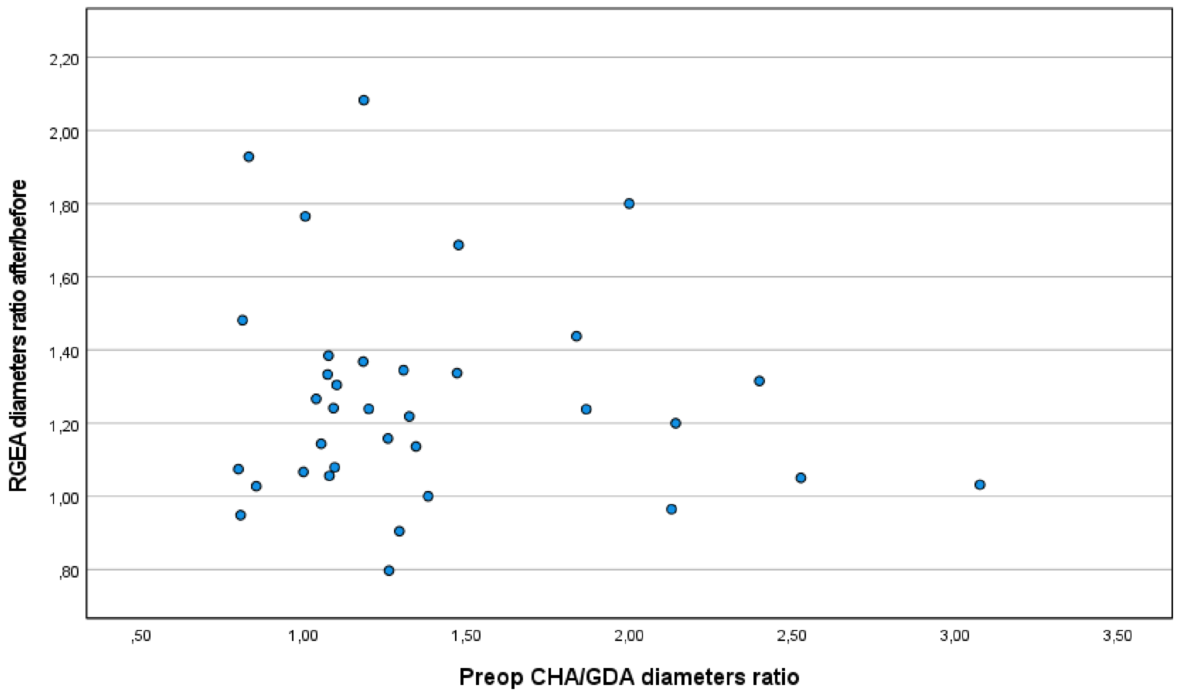


Correlations

**Correlations**

| | | Preop CHA/GDA diameters ratio | GDA diameters ratio after/before | PDA diameters ratio after/before | PHA diameters ratio after/before | RGEA diameters ratio after/before |
|---|---|---|---|---|---|---|
| Preop CHA/GDA diameters ratio | Pearson Correlation | 1 | .620 | -.179 | .047 | -.109 |
| | Sig. (2-tailed) | | .000 | .283 | .778 | .535 |
| | N | 38 | 38 | 38 | 38 | 35 |
| GDA diameters ratio after/before | Pearson Correlation | .620 | 1 | -.159 | .152 | -.073 |
| | Sig. (2-tailed) | .000 | | .341 | .361 | .678 |
| | N | 38 | 38 | 38 | 38 | 35 |
| PDA diameters ratio after/before | Pearson Correlation | -.179 | -.159 | 1 | -.107 | .111 |
| | Sig. (2-tailed) | .283 | .341 | | .522 | .524 |
| | N | 38 | 38 | 38 | 38 | 35 |
| PHA diameters ratio after/before | Pearson Correlation | .047 | .152 | -.107 | 1 | .403 |
| | Sig. (2-tailed) | .778 | .361 | .522 | | .016 |
| | N | 38 | 38 | 38 | 38 | 35 |
| RGEA diameters ratio after/before | Pearson Correlation | -.109 | -.073 | .111 | .403 | 1 |
| | Sig. (2-tailed) | .535 | .678 | .524 | .016 | |
| | N | 35 | 35 | 35 | 35 | 35 |

Nonparametric Correlations

**Correlations**

| | | | Preop CHA/GDA diameters ratio | GDA diameters ratio after/before | PDA diameters ratio after/before | PHA diameters ratio after/before | RGEA diameters ratio after/before |
|---|---|---|---|---|---|---|---|
| Spearman's rho | Preop CHA/GDA diameters ratio | Correlation Coefficient | 1.000 | .534 | -.094 | .068 | -.066 |
| | | Sig. (2-tailed) | . | .001 | .574 | .686 | .706 |
| | | N | 38 | 38 | 38 | 38 | 35 |
| | GDA diameters ratio after/before | Correlation Coefficient | .534 | 1.000 | -.244 | .106 | -.059 |
| | | Sig. (2-tailed) | .001 | . | .140 | .527 | .737 |
| | | N | 38 | 38 | 38 | 38 | 35 |
| | PDA diameters ratio after/before | Correlation Coefficient | -.094 | -.244 | 1.000 | -.072 | .268 |
| | | Sig. (2-tailed) | .574 | .140 | . | .669 | .120 |
| | | N | 38 | 38 | 38 | 38 | 35 |
| | PHA diameters ratio after/before | Correlation Coefficient | .068 | .106 | -.072 | 1.000 | .198 |
| | | Sig. (2-tailed) | .686 | .527 | .669 | . | .254 |
| | | N | 38 | 38 | 38 | 38 | 35 |
| | RGEA diameters ratio after/before | Correlation Coefficient | -.066 | -.059 | .268 | .198 | 1.000 |
| | | Sig. (2-tailed) | .706 | .737 | .120 | .254 | . |
| | | N | 35 | 35 | 35 | 35 | 35 |

Graph

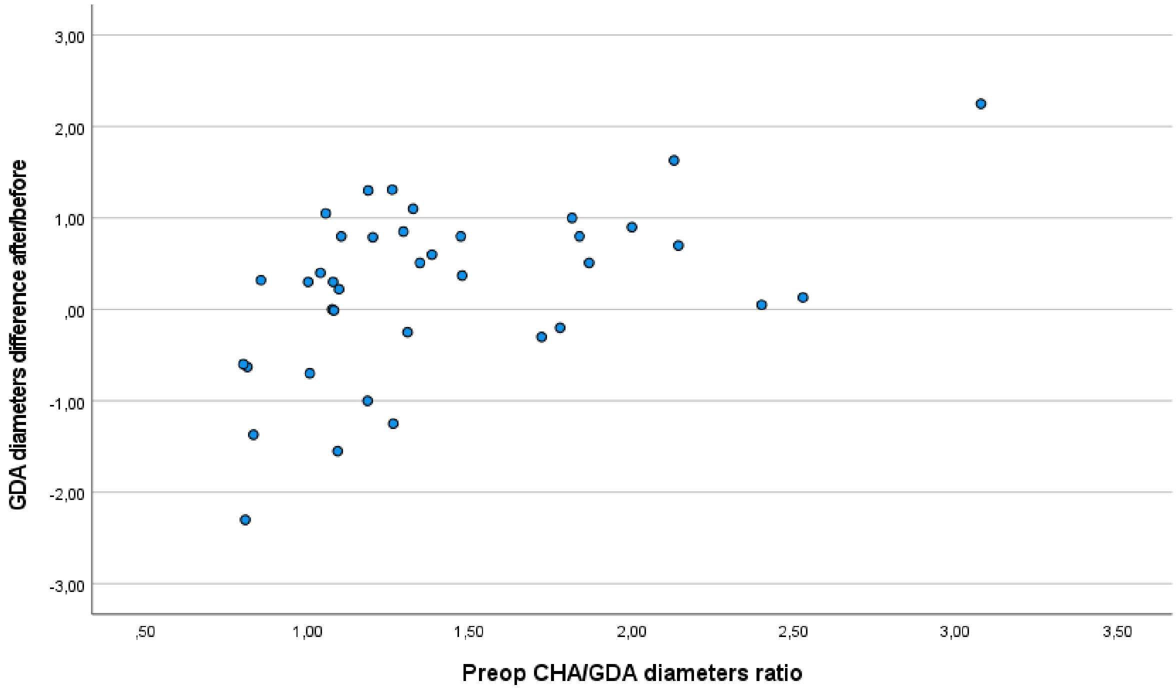
GDA diameters difference after/before
3,00
2,00
1,00
,00
-1,00
-2,00
-3,00
,50
1,00
1,50
2,00
2,50
3,00
3,50
Preop CHA/GDA diameters ratio

Graph

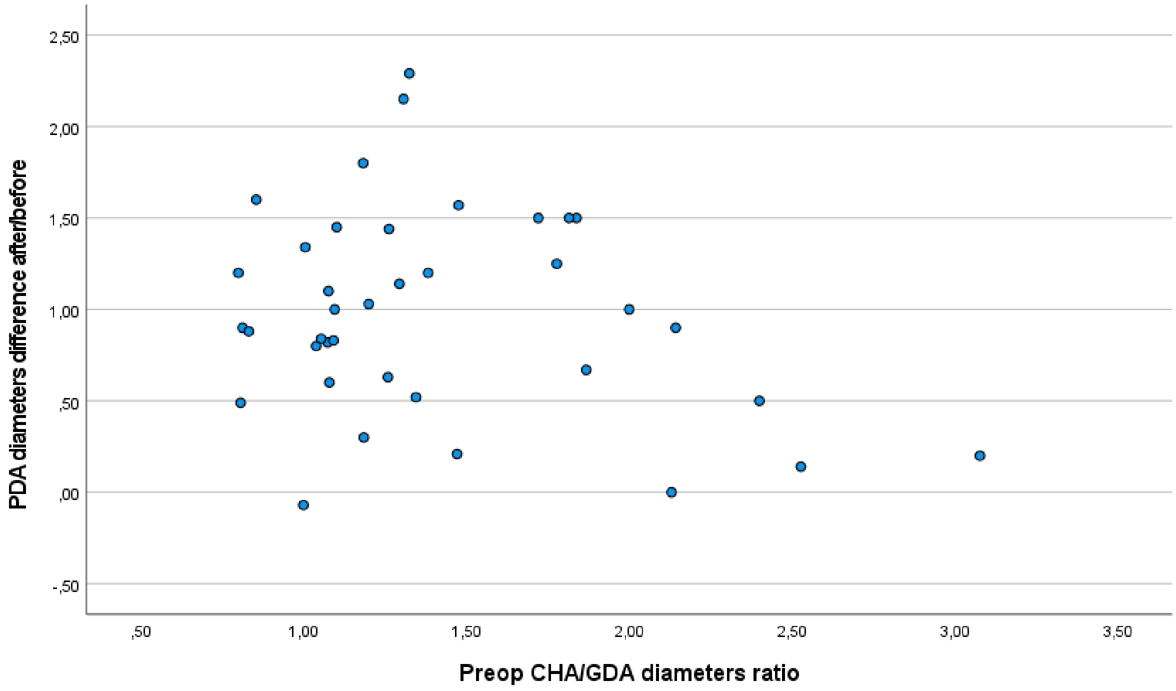
PDA diameters difference after/before
2,50
2,00
1,50
1,00
,50
,00
-,50
,50
1,00
1,50
2,00
2,50
3,00
3,50
Preop CHA/GDA diameters ratio

Graph

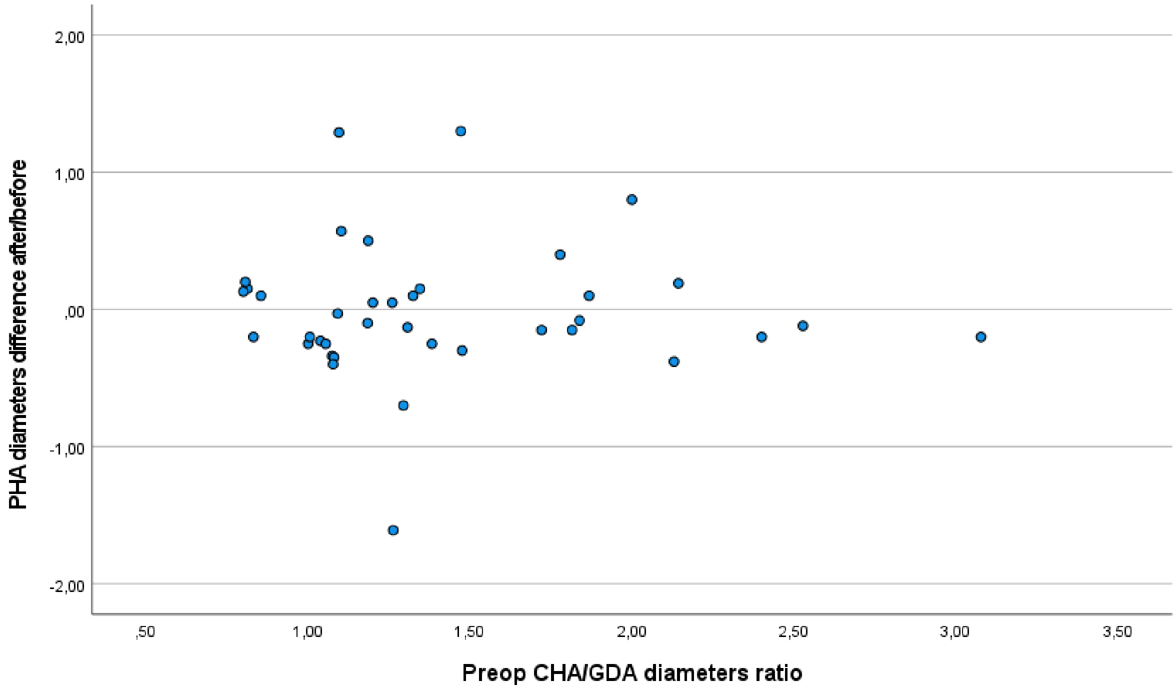


Graph

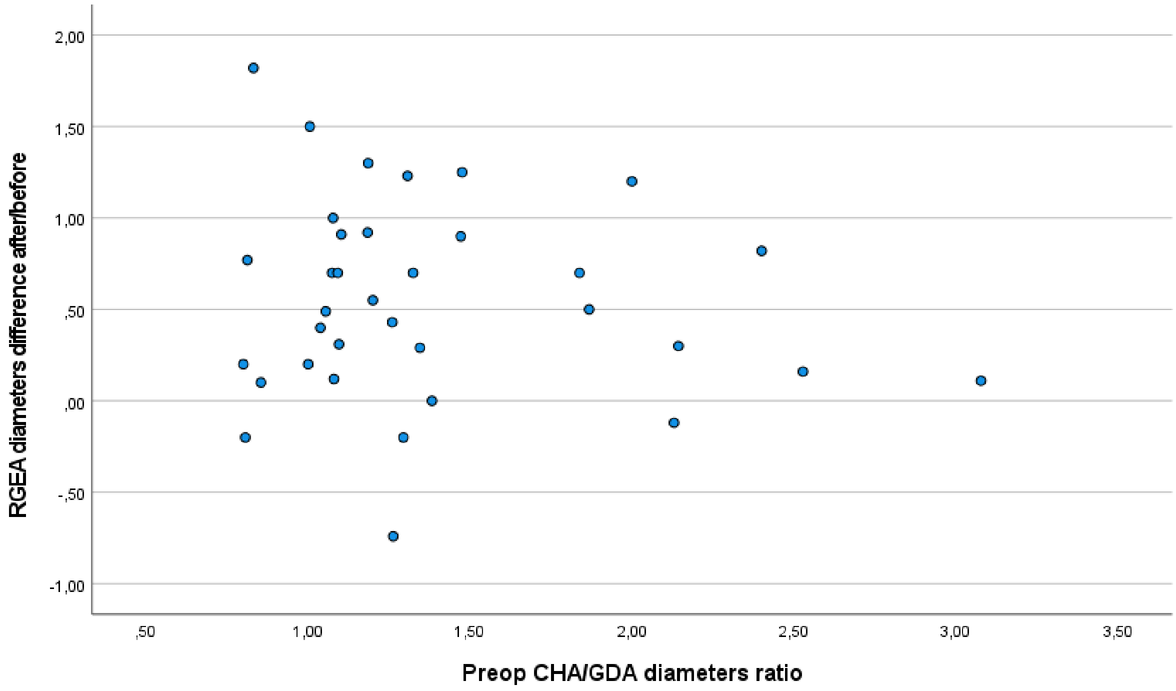


Correlations

**Correlations**

| | | Preop CHA/GDA diameters ratio | GDA diameters difference after/before | PDA diameters difference after/before | PHA diameters difference after/before | RGEA diameters difference after/before |
|---|---|---|---|---|---|---|
| Preop CHA/GDA diameters ratio | Pearson Correlation | 1 | .494 | -.259 | -.008 | -.134 |

| | | | | | | |
|---|---|---|---|---|---|---|
| | Sig. (2-tailed) | | .002 | .117 | .960 | .444 |
| | N | 38 | 38 | 38 | 38 | 35 |
| GDA diameters difference after/before | Pearson Correlation | .494 | 1 | -.187 | .176 | -.055 |
| | Sig. (2-tailed) | .002 | | .261 | .290 | .752 |
| | N | 38 | 38 | 38 | 38 | 35 |
| PDA diameters difference after/before | Pearson Correlation | -.259 | -.187 | 1 | -.126 | .241 |
| | Sig. (2-tailed) | .117 | .261 | | .450 | .163 |
| | N | 38 | 38 | 38 | 38 | 35 |
| PHA diameters difference after/before | Pearson Correlation | -.008 | .176 | -.126 | 1 | .349 |
| | Sig. (2-tailed) | .960 | .290 | .450 | | .040 |
| | N | 38 | 38 | 38 | 38 | 35 |
| RGEA diameters difference after/before | Pearson Correlation | -.134 | -.055 | .241 | .349 | 1 |
| | Sig. (2-tailed) | .444 | .752 | .163 | .040 | |
| | N | 35 | 35 | 35 | 35 | 35 |

Nonparametric Correlations

**Correlations**

| | | | Preop CHA/GDA diameters ratio | GDA diameters difference after/before | PDA diameters difference after/before | PHA diameters difference after/before | RGEA diameters difference after/before |
|---|---|---|---|---|---|---|---|
| Spearman's rho | Preop CHA/GDA diameters ratio | Correlation Coefficient | 1.000 | .474 | -.068 | .026 | -.061 |
| | | Sig. (2-tailed) | . | .003 | .686 | .878 | .726 |
| | | N | 38 | 38 | 38 | 38 | 35 |
| | GDA diameters difference after/before | Correlation Coefficient | .474 | 1.000 | -.166 | .057 | -.095 |
| | | Sig. (2-tailed) | .003 | . | .318 | .732 | .587 |
| | | N | 38 | 38 | 38 | 38 | 35 |
| | PDA diameters difference after/before | Correlation Coefficient | -.068 | -.166 | 1.000 | -.002 | .255 |
| | | Sig. (2-tailed) | .686 | .318 | . | .990 | .139 |
| | | N | 38 | 38 | 38 | 38 | 35 |
| | PHA diameters difference after/before | Correlation Coefficient | .026 | .057 | -.002 | 1.000 | .235 |
| | | Sig. (2-tailed) | .878 | .732 | .990 | . | .174 |
| | | N | 38 | 38 | 38 | 38 | 35 |

| | | | | | | | |
|---|---|---|---|---|---|---|---|
| | RGEA diameters difference after/before | Correlation Coefficient | -.061 | -.095 | .255 | .235 | 1.000 |
| | | Sig. (2-tailed) | .726 | .587 | .139 | .174 | . |
| | | N | 35 | 35 | 35 | 35 | 35 |

Graph

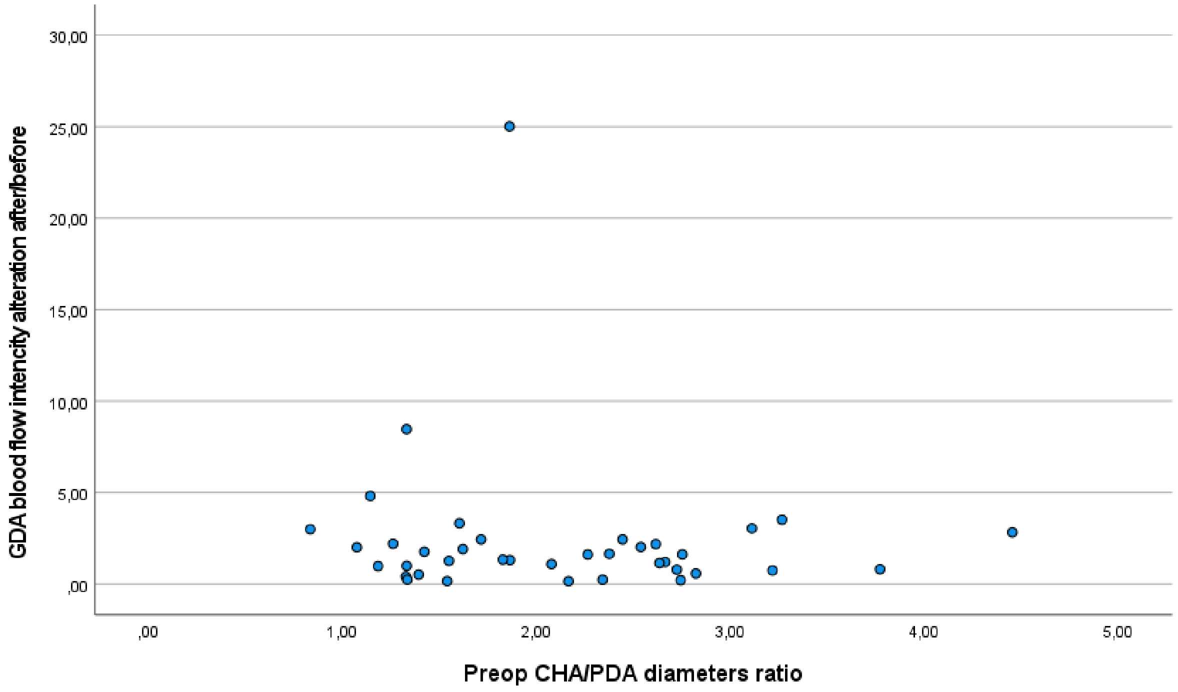


Graph

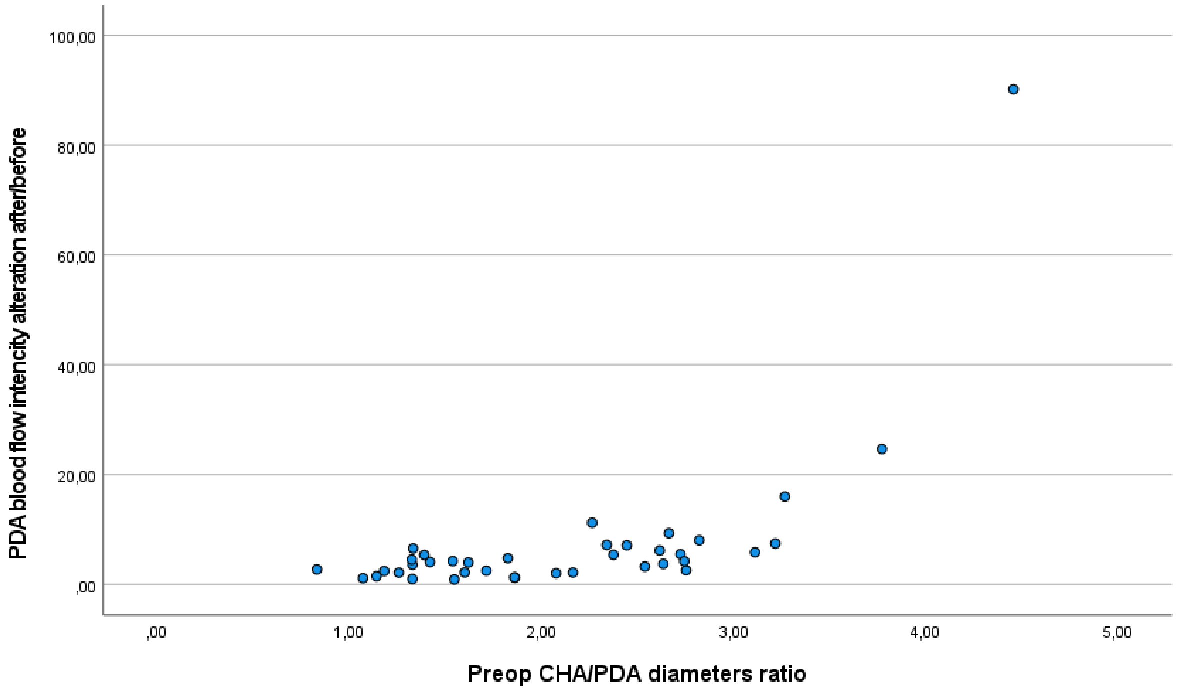


Graph

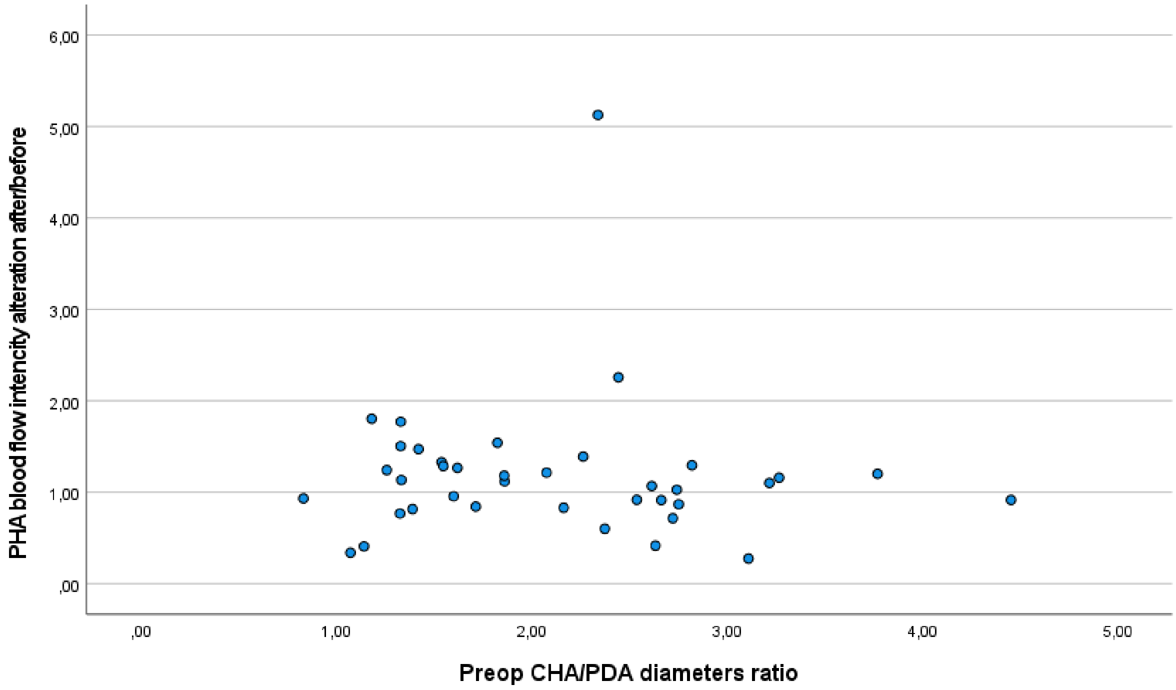


Graph

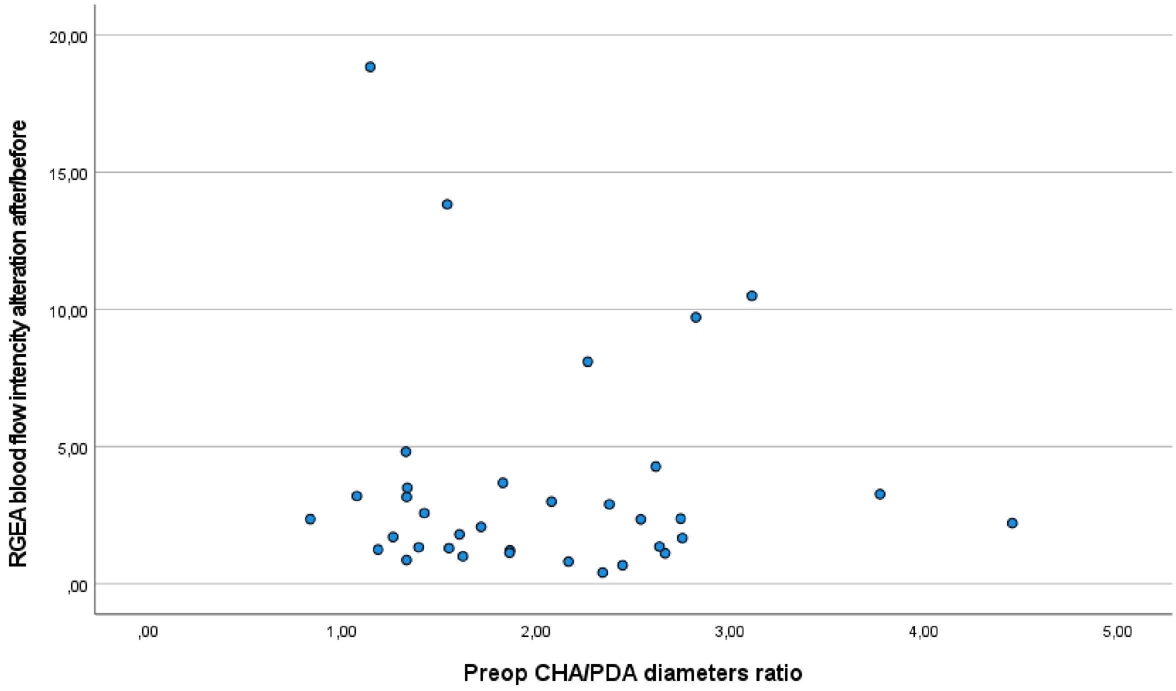


Correlations

**Correlations**

| | Preop CHA/PDA diameters ratio | GDA blood flow intencity alteration after/before | PDA blood flow intencity alteration after/before | PHA blood flow intencity alteration after/before | RGEA blood flow intencity alteration after/before |
|---|---|---|---|---|---|

| | | | | | | |
|---|---|---|---|---|---|---|
| Preop CHA/PDA diameters ratio | Pearson Correlation | 1 | -.092 | .633 | -.030 | -.040 |
| | Sig. (2-tailed) | | .583 | .000 | .857 | .819 |
| | N | 38 | 38 | 38 | 38 | 35 |
| GDA blood flow intencity alteration after/before | Pearson Correlation | -.092 | 1 | -.042 | -.057 | -.057 |
| | Sig. (2-tailed) | .583 | | .800 | .735 | .744 |
| | N | 38 | 38 | 38 | 38 | 35 |
| PDA blood flow intencity alteration after/before | Pearson Correlation | .633 | -.042 | 1 | -.015 | -.033 |
| | Sig. (2-tailed) | .000 | .800 | | .926 | .851 |
| | N | 38 | 38 | 38 | 38 | 35 |
| PHA blood flow intencity alteration after/before | Pearson Correlation | -.030 | -.057 | -.015 | 1 | -.246 |
| | Sig. (2-tailed) | .857 | .735 | .926 | | .155 |
| | N | 38 | 38 | 38 | 38 | 35 |
| RGEA blood flow intencity alteration after/before | Pearson Correlation | -.040 | -.057 | -.033 | -.246 | 1 |
| | Sig. (2-tailed) | .819 | .744 | .851 | .155 | |
| | N | 35 | 35 | 35 | 35 | 35 |

Nonparametric Correlations

**Correlations**

| | | | Preop CHA/PDA diameters ratio | GDA blood flow intencity alteration after/before | PDA blood flow intencity alteration after/before | PHA blood flow intencity alteration after/before | RGEA blood flow intencity alteration after/before |
|---|---|---|---|---|---|---|---|
| Spearman's rho | Preop CHA/PDA diameters ratio | Correlation Coefficient | 1.000 | -.089 | .635 | -.131 | -.023 |
| | | Sig. (2-tailed) | . | .594 | .000 | .432 | .897 |
| | | N | 38 | 38 | 38 | 38 | 35 |
| | GDA blood flow intencity alteration after/before | Correlation Coefficient | -.089 | 1.000 | -.246 | -.109 | -.057 |
| | | Sig. (2-tailed) | .594 | . | .137 | .515 | .746 |
| | | N | 38 | 38 | 38 | 38 | 35 |
| | PDA blood flow intencity alteration after/before | Correlation Coefficient | .635 | -.246 | 1.000 | .072 | .268 |
| | | Sig. (2-tailed) | .000 | .137 | . | .669 | .120 |
| | | N | 38 | 38 | 38 | 38 | 35 |

| | | | | | | | |
|---|---|---|---|---|---|---|---|
| | PHA blood flow intencity alteration after/before | Correlation Coefficient | -.131 | -.109 | .072 | 1.000 | -.198 |
| | | Sig. (2-tailed) | .432 | .515 | .669 | . | .254 |
| | | N | 38 | 38 | 38 | 38 | 35 |
| | RGEA blood flow intencity alteration after/before | Correlation Coefficient | -.023 | -.057 | .268 | -.198 | 1.000 |
| | | Sig. (2-tailed) | .897 | .746 | .120 | .254 | . |
| | | N | 35 | 35 | 35 | 35 | 35 |

Graph

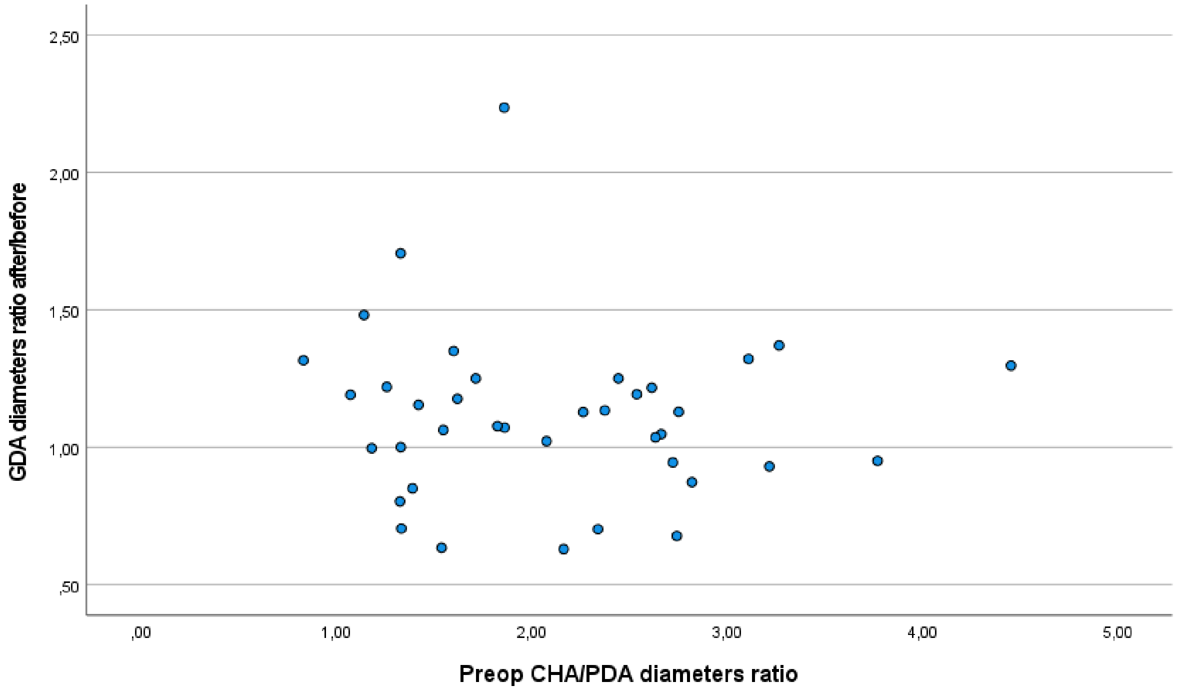


Graph

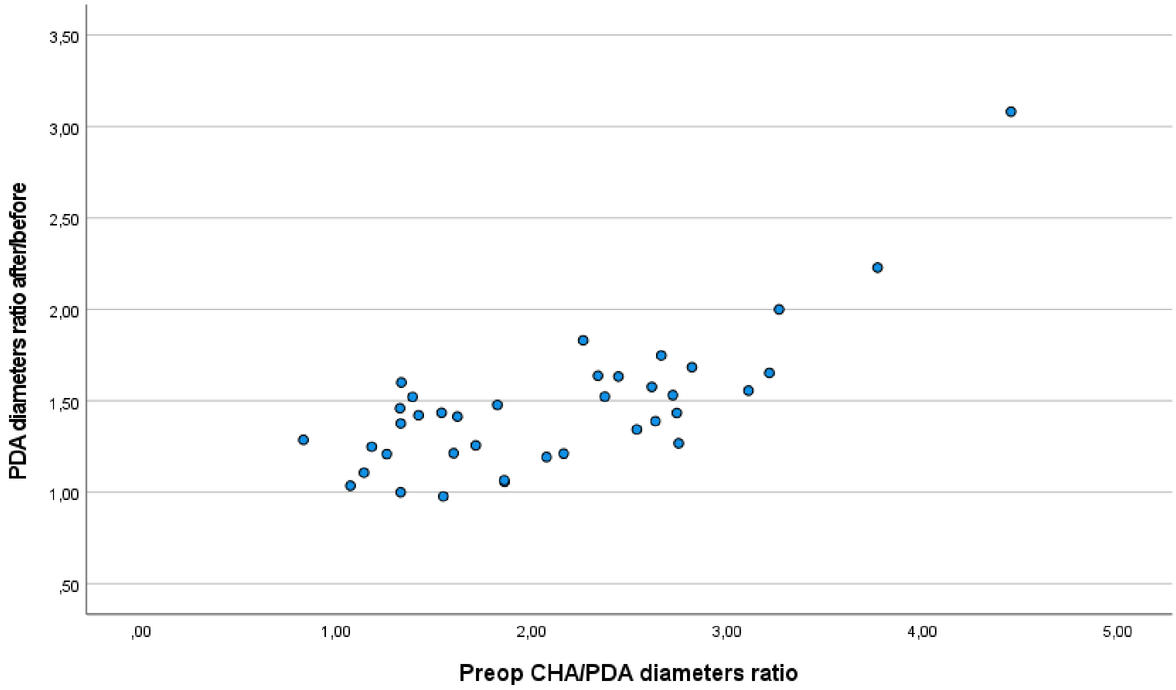
PDA diameters ratio after/before
3,50
3,00
2,50
2,00
1,50
1,00
,50
,00
1,00
2,00
3,00
4,00
5,00
Preop CHA/PDA diameters ratio

Graph

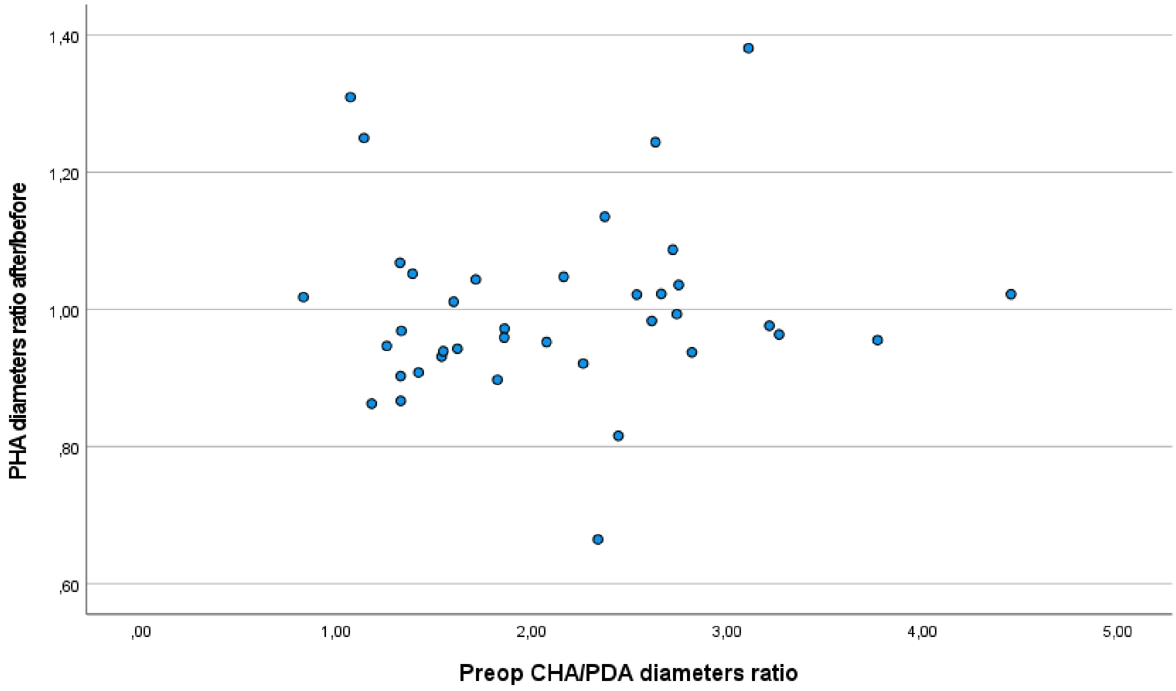
PHA diameters ratio after/before
1,40
1,20
1,00
,80
,60
,00
1,00
2,00
3,00
4,00
5,00
Preop CHA/PDA diameters ratio

Graph

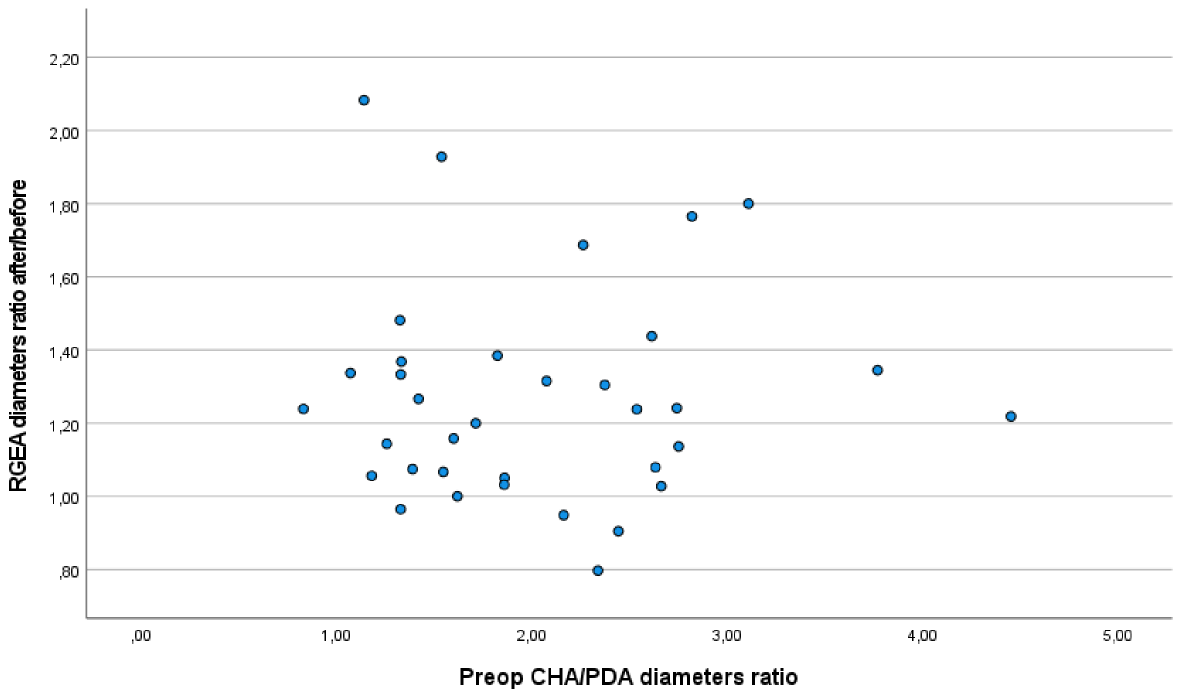


Correlations

**Correlations**

| | | Preop CHA/PDA diameters ratio | GDA diameters ratio after/before | PDA diameters ratio after/before | PHA diameters ratio after/before | RGEA diameters ratio after/before |
|---|---|---|---|---|---|---|
| Preop CHA/PDA diameters ratio | Pearson Correlation | 1 | -.068 | .754 | .059 | .007 |
| | Sig. (2-tailed) | | .687 | .000 | .726 | .968 |
| | N | 38 | 38 | 38 | 38 | 35 |
| GDA diameters ratio after/before | Pearson Correlation | -.068 | 1 | -.159 | .152 | -.073 |
| | Sig. (2-tailed) | .687 | | .341 | .361 | .678 |
| | N | 38 | 38 | 38 | 38 | 35 |
| PDA diameters ratio after/before | Pearson Correlation | .754 | -.159 | 1 | -.107 | .111 |
| | Sig. (2-tailed) | .000 | .341 | | .522 | .524 |
| | N | 38 | 38 | 38 | 38 | 35 |
| PHA diameters ratio after/before | Pearson Correlation | .059 | .152 | -.107 | 1 | .403 |
| | Sig. (2-tailed) | .726 | .361 | .522 | | .016 |
| | N | 38 | 38 | 38 | 38 | 35 |
| RGEA diameters ratio after/before | Pearson Correlation | .007 | -.073 | .111 | .403 | 1 |
| | Sig. (2-tailed) | .968 | .678 | .524 | .016 | |
| | N | 35 | 35 | 35 | 35 | 35 |

Nonparametric Correlations

**Correlations**

| | | | Preop CHA/PDA diameters ratio | GDA diameters ratio after/before | PDA diameters ratio after/before | PHA diameters ratio after/before | RGEA diameters ratio after/before |
|---|---|---|---|---|---|---|---|
| Spearman's rho | Preop CHA/PDA diameters ratio | Correlation Coefficient | 1.000 | -.088 | .635 | .131 | -.023 |
| | | Sig. (2-tailed) | . | .598 | .000 | .432 | .897 |
| | | N | 38 | 38 | 38 | 38 | 35 |
| | GDA diameters ratio after/before | Correlation Coefficient | -.088 | 1.000 | -.244 | .106 | -.059 |
| | | Sig. (2-tailed) | .598 | . | .140 | .527 | .737 |
| | | N | 38 | 38 | 38 | 38 | 35 |
| | PDA diameters ratio after/before | Correlation Coefficient | .635 | -.244 | 1.000 | -.072 | .268 |
| | | Sig. (2-tailed) | .000 | .140 | . | .669 | .120 |
| | | N | 38 | 38 | 38 | 38 | 35 |
| | PHA diameters ratio after/before | Correlation Coefficient | .131 | .106 | -.072 | 1.000 | .198 |
| | | Sig. (2-tailed) | .432 | .527 | .669 | . | .254 |
| | | N | 38 | 38 | 38 | 38 | 35 |
| | RGEA diameters ratio after/before | Correlation Coefficient | -.023 | -.059 | .268 | .198 | 1.000 |
| | | Sig. (2-tailed) | .897 | .737 | .120 | .254 | . |
| | | N | 35 | 35 | 35 | 35 | 35 |

Graph

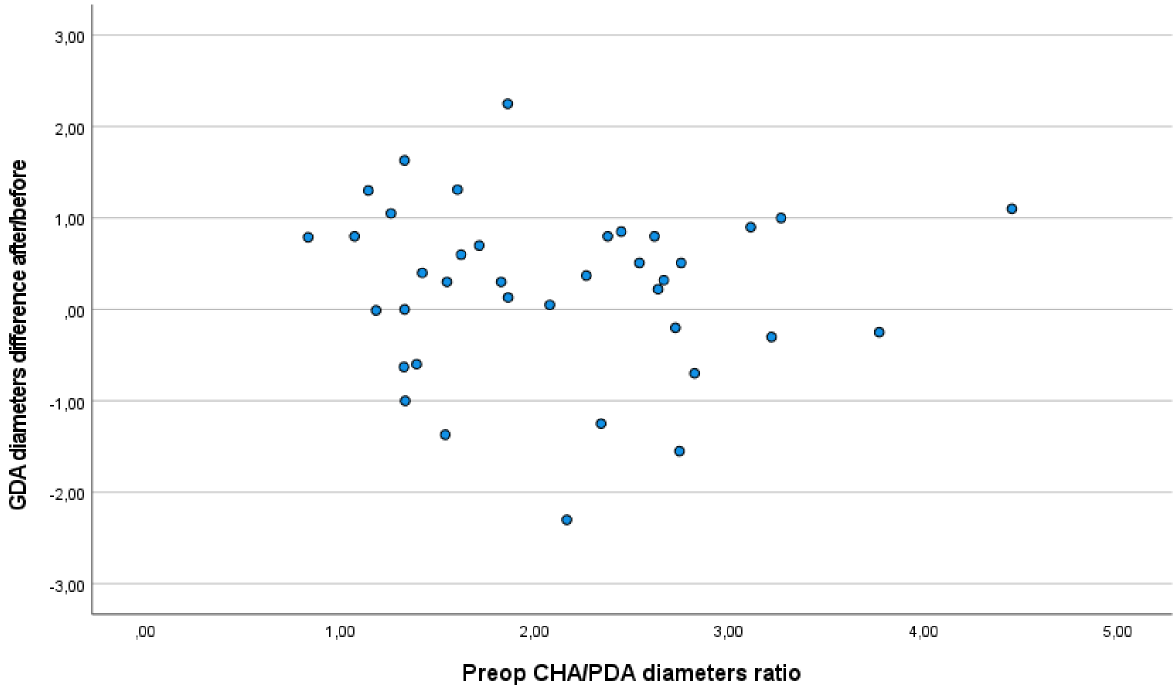

3,00
2,00
1,00
,00
-1,00
-2,00
-3,00
GDA diameters difference after/before
,00
1,00
2,00
3,00
4,00
5,00
Preop CHA/PDA diameters ratio


Graph

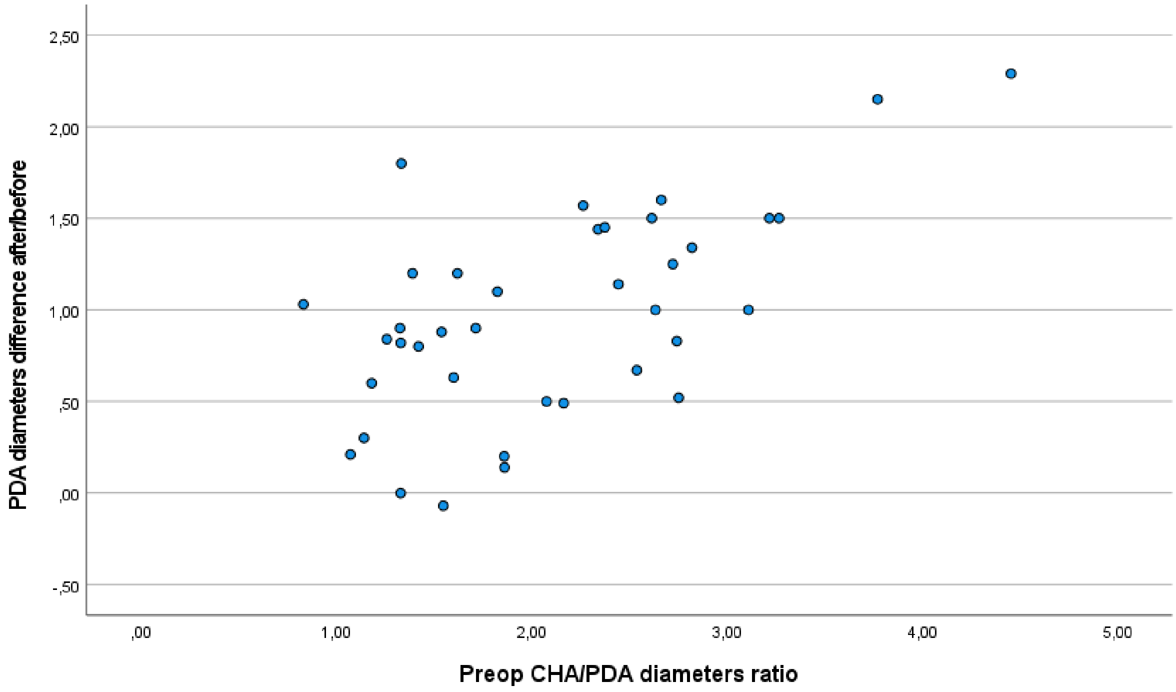

2,50
2,00
1,50
1,00
,50
,00
-,50
PDA diameters difference after/before
,00
1,00
2,00
3,00
4,00
5,00
Preop CHA/PDA diameters ratio


Graph

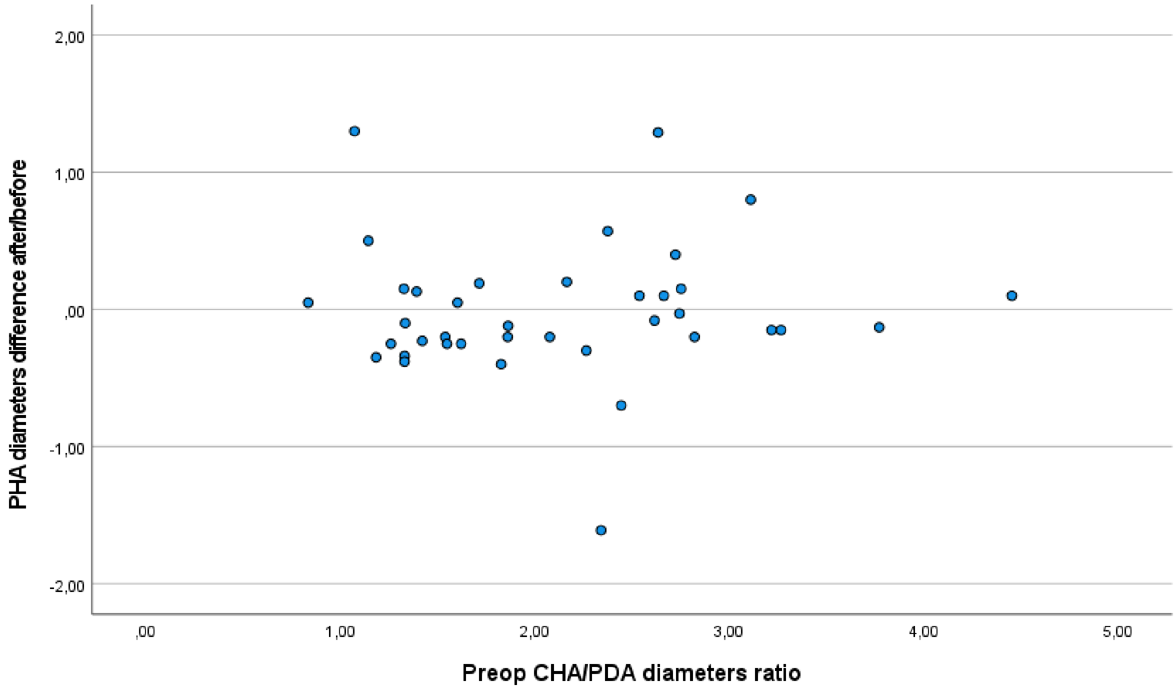


Graph

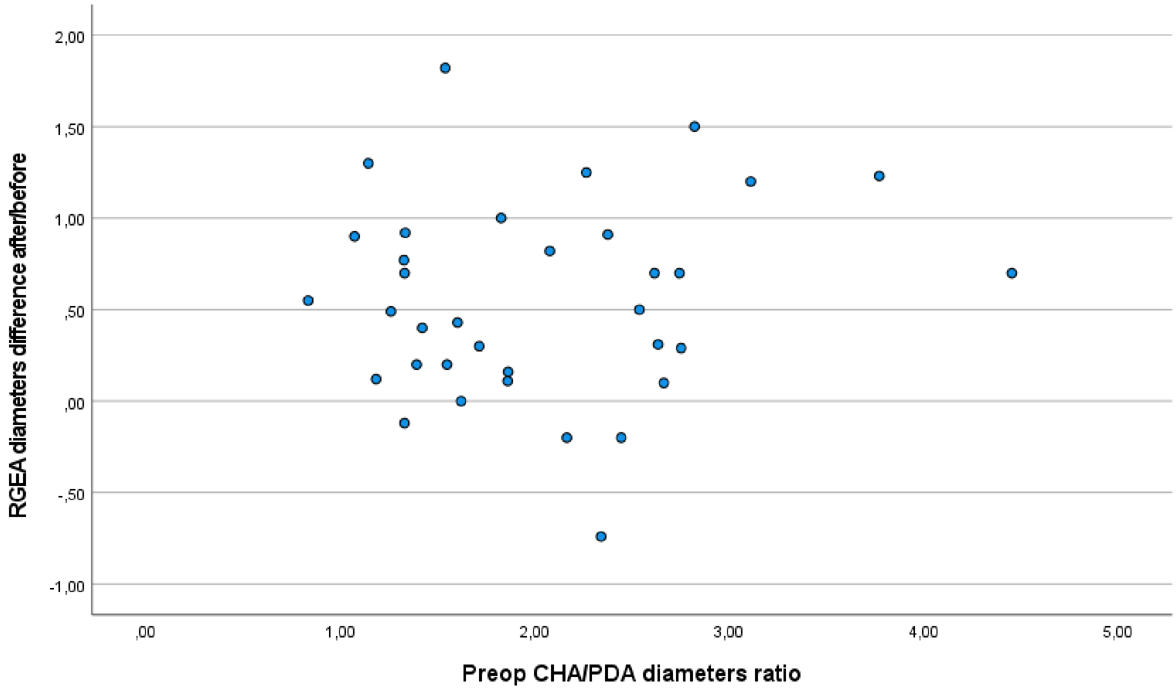


Correlations

**Correlations**

| | | Preop CHA/PDA diameters ratio | GDA diameters difference after/before | PDA diameters difference after/before | PHA diameters difference after/before | RGEA diameters difference after/before |
|---|---|---|---|---|---|---|
| Preop CHA/PDA diameters ratio | Pearson Correlation | 1 | -.052 | .607 | .040 | .113 |
| | Sig. (2-tailed) | | .758 | .000 | .810 | .518 |

| | | | | | | |
|---|---|---|---|---|---|---|
| | N | 38 | 38 | 38 | 38 | 35 |
| GDA diameters difference after/before | Pearson Correlation | -.052 | 1 | -.187 | .176 | -.055 |
| | Sig. (2-tailed) | .758 | | .261 | .290 | .752 |
| | N | 38 | 38 | 38 | 38 | 35 |
| PDA diameters difference after/before | Pearson Correlation | .607 | -.187 | 1 | -.126 | .241 |
| | Sig. (2-tailed) | .000 | .261 | | .450 | .163 |
| | N | 38 | 38 | 38 | 38 | 35 |
| PHA diameters difference after/before | Pearson Correlation | .040 | .176 | -.126 | 1 | .349 |
| | Sig. (2-tailed) | .810 | .290 | .450 | | .040 |
| | N | 38 | 38 | 38 | 38 | 35 |
| RGEA diameters difference after/before | Pearson Correlation | .113 | -.055 | .241 | .349 | 1 |
| | Sig. (2-tailed) | .518 | .752 | .163 | .040 | |
| | N | 35 | 35 | 35 | 35 | 35 |

Nonparametric Correlations

**Correlations**

| | | | Preop CHA/PDA diameters ratio | GDA diameters difference after/before | PDA diameters difference after/before | PHA diameters difference after/before | RGEA diameters difference after/before |
|---|---|---|---|---|---|---|---|
| Spearman's rho | Preop CHA/PDA diameters ratio | Correlation Coefficient | 1.000 | -.089 | .511 | .144 | .051 |
| | | Sig. (2-tailed) | . | .597 | .001 | .389 | .771 |
| | | N | 38 | 38 | 38 | 38 | 35 |
| | GDA diameters difference after/before | Correlation Coefficient | -.089 | 1.000 | -.166 | .057 | -.095 |
| | | Sig. (2-tailed) | .597 | . | .318 | .732 | .587 |
| | | N | 38 | 38 | 38 | 38 | 35 |
| | PDA diameters difference after/before | Correlation Coefficient | .511 | -.166 | 1.000 | -.002 | .255 |
| | | Sig. (2-tailed) | .001 | .318 | . | .990 | .139 |
| | | N | 38 | 38 | 38 | 38 | 35 |
| | PHA diameters difference after/before | Correlation Coefficient | .144 | .057 | -.002 | 1.000 | .235 |
| | | Sig. (2-tailed) | .389 | .732 | .990 | . | .174 |
| | | N | 38 | 38 | 38 | 38 | 35 |

| | | | | | | | |
|---|---|---|---|---|---|---|---|
| | RGEA diameters difference after/before | Correlation Coefficient | .051 | -.095 | .255 | .235 | 1.000 |
| | | Sig. (2-tailed) | .771 | .587 | .139 | .174 | . |
| | | N | 35 | 35 | 35 | 35 | 35 |

Graph

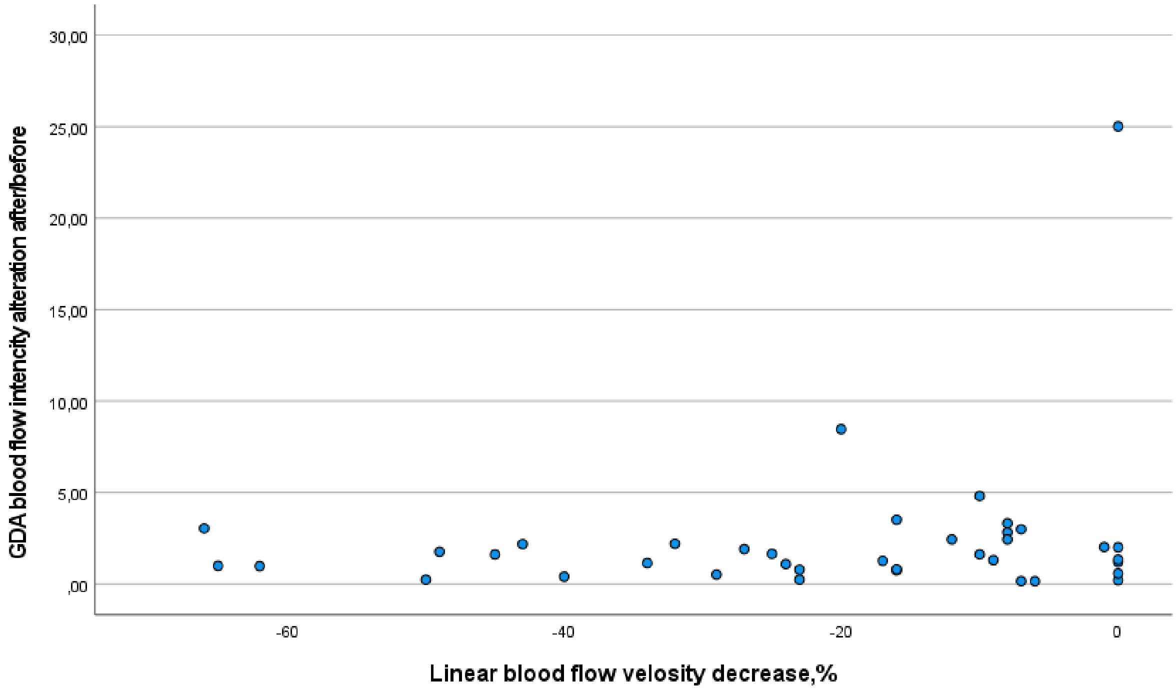


Graph

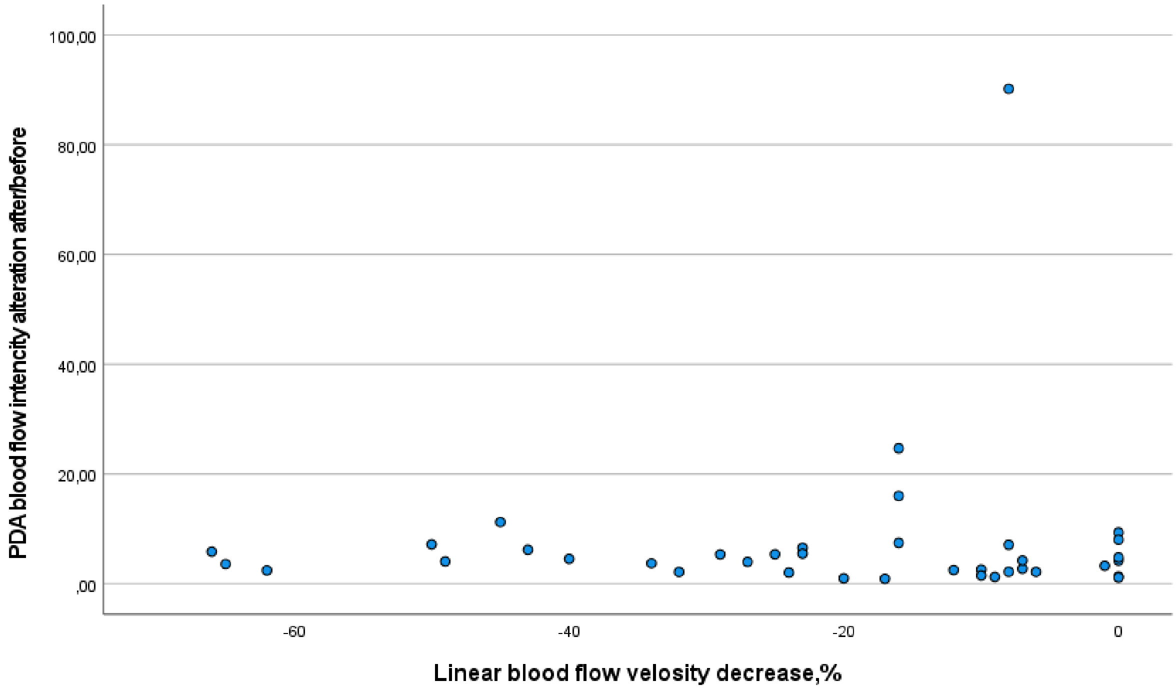


Graph

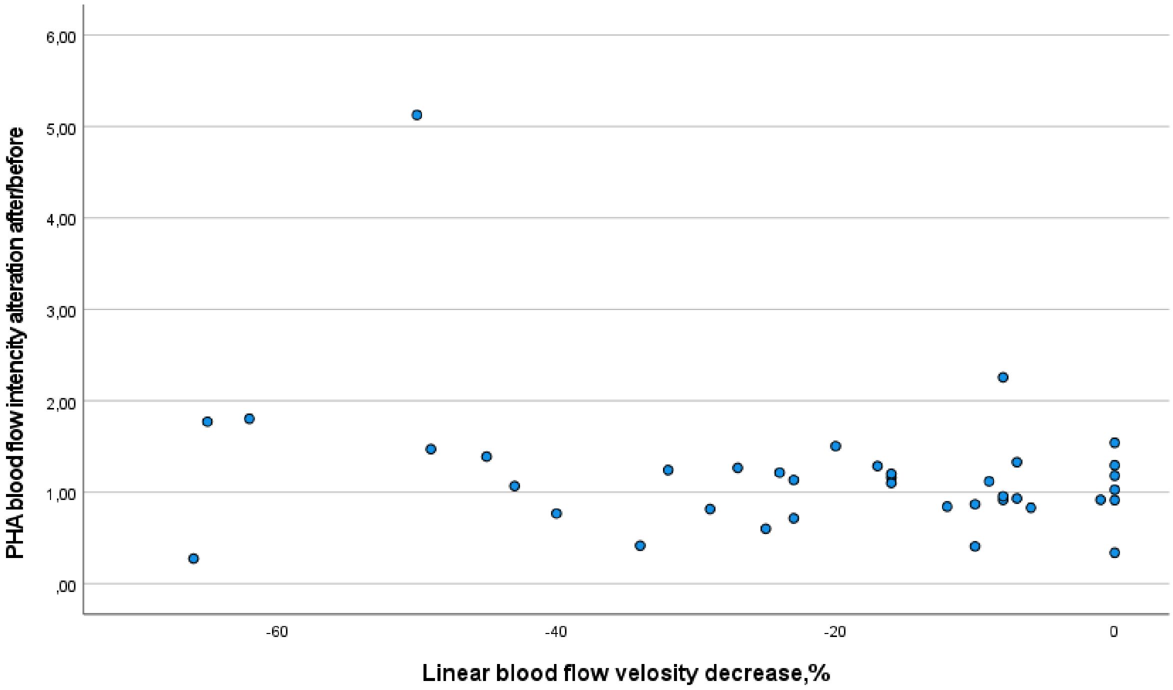


Graph

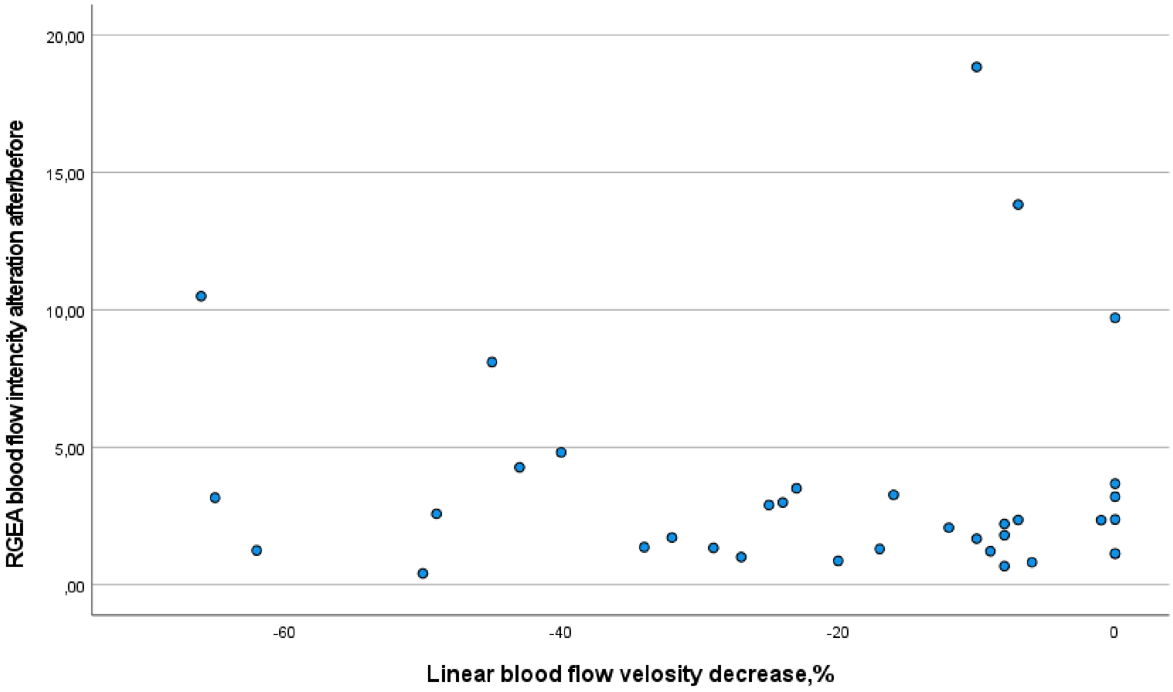


Correlations

**Correlations**

| | | Linear blood flow velosity decrease.% | GDA blood flow intencity alteration after/before | PDA blood flow intencity alteration after/before | PHA blood flow intencity alteration after/before | RGEA blood flow intencity alteration after/before |
|---|---|---|---|---|---|---|
| Linear blood flow velosity decrease.% | Pearson Correlation | 1 | .195 | .093 | -.270 | -.025 |

| | | | | | | |
|---|---|---|---|---|---|---|
| | Sig. (2-tailed) | | .241 | .578 | .101 | .885 |
| | N | 38 | 38 | 38 | 38 | 35 |
| GDA blood flow intencity alteration after/before | Pearson Correlation | .195 | 1 | -.042 | -.057 | -.057 |
| | Sig. (2-tailed) | .241 | | .800 | .735 | .744 |
| | N | 38 | 38 | 38 | 38 | 35 |
| PDA blood flow intencity alteration after/before | Pearson Correlation | .093 | -.042 | 1 | -.015 | -.033 |
| | Sig. (2-tailed) | .578 | .800 | | .926 | .851 |
| | N | 38 | 38 | 38 | 38 | 35 |
| PHA blood flow intencity alteration after/before | Pearson Correlation | -.270 | -.057 | -.015 | 1 | -.246 |
| | Sig. (2-tailed) | .101 | .735 | .926 | | .155 |
| | N | 38 | 38 | 38 | 38 | 35 |
| RGEA blood flow intencity alteration after/before | Pearson Correlation | -.025 | -.057 | -.033 | -.246 | 1 |
| | Sig. (2-tailed) | .885 | .744 | .851 | .155 | |
| | N | 35 | 35 | 35 | 35 | 35 |

Nonparametric Correlations

**Correlations**

| | | | Linear blood flow velosity decrease.% | GDA blood flow intencity alteration after/before | PDA blood flow intencity alteration after/before | PHA blood flow intencity alteration after/before | RGEA blood flow intencity alteration after/before |
|---|---|---|---|---|---|---|---|
| Spearman's rho | Linear blood flow velosity decrease.% | Correlation Coefficient | 1.000 | .085 | -.114 | -.142 | -.063 |
| | | Sig. (2-tailed) | . | .613 | .496 | .397 | .719 |
| | | N | 38 | 38 | 38 | 38 | 35 |
| | GDA blood flow intencity alteration after/before | Correlation Coefficient | .085 | 1.000 | -.246 | -.109 | -.057 |
| | | Sig. (2-tailed) | .613 | . | .137 | .515 | .746 |
| | | N | 38 | 38 | 38 | 38 | 35 |
| | PDA blood flow intencity alteration after/before | Correlation Coefficient | -.114 | -.246 | 1.000 | .072 | .268 |
| | | Sig. (2-tailed) | .496 | .137 | . | .669 | .120 |
| | | N | 38 | 38 | 38 | 38 | 35 |
| | | Correlation Coefficient | -.142 | -.109 | .072 | 1.000 | -.198 |

| | | | | | | |
|---|---|---|---|---|---|---|
| PHA blood flow intencity alteration after/before | Sig. (2-tailed) | .397 | .515 | .669 | . | .254 |
| | N | 38 | 38 | 38 | 38 | 35 |
| RGEA blood flow intencity alteration after/before | Correlation Coefficient | -.063 | -.057 | .268 | -.198 | 1.000 |
| | Sig. (2-tailed) | .719 | .746 | .120 | .254 | . |
| | N | 35 | 35 | 35 | 35 | 35 |

Graph

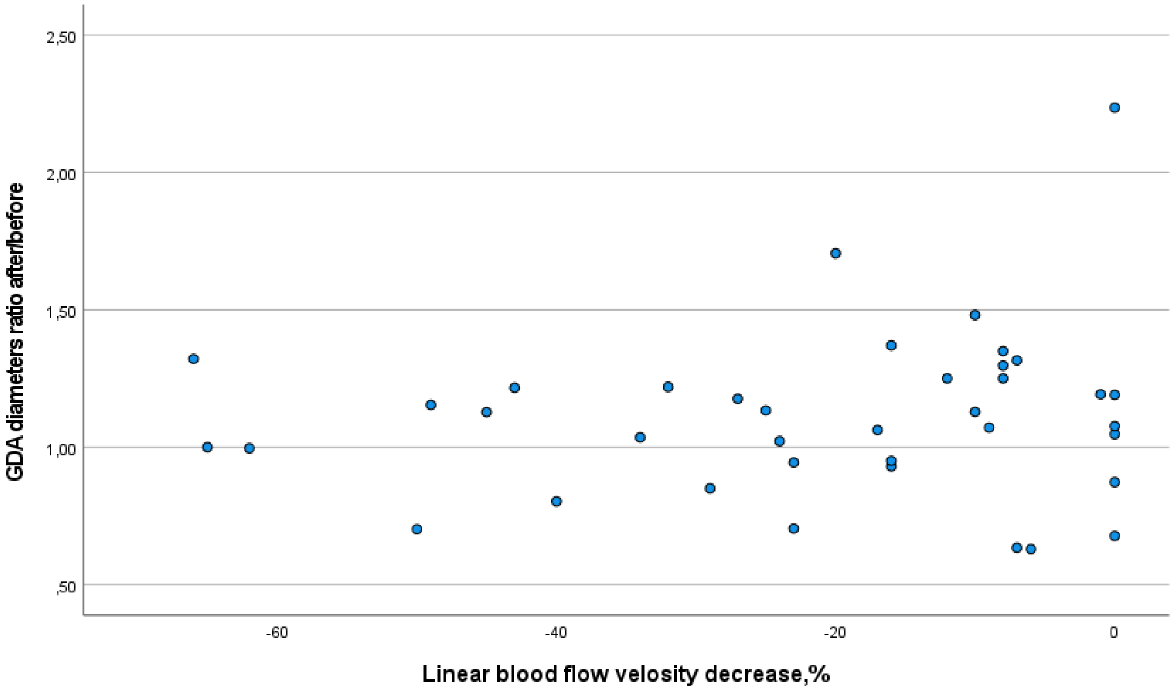


Graph

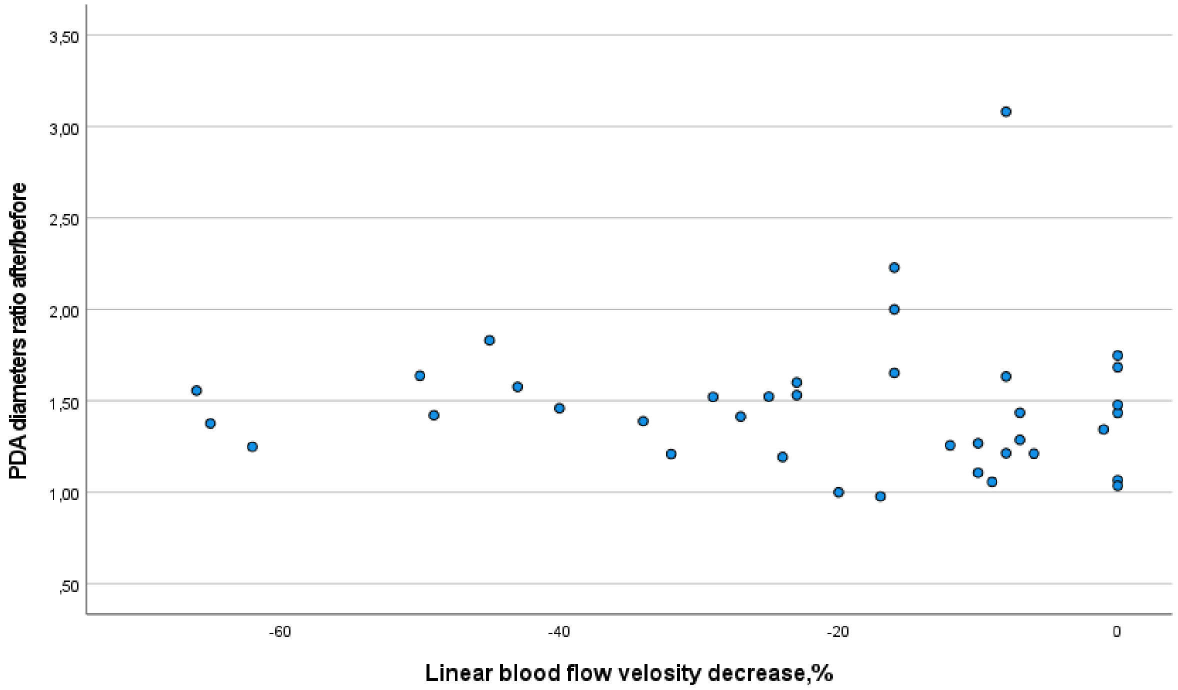
3,50
3,00
2,50
2,00
1,50
1,00
,50
PDA diameters ratio after/before
-60
-40
-20
0
Linear blood flow velosity decrease,%

Graph

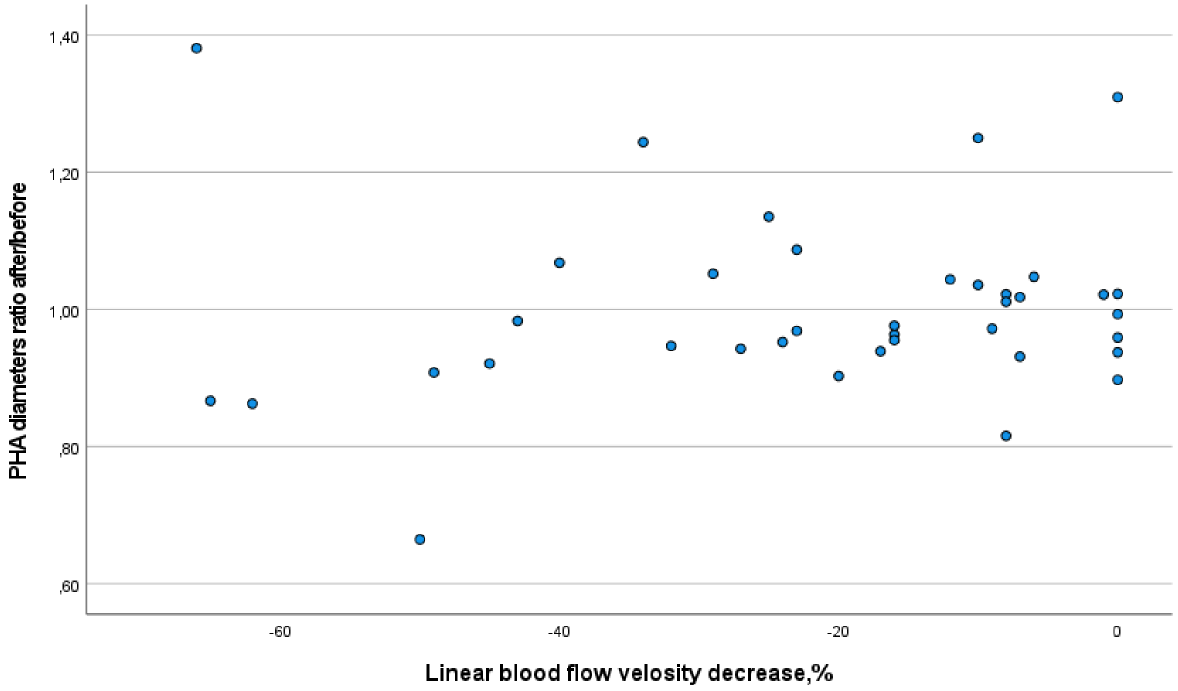
1,40
1,20
1,00
,80
,60
PHA diameters ratio after/before
-60
-40
-20
0
Linear blood flow velosity decrease,%

Graph

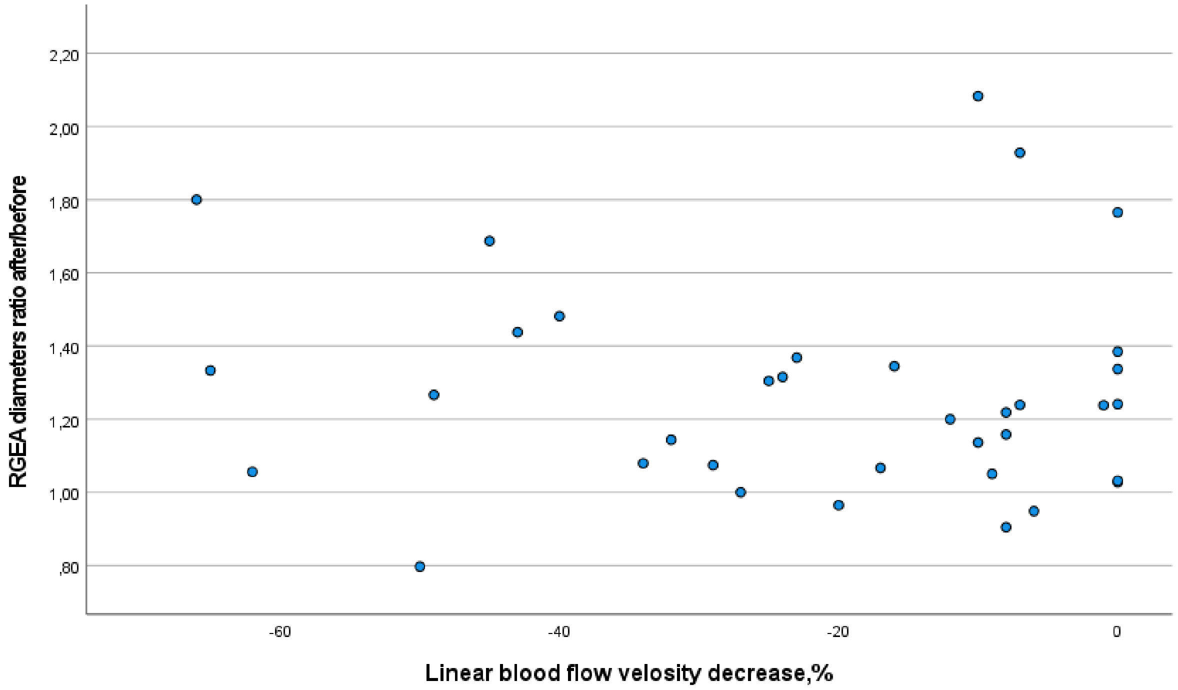


Correlations

**Correlations**

| | | Linear blood flow velosity decrease.% | GDA diameters ratio after/before | PDA diameters ratio after/before | PHA diameters ratio after/before | RGEA diameters ratio after/before |
|---|---|---|---|---|---|---|
| Linear blood flow velosity decrease.% | Pearson Correlation | 1 | .132 | -.024 | .082 | -.059 |
| | Sig. (2-tailed) | | .428 | .886 | .624 | .738 |
| | N | 38 | 38 | 38 | 38 | 35 |
| GDA diameters ratio after/before | Pearson Correlation | .132 | 1 | -.159 | .152 | -.073 |
| | Sig. (2-tailed) | .428 | | .341 | .361 | .678 |
| | N | 38 | 38 | 38 | 38 | 35 |
| PDA diameters ratio after/before | Pearson Correlation | -.024 | -.159 | 1 | -.107 | .111 |
| | Sig. (2-tailed) | .886 | .341 | | .522 | .524 |
| | N | 38 | 38 | 38 | 38 | 35 |
| PHA diameters ratio after/before | Pearson Correlation | .082 | .152 | -.107 | 1 | .403 |
| | Sig. (2-tailed) | .624 | .361 | .522 | | .016 |
| | N | 38 | 38 | 38 | 38 | 35 |
| RGEA diameters ratio after/before | Pearson Correlation | -.059 | -.073 | .111 | .403 | 1 |
| | Sig. (2-tailed) | .738 | .678 | .524 | .016 | |

| | | | | | |
|---|---|---|---|---|---|
| N | 35 | 35 | 35 | 35 | 35 |

Nonparametric Correlations

**Correlations**

| | | | Linear blood flow velosity decrease.% | GDA diameters ratio after/before | PDA diameters ratio after/before | PHA diameters ratio after/before | RGEA diameters ratio after/before |
|---|---|---|---|---|---|---|---|
| Spearman's rho | Linear blood flow velosity decrease.% | Correlation Coefficient | 1.000 | .085 | -.114 | .142 | -.063 |
| | | Sig. (2-tailed) | . | .611 | .496 | .397 | .719 |
| | | N | 38 | 38 | 38 | 38 | 35 |
| | GDA diameters ratio after/before | Correlation Coefficient | .085 | 1.000 | -.244 | .106 | -.059 |
| | | Sig. (2-tailed) | .611 | . | .140 | .527 | .737 |
| | | N | 38 | 38 | 38 | 38 | 35 |
| | PDA diameters ratio after/before | Correlation Coefficient | -.114 | -.244 | 1.000 | -.072 | .268 |
| | | Sig. (2-tailed) | .496 | .140 | . | .669 | .120 |
| | | N | 38 | 38 | 38 | 38 | 35 |
| | PHA diameters ratio after/before | Correlation Coefficient | .142 | .106 | -.072 | 1.000 | .198 |
| | | Sig. (2-tailed) | .397 | .527 | .669 | . | .254 |
| | | N | 38 | 38 | 38 | 38 | 35 |
| | RGEA diameters ratio after/before | Correlation Coefficient | -.063 | -.059 | .268 | .198 | 1.000 |
| | | Sig. (2-tailed) | .719 | .737 | .120 | .254 | . |
| | | N | 35 | 35 | 35 | 35 | 35 |

Graph

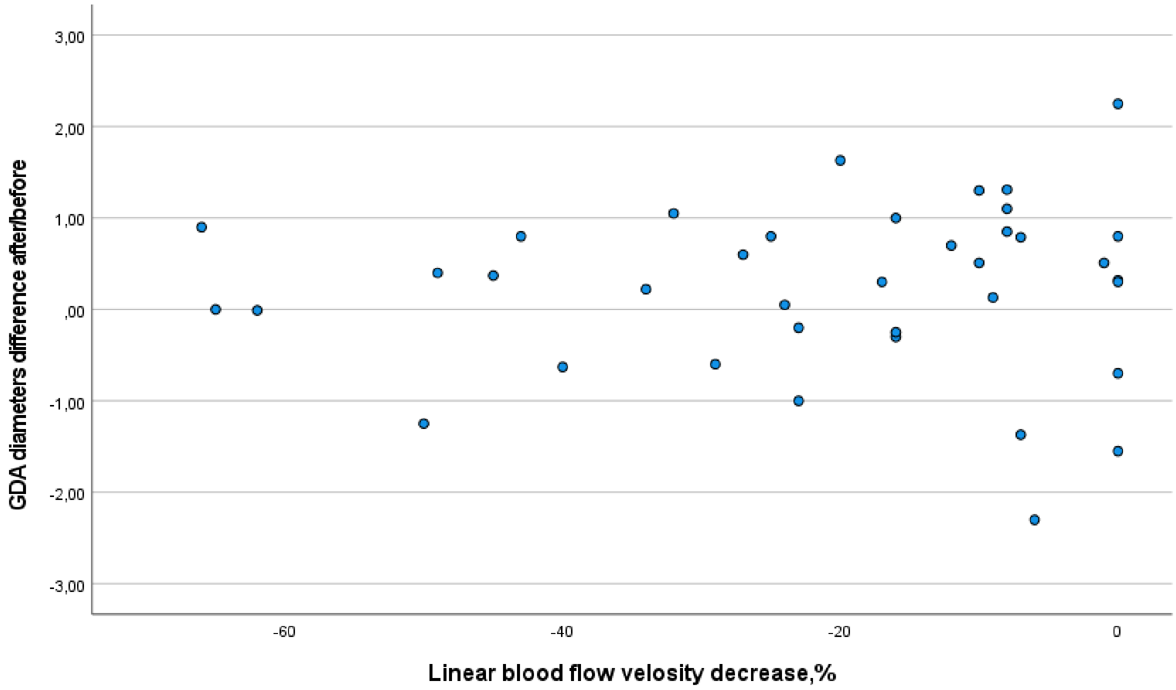
3,00
2,00
1,00
,00
-1,00
-2,00
-3,00
GDA diameters difference after/before
-60
-40
-20
0
Linear blood flow velosity decrease,%

Graph

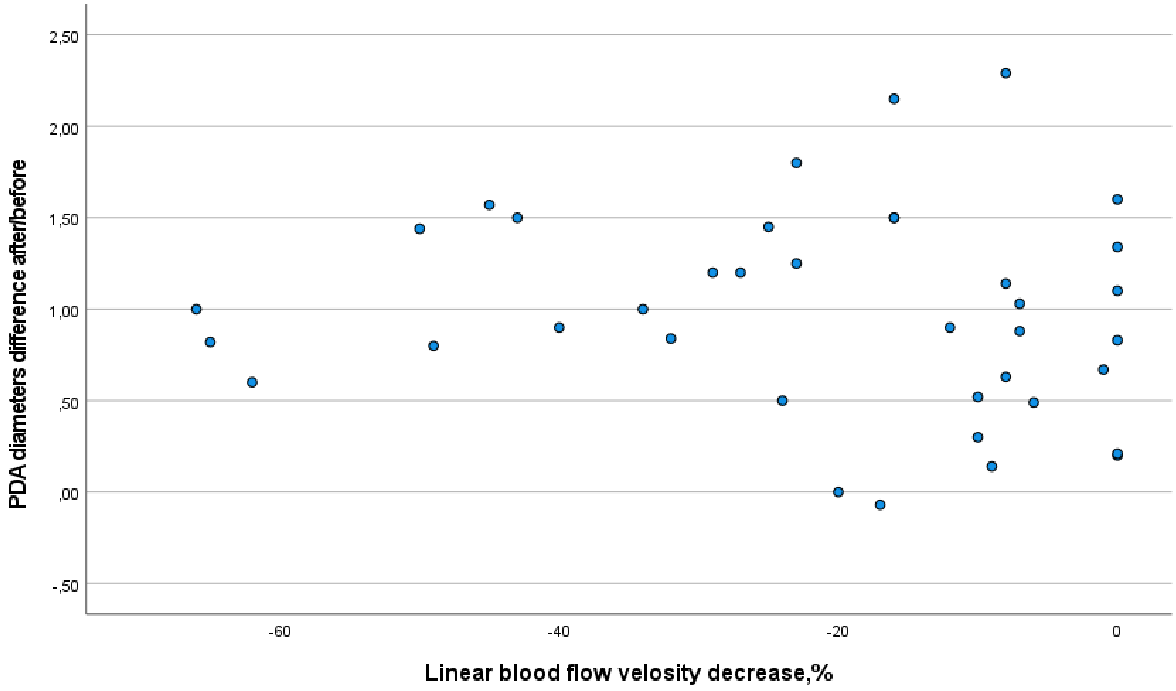
2,50
2,00
1,50
1,00
,50
,00
-,50
PDA diameters difference after/before
-60
-40
-20
0
Linear blood flow velosity decrease,%

Graph

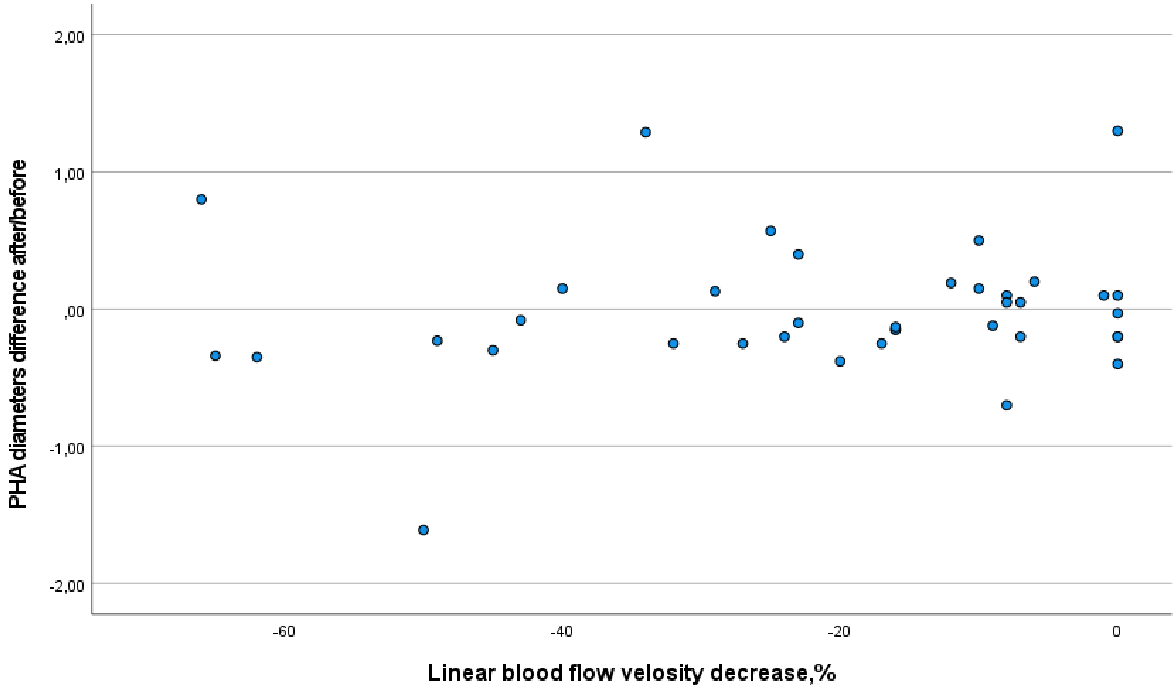


Graph

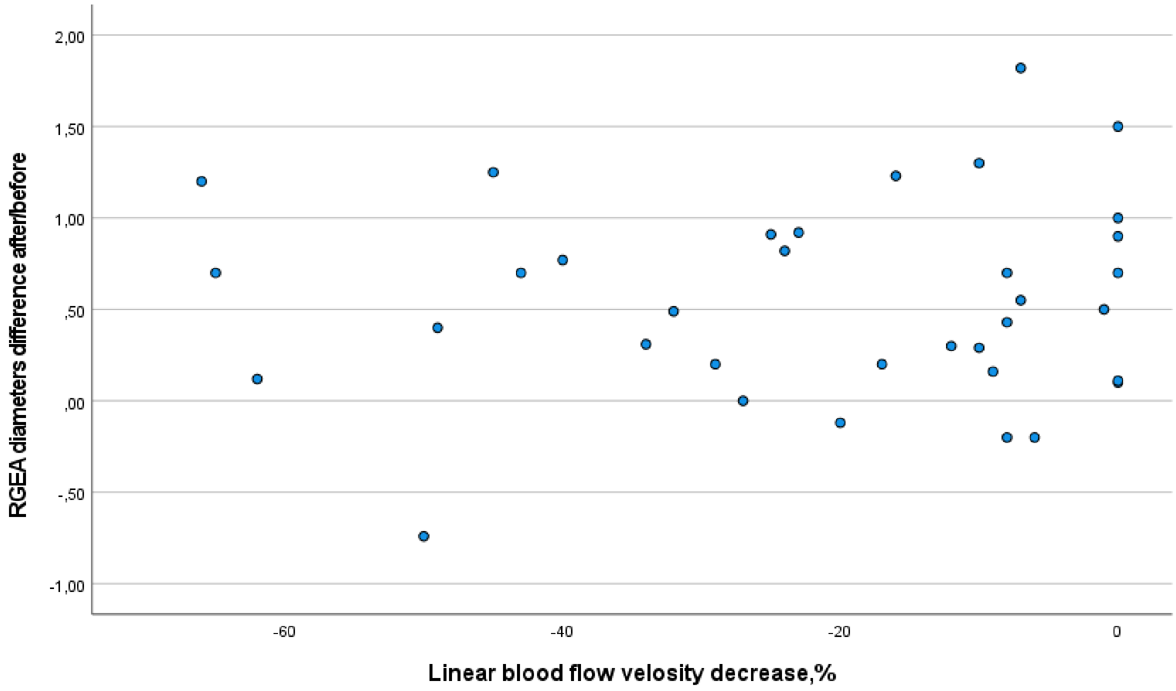


Correlations

**Correlations**

| | | Linear blood flow velosity decrease.% | GDA diameters difference after/before | PDA diameters difference after/before | PHA diameters difference after/before | RGEA diameters difference after/before |
|---|---|---|---|---|---|---|
| Linear blood flow velosity decrease.% | Pearson Correlation | 1 | .047 | -.112 | .136 | .063 |

| | | | | | | |
|---|---|---|---|---|---|---|
| | Sig. (2-tailed) | | .780 | .505 | .416 | .721 |
| | N | 38 | 38 | 38 | 38 | 35 |
| GDA diameters difference after/before | Pearson Correlation | .047 | 1 | -.187 | .176 | -.055 |
| | Sig. (2-tailed) | .780 | | .261 | .290 | .752 |
| | N | 38 | 38 | 38 | 38 | 35 |
| PDA diameters difference after/before | Pearson Correlation | -.112 | -.187 | 1 | -.126 | .241 |
| | Sig. (2-tailed) | .505 | .261 | | .450 | .163 |
| | N | 38 | 38 | 38 | 38 | 35 |
| PHA diameters difference after/before | Pearson Correlation | .136 | .176 | -.126 | 1 | .349 |
| | Sig. (2-tailed) | .416 | .290 | .450 | | .040 |
| | N | 38 | 38 | 38 | 38 | 35 |
| RGEA diameters difference after/before | Pearson Correlation | .063 | -.055 | .241 | .349 | 1 |
| | Sig. (2-tailed) | .721 | .752 | .163 | .040 | |
| | N | 35 | 35 | 35 | 35 | 35 |

Nonparametric Correlations

**Correlations**

| | | | Linear blood flow velosity decrease.% | GDA diameters difference after/before | PDA diameters difference after/before | PHA diameters difference after/before | RGEA diameters difference after/before |
|---|---|---|---|---|---|---|---|
| Spearman's rho | Linear blood flow velosity decrease.% | Correlation Coefficient | 1.000 | .060 | -.142 | .160 | .046 |
| | | Sig. (2-tailed) | . | .721 | .395 | .338 | .791 |
| | | N | 38 | 38 | 38 | 38 | 35 |
| | GDA diameters difference after/before | Correlation Coefficient | .060 | 1.000 | -.166 | .057 | -.095 |
| | | Sig. (2-tailed) | .721 | . | .318 | .732 | .587 |
| | | N | 38 | 38 | 38 | 38 | 35 |
| | PDA diameters difference after/before | Correlation Coefficient | -.142 | -.166 | 1.000 | -.002 | .255 |
| | | Sig. (2-tailed) | .395 | .318 | . | .990 | .139 |
| | | N | 38 | 38 | 38 | 38 | 35 |
| | PHA diameters difference after/before | Correlation Coefficient | .160 | .057 | -.002 | 1.000 | .235 |
| | | Sig. (2-tailed) | .338 | .732 | .990 | . | .174 |

| | | | | | | | |
|---|---|---|---|---|---|---|---|
| | | N | 38 | 38 | 38 | 38 | 35 |
| | RGEA diameters difference after/before | Correlation Coefficient | .046 | -.095 | .255 | .235 | 1.000 |
| | | Sig. (2-tailed) | .791 | .587 | .139 | .174 | . |
| | | N | 35 | 35 | 35 | 35 | 35 |

T-Test

**Group Statistics**

| | Puls preservation | N | Mean | Std. Deviation | Std. Error Mean |
|---|---|---|---|---|---|
| GDA blood flow intencity alteration after/before | no | 4 | 1.5601 | .99783 | .49891 |
| | yes | 34 | 2.4687 | 4.29446 | .73649 |
| PDA blood flow intencity alteration after/before | no | 4 | 5.3010 | 3.03835 | 1.51918 |
| | yes | 34 | 7.5634 | 15.33233 | 2.62947 |
| PHA blood flow intencity alteration after/before | no | 4 | 1.1916 | .73733 | .36866 |
| | yes | 34 | 1.1842 | .78858 | .13524 |
| RGEA blood flow intencity alteration after/before | no | 4 | 4.0041 | 4.42891 | 2.21446 |
| | yes | 31 | 3.4930 | 4.02407 | .72274 |
| GDA diameters ratio after/before | no | 4 | 1.0914 | .15515 | .07757 |
| | yes | 34 | 1.1106 | .31842 | .05461 |
| PDA diameters ratio after/before | no | 4 | 1.4821 | .21718 | .10859 |
| | yes | 34 | 1.4635 | .40070 | .06872 |
| PHA diameters ratio after/before | no | 4 | 1.0333 | .24347 | .12173 |
| | yes | 34 | .9964 | .11895 | .02040 |
| RGEA diameters ratio after/before | no | 4 | 1.3042 | .35815 | .17907 |
| | yes | 31 | 1.2644 | .29022 | .05212 |
| GDA diameters difference after/before | no | 4 | .3025 | .42680 | .21340 |
| | yes | 34 | .2241 | .97675 | .16751 |
| PDA diameters difference after/before | no | 4 | 1.0050 | .42907 | .21453 |
| | yes | 34 | .9765 | .58052 | .09956 |
| PHA diameters difference after/before | no | 4 | .0525 | .54070 | .27035 |
| | yes | 34 | -.0221 | .49866 | .08552 |
| RGEA diameters difference after/before | no | 4 | .5300 | .52624 | .26312 |
| | yes | 31 | .5548 | .55292 | .09931 |

**Independent Samples Test**

| | Levene's Test for Equality of Variances | | t-test for Equality of Means | | | | | |
|---|---|---|---|---|---|---|---|---|
| | F | Sig. | t | df | Sig. (2-tailed) | Mean Difference | Std. Error | 95% Confidence Interval of the Difference |

| | | | | | | | | Difference | Lower | Upper |
|---|---|---|---|---|---|---|---|---|---|---|
| GDA blood flow intencity alteration after/before | Equal variances assumed | .414 | .524 | -.417 | 36 | .679 | -.90868 | 2.17871 | -5.32731 | 3.50996 |
| | Equal variances not assumed | | | -1.021 | 21.178 | .319 | -.90868 | .88957 | -2.75770 | .94034 |
| PDA blood flow intencity alteration after/before | Equal variances assumed | .382 | .540 | -.291 | 36 | .773 | -2.26241 | 7.77338 | -18.02756 | 13.50275 |
| | Equal variances not assumed | | | -.745 | 26.378 | .463 | -2.26241 | 3.03678 | -8.50024 | 3.97543 |
| PHA blood flow intencity alteration after/before | Equal variances assumed | .338 | .565 | .018 | 36 | .986 | .00746 | .41465 | -.83348 | .84841 |
| | Equal variances not assumed | | | .019 | 3.855 | .986 | .00746 | .39269 | -1.09912 | 1.11405 |
| RGEA blood flow intencity alteration after/before | Equal variances assumed | .210 | .650 | .237 | 33 | .814 | .51109 | 2.15835 | -3.88010 | 4.90228 |
| | Equal variances not assumed | | | .219 | 3.669 | .838 | .51109 | 2.32942 | -6.19314 | 7.21532 |
| GDA diameters ratio after/before | Equal variances assumed | .918 | .345 | -.118 | 36 | .907 | -.01922 | .16288 | -.34956 | .31111 |
| | Equal variances not assumed | | | -.203 | 6.563 | .846 | -.01922 | .09487 | -.24661 | .20816 |
| PDA diameters ratio after/before | Equal variances assumed | .390 | .536 | .090 | 36 | .929 | .01854 | .20548 | -.39819 | .43528 |
| | Equal variances not assumed | | | .144 | 5.800 | .890 | .01854 | .12851 | -.29855 | .33564 |
| PHA diameters | Equal variances assumed | 3.679 | .063 | .522 | 36 | .605 | .03692 | .07074 | -.10655 | .18038 |

| | | | | | | | | | | |
|---|---|---|---|---|---|---|---|---|---|---|
| ratio after/before | Equal variances not assumed | | | .299 | 3.171 | .783 | .03692 | .12343 | -.34420 | .41804 |
| RGEA diameters ratio after/before | Equal variances assumed | .248 | .622 | .253 | 33 | .802 | .03986 | .15781 | -.28120 | .36093 |
| | Equal variances not assumed | | | .214 | 3.527 | .843 | .03986 | .18651 | -.50651 | .58623 |
| GDA diameters difference after/before | Equal variances assumed | 2.160 | .150 | .157 | 36 | .876 | .07838 | .49859 | -.93281 | 1.08958 |
| | Equal variances not assumed | | | .289 | 7.575 | .780 | .07838 | .27129 | -.55339 | .71015 |
| PDA diameters difference after/before | Equal variances assumed | .864 | .359 | .095 | 36 | .925 | .02853 | .30100 | -.58193 | .63899 |
| | Equal variances not assumed | | | .121 | 4.413 | .909 | .02853 | .23651 | -.60460 | .66166 |
| PHA diameters difference after/before | Equal variances assumed | .146 | .704 | .281 | 36 | .780 | .07456 | .26551 | -.46393 | .61305 |
| | Equal variances not assumed | | | .263 | 3.627 | .807 | .07456 | .28355 | -.74568 | .89480 |
| RGEA diameters difference after/before | Equal variances assumed | .008 | .928 | -.085 | 33 | .933 | -.02484 | .29250 | -.61993 | .57025 |
| | Equal variances not assumed | | | -.088 | 3.908 | .934 | -.02484 | .28124 | -.81302 | .76334 |

NPar Tests
Mann-Whitney Test

**Ranks**

| | Puls preservation | N | Mean Rank | Sum of Ranks |
|---|---|---|---|---|
| GDA blood flow intencity alteration after/before | no | 4 | 18.50 | 74.00 |
| | yes | 34 | 19.62 | 667.00 |
| | Total | 38 | | |
| PDA blood flow intencity alteration after/before | no | 4 | 22.00 | 88.00 |
| | yes | 34 | 19.21 | 653.00 |

| | | | | |
|---|---|---|---|---|
| | Total | 38 | | |
| PHA blood flow intencity alteration after/before | no | 4 | 21.00 | 84.00 |
| | yes | 34 | 19.32 | 657.00 |
| | Total | 38 | | |
| RGEA blood flow intencity alteration after/before | no | 4 | 18.00 | 72.00 |
| | yes | 31 | 18.00 | 558.00 |
| | Total | 35 | | |
| GDA diameters ratio after/before | no | 4 | 18.50 | 74.00 |
| | yes | 34 | 19.62 | 667.00 |
| | Total | 38 | | |
| PDA diameters ratio after/before | no | 4 | 22.00 | 88.00 |
| | yes | 34 | 19.21 | 653.00 |
| | Total | 38 | | |
| PHA diameters ratio after/before | no | 4 | 18.00 | 72.00 |
| | yes | 34 | 19.68 | 669.00 |
| | Total | 38 | | |
| RGEA diameters ratio after/before | no | 4 | 18.00 | 72.00 |
| | yes | 31 | 18.00 | 558.00 |
| | Total | 35 | | |
| GDA diameters difference after/before | no | 4 | 18.75 | 75.00 |
| | yes | 34 | 19.59 | 666.00 |
| | Total | 38 | | |
| PDA diameters difference after/before | no | 4 | 19.88 | 79.50 |
| | yes | 34 | 19.46 | 661.50 |
| | Total | 38 | | |
| PHA diameters difference after/before | no | 4 | 18.25 | 73.00 |
| | yes | 34 | 19.65 | 668.00 |
| | Total | 38 | | |
| RGEA diameters difference after/before | no | 4 | 16.38 | 65.50 |
| | yes | 31 | 18.21 | 564.50 |
| | Total | 35 | | |

**Test Statistics**

| | Mann-Whitney U | Wilcoxon W | Z | Asymp. Sig. (2-tailed) | Exact Sig. [2*(1-tailed Sig.)] |
|---|---|---|---|---|---|
| GDA blood flow intencity alteration after/before | 64.000 | 74.000 | -.190 | .849 | .873 |
| PDA blood flow intencity alteration after/before | 58.000 | 653.000 | -.476 | .634 | .663 |
| PHA blood flow intencity alteration after/before | 62.000 | 657.000 | -.285 | .775 | .801 |

| | | | | | |
|---|---|---|---|---|---|
| RGEA blood flow intencity alteration after/before | 62.000 | 558.000 | .000 | 1.000 | 1.000 |
| GDA diameters ratio after/before | 64.000 | 74.000 | -.190 | .849 | .873 |
| PDA diameters ratio after/before | 58.000 | 653.000 | -.476 | .634 | .663 |
| PHA diameters ratio after/before | 62.000 | 72.000 | -.285 | .775 | .801 |
| RGEA diameters ratio after/before | 62.000 | 558.000 | .000 | 1.000 | 1.000 |
| GDA diameters difference after/before | 65.000 | 75.000 | -.143 | .886 | .909 |
| PDA diameters difference after/before | 66.500 | 661.500 | -.071 | .943 | .945 |
| PHA diameters difference after/before | 63.000 | 73.000 | -.238 | .812 | .836 |
| RGEA diameters difference after/before | 55.500 | 65.500 | -.337 | .736 | .745 |

Graph

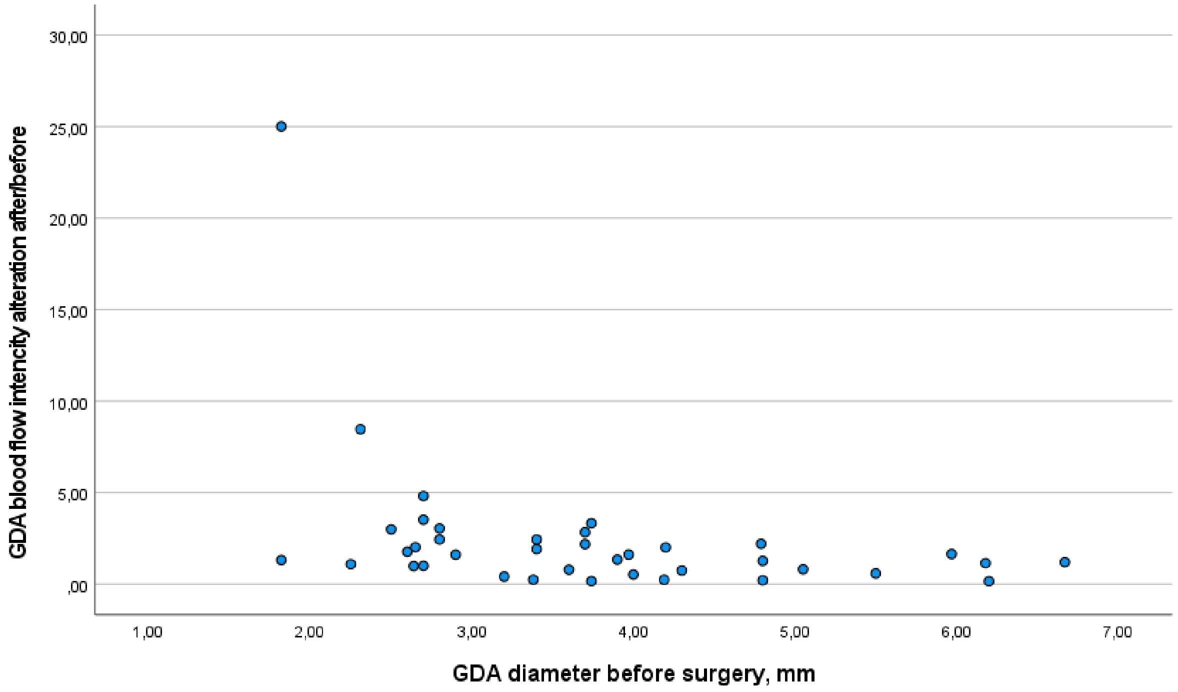


Graph

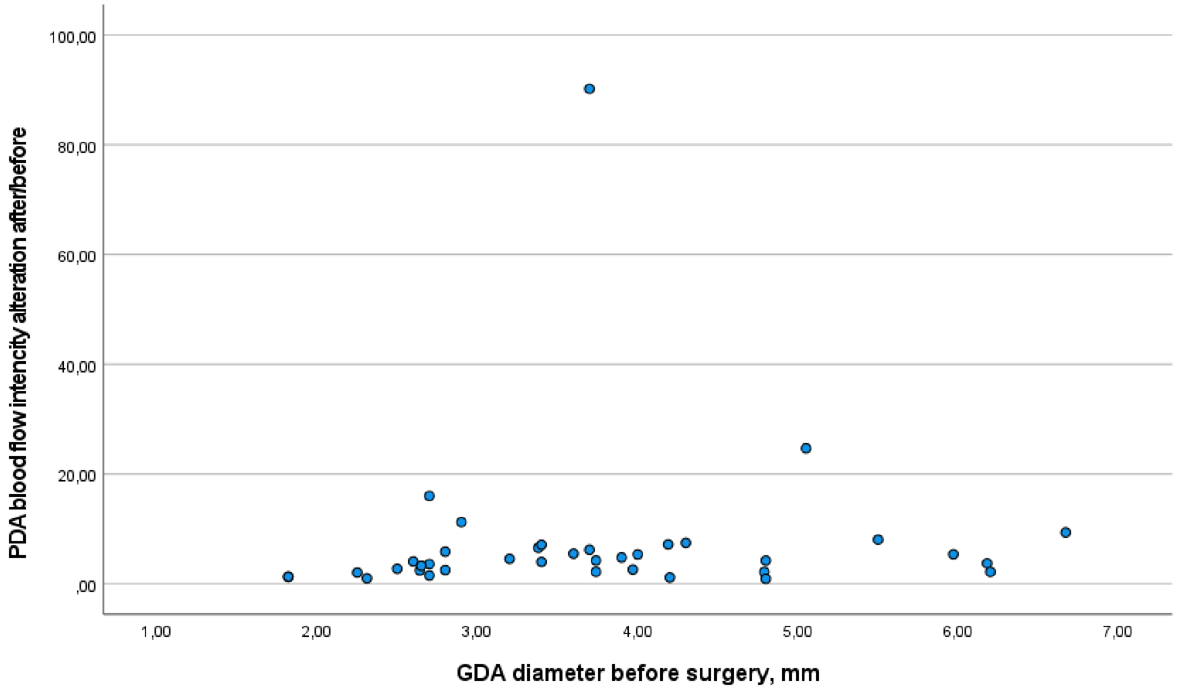
100,00
80,00
60,00
40,00
20,00
,00
PDA blood flow intencity alteration after/before
1,00
2,00
3,00
4,00
5,00
6,00
7,00
GDA diameter before surgery, mm

Graph

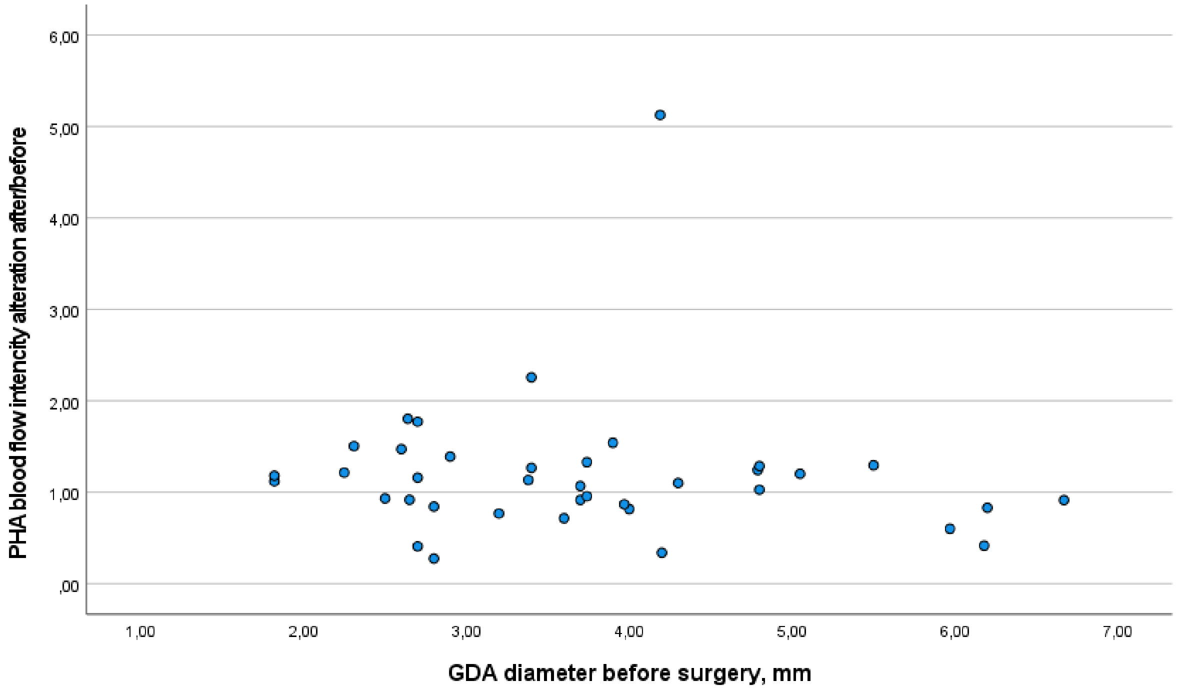
6,00
5,00
4,00
3,00
2,00
1,00
,00
PHA blood flow intencity alteration after/before
1,00
2,00
3,00
4,00
5,00
6,00
7,00
GDA diameter before surgery, mm

Graph

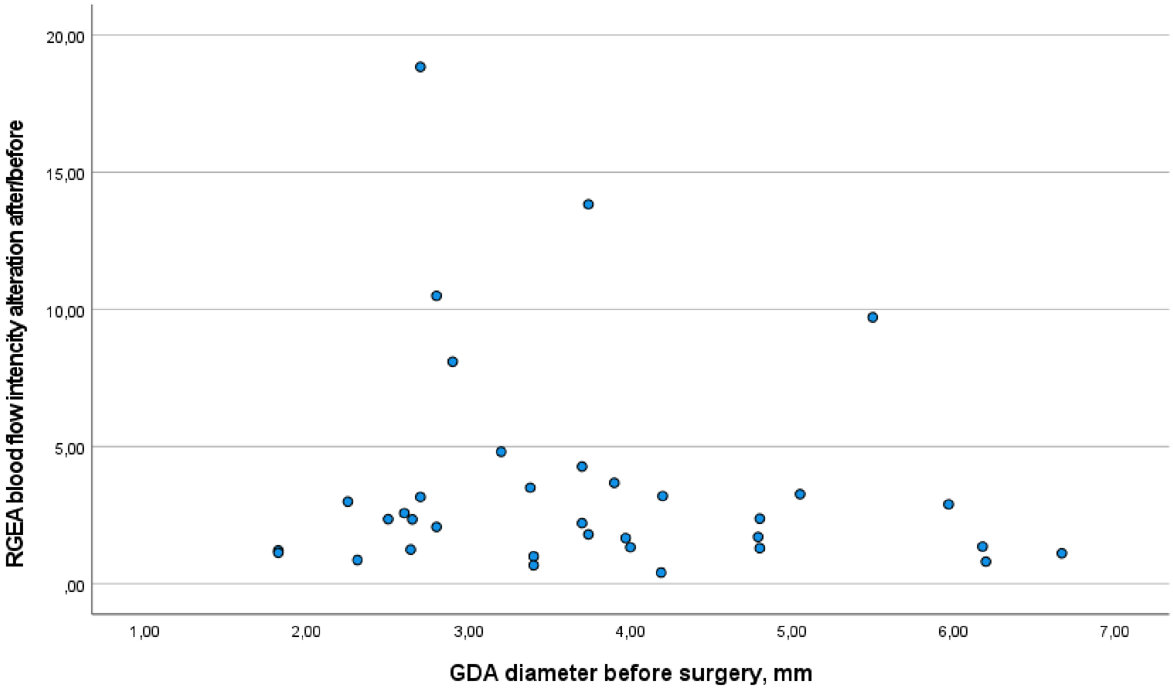


Correlations

**Correlations**

| | | GDA diameter before surgery. mm | GDA blood flow intencity alteration after/before | PDA blood flow intencity alteration after/before | PHA blood flow intencity alteration after/before | RGEA blood flow intencity alteration after/before |
|---|---|---|---|---|---|---|
| GDA diameter before surgery. mm | Pearson Correlation | 1 | -.381 | .069 | -.084 | -.122 |
| | Sig. (2-tailed) | | .018 | .679 | .616 | .486 |
| | N | 38 | 38 | 38 | 38 | 35 |
| GDA blood flow intencity alteration after/before | Pearson Correlation | -.381 | 1 | -.042 | -.057 | -.057 |
| | Sig. (2-tailed) | .018 | | .800 | .735 | .744 |
| | N | 38 | 38 | 38 | 38 | 35 |
| PDA blood flow intencity alteration after/before | Pearson Correlation | .069 | -.042 | 1 | -.015 | -.033 |
| | Sig. (2-tailed) | .679 | .800 | | .926 | .851 |
| | N | 38 | 38 | 38 | 38 | 35 |
| PHA blood flow intencity alteration after/before | Pearson Correlation | -.084 | -.057 | -.015 | 1 | -.246 |
| | Sig. (2-tailed) | .616 | .735 | .926 | | .155 |
| | N | 38 | 38 | 38 | 38 | 35 |
| | Pearson Correlation | -.122 | -.057 | -.033 | -.246 | 1 |

| | | | | | | |
|---|---|---|---|---|---|---|
| RGEA blood flow intencity alteration after/before | Sig. (2-tailed) | .486 | .744 | .851 | .155 | |
| | N | 35 | 35 | 35 | 35 | 35 |

Nonparametric Correlations

**Correlations**

| | | | GDA diameter before surgery. mm | GDA blood flow intencity alteration after/before | PDA blood flow intencity alteration after/before | PHA blood flow intencity alteration after/before | RGEA blood flow intencity alteration after/before |
|---|---|---|---|---|---|---|---|
| Spearman's rho | GDA diameter before surgery. mm | Correlation Coefficient | 1.000 | -.460 | .310 | -.222 | -.059 |
| | | Sig. (2-tailed) | . | .004 | .058 | .180 | .736 |
| | | N | 38 | 38 | 38 | 38 | 35 |
| | GDA blood flow intencity alteration after/before | Correlation Coefficient | -.460 | 1.000 | -.246 | -.109 | -.057 |
| | | Sig. (2-tailed) | .004 | . | .137 | .515 | .746 |
| | | N | 38 | 38 | 38 | 38 | 35 |
| | PDA blood flow intencity alteration after/before | Correlation Coefficient | .310 | -.246 | 1.000 | .072 | .268 |
| | | Sig. (2-tailed) | .058 | .137 | . | .669 | .120 |
| | | N | 38 | 38 | 38 | 38 | 35 |
| | PHA blood flow intencity alteration after/before | Correlation Coefficient | -.222 | -.109 | .072 | 1.000 | -.198 |
| | | Sig. (2-tailed) | .180 | .515 | .669 | . | .254 |
| | | N | 38 | 38 | 38 | 38 | 35 |
| | RGEA blood flow intencity alteration after/before | Correlation Coefficient | -.059 | -.057 | .268 | -.198 | 1.000 |
| | | Sig. (2-tailed) | .736 | .746 | .120 | .254 | . |
| | | N | 35 | 35 | 35 | 35 | 35 |

Graph

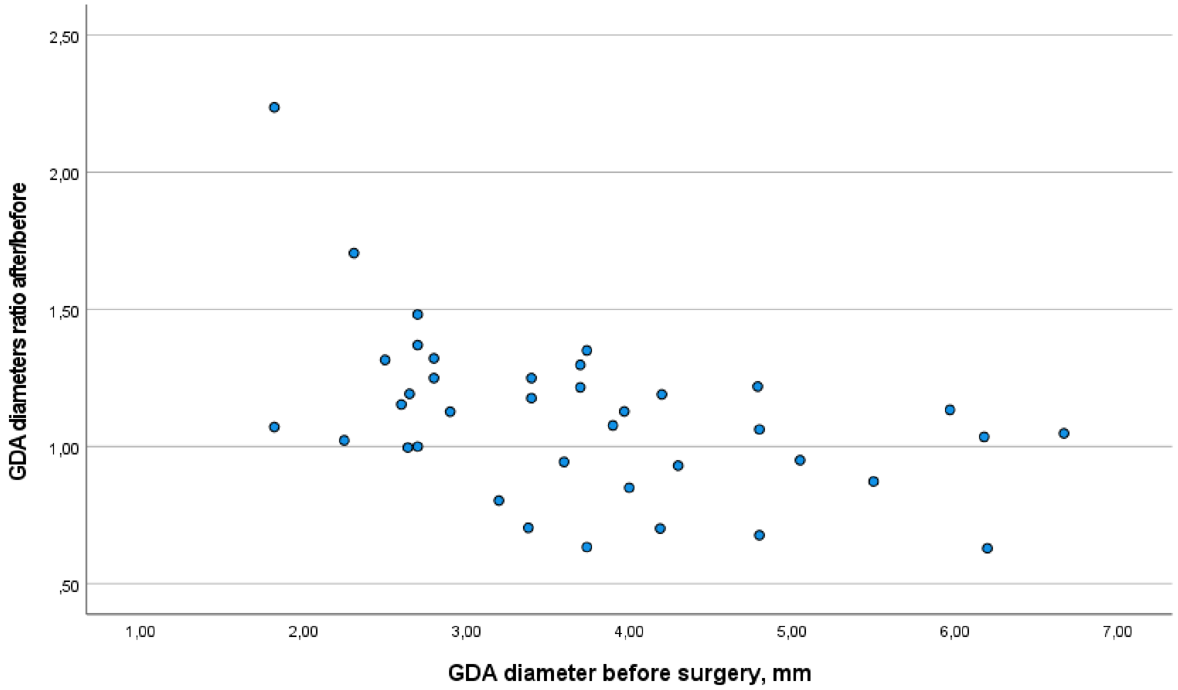
2,50
2,00
1,50
1,00
,50
GDA diameters ratio after/before
1,00
2,00
3,00
4,00
5,00
6,00
7,00
GDA diameter before surgery, mm

Graph

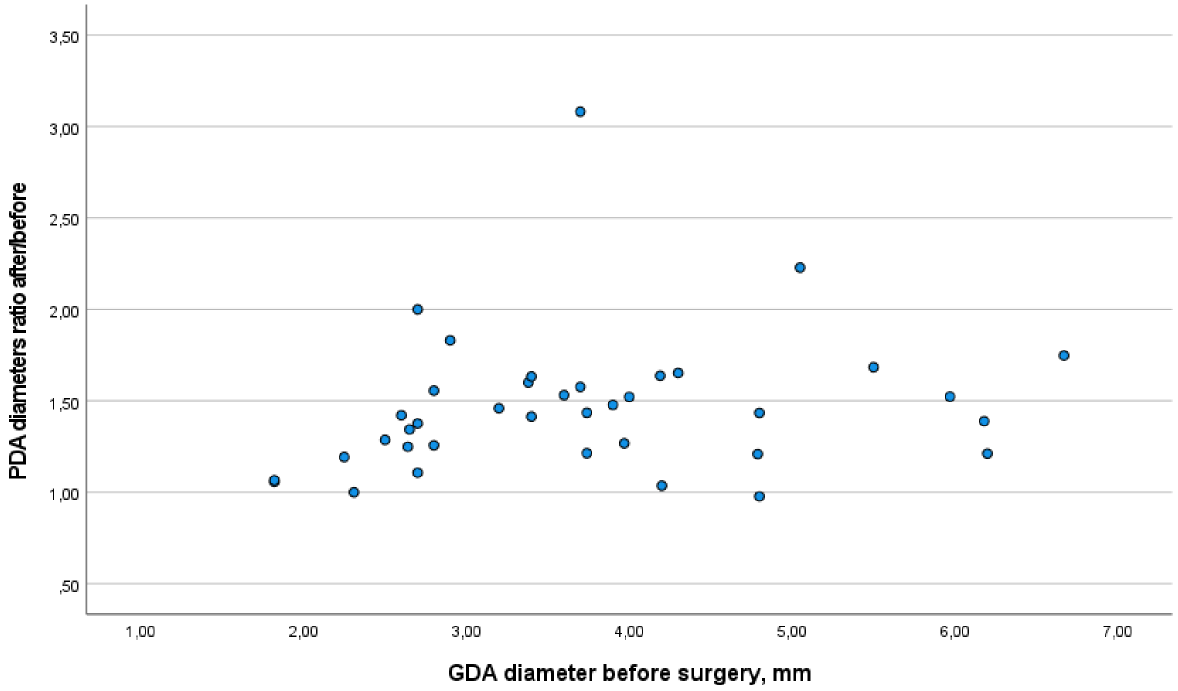
3,50
3,00
2,50
2,00
1,50
1,00
,50
PDA diameters ratio after/before
1,00
2,00
3,00
4,00
5,00
6,00
7,00
GDA diameter before surgery, mm

Graph

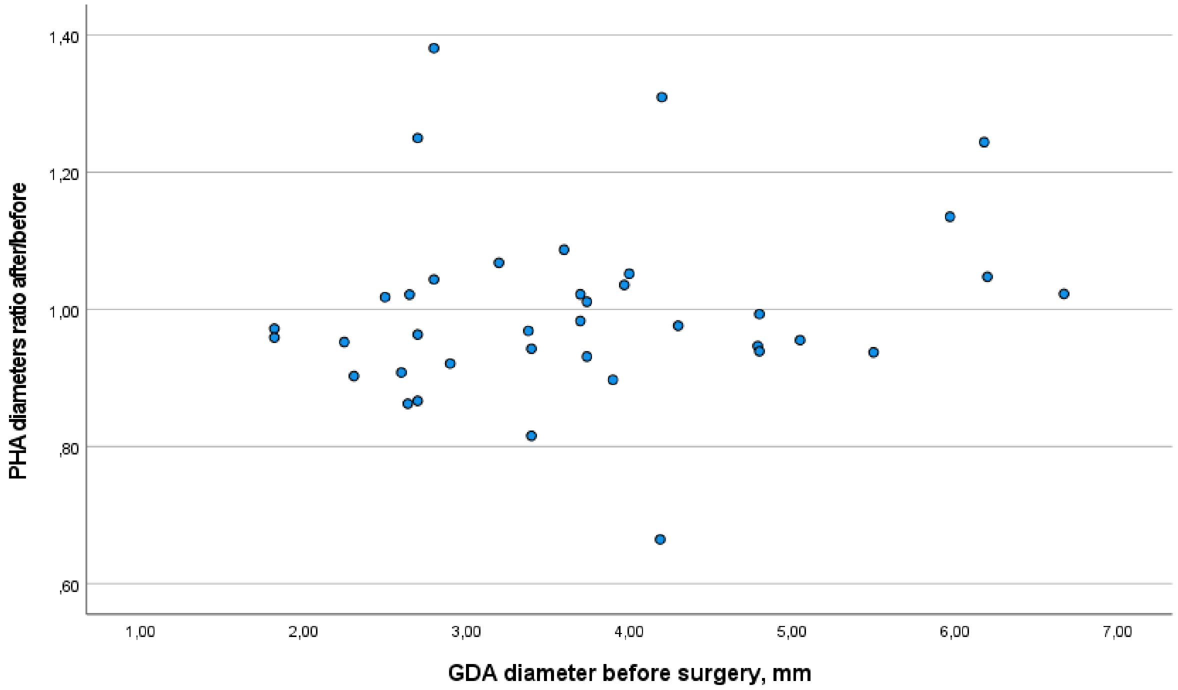


Graph

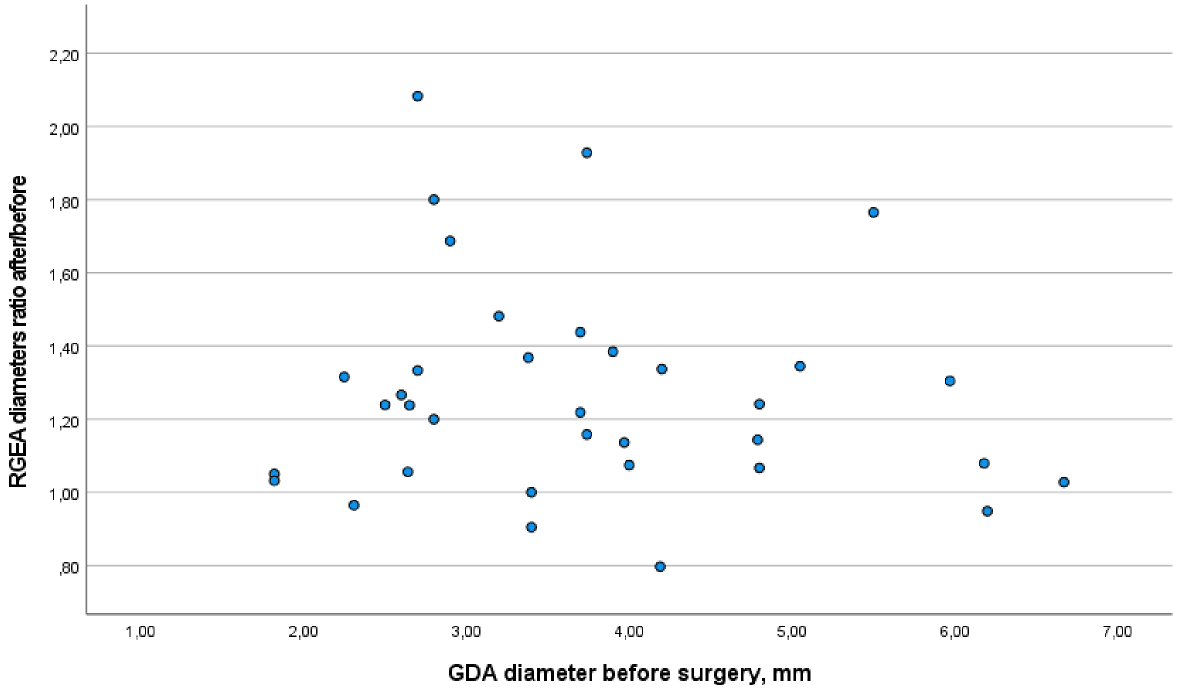


Correlations

**Correlations**

| | GDA diameter before surgery. mm | GDA diameters ratio after/before | PDA diameters ratio after/before | PHA diameters ratio after/before | RGEA diameters ratio after/before |
|---|---|---|---|---|---|

| | | | | | | |
|---|---|---|---|---|---|---|
| GDA diameter before surgery. mm | Pearson Correlation | 1 | -.465 | .196 | .154 | -.126 |
| | Sig. (2-tailed) | | .003 | .237 | .354 | .471 |
| | N | 38 | 38 | 38 | 38 | 35 |
| GDA diameters ratio after/before | Pearson Correlation | -.465 | 1 | -.159 | .152 | -.073 |
| | Sig. (2-tailed) | .003 | | .341 | .361 | .678 |
| | N | 38 | 38 | 38 | 38 | 35 |
| PDA diameters ratio after/before | Pearson Correlation | .196 | -.159 | 1 | -.107 | .111 |
| | Sig. (2-tailed) | .237 | .341 | | .522 | .524 |
| | N | 38 | 38 | 38 | 38 | 35 |
| PHA diameters ratio after/before | Pearson Correlation | .154 | .152 | -.107 | 1 | .403 |
| | Sig. (2-tailed) | .354 | .361 | .522 | | .016 |
| | N | 38 | 38 | 38 | 38 | 35 |
| RGEA diameters ratio after/before | Pearson Correlation | -.126 | -.073 | .111 | .403 | 1 |
| | Sig. (2-tailed) | .471 | .678 | .524 | .016 | |
| | N | 35 | 35 | 35 | 35 | 35 |

Nonparametric Correlations

**Correlations**

| | | | GDA diameter before surgery. mm | GDA diameters ratio after/before | PDA diameters ratio after/before | PHA diameters ratio after/before | RGEA diameters ratio after/before |
|---|---|---|---|---|---|---|---|
| Spearman's rho | GDA diameter before surgery. mm | Correlation Coefficient | 1.000 | -.459 | .310 | .222 | -.059 |
| | | Sig. (2-tailed) | . | .004 | .058 | .180 | .736 |
| | | N | 38 | 38 | 38 | 38 | 35 |
| | GDA diameters ratio after/before | Correlation Coefficient | -.459 | 1.000 | -.244 | .106 | -.059 |
| | | Sig. (2-tailed) | .004 | . | .140 | .527 | .737 |
| | | N | 38 | 38 | 38 | 38 | 35 |
| | PDA diameters ratio after/before | Correlation Coefficient | .310 | -.244 | 1.000 | -.072 | .268 |
| | | Sig. (2-tailed) | .058 | .140 | . | .669 | .120 |
| | | N | 38 | 38 | 38 | 38 | 35 |
| | PHA diameters ratio after/before | Correlation Coefficient | .222 | .106 | -.072 | 1.000 | .198 |

| | | | | | | |
|---|---|---|---|---|---|---|
| | Sig. (2-tailed) | .180 | .527 | .669 | . | .254 |
| | N | 38 | 38 | 38 | 38 | 35 |
| RGEA diameters ratio after/before | Correlation Coefficient | -.059 | -.059 | .268 | .198 | 1.000 |
| | Sig. (2-tailed) | .736 | .737 | .120 | .254 | . |
| | N | 35 | 35 | 35 | 35 | 35 |

Graph

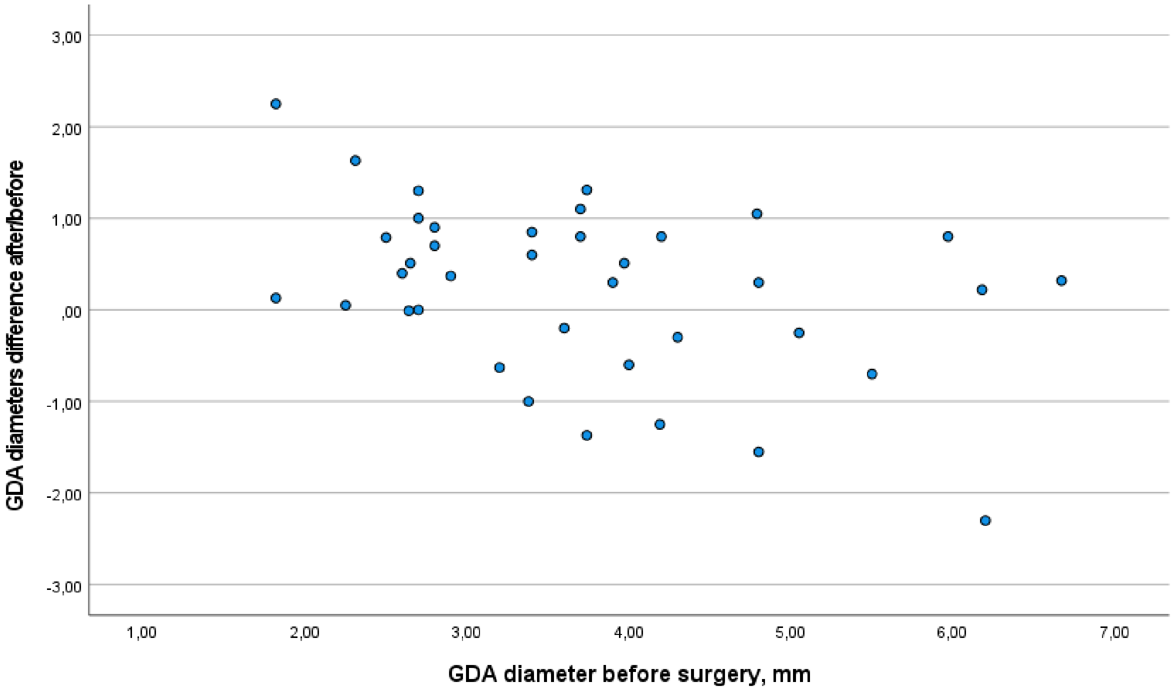


Graph

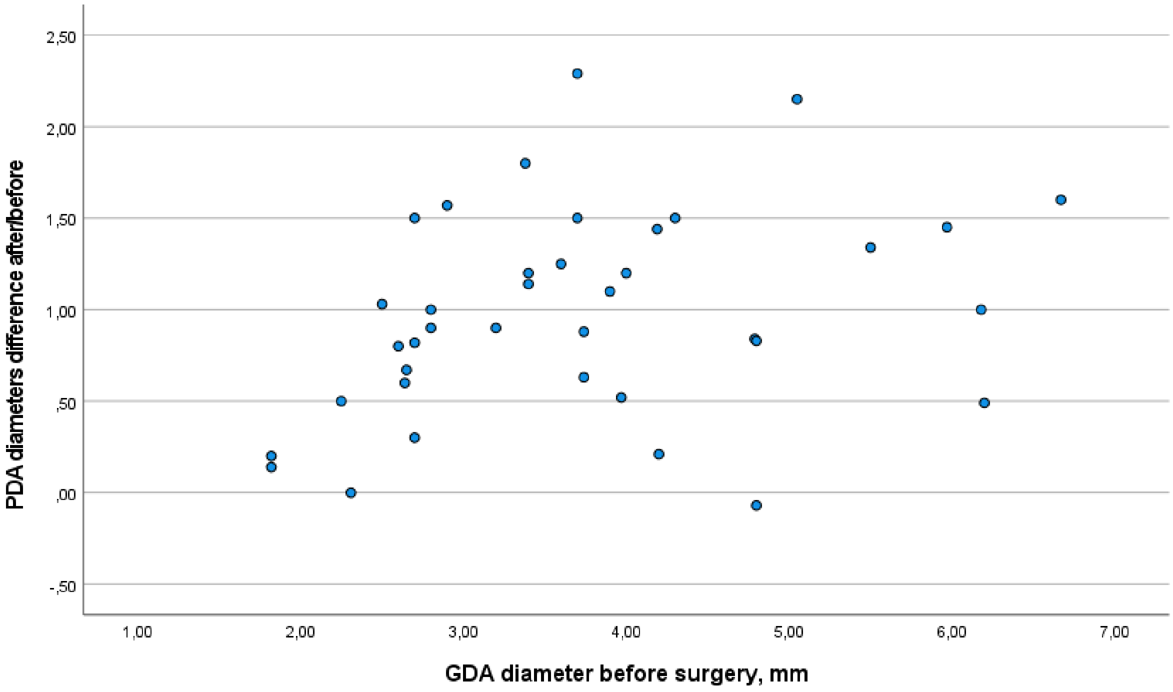

Graph

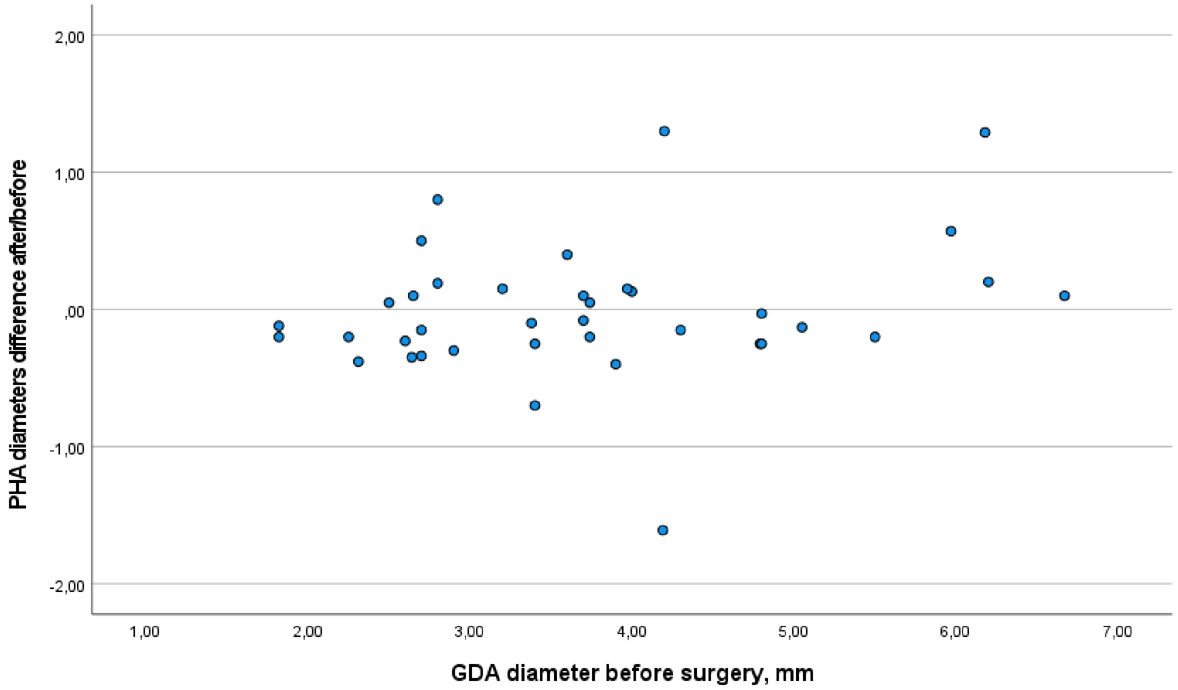


Graph

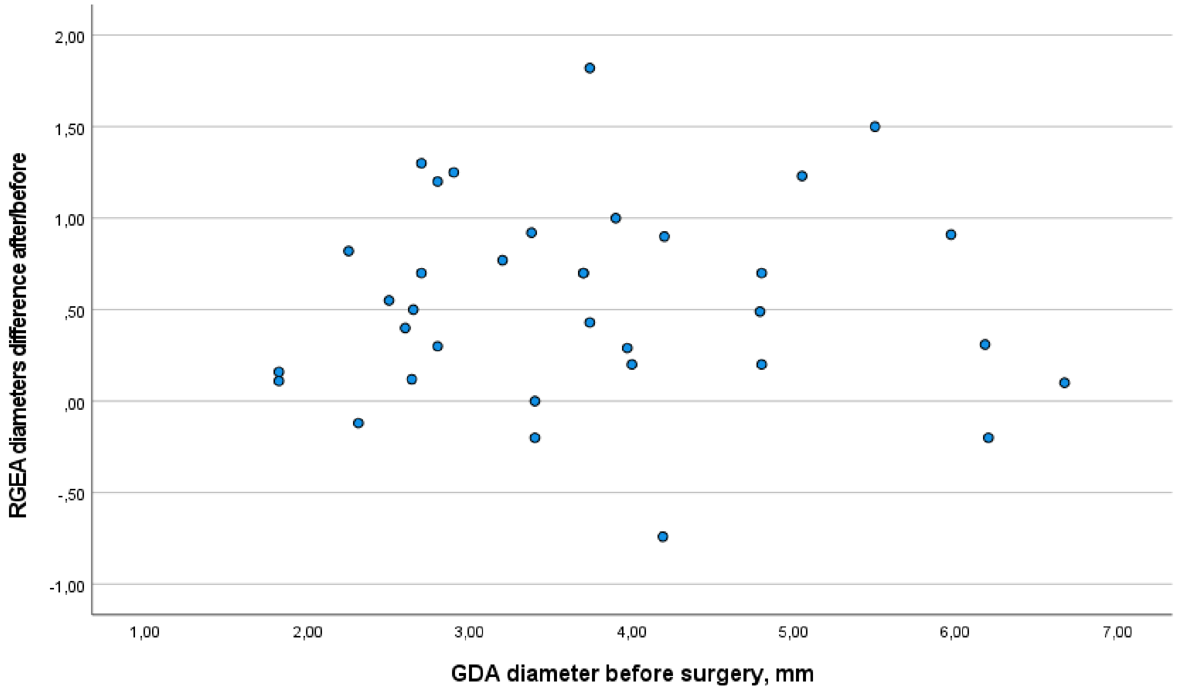


Correlations

**Correlations**

| | GDA diameter before surgery. mm | GDA diameters difference after/before | PDA diameters difference after/before | PHA diameters difference after/before | RGEA diameters difference after/before |
|---|---|---|---|---|---|

| | | | | | | |
|---|---|---|---|---|---|---|
| GDA diameter before surgery. mm | Pearson Correlation | 1 | -.404 | .312 | .234 | -.001 |
| | Sig. (2-tailed) | | .012 | .057 | .157 | .994 |
| | N | 38 | 38 | 38 | 38 | 35 |
| GDA diameters difference after/before | Pearson Correlation | -.404 | 1 | -.187 | .176 | -.055 |
| | Sig. (2-tailed) | .012 | | .261 | .290 | .752 |
| | N | 38 | 38 | 38 | 38 | 35 |
| PDA diameters difference after/before | Pearson Correlation | .312 | -.187 | 1 | -.126 | .241 |
| | Sig. (2-tailed) | .057 | .261 | | .450 | .163 |
| | N | 38 | 38 | 38 | 38 | 35 |
| PHA diameters difference after/before | Pearson Correlation | .234 | .176 | -.126 | 1 | .349 |
| | Sig. (2-tailed) | .157 | .290 | .450 | | .040 |
| | N | 38 | 38 | 38 | 38 | 35 |
| RGEA diameters difference after/before | Pearson Correlation | -.001 | -.055 | .241 | .349 | 1 |
| | Sig. (2-tailed) | .994 | .752 | .163 | .040 | |
| | N | 35 | 35 | 35 | 35 | 35 |

Nonparametric Correlations

**Correlations**

| | | | GDA diameter before surgery. mm | GDA diameters difference after/before | PDA diameters difference after/before | PHA diameters difference after/before | RGEA diameters difference after/before |
|---|---|---|---|---|---|---|---|
| Spearman's rho | GDA diameter before surgery. mm | Correlation Coefficient | 1.000 | -.350 | .343 | .237 | .051 |
| | | Sig. (2-tailed) | . | .031 | .035 | .151 | .770 |
| | | N | 38 | 38 | 38 | 38 | 35 |
| | GDA diameters difference after/before | Correlation Coefficient | -.350 | 1.000 | -.166 | .057 | -.095 |
| | | Sig. (2-tailed) | .031 | . | .318 | .732 | .587 |
| | | N | 38 | 38 | 38 | 38 | 35 |
| | PDA diameters difference after/before | Correlation Coefficient | .343 | -.166 | 1.000 | -.002 | .255 |
| | | Sig. (2-tailed) | .035 | .318 | . | .990 | .139 |
| | | N | 38 | 38 | 38 | 38 | 35 |
| | | Correlation Coefficient | .237 | .057 | -.002 | 1.000 | .235 |

| | | | | | | | |
|---|---|---|---|---|---|---|---|
| | PHA diameters difference after/before | Sig. (2-tailed) | .151 | .732 | .990 | . | .174 |
| | | N | 38 | 38 | 38 | 38 | 35 |
| | RGEA diameters difference after/before | Correlation Coefficient | .051 | -.095 | .255 | .235 | 1.000 |
| | | Sig. (2-tailed) | .770 | .587 | .139 | .174 | . |
| | | N | 35 | 35 | 35 | 35 | 35 |

Graph

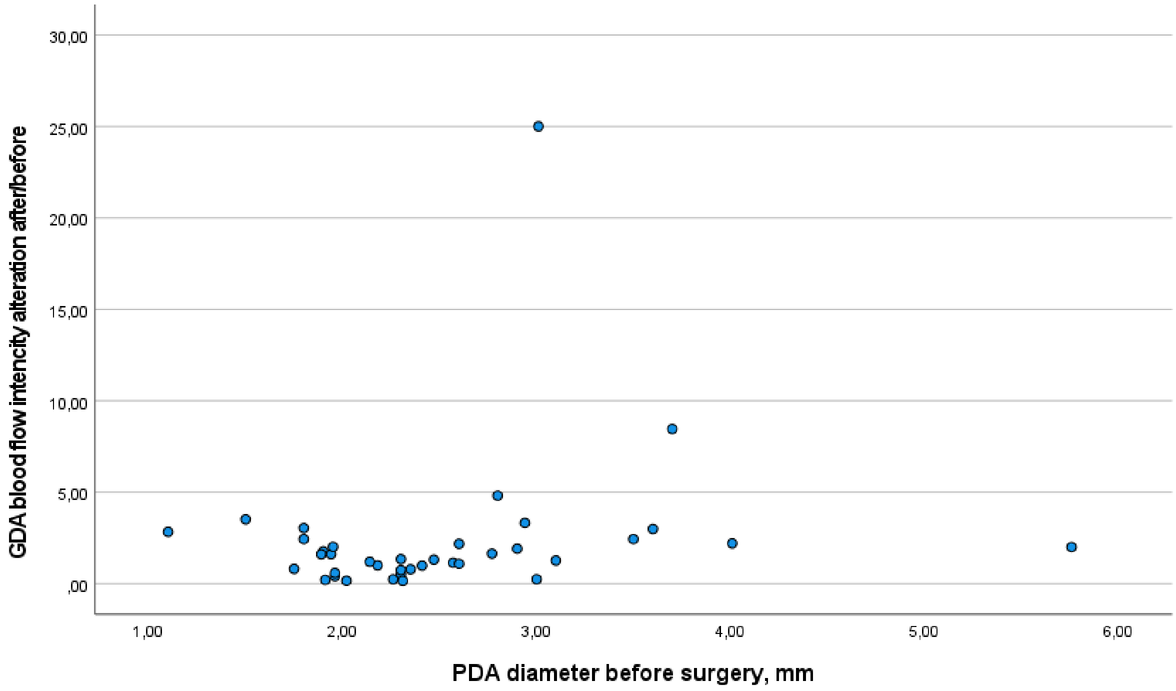


Graph

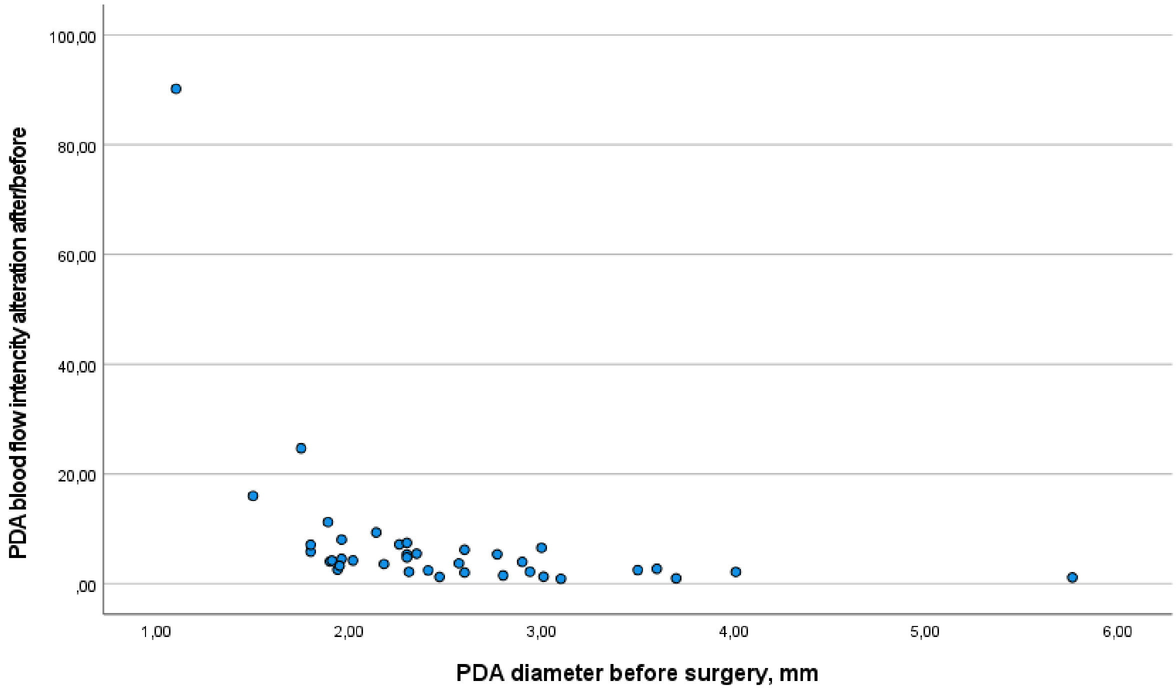
100,00
80,00
60,00
40,00
20,00
,00
1,00
2,00
3,00
4,00
5,00
6,00
PDA blood flow intencity alteration after/before
PDA diameter before surgery, mm

Graph

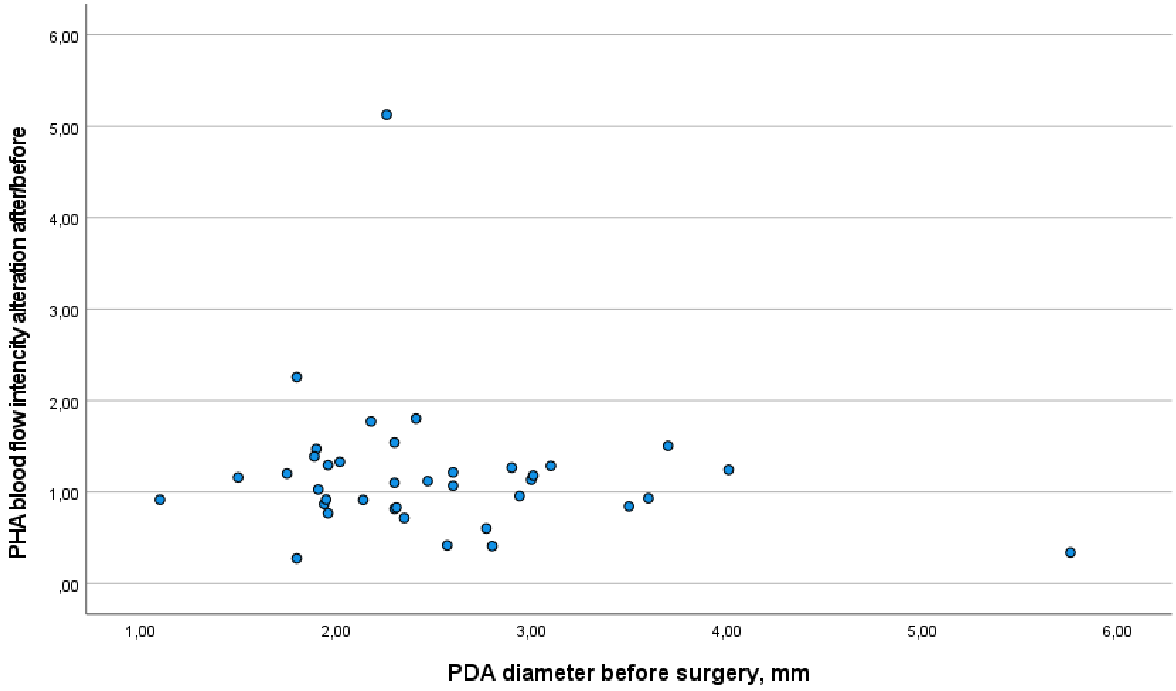
6,00
5,00
4,00
3,00
2,00
1,00
,00
1,00
2,00
3,00
4,00
5,00
6,00
PHA blood flow intencity alteration after/before
PDA diameter before surgery, mm

Graph

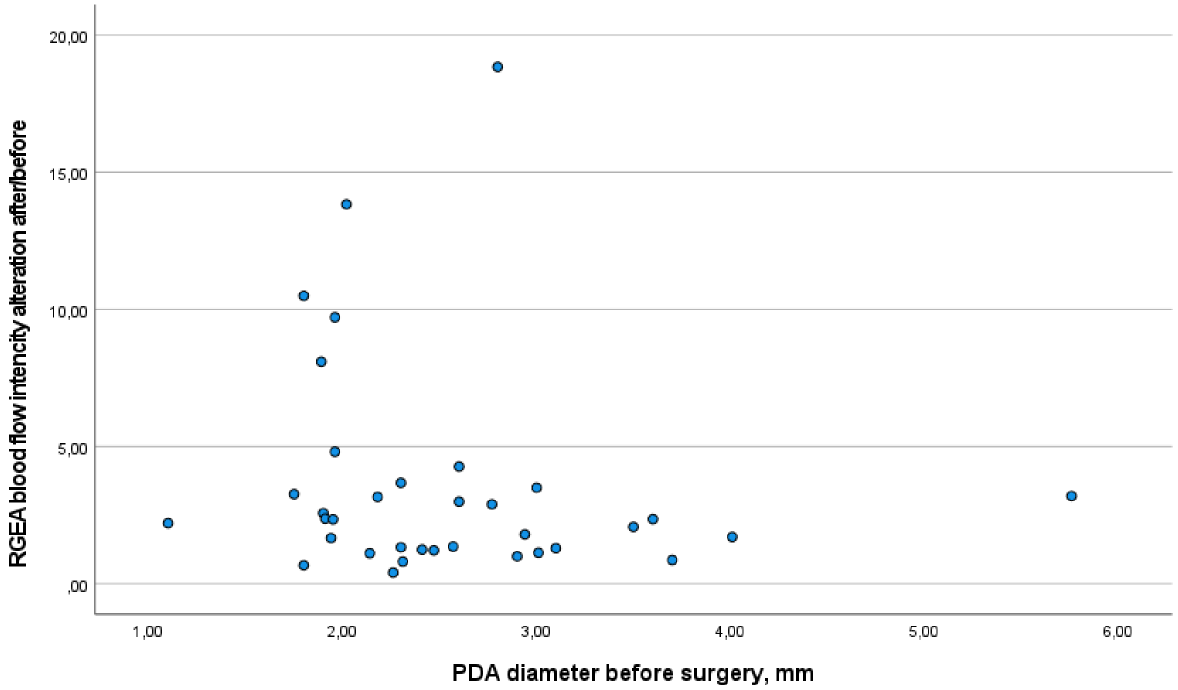


Correlations

**Correlations**

| | | PDA diameter before surgery. mm | GDA blood flow intencity alteration after/before | PDA blood flow intencity alteration after/before | PHA blood flow intencity alteration after/before | RGEA blood flow intencity alteration after/before |
|---|---|---|---|---|---|---|
| PDA diameter before surgery. mm | Pearson Correlation | 1 | .198 | -.413 | -.161 | -.144 |
| | Sig. (2-tailed) | | .234 | .010 | .333 | .409 |
| | N | 38 | 38 | 38 | 38 | 35 |
| GDA blood flow intencity alteration after/before | Pearson Correlation | .198 | 1 | -.042 | -.057 | -.057 |
| | Sig. (2-tailed) | .234 | | .800 | .735 | .744 |
| | N | 38 | 38 | 38 | 38 | 35 |
| PDA blood flow intencity alteration after/before | Pearson Correlation | -.413 | -.042 | 1 | -.015 | -.033 |
| | Sig. (2-tailed) | .010 | .800 | | .926 | .851 |
| | N | 38 | 38 | 38 | 38 | 35 |
| PHA blood flow intencity alteration after/before | Pearson Correlation | -.161 | -.057 | -.015 | 1 | -.246 |
| | Sig. (2-tailed) | .333 | .735 | .926 | | .155 |
| | N | 38 | 38 | 38 | 38 | 35 |
| RGEA blood flow intencity alteration after/before | Pearson Correlation | -.144 | -.057 | -.033 | -.246 | 1 |
| | Sig. (2-tailed) | .409 | .744 | .851 | .155 | |

| | | | | | |
|---|---|---|---|---|---|
| N | 35 | 35 | 35 | 35 | 35 |

Nonparametric Correlations

**Correlations**

| | | | PDA diameter before surgery. mm | GDA blood flow intencity alteration after/before | PDA blood flow intencity alteration after/before | PHA blood flow intencity alteration after/before | RGEA blood flow intencity alteration after/before |
|---|---|---|---|---|---|---|---|
| Spearman's rho | PDA diameter before surgery. mm | Correlation Coefficient | 1.000 | .227 | -.693 | -.115 | -.207 |
| | | Sig. (2-tailed) | . | .170 | .000 | .490 | .233 |
| | | N | 38 | 38 | 38 | 38 | 35 |
| | GDA blood flow intencity alteration after/before | Correlation Coefficient | .227 | 1.000 | -.246 | -.109 | -.057 |
| | | Sig. (2-tailed) | .170 | . | .137 | .515 | .746 |
| | | N | 38 | 38 | 38 | 38 | 35 |
| | PDA blood flow intencity alteration after/before | Correlation Coefficient | -.693 | -.246 | 1.000 | .072 | .268 |
| | | Sig. (2-tailed) | .000 | .137 | . | .669 | .120 |
| | | N | 38 | 38 | 38 | 38 | 35 |
| | PHA blood flow intencity alteration after/before | Correlation Coefficient | -.115 | -.109 | .072 | 1.000 | -.198 |
| | | Sig. (2-tailed) | .490 | .515 | .669 | . | .254 |
| | | N | 38 | 38 | 38 | 38 | 35 |
| | RGEA blood flow intencity alteration after/before | Correlation Coefficient | -.207 | -.057 | .268 | -.198 | 1.000 |
| | | Sig. (2-tailed) | .233 | .746 | .120 | .254 | . |
| | | N | 35 | 35 | 35 | 35 | 35 |

Graph

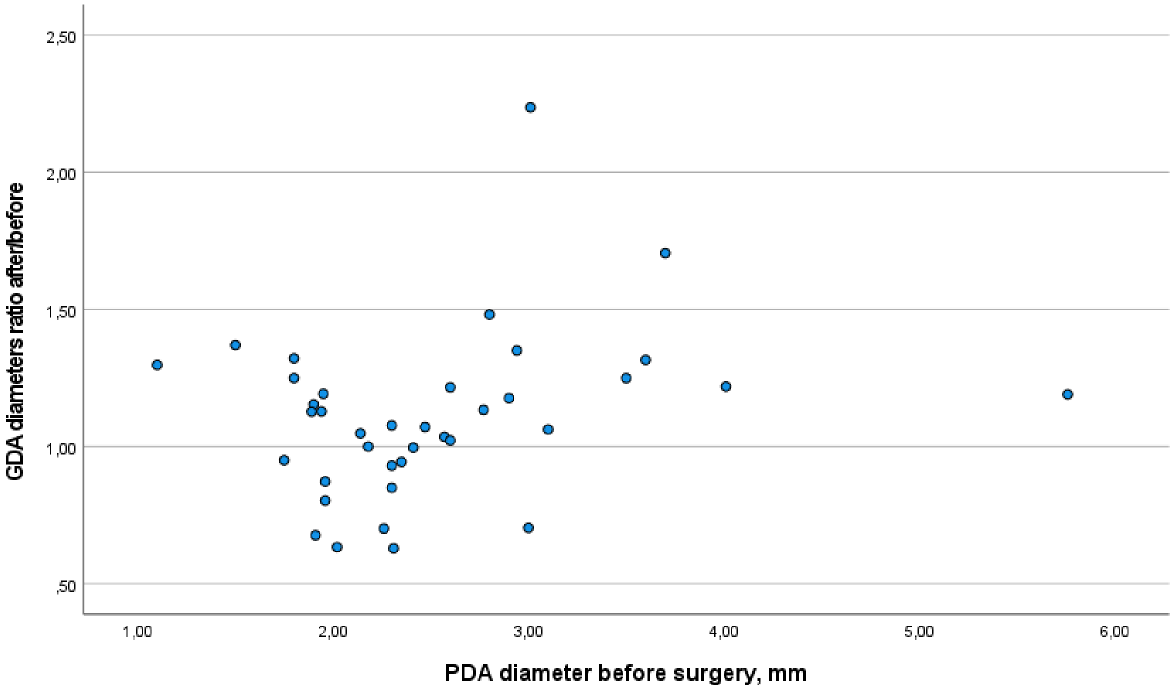
GDA diameters ratio after/before
2,50
2,00
1,50
1,00
,50
1,00
2,00
3,00
4,00
5,00
6,00
PDA diameter before surgery, mm

Graph

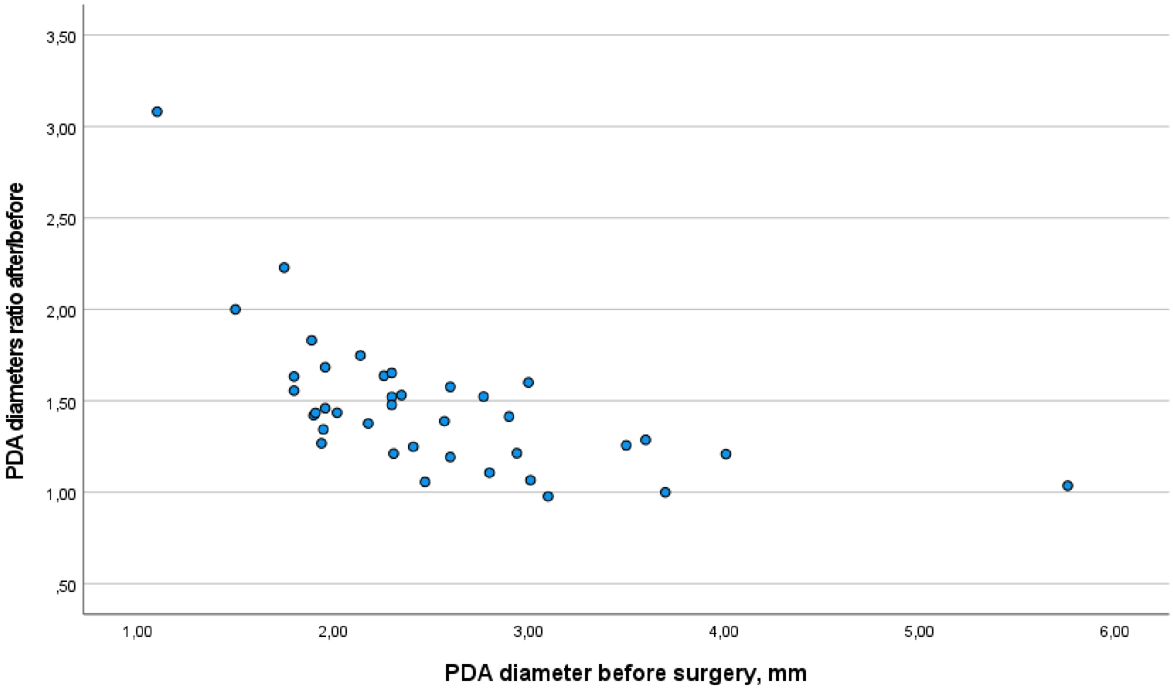
PDA diameters ratio after/before
3,50
3,00
2,50
2,00
1,50
1,00
,50
1,00
2,00
3,00
4,00
5,00
6,00
PDA diameter before surgery, mm

Graph

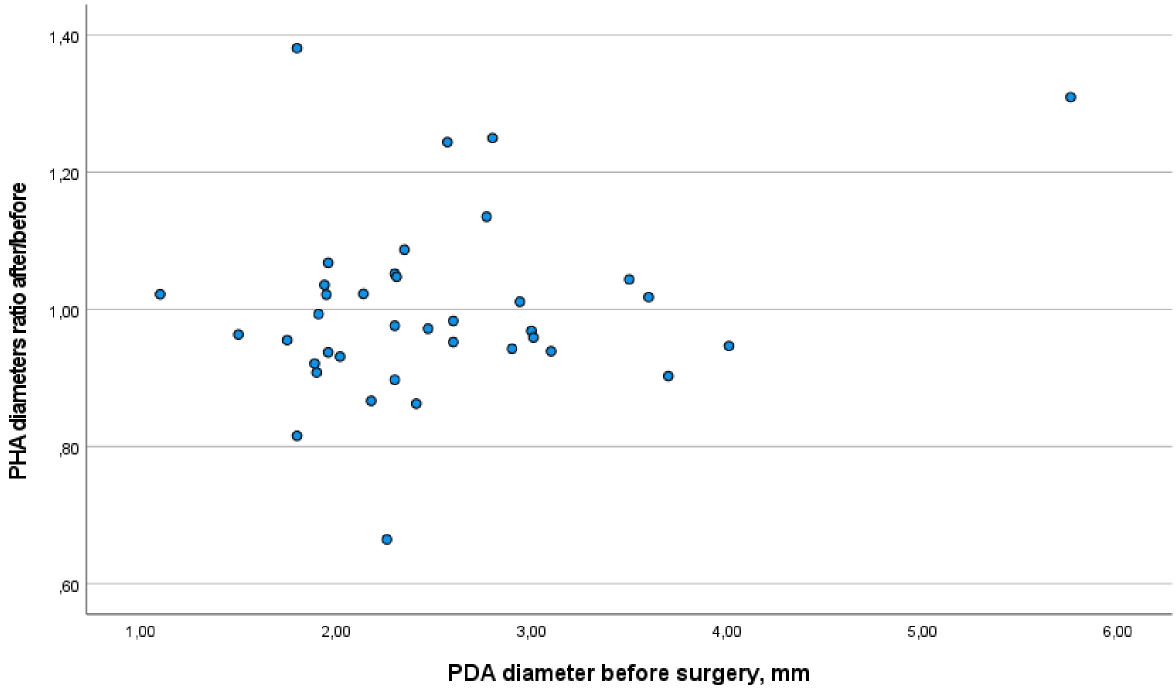


Graph

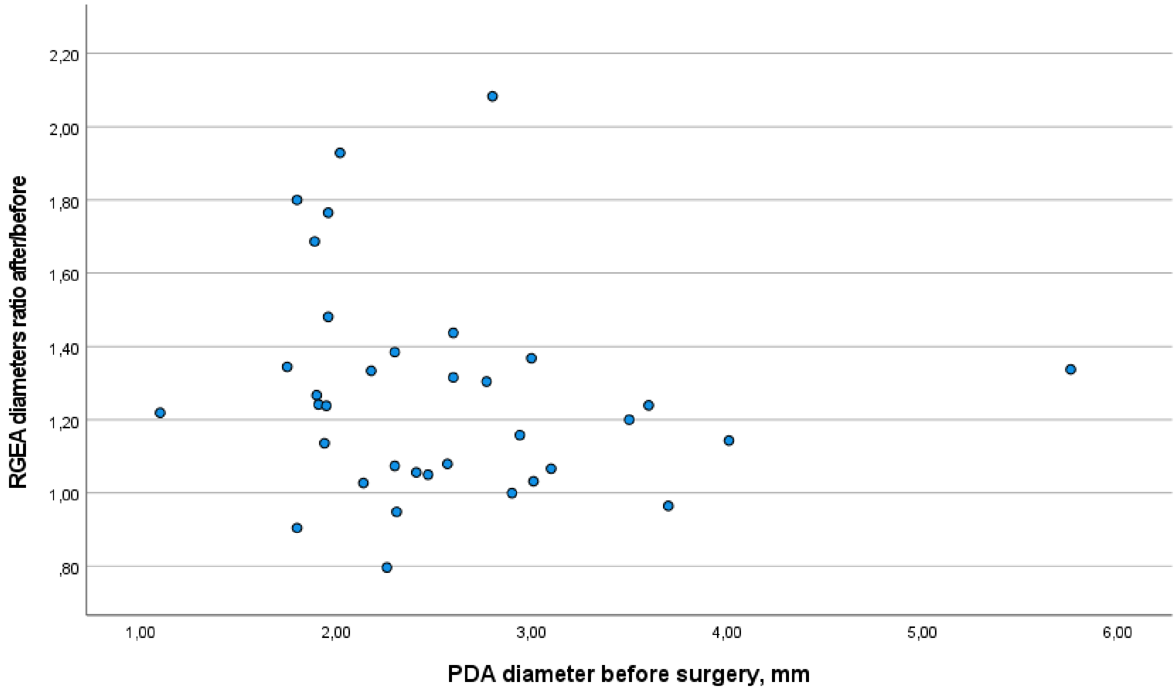


Correlations

**Correlations**

| | | PDA diameter before surgery. mm | GDA diameters ratio after/before | PDA diameters ratio after/before | PHA diameters ratio after/before | RGEA diameters ratio after/before |
|---|---|---|---|---|---|---|
| PDA diameter before surgery. mm | Pearson Correlation | 1 | .269 | -.609 | .254 | -.141 |

| | | | | | | |
|---|---|---|---|---|---|---|
| | Sig. (2-tailed) | | .102 | .000 | .123 | .419 |
| | N | 38 | 38 | 38 | 38 | 35 |
| GDA diameters ratio after/before | Pearson Correlation | .269 | 1 | -.159 | .152 | -.073 |
| | Sig. (2-tailed) | .102 | | .341 | .361 | .678 |
| | N | 38 | 38 | 38 | 38 | 35 |
| PDA diameters ratio after/before | Pearson Correlation | -.609 | -.159 | 1 | -.107 | .111 |
| | Sig. (2-tailed) | .000 | .341 | | .522 | .524 |
| | N | 38 | 38 | 38 | 38 | 35 |
| PHA diameters ratio after/before | Pearson Correlation | .254 | .152 | -.107 | 1 | .403 |
| | Sig. (2-tailed) | .123 | .361 | .522 | | .016 |
| | N | 38 | 38 | 38 | 38 | 35 |
| RGEA diameters ratio after/before | Pearson Correlation | -.141 | -.073 | .111 | .403 | 1 |
| | Sig. (2-tailed) | .419 | .678 | .524 | .016 | |
| | N | 35 | 35 | 35 | 35 | 35 |

Nonparametric Correlations

**Correlations**

| | | | PDA diameter before surgery. mm | GDA diameters ratio after/before | PDA diameters ratio after/before | PHA diameters ratio after/before | RGEA diameters ratio after/before |
|---|---|---|---|---|---|---|---|
| Spearman's rho | PDA diameter before surgery. mm | Correlation Coefficient | 1.000 | .224 | -.693 | .115 | -.207 |
| | | Sig. (2-tailed) | . | .176 | .000 | .490 | .233 |
| | | N | 38 | 38 | 38 | 38 | 35 |
| | GDA diameters ratio after/before | Correlation Coefficient | .224 | 1.000 | -.244 | .106 | -.059 |
| | | Sig. (2-tailed) | .176 | . | .140 | .527 | .737 |
| | | N | 38 | 38 | 38 | 38 | 35 |
| | PDA diameters ratio after/before | Correlation Coefficient | -.693 | -.244 | 1.000 | -.072 | .268 |
| | | Sig. (2-tailed) | .000 | .140 | . | .669 | .120 |
| | | N | 38 | 38 | 38 | 38 | 35 |
| | PHA diameters ratio after/before | Correlation Coefficient | .115 | .106 | -.072 | 1.000 | .198 |
| | | Sig. (2-tailed) | .490 | .527 | .669 | . | .254 |

| | | | | | | |
|---|---|---|---|---|---|---|
| | N | 38 | 38 | 38 | 38 | 35 |
| RGEA diameters ratio after/before | Correlation Coefficient | -.207 | -.059 | .268 | .198 | 1.000 |
| | Sig. (2-tailed) | .233 | .737 | .120 | .254 | . |
| | N | 35 | 35 | 35 | 35 | 35 |

Graph

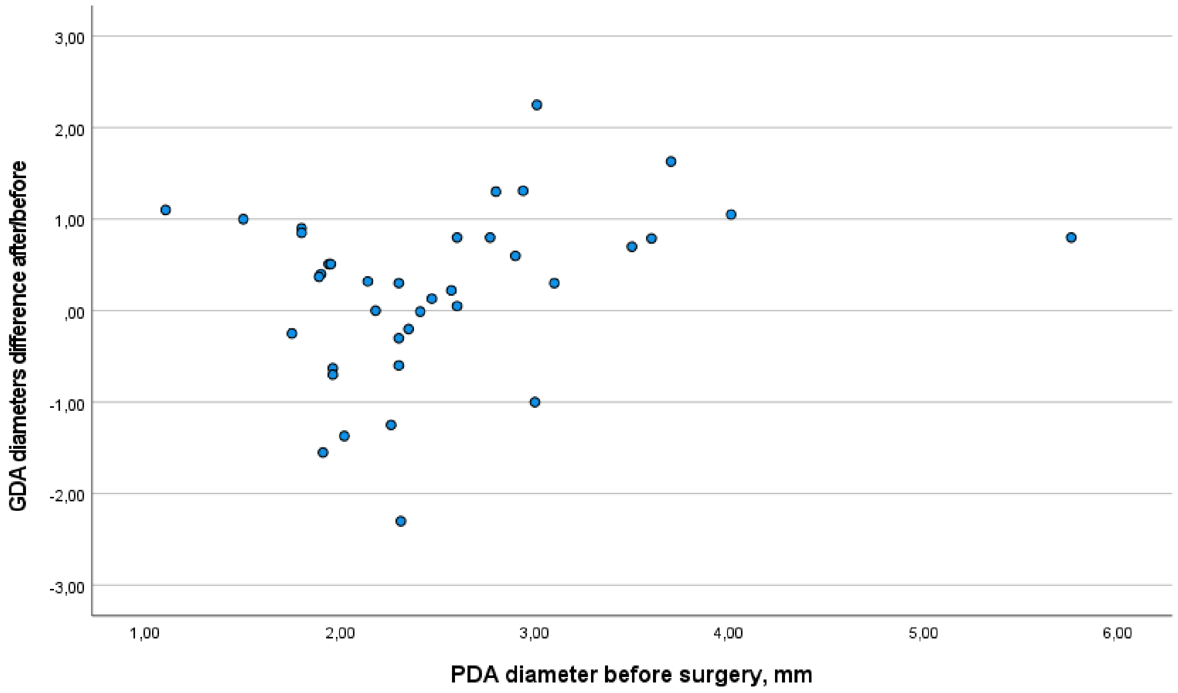


Graph

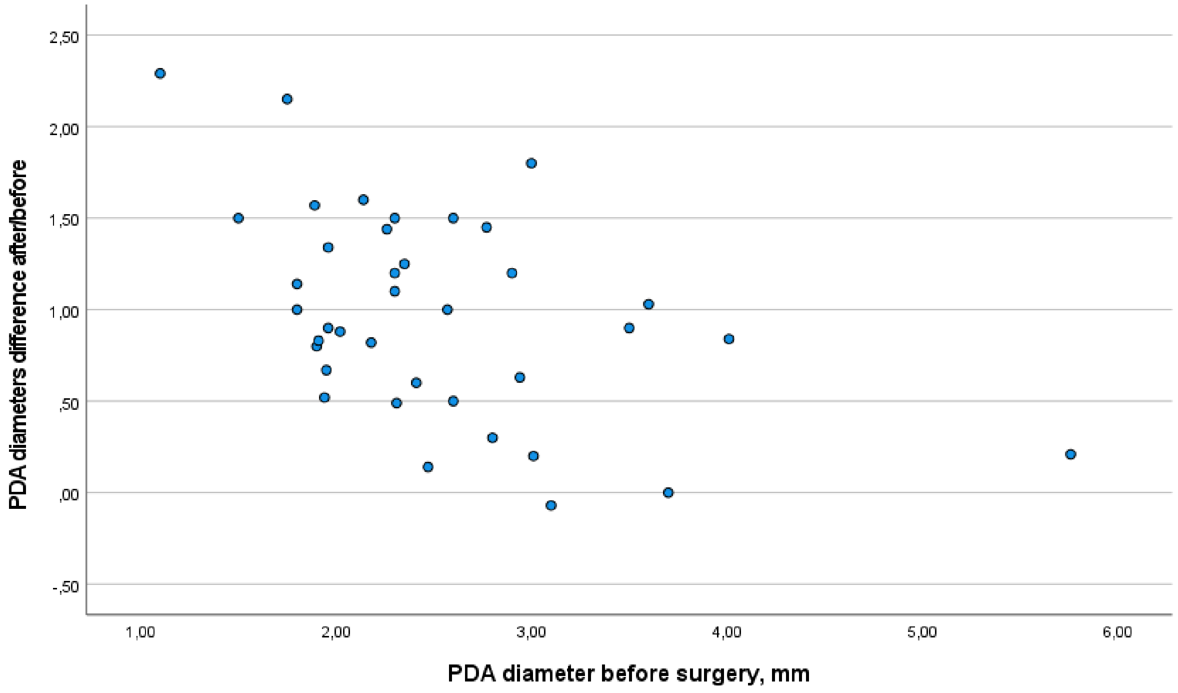


Graph

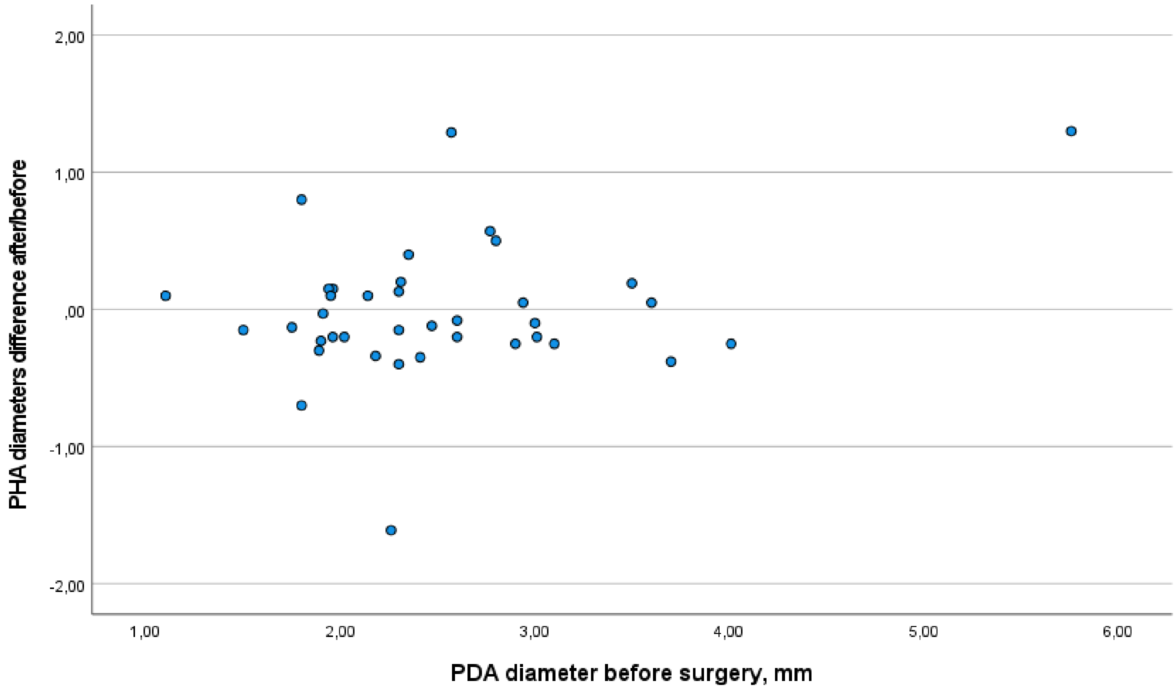


Graph

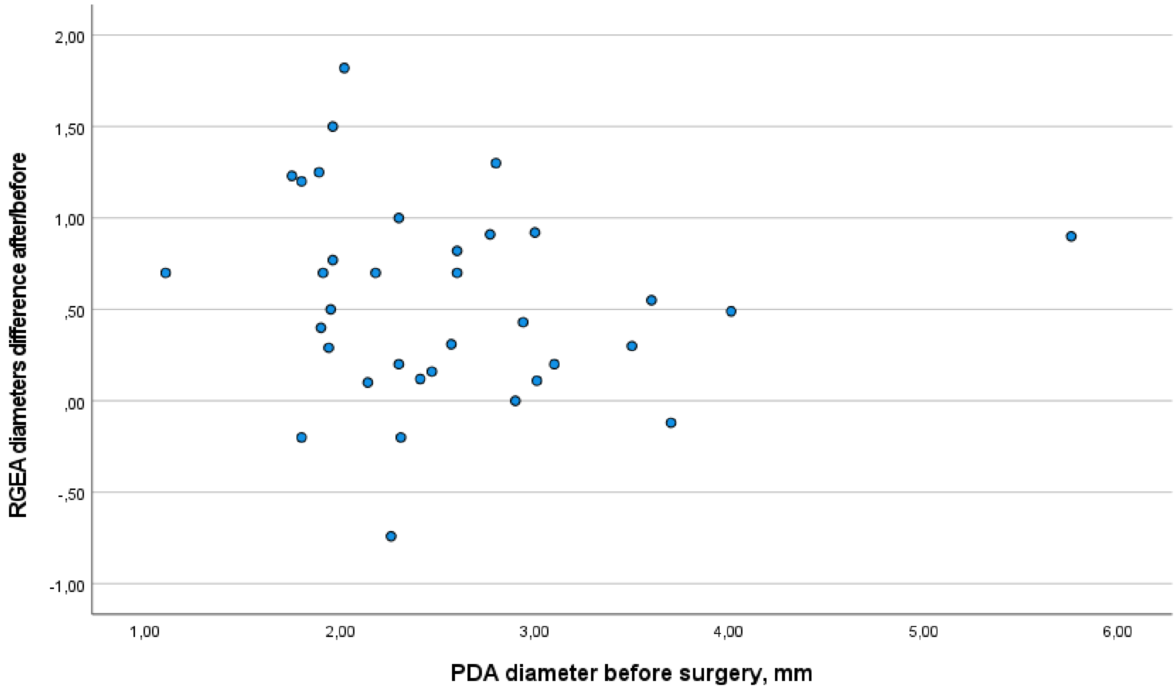


Correlations

**Correlations**

| | PDA diameter before surgery. mm | GDA diameters difference after/before | PDA diameters difference after/before | PHA diameters difference after/before | RGEA diameters difference after/before |
|---|---|---|---|---|---|

| | | | | | | |
|---|---|---|---|---|---|---|
| PDA diameter before surgery. mm | Pearson Correlation | 1 | .295 | -.477 | .294 | -.111 |
| | Sig. (2-tailed) | | .072 | .002 | .073 | .526 |
| | N | 38 | 38 | 38 | 38 | 35 |
| GDA diameters difference after/before | Pearson Correlation | .295 | 1 | -.187 | .176 | -.055 |
| | Sig. (2-tailed) | .072 | | .261 | .290 | .752 |
| | N | 38 | 38 | 38 | 38 | 35 |
| PDA diameters difference after/before | Pearson Correlation | -.477 | -.187 | 1 | -.126 | .241 |
| | Sig. (2-tailed) | .002 | .261 | | .450 | .163 |
| | N | 38 | 38 | 38 | 38 | 35 |
| PHA diameters difference after/before | Pearson Correlation | .294 | .176 | -.126 | 1 | .349 |
| | Sig. (2-tailed) | .073 | .290 | .450 | | .040 |
| | N | 38 | 38 | 38 | 38 | 35 |
| RGEA diameters difference after/before | Pearson Correlation | -.111 | -.055 | .241 | .349 | 1 |
| | Sig. (2-tailed) | .526 | .752 | .163 | .040 | |
| | N | 35 | 35 | 35 | 35 | 35 |

Nonparametric Correlations

**Correlations**

| | | | PDA diameter before surgery. mm | GDA diameters difference after/before | PDA diameters difference after/before | PHA diameters difference after/before | RGEA diameters difference after/before |
|---|---|---|---|---|---|---|---|
| Spearman's rho | PDA diameter before surgery. mm | Correlation Coefficient | 1.000 | .250 | -.430 | .065 | -.201 |
| | | Sig. (2-tailed) | . | .130 | .007 | .697 | .246 |
| | | N | 38 | 38 | 38 | 38 | 35 |
| | GDA diameters difference after/before | Correlation Coefficient | .250 | 1.000 | -.166 | .057 | -.095 |
| | | Sig. (2-tailed) | .130 | . | .318 | .732 | .587 |
| | | N | 38 | 38 | 38 | 38 | 35 |
| | PDA diameters difference after/before | Correlation Coefficient | -.430 | -.166 | 1.000 | -.002 | .255 |
| | | Sig. (2-tailed) | .007 | .318 | . | .990 | .139 |
| | | N | 38 | 38 | 38 | 38 | 35 |

| | | | | | | | |
|---|---|---|---|---|---|---|---|
| | PHA diameters difference after/before | Correlation Coefficient | .065 | .057 | -.002 | 1.000 | .235 |
| | | Sig. (2-tailed) | .697 | .732 | .990 | . | .174 |
| | | N | 38 | 38 | 38 | 38 | 35 |
| | RGEA diameters difference after/before | Correlation Coefficient | -.201 | -.095 | .255 | .235 | 1.000 |
| | | Sig. (2-tailed) | .246 | .587 | .139 | .174 | . |
| | | N | 35 | 35 | 35 | 35 | 35 |

Graph

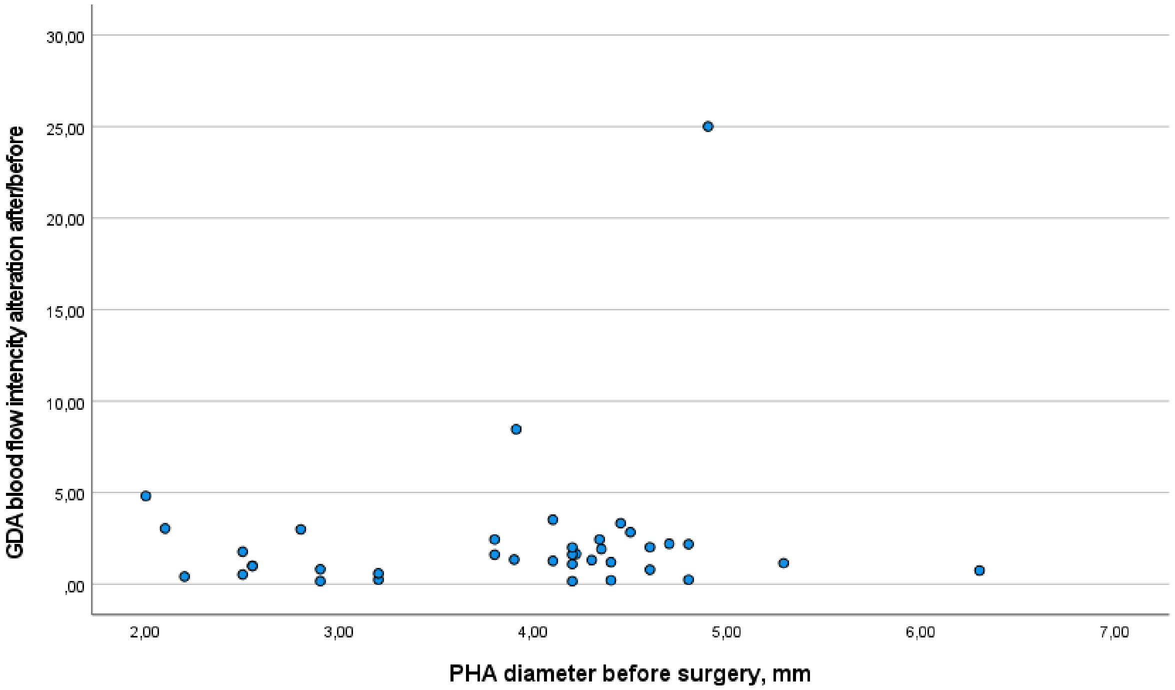


Graph

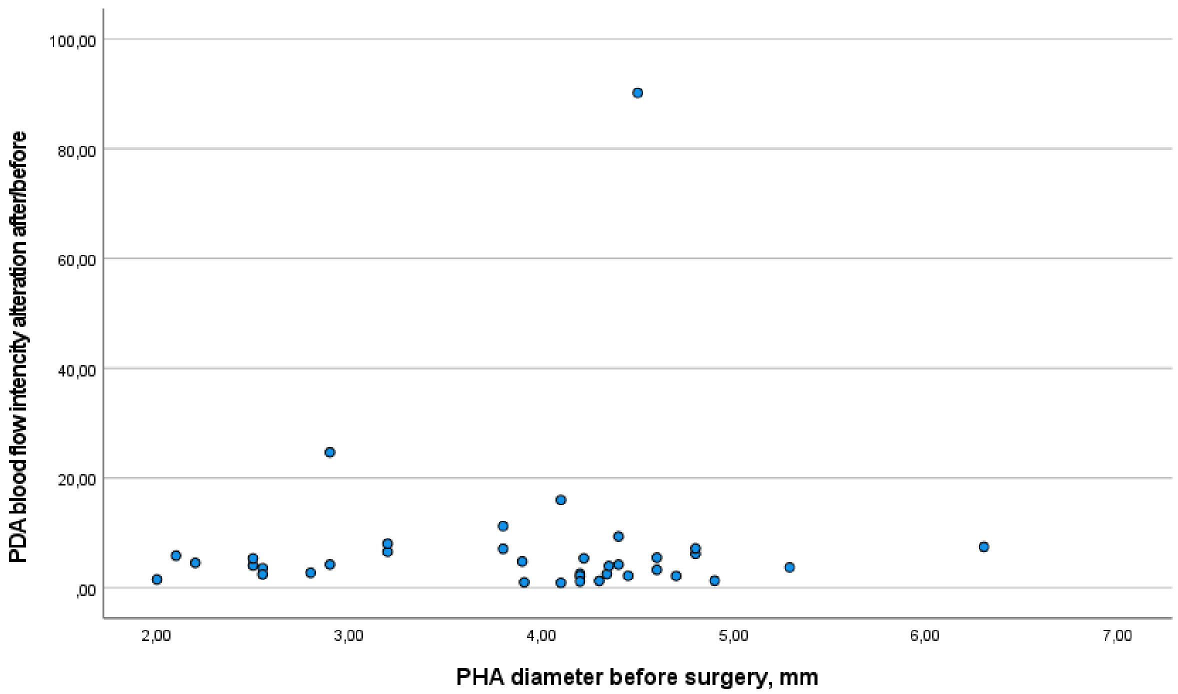
PDA blood flow intencity alteration after/before
100,00
80,00
60,00
40,00
20,00
,00
2,00
3,00
4,00
5,00
6,00
7,00
PHA diameter before surgery, mm

Graph

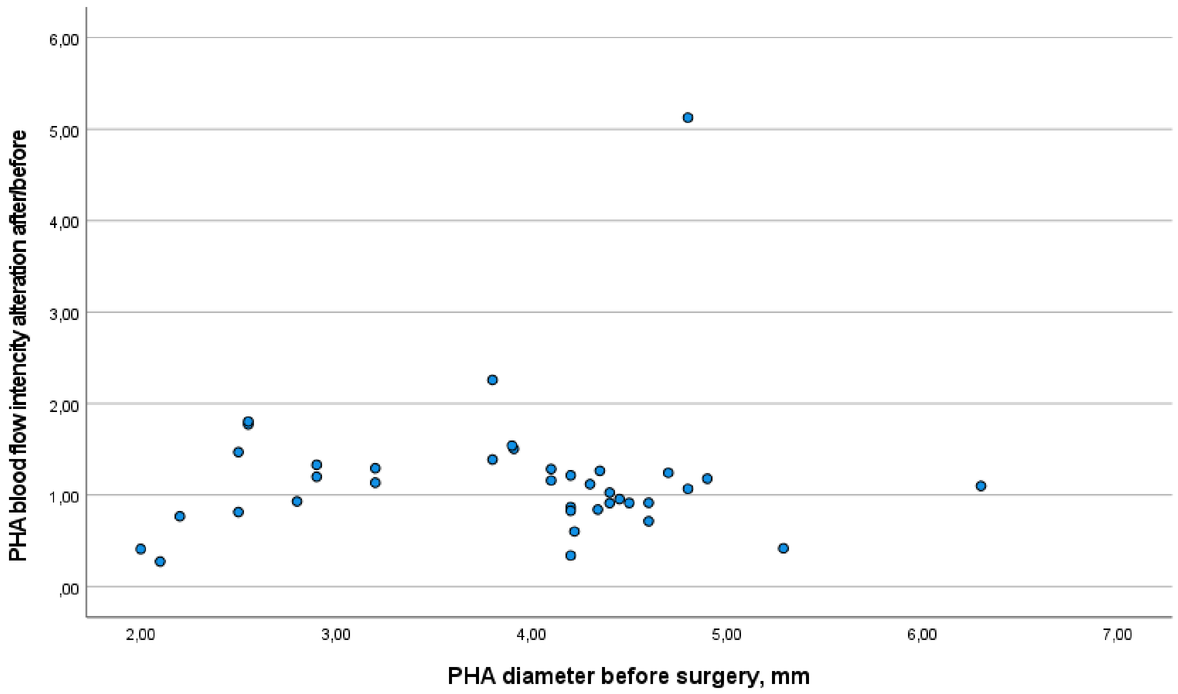
PHA blood flow intencity alteration after/before
6,00
5,00
4,00
3,00
2,00
1,00
,00
2,00
3,00
4,00
5,00
6,00
7,00
PHA diameter before surgery, mm

Graph

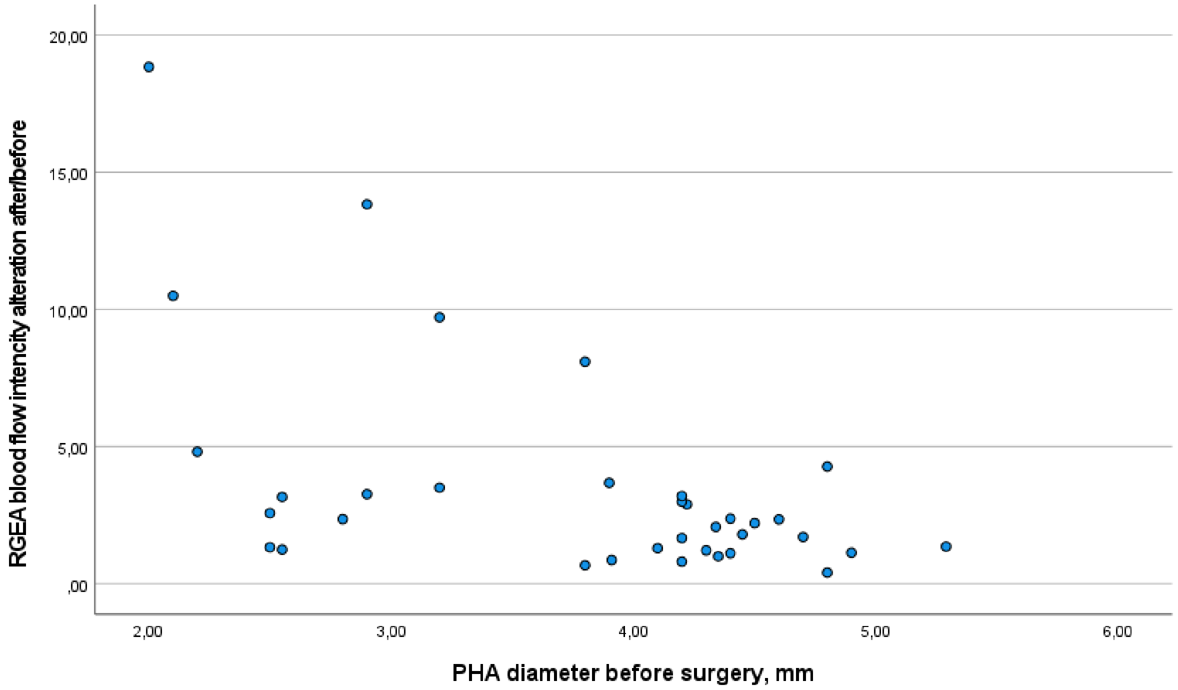


Correlations

**Correlations**

| | | PHA diameter before surgery. mm | GDA blood flow intencity alteration after/before | PDA blood flow intencity alteration after/before | PHA blood flow intencity alteration after/before | RGEA blood flow intencity alteration after/before |
|---|---|---|---|---|---|---|
| PHA diameter before surgery. mm | Pearson Correlation | 1 | .142 | .078 | .096 | -.537 |
| | Sig. (2-tailed) | | .396 | .640 | .566 | .001 |
| | N | 38 | 38 | 38 | 38 | 35 |
| GDA blood flow intencity alteration after/before | Pearson Correlation | .142 | 1 | -.042 | -.057 | -.057 |
| | Sig. (2-tailed) | .396 | | .800 | .735 | .744 |
| | N | 38 | 38 | 38 | 38 | 35 |
| PDA blood flow intencity alteration after/before | Pearson Correlation | .078 | -.042 | 1 | -.015 | -.033 |
| | Sig. (2-tailed) | .640 | .800 | | .926 | .851 |
| | N | 38 | 38 | 38 | 38 | 35 |
| PHA blood flow intencity alteration after/before | Pearson Correlation | .096 | -.057 | -.015 | 1 | -.246 |
| | Sig. (2-tailed) | .566 | .735 | .926 | | .155 |
| | N | 38 | 38 | 38 | 38 | 35 |
| RGEA blood flow intencity alteration after/before | Pearson Correlation | -.537 | -.057 | -.033 | -.246 | 1 |
| | Sig. (2-tailed) | .001 | .744 | .851 | .155 | |

| | | | | | | |
|---|---|---|---|---|---|---|
| N | | 35 | 35 | 35 | 35 | 35 |

Nonparametric Correlations

**Correlations**

| | | | PHA diameter before surgery. mm | GDA blood flow intencity alteration after/before | PDA blood flow intencity alteration after/before | PHA blood flow intencity alteration after/before | RGEA blood flow intencity alteration after/before |
|---|---|---|---|---|---|---|---|
| Spearman's rho | PHA diameter before surgery. mm | Correlation Coefficient | 1.000 | .074 | -.011 | -.101 | -.465 |
| | | Sig. (2-tailed) | . | .658 | .947 | .545 | .005 |
| | | N | 38 | 38 | 38 | 38 | 35 |
| | GDA blood flow intencity alteration after/before | Correlation Coefficient | .074 | 1.000 | -.246 | -.109 | -.057 |
| | | Sig. (2-tailed) | .658 | . | .137 | .515 | .746 |
| | | N | 38 | 38 | 38 | 38 | 35 |
| | PDA blood flow intencity alteration after/before | Correlation Coefficient | -.011 | -.246 | 1.000 | .072 | .268 |
| | | Sig. (2-tailed) | .947 | .137 | . | .669 | .120 |
| | | N | 38 | 38 | 38 | 38 | 35 |
| | PHA blood flow intencity alteration after/before | Correlation Coefficient | -.101 | -.109 | .072 | 1.000 | -.198 |
| | | Sig. (2-tailed) | .545 | .515 | .669 | . | .254 |
| | | N | 38 | 38 | 38 | 38 | 35 |
| | RGEA blood flow intencity alteration after/before | Correlation Coefficient | -.465 | -.057 | .268 | -.198 | 1.000 |
| | | Sig. (2-tailed) | .005 | .746 | .120 | .254 | . |
| | | N | 35 | 35 | 35 | 35 | 35 |

Graph

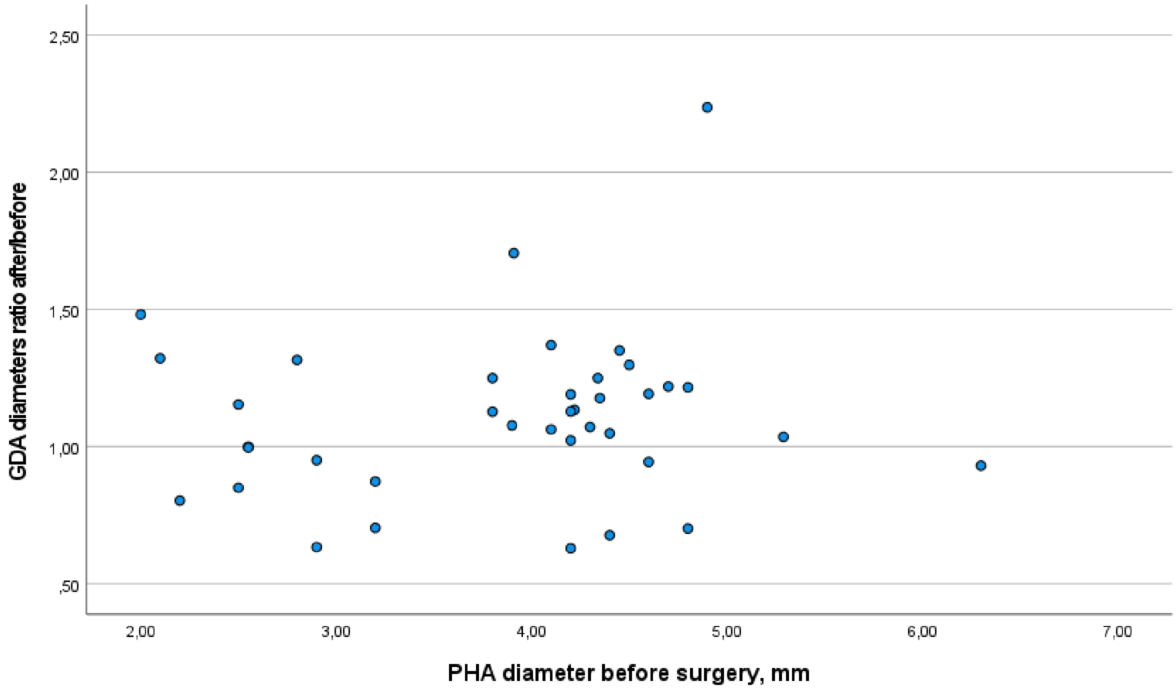

2,50
2,00
1,50
1,00
,50
GDA diameters ratio after/before
2,00
3,00
4,00
5,00
6,00
7,00
PHA diameter before surgery, mm


Graph

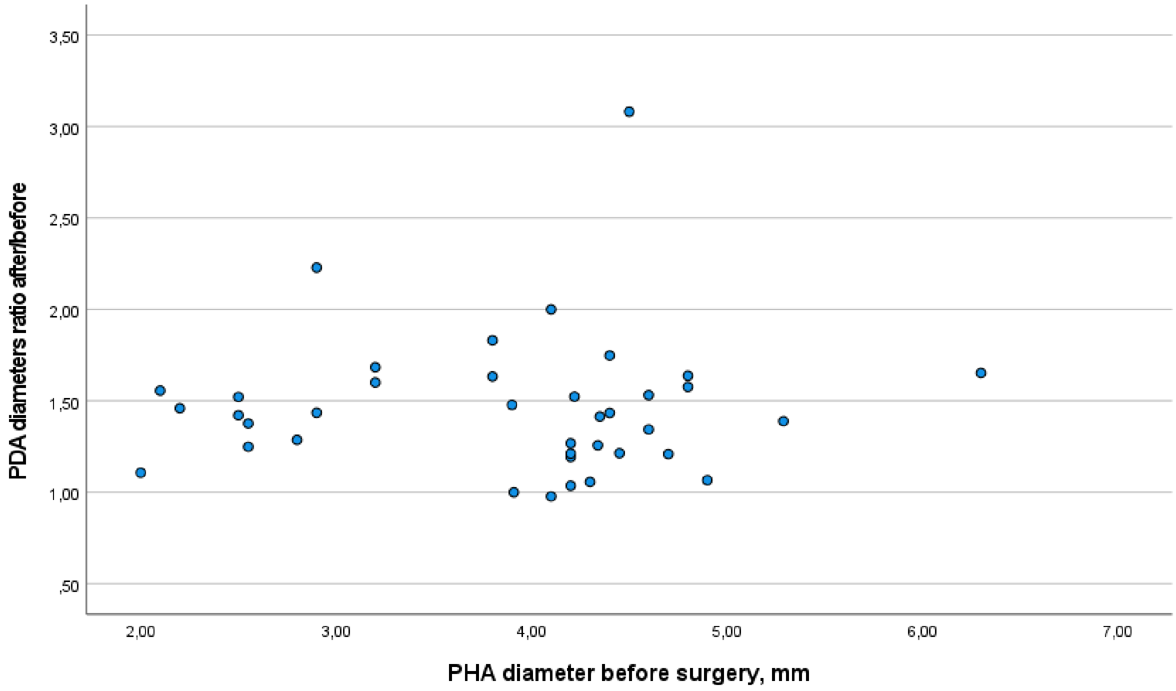

3,50
3,00
2,50
2,00
1,50
1,00
,50
PDA diameters ratio after/before
2,00
3,00
4,00
5,00
6,00
7,00
PHA diameter before surgery, mm


Graph

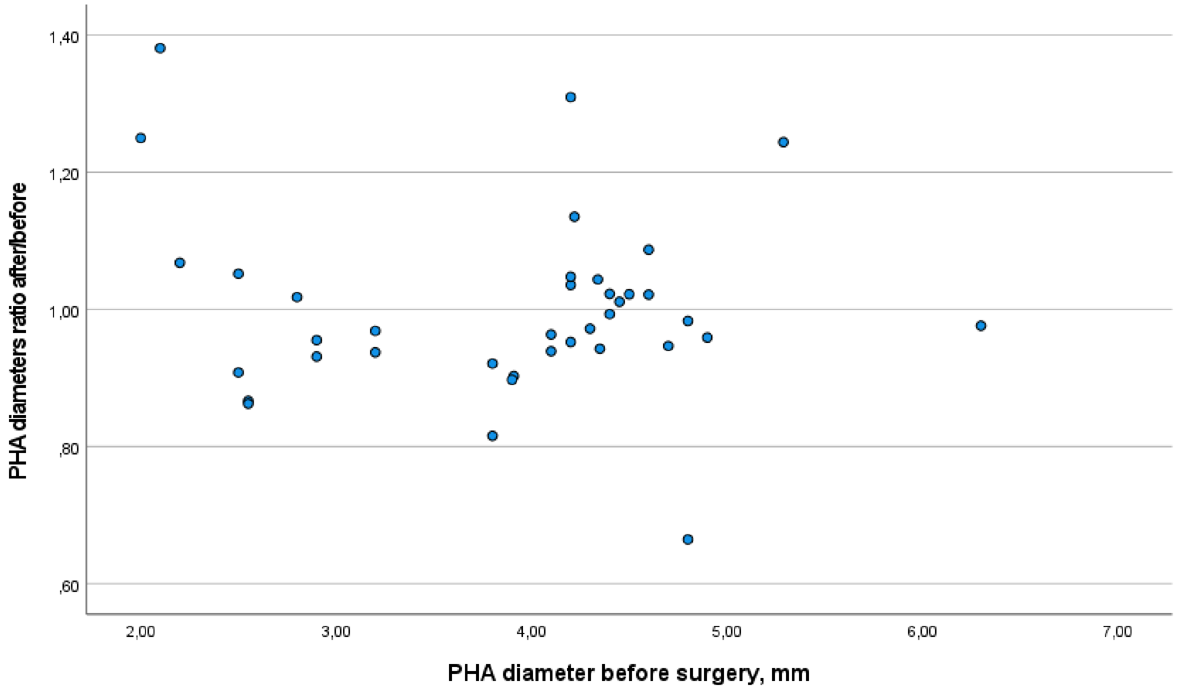


Graph

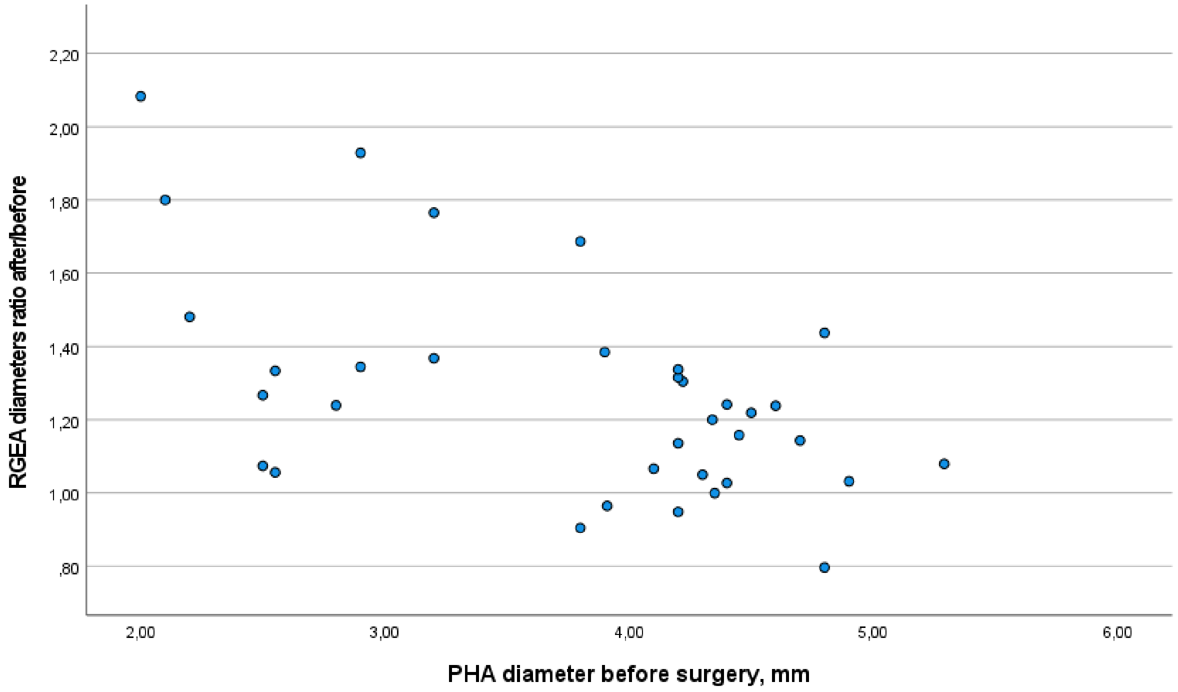


Correlations

**Correlations**

| | | PHA diameter before surgery. mm | GDA diameters ratio after/before | PDA diameters ratio after/before | PHA diameters ratio after/before | RGEA diameters ratio after/before |
|---|---|---|---|---|---|---|
| PHA diameter before surgery. mm | Pearson Correlation | 1 | .110 | .029 | -.120 | -.546 |

| | | | | | | |
|---|---|---|---|---|---|---|
| | Sig. (2-tailed) | | .509 | .864 | .475 | .001 |
| | N | 38 | 38 | 38 | 38 | 35 |
| GDA diameters ratio after/before | Pearson Correlation | .110 | 1 | -.159 | .152 | -.073 |
| | Sig. (2-tailed) | .509 | | .341 | .361 | .678 |
| | N | 38 | 38 | 38 | 38 | 35 |
| PDA diameters ratio after/before | Pearson Correlation | .029 | -.159 | 1 | -.107 | .111 |
| | Sig. (2-tailed) | .864 | .341 | | .522 | .524 |
| | N | 38 | 38 | 38 | 38 | 35 |
| PHA diameters ratio after/before | Pearson Correlation | -.120 | .152 | -.107 | 1 | .403 |
| | Sig. (2-tailed) | .475 | .361 | .522 | | .016 |
| | N | 38 | 38 | 38 | 38 | 35 |
| RGEA diameters ratio after/before | Pearson Correlation | -.546 | -.073 | .111 | .403 | 1 |
| | Sig. (2-tailed) | .001 | .678 | .524 | .016 | |
| | N | 35 | 35 | 35 | 35 | 35 |

Nonparametric Correlations

**Correlations**

| | | | PHA diameter before surgery. mm | GDA diameters ratio after/before | PDA diameters ratio after/before | PHA diameters ratio after/before | RGEA diameters ratio after/before |
|---|---|---|---|---|---|---|---|
| Spearman's rho | PHA diameter before surgery. mm | Correlation Coefficient | 1.000 | .073 | -.011 | .101 | -.465 |
| | | Sig. (2-tailed) | . | .663 | .947 | .545 | .005 |
| | | N | 38 | 38 | 38 | 38 | 35 |
| | GDA diameters ratio after/before | Correlation Coefficient | .073 | 1.000 | -.244 | .106 | -.059 |
| | | Sig. (2-tailed) | .663 | . | .140 | .527 | .737 |
| | | N | 38 | 38 | 38 | 38 | 35 |
| | PDA diameters ratio after/before | Correlation Coefficient | -.011 | -.244 | 1.000 | -.072 | .268 |
| | | Sig. (2-tailed) | .947 | .140 | . | .669 | .120 |
| | | N | 38 | 38 | 38 | 38 | 35 |
| | PHA diameters ratio after/before | Correlation Coefficient | .101 | .106 | -.072 | 1.000 | .198 |
| | | Sig. (2-tailed) | .545 | .527 | .669 | . | .254 |
| | | N | 38 | 38 | 38 | 38 | 35 |

| | | | | | | | |
|---|---|---|---|---|---|---|---|
| | RGEA diameters ratio after/before | Correlation Coefficient | -.465 | -.059 | .268 | .198 | 1.000 |
| | | Sig. (2-tailed) | .005 | .737 | .120 | .254 | . |
| | | N | 35 | 35 | 35 | 35 | 35 |

Graph

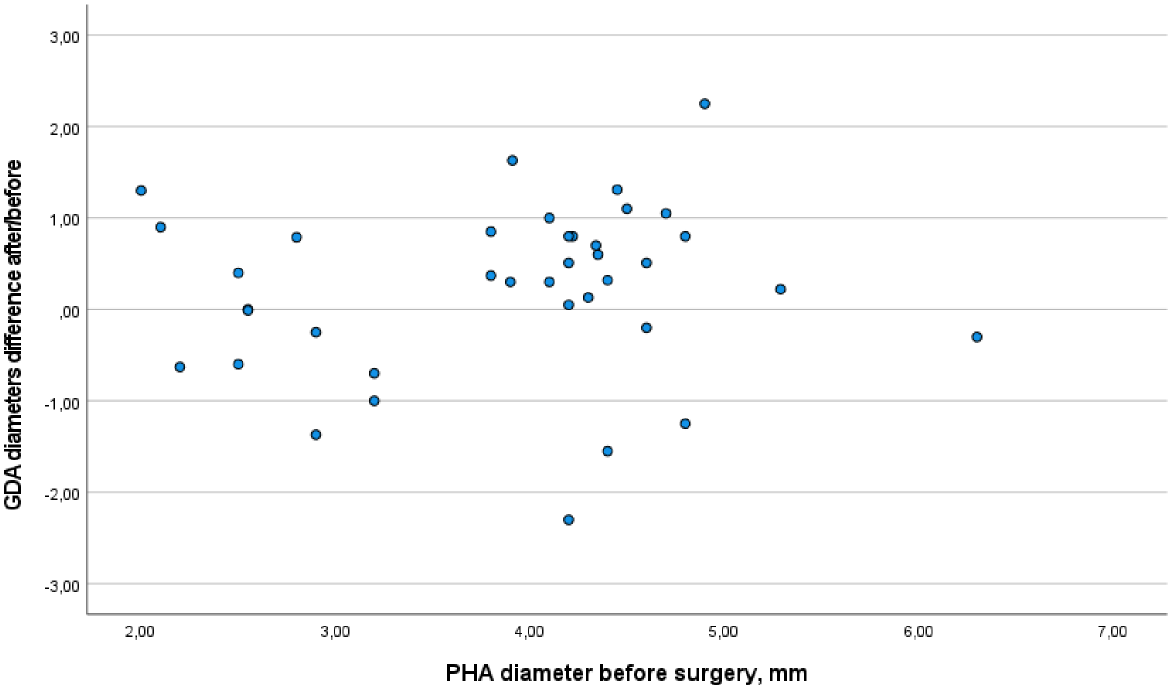


Graph

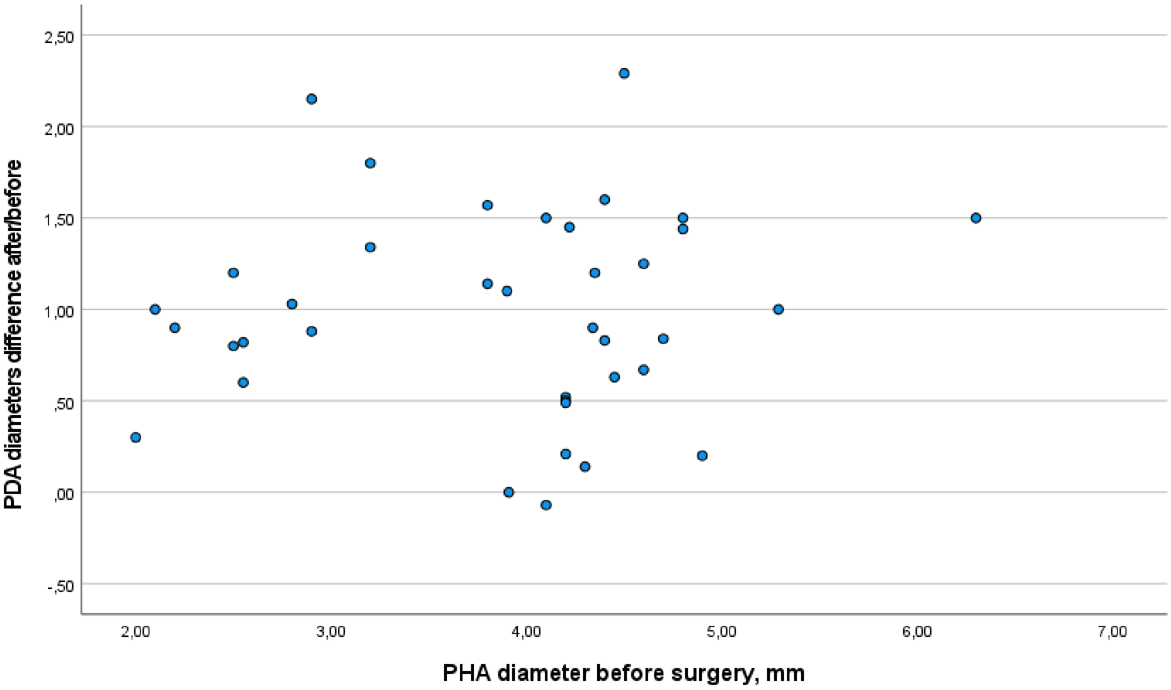


Graph

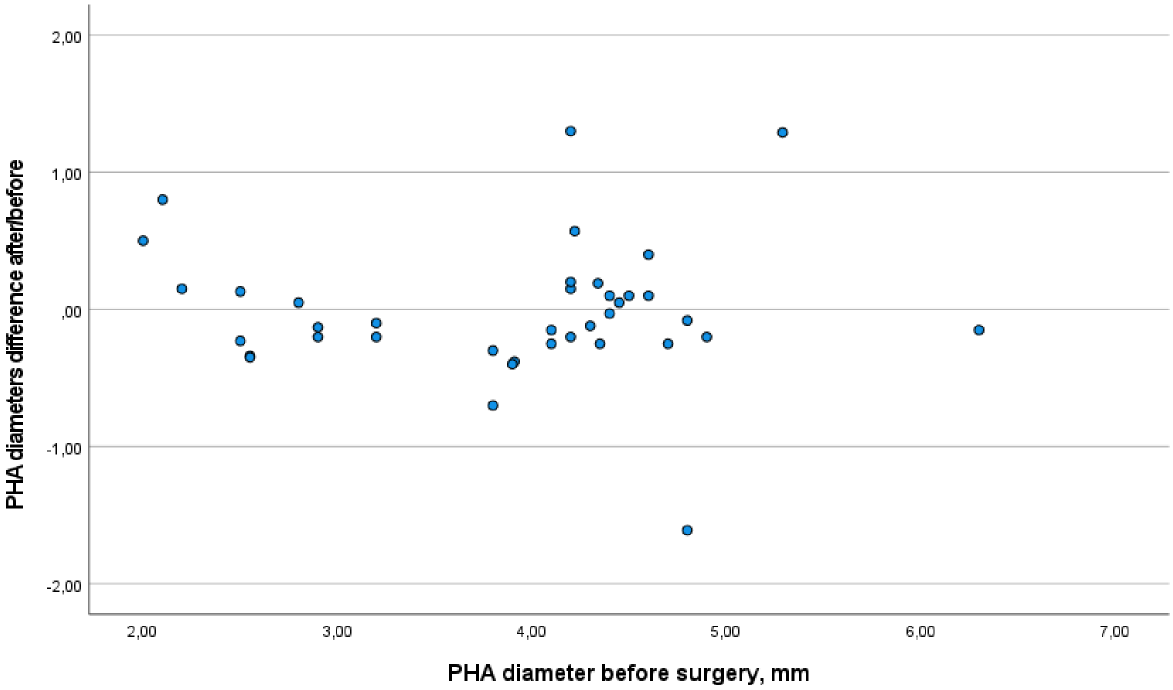


Graph

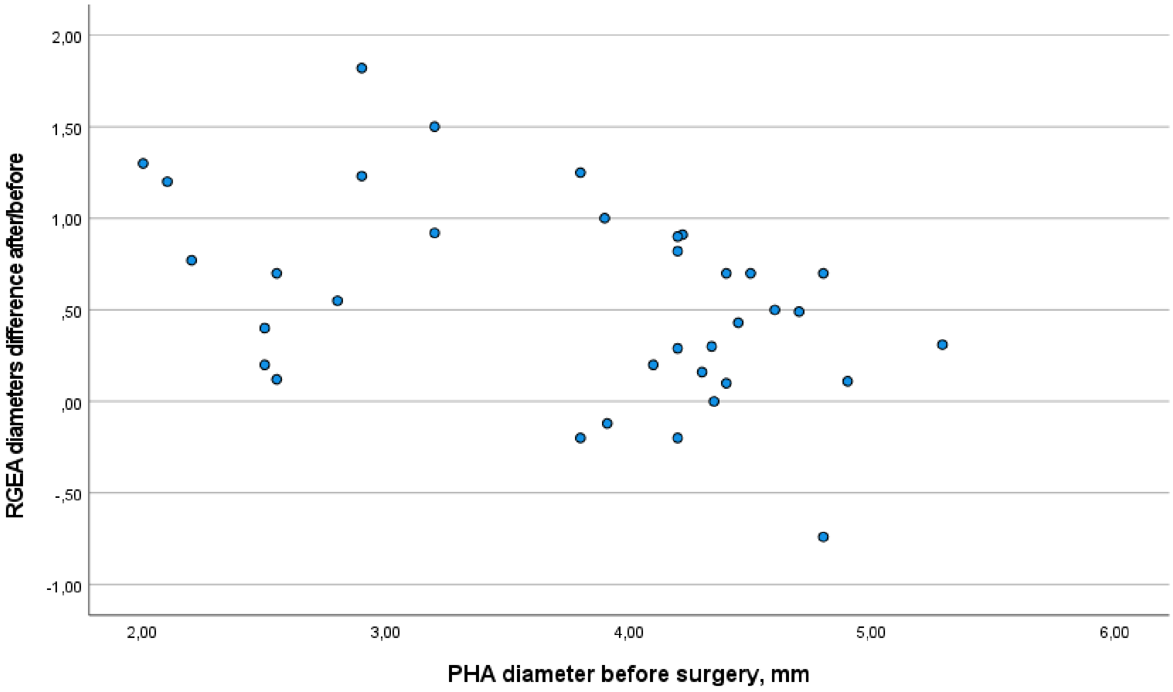


Correlations

**Correlations**

| | PHA diameter before surgery. mm | GDA diameters difference after/before | PDA diameters difference after/before | PHA diameters difference after/before | RGEA diameters difference after/before |
|---|---|---|---|---|---|

| | | | | | | |
|---|---|---|---|---|---|---|
| PHA diameter before surgery. mm | Pearson Correlation | 1 | .105 | .059 | -.032 | -.433 |
| | Sig. (2-tailed) | | .529 | .724 | .851 | .009 |
| | N | 38 | 38 | 38 | 38 | 35 |
| GDA diameters difference after/before | Pearson Correlation | .105 | 1 | -.187 | .176 | -.055 |
| | Sig. (2-tailed) | .529 | | .261 | .290 | .752 |
| | N | 38 | 38 | 38 | 38 | 35 |
| PDA diameters difference after/before | Pearson Correlation | .059 | -.187 | 1 | -.126 | .241 |
| | Sig. (2-tailed) | .724 | .261 | | .450 | .163 |
| | N | 38 | 38 | 38 | 38 | 35 |
| PHA diameters difference after/before | Pearson Correlation | -.032 | .176 | -.126 | 1 | .349 |
| | Sig. (2-tailed) | .851 | .290 | .450 | | .040 |
| | N | 38 | 38 | 38 | 38 | 35 |
| RGEA diameters difference after/before | Pearson Correlation | -.433 | -.055 | .241 | .349 | 1 |
| | Sig. (2-tailed) | .009 | .752 | .163 | .040 | |
| | N | 35 | 35 | 35 | 35 | 35 |

Nonparametric Correlations

**Correlations**

| | | | PHA diameter before surgery. mm | GDA diameters difference after/before | PDA diameters difference after/before | PHA diameters difference after/before | RGEA diameters difference after/before |
|---|---|---|---|---|---|---|---|
| Spearman's rho | PHA diameter before surgery. mm | Correlation Coefficient | 1.000 | .132 | .099 | .056 | -.386 |
| | | Sig. (2-tailed) | . | .430 | .555 | .736 | .022 |
| | | N | 38 | 38 | 38 | 38 | 35 |
| | GDA diameters difference after/before | Correlation Coefficient | .132 | 1.000 | -.166 | .057 | -.095 |
| | | Sig. (2-tailed) | .430 | . | .318 | .732 | .587 |
| | | N | 38 | 38 | 38 | 38 | 35 |
| | PDA diameters difference after/before | Correlation Coefficient | .099 | -.166 | 1.000 | -.002 | .255 |
| | | Sig. (2-tailed) | .555 | .318 | . | .990 | .139 |
| | | N | 38 | 38 | 38 | 38 | 35 |
| | | Correlation Coefficient | .056 | .057 | -.002 | 1.000 | .235 |

| | | | | | | | |
|---|---|---|---|---|---|---|---|
| | PHA diameters difference after/before | Sig. (2-tailed) | .736 | .732 | .990 | . | .174 |
| | | N | 38 | 38 | 38 | 38 | 35 |
| | RGEA diameters difference after/before | Correlation Coefficient | -.386 | -.095 | .255 | .235 | 1.000 |
| | | Sig. (2-tailed) | .022 | .587 | .139 | .174 | . |
| | | N | 35 | 35 | 35 | 35 | 35 |

T-Test

**Group Statistics**

| | Type od parenchimal blood flow | N | Mean | Std. Deviation | Std. Error Mean |
|---|---|---|---|---|---|
| GDA blood flow intencity alteration after/before | collateral | 12 | 1.4574 | .79469 | .22941 |
| | main | 26 | 2.7957 | 4.86987 | .95506 |
| PDA blood flow intencity alteration after/before | collateral | 12 | 5.4721 | 2.72061 | .78537 |
| | main | 26 | 8.1805 | 17.50659 | 3.43333 |
| PHA blood flow intencity alteration after/before | collateral | 12 | 1.4046 | 1.27309 | .36751 |
| | main | 26 | 1.0835 | .37297 | .07315 |
| RGEA blood flow intencity alteration after/before | collateral | 12 | 3.5111 | 3.04395 | .87871 |
| | main | 23 | 3.5725 | 4.49047 | .93633 |
| GDA diameters ratio after/before | collateral | 12 | 1.0631 | .17571 | .05072 |
| | main | 26 | 1.1296 | .34891 | .06843 |
| PDA diameters ratio after/before | collateral | 12 | 1.4979 | .18635 | .05379 |
| | main | 26 | 1.4505 | .44903 | .08806 |
| PHA diameters ratio after/before | collateral | 12 | 1.0003 | .18948 | .05470 |
| | main | 26 | 1.0002 | .10093 | .01979 |
| RGEA diameters ratio after/before | collateral | 12 | 1.2844 | .28779 | .08308 |
| | main | 23 | 1.2608 | .30165 | .06290 |
| GDA diameters difference after/before | collateral | 12 | .2475 | .66501 | .19197 |
| | main | 26 | .2254 | 1.04274 | .20450 |
| PDA diameters difference after/before | collateral | 12 | 1.1267 | .35758 | .10322 |
| | main | 26 | .9115 | .62897 | .12335 |
| PHA diameters difference after/before | collateral | 12 | -.0208 | .71974 | .20777 |
| | main | 26 | -.0112 | .36899 | .07237 |
| RGEA diameters difference after/before | collateral | 12 | .5175 | .54423 | .15711 |
| | main | 23 | .5700 | .55288 | .11528 |

**Independent Samples Test**

| | | Levene's Test for Equality of Variances | | t-test for Equality of Means | | | | | | |
|---|---|---|---|---|---|---|---|---|---|---|
| | | F | Sig. | t | df | Sig. (2-tailed) | Mean Difference | Std. Error Difference | 95% Confidence Interval of the Difference Lower | Upper |
| GDA blood flow intencity alteration after/before | Equal variances assumed | 2.139 | .152 | -.939 | 36 | .354 | -1.33825 | 1.42456 | -4.22739 | 1.55088 |
| | Equal variances not assumed | | | -1.362 | 27.758 | .184 | -1.33825 | .98223 | -3.35104 | .67453 |
| PDA blood flow intencity alteration after/before | Equal variances assumed | 1.853 | .182 | -.529 | 36 | .600 | -2.70841 | 5.11835 | -13.08891 | 7.67209 |
| | Equal variances not assumed | | | -.769 | 27.514 | .448 | -2.70841 | 3.52201 | -9.92866 | 4.51185 |
| PHA blood flow intencity alteration after/before | Equal variances assumed | 5.773 | .022 | 1.196 | 36 | .240 | .32109 | .26848 | -.22342 | .86559 |
| | Equal variances not assumed | | | .857 | 11.881 | .408 | .32109 | .37472 | -.49626 | 1.13844 |
| RGEA blood flow intencity alteration after/before | Equal variances assumed | .203 | .655 | -.042 | 33 | .966 | -.06134 | 1.44789 | -3.00709 | 2.88440 |
| | Equal variances not assumed | | | -.048 | 30.500 | .962 | -.06134 | 1.28407 | -2.68197 | 2.55928 |
| GDA diameters ratio after/before | Equal variances assumed | 2.704 | .109 | -.622 | 36 | .538 | -.06655 | .10698 | -.28352 | .15043 |
| | Equal variances not assumed | | | -.781 | 35.596 | .440 | -.06655 | .08518 | -.23936 | .10627 |
| PDA diameters | Equal variances assumed | 2.465 | .125 | .350 | 36 | .728 | .04744 | .13545 | -.22726 | .32214 |

| | | | | | | | | | | |
|---|---|---|---|---|---|---|---|---|---|---|
| ratio after/before | Equal variances not assumed | | | .460 | 35.807 | .648 | .04744 | .10319 | -.16188 | .25676 |
| PHA diameters ratio after/before | Equal variances assumed | 5.449 | .025 | .003 | 36 | .998 | .00013 | .04688 | -.09494 | .09521 |
| | Equal variances not assumed | | | .002 | 13.964 | .998 | .00013 | .05817 | -.12466 | .12492 |
| RGEA diameters ratio after/before | Equal variances assumed | .023 | .881 | .223 | 33 | .825 | .02356 | .10580 | -.19169 | .23881 |
| | Equal variances not assumed | | | .226 | 23.383 | .823 | .02356 | .10420 | -.19180 | .23892 |
| GDA diameters difference after/before | Equal variances assumed | 2.647 | .112 | .067 | 36 | .947 | .02212 | .32928 | -.64569 | .68992 |
| | Equal variances not assumed | | | .079 | 31.999 | .938 | .02212 | .28049 | -.54922 | .59345 |
| PDA diameters difference after/before | Equal variances assumed | 2.901 | .097 | 1.100 | 36 | .278 | .21513 | .19549 | -.18135 | .61161 |
| | Equal variances not assumed | | | 1.338 | 34.180 | .190 | .21513 | .16084 | -.11168 | .54194 |
| PHA diameters difference after/before | Equal variances assumed | 4.162 | .049 | -.055 | 36 | .956 | -.00968 | .17548 | -.36558 | .34622 |
| | Equal variances not assumed | | | -.044 | 13.742 | .966 | -.00968 | .22001 | -.48239 | .46303 |
| RGEA diameters difference after/before | Equal variances assumed | .173 | .680 | -.268 | 33 | .790 | -.05250 | .19586 | -.45099 | .34599 |
| | Equal variances not assumed | | | -.269 | 22.739 | .790 | -.05250 | .19487 | -.45587 | .35087 |

NPar Tests
Mann-Whitney Test

**Ranks**

| | Type od parenchimal blood flow | N | Mean Rank | Sum of Ranks |
|---|---|---|---|---|
| GDA blood flow intencity alteration after/before | collateral | 12 | 18.25 | 219.00 |
| | main | 26 | 20.08 | 522.00 |
| | Total | 38 | | |
| PDA blood flow intencity alteration after/before | collateral | 12 | 22.75 | 273.00 |
| | main | 26 | 18.00 | 468.00 |
| | Total | 38 | | |
| PHA blood flow intencity alteration after/before | collateral | 12 | 20.42 | 245.00 |
| | main | 26 | 19.08 | 496.00 |
| | Total | 38 | | |
| RGEA blood flow intencity alteration after/before | collateral | 12 | 19.33 | 232.00 |
| | main | 23 | 17.30 | 398.00 |
| | Total | 35 | | |
| GDA diameters ratio after/before | collateral | 12 | 18.25 | 219.00 |
| | main | 26 | 20.08 | 522.00 |
| | Total | 38 | | |
| PDA diameters ratio after/before | collateral | 12 | 22.75 | 273.00 |
| | main | 26 | 18.00 | 468.00 |
| | Total | 38 | | |
| PHA diameters ratio after/before | collateral | 12 | 18.58 | 223.00 |
| | main | 26 | 19.92 | 518.00 |
| | Total | 38 | | |
| RGEA diameters ratio after/before | collateral | 12 | 19.33 | 232.00 |
| | main | 23 | 17.30 | 398.00 |
| | Total | 35 | | |
| GDA diameters difference after/before | collateral | 12 | 19.33 | 232.00 |
| | main | 26 | 19.58 | 509.00 |
| | Total | 38 | | |
| PDA diameters difference after/before | collateral | 12 | 22.71 | 272.50 |
| | main | 26 | 18.02 | 468.50 |
| | Total | 38 | | |
| PHA diameters difference after/before | collateral | 12 | 18.63 | 223.50 |
| | main | 26 | 19.90 | 517.50 |
| | Total | 38 | | |
| RGEA diameters difference after/before | collateral | 12 | 18.08 | 217.00 |
| | main | 23 | 17.96 | 413.00 |
| | Total | 35 | | |

**Test Statistics**

| | Mann-Whitney U | Wilcoxon W | Z | Asymp. Sig. (2-tailed) | Exact Sig. [2*(1-tailed Sig.)] |
|---|---|---|---|---|---|
| GDA blood flow intencity alteration after/before | 141.000 | 219.000 | -.471 | .638 | .653 |
| PDA blood flow intencity alteration after/before | 117.000 | 468.000 | -1.225 | .221 | .230 |
| PHA blood flow intencity alteration after/before | 145.000 | 496.000 | -.345 | .730 | .745 |
| RGEA blood flow intencity alteration after/before | 122.000 | 398.000 | -.556 | .578 | .595 |
| GDA diameters ratio after/before | 141.000 | 219.000 | -.471 | .638 | .653 |
| PDA diameters ratio after/before | 117.000 | 468.000 | -1.225 | .221 | .230 |
| PHA diameters ratio after/before | 145.000 | 223.000 | -.345 | .730 | .745 |
| RGEA diameters ratio after/before | 122.000 | 398.000 | -.556 | .578 | .595 |
| GDA diameters difference after/before | 154.000 | 232.000 | -.063 | .950 | .963 |
| PDA diameters difference after/before | 117.500 | 468.500 | -1.210 | .226 | .230 |
| PHA diameters difference after/before | 145.500 | 223.500 | -.330 | .741 | .745 |
| RGEA diameters difference after/before | 137.000 | 413.000 | -.035 | .972 | .986 |

Crosstabs

**Case Processing Summary**

| | Cases | | | | | |
|---|---|---|---|---|---|---|
| | Valid | | Missing | | Total | |
| | N | Percent | N | Percent | N | Percent |
| Puls preservation * Type od parenchimal blood flow | 38 | 100.0% | 0 | 0.0% | 38 | 100.0% |

**Puls preservation * Type od parenchimal blood flow Crosstabulation**

| | | | Type od parenchimal blood flow | | |
|---|---|---|---|---|---|
| | | | collateral | main | Total |
| Puls preservation | no | Count | $4_a$ | $0_b$ | 4 |
| | | % within Type od parenchimal blood flow | 33.3% | 0.0% | 10.5% |
| | yes | Count | $8_a$ | $26_b$ | 34 |

| | | | | |
|---|---|---|---|---|
| | % within Type od parenchimal blood flow | 66.7% | 100.0% | 89.5% |
| Total | Count | 12 | 26 | 38 |
| | % within Type od parenchimal blood flow | 100.0% | 100.0% | 100.0% |

**Chi-Square Tests**

| | Value | df | Asymptotic Significance (2-sided) | Exact Sig. (2-sided) | Exact Sig. (1-sided) |
|---|---|---|---|---|---|
| Pearson Chi-Square | 9.686 | 1 | .002 | | |
| Continuity Correction | 6.470 | 1 | .011 | | |
| Likelihood Ratio | 10.297 | 1 | .001 | | |
| Fisher's Exact Test | | | | .007 | .007 |
| Linear-by-Linear Association | 9.431 | 1 | .002 | | |
| N of Valid Cases | 38 | | | | |

T-Test

**Group Statistics**

| | Puls preservation | N | Mean | Std. Deviation | Std. Error Mean |
|---|---|---|---|---|---|
| Linear blood flow velosity decrease.% | no | 4 | -48.25 | 32.212 | 16.106 |
| | yes | 34 | -18.09 | 15.044 | 2.580 |

**Independent Samples Test**

| | | Levene's Test for Equality of Variances | | t-test for Equality of Means | | | | | | |
|---|---|---|---|---|---|---|---|---|---|---|
| | | | | | | | | | 95% Confidence Interval of the Difference | |
| | | F | Sig. | t | df | Sig. (2-tailed) | Mean Difference | Std. Error Difference | Lower | Upper |
| Linear blood flow velosity decrease.% | Equal variances assumed | 5.853 | .021 | -3.328 | 36 | .002 | -30.162 | 9.062 | -48.541 | -11.782 |
| | Equal variances not assumed | | | -1.849 | 3.156 | .157 | -30.162 | 16.311 | -80.651 | 20.328 |

NPar Tests

Mann-Whitney Test

**Ranks**

| | Puls preservation | N | Mean Rank | Sum of Ranks |
|---|---|---|---|---|
| Linear blood flow velosity decrease.% | no | 4 | 10.38 | 41.50 |
| | yes | 34 | 20.57 | 699.50 |
| | Total | 38 | | |

**Test Statistics**

| | Linear blood flow velosity decrease.% |
|---|---|
| Mann-Whitney U | 31.500 |
| Wilcoxon W | 41.500 |
| Z | -1.741 |
| Asymp. Sig. (2-tailed) | .082 |
| Exact Sig. [2*(1-tailed Sig.)] | .083 |

T-Test

**Group Statistics**

| | Type od parenchimal blood flow | N | Mean | Std. Deviation | Std. Error Mean |
|---|---|---|---|---|---|
| Linear blood flow velosity decrease.% | collateral | 12 | -42.58 | 18.696 | 5.397 |
| | main | 26 | -11.42 | 8.990 | 1.763 |

**Independent Samples Test**

| | | Levene's Test for Equality of Variances | | t-test for Equality of Means | | | | | | |
|---|---|---|---|---|---|---|---|---|---|---|
| | | | | | | | | | 95% Confidence Interval of the Difference | |
| | | F | Sig. | t | df | Sig. (2-tailed) | Mean Difference | Std. Error Difference | Lower | Upper |
| Linear blood flow velosity decrease.% | Equal variances assumed | 5.175 | .029 | -6.995 | 36 | .000 | -31.160 | 4.455 | -40.195 | -22.126 |
| | Equal variances not assumed | | | -5.488 | 13.406 | .000 | -31.160 | 5.678 | -43.389 | -18.932 |

NPar Tests

Mann-Whitney Test

**Ranks**

| Type od parenchimal blood flow | N | Mean Rank | Sum of Ranks |
|---|---|---|---|

| | | | | |
|---|---|---|---|---|
| Linear blood flow velosity decrease.% | collateral | 12 | 8.63 | 103.50 |
| | main | 26 | 24.52 | 637.50 |
| | Total | 38 | | |

**Test Statistics**

| | Linear blood flow velosity decrease.% |
|---|---|
| Mann-Whitney U | 25.500 |
| Wilcoxon W | 103.500 |
| Z | -4.109 |
| Asymp. Sig. (2-tailed) | .000 |
| Exact Sig. [2*(1-tailed Sig.)] | .000 |

Graph

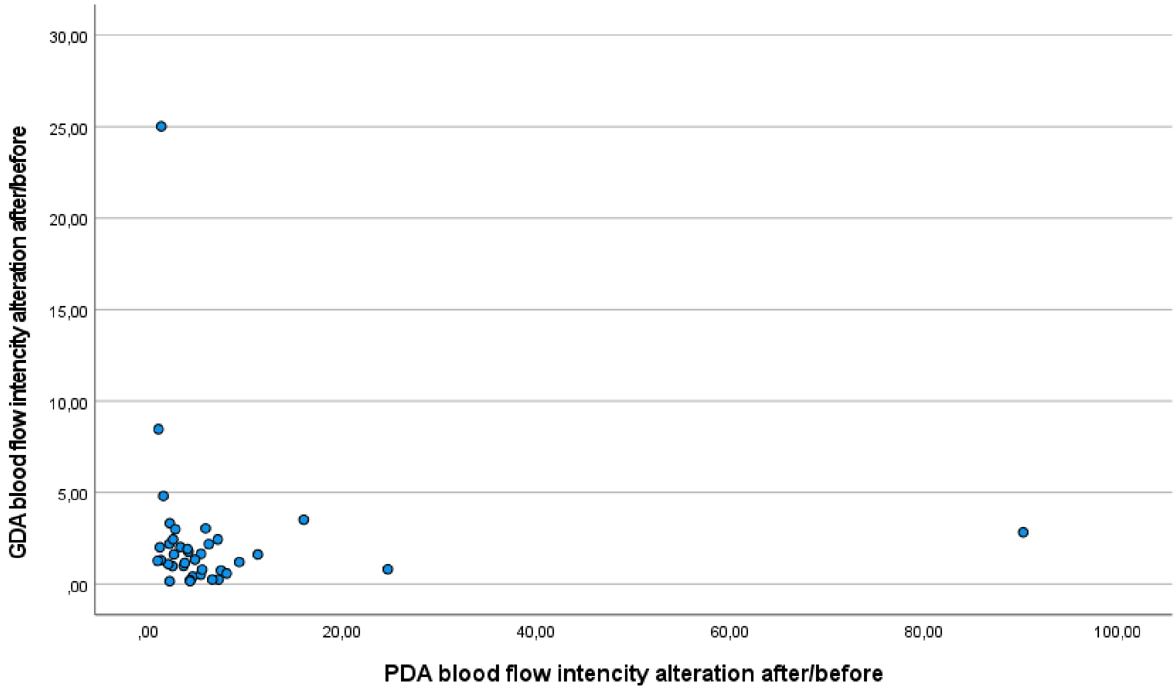


Graph

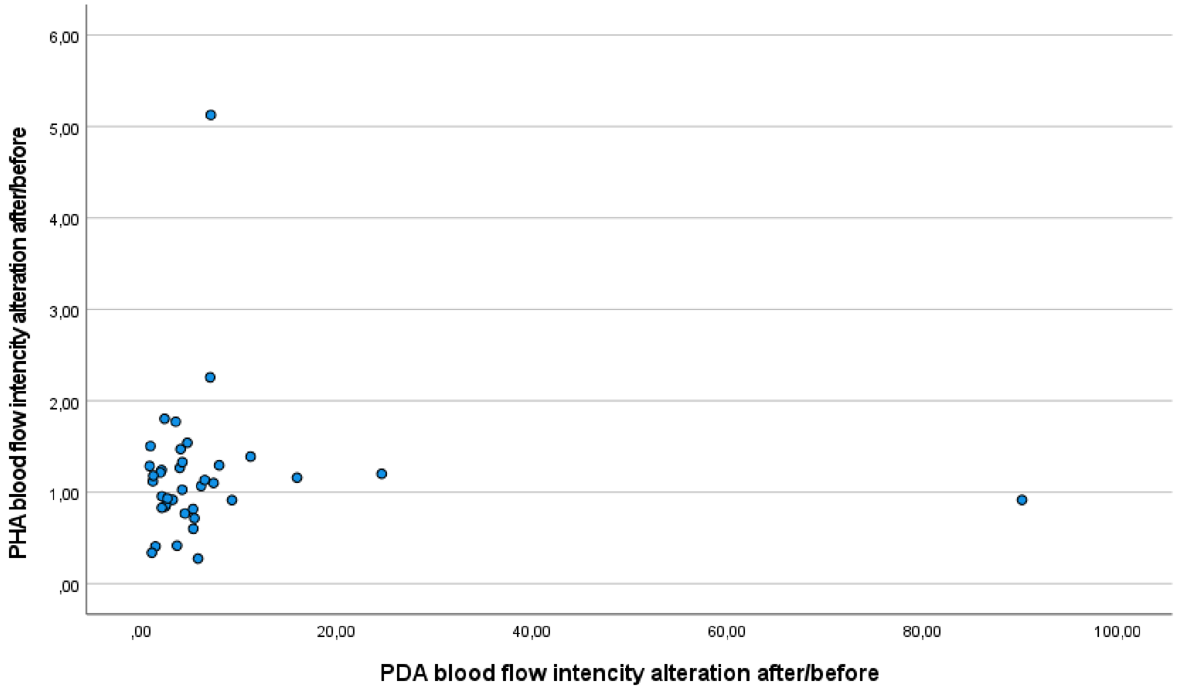


Graph

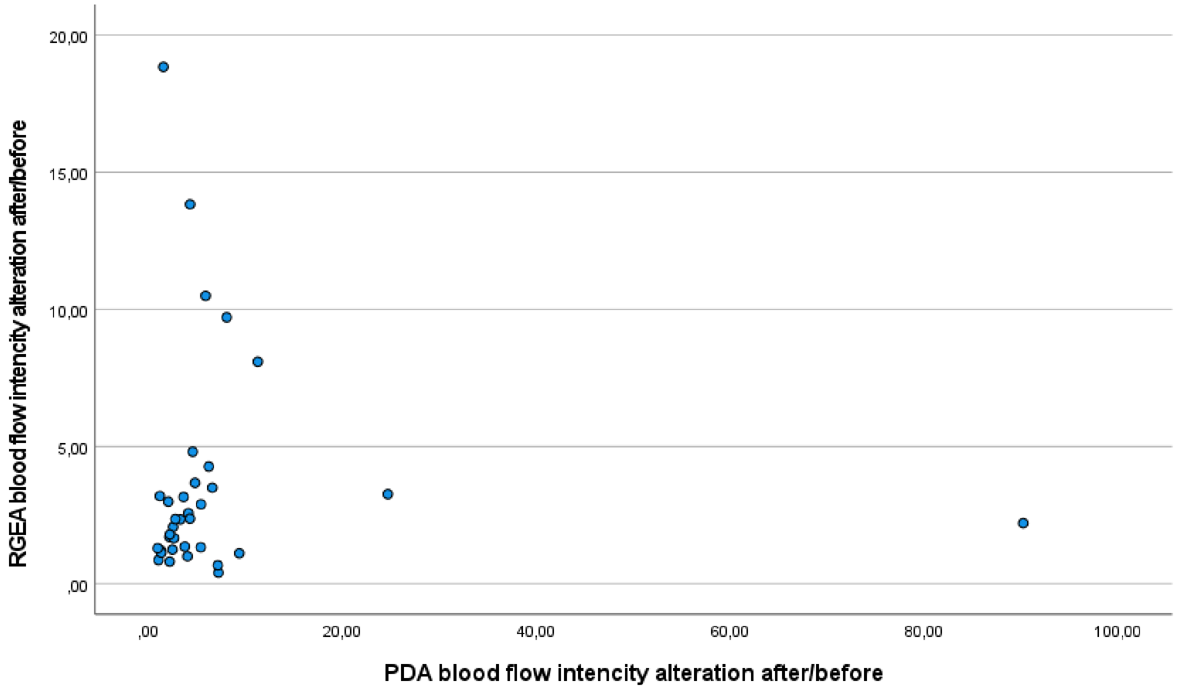


Correlations

**Correlations**

| | | GDA blood flow intencity alteration after/before | PDA blood flow intencity alteration after/before | PHA blood flow intencity alteration after/before | RGEA blood flow intencity alteration after/before |
|---|---|---|---|---|---|
| | Pearson Correlation | 1 | -.042 | -.057 | -.057 |
| | Sig. (2-tailed) | | .800 | .735 | .744 |

| | | | | | |
|---|---|---|---|---|---|
| GDA blood flow intencity alteration after/before | N | 38 | 38 | 38 | 35 |
| PDA blood flow intencity alteration after/before | Pearson Correlation | -.042 | 1 | -.015 | -.033 |
| | Sig. (2-tailed) | .800 | | .926 | .851 |
| | N | 38 | 38 | 38 | 35 |
| PHA blood flow intencity alteration after/before | Pearson Correlation | -.057 | -.015 | 1 | -.246 |
| | Sig. (2-tailed) | .735 | .926 | | .155 |
| | N | 38 | 38 | 38 | 35 |
| RGEA blood flow intencity alteration after/before | Pearson Correlation | -.057 | -.033 | -.246 | 1 |
| | Sig. (2-tailed) | .744 | .851 | .155 | |
| | N | 35 | 35 | 35 | 35 |

Nonparametric Correlations

**Correlations**

| | | | GDA blood flow intencity alteration after/before | PDA blood flow intencity alteration after/before | PHA blood flow intencity alteration after/before | RGEA blood flow intencity alteration after/before |
|---|---|---|---|---|---|---|
| Spearman's rho | GDA blood flow intencity alteration after/before | Correlation Coefficient | 1.000 | -.246 | -.109 | -.057 |
| | | Sig. (2-tailed) | . | .137 | .515 | .746 |
| | | N | 38 | 38 | 38 | 35 |
| | PDA blood flow intencity alteration after/before | Correlation Coefficient | -.246 | 1.000 | .072 | .268 |
| | | Sig. (2-tailed) | .137 | . | .669 | .120 |
| | | N | 38 | 38 | 38 | 35 |
| | PHA blood flow intencity alteration after/before | Correlation Coefficient | -.109 | .072 | 1.000 | -.198 |
| | | Sig. (2-tailed) | .515 | .669 | . | .254 |
| | | N | 38 | 38 | 38 | 35 |
| | RGEA blood flow intencity alteration after/before | Correlation Coefficient | -.057 | .268 | -.198 | 1.000 |
| | | Sig. (2-tailed) | .746 | .120 | .254 | . |
| | | N | 35 | 35 | 35 | 35 |

T-Test

**Group Statistics**

| | Type | N | Mean | Std. Deviation | Std. Error Mean |
|---|---|---|---|---|---|
| | classical | 30 | 2.4920 | 4.53800 | .82852 |

| | | | | | |
|---|---|---|---|---|---|
| GDA blood flow intencity alteration after/before | aberrant | 8 | 1.9273 | 1.47923 | .52299 |
| PDA blood flow intencity alteration after/before | classical | 30 | 8.1891 | 16.26124 | 2.96888 |
| | aberrant | 8 | 4.0855 | 2.03198 | .71841 |
| PHA blood flow intencity alteration after/before | classical | 30 | 1.3255 | .80462 | .14690 |
| | aberrant | 8 | .6580 | .28386 | .10036 |
| RGEA blood flow intencity alteration after/before | classical | 28 | 2.8460 | 2.97509 | .56224 |
| | aberrant | 7 | 6.3733 | 6.28371 | 2.37502 |
| GDA diameters ratio after/before | classical | 30 | 1.1064 | .32319 | .05901 |
| | aberrant | 8 | 1.1170 | .23592 | .08341 |
| PDA diameters ratio after/before | classical | 30 | 1.4877 | .41716 | .07616 |
| | aberrant | 8 | 1.3821 | .21514 | .07606 |
| PHA diameters ratio after/before | classical | 30 | .9614 | .09914 | .01810 |
| | aberrant | 8 | 1.1458 | .14627 | .05171 |
| RGEA diameters ratio after/before | classical | 28 | 1.2165 | .25541 | .04827 |
| | aberrant | 7 | 1.4785 | .35862 | .13554 |
| GDA diameters difference after/before | classical | 30 | .1983 | .98412 | .17967 |
| | aberrant | 8 | .3600 | .73681 | .26050 |
| PDA diameters difference after/before | classical | 30 | 1.0113 | .58668 | .10711 |
| | aberrant | 8 | .8600 | .47059 | .16638 |
| PHA diameters difference after/before | classical | 30 | -.1297 | .44713 | .08163 |
| | aberrant | 8 | .4187 | .44809 | .15842 |
| RGEA diameters difference after/before | classical | 28 | .4986 | .56314 | .10642 |
| | aberrant | 7 | .7657 | .41689 | .15757 |

**Independent Samples Test**

| | | Levene's Test for Equality of Variances | | t-test for Equality of Means | | | | | | |
|---|---|---|---|---|---|---|---|---|---|---|
| | | F | Sig. | t | df | Sig. (2-tailed) | Mean Difference | Std. Error Difference | 95% Confidence Interval of the Difference Lower | Upper |
| GDA blood flow intencity alteration after/before | Equal variances assumed | .473 | .496 | .344 | 36 | .733 | .56472 | 1.64134 | -2.76407 | 3.89350 |
| | Equal variances not assumed | | | .576 | 34.212 | .568 | .56472 | .97978 | -1.42598 | 2.55541 |

| | | | | | | | | | | |
|---|---|---|---|---|---|---|---|---|---|---|
| PDA blood flow intencity alteration after/before | Equal variances assumed | 1.185 | .284 | .705 | 36 | .485 | 4.10364 | 5.81841 | -7.69665 | 15.90392 |
| | Equal variances not assumed | | | 1.343 | 32.040 | .189 | 4.10364 | 3.05457 | -2.11801 | 10.32528 |
| PHA blood flow intencity alteration after/before | Equal variances assumed | .544 | .466 | 2.289 | 36 | .028 | .66744 | .29164 | .07596 | 1.25892 |
| | Equal variances not assumed | | | 3.751 | 32.793 | .001 | .66744 | .17791 | .30539 | 1.02949 |
| RGEA blood flow intencity alteration after/before | Equal variances assumed | 6.969 | .013 | -2.198 | 33 | .035 | -3.52731 | 1.60473 | -6.79217 | -.26246 |
| | Equal variances not assumed | | | -1.445 | 6.687 | .194 | -3.52731 | 2.44066 | -9.35384 | 2.29921 |
| GDA diameters ratio after/before | Equal variances assumed | .129 | .721 | -.086 | 36 | .932 | -.01058 | .12262 | -.25927 | .23811 |
| | Equal variances not assumed | | | -.104 | 14.861 | .919 | -.01058 | .10217 | -.22853 | .20737 |
| PDA diameters ratio after/before | Equal variances assumed | .844 | .364 | .687 | 36 | .496 | .10560 | .15369 | -.20610 | .41731 |
| | Equal variances not assumed | | | .981 | 22.591 | .337 | .10560 | .10764 | -.11729 | .32850 |
| PHA diameters ratio after/before | Equal variances assumed | 4.604 | .039 | -4.217 | 36 | .000 | -.18442 | .04373 | -.27311 | -.09573 |
| | Equal variances not assumed | | | -3.366 | 8.788 | .009 | -.18442 | .05479 | -.30882 | -.06002 |
| RGEA diameters ratio after/before | Equal variances assumed | 1.090 | .304 | -2.238 | 33 | .032 | -.26196 | .11707 | -.50016 | -.02377 |
| | Equal variances not assumed | | | -1.821 | 7.591 | .108 | -.26196 | .14388 | -.59690 | .07297 |

| | | | | | | | | | | |
|---|---|---|---|---|---|---|---|---|---|---|
| GDA diameters difference after/before | Equal variances assumed | .182 | .672 | -.432 | 36 | .669 | -.16167 | .37449 | -.92116 | .59783 |
| | Equal variances not assumed | | | -.511 | 14.455 | .617 | -.16167 | .31645 | -.83840 | .51506 |
| PDA diameters difference after/before | Equal variances assumed | .337 | .565 | .672 | 36 | .506 | .15133 | .22521 | -.30541 | .60807 |
| | Equal variances not assumed | | | .765 | 13.447 | .458 | .15133 | .19788 | -.27471 | .57738 |
| PHA diameters difference after/before | Equal variances assumed | .281 | .599 | -3.081 | 36 | .004 | -.54842 | .17799 | -.90940 | -.18743 |
| | Equal variances not assumed | | | -3.077 | 11.023 | .010 | -.54842 | .17822 | -.94057 | -.15626 |
| RGEA diameters difference after/before | Equal variances assumed | .757 | .391 | -1.172 | 33 | .250 | -.26714 | .22798 | -.73098 | .19669 |
| | Equal variances not assumed | | | -1.405 | 12.160 | .185 | -.26714 | .19014 | -.68082 | .14653 |

NPar Tests
Mann-Whitney Test

**Ranks**

| | Type | N | Mean Rank | Sum of Ranks |
|---|---|---|---|---|
| GDA blood flow intencity alteration after/before | classical | 30 | 19.20 | 576.00 |
| | aberrant | 8 | 20.63 | 165.00 |
| | Total | 38 | | |
| PDA blood flow intencity alteration after/before | classical | 30 | 19.73 | 592.00 |
| | aberrant | 8 | 18.63 | 149.00 |
| | Total | 38 | | |
| PHA blood flow intencity alteration after/before | classical | 30 | 22.83 | 685.00 |
| | aberrant | 8 | 7.00 | 56.00 |
| | Total | 38 | | |
| RGEA blood flow intencity alteration after/before | classical | 28 | 16.21 | 454.00 |
| | aberrant | 7 | 25.14 | 176.00 |
| | Total | 35 | | |
| | classical | 30 | 19.20 | 576.00 |

| | | | | |
|---|---|---|---|---|
| GDA diameters ratio after/before | aberrant | 8 | 20.63 | 165.00 |
| | Total | 38 | | |
| PDA diameters ratio after/before | classical | 30 | 19.73 | 592.00 |
| | aberrant | 8 | 18.63 | 149.00 |
| | Total | 38 | | |
| PHA diameters ratio after/before | classical | 30 | 16.17 | 485.00 |
| | aberrant | 8 | 32.00 | 256.00 |
| | Total | 38 | | |
| RGEA diameters ratio after/before | classical | 28 | 16.21 | 454.00 |
| | aberrant | 7 | 25.14 | 176.00 |
| | Total | 35 | | |
| GDA diameters difference after/before | classical | 30 | 19.02 | 570.50 |
| | aberrant | 8 | 21.31 | 170.50 |
| | Total | 38 | | |
| PDA diameters difference after/before | classical | 30 | 19.92 | 597.50 |
| | aberrant | 8 | 17.94 | 143.50 |
| | Total | 38 | | |
| PHA diameters difference after/before | classical | 30 | 16.40 | 492.00 |
| | aberrant | 8 | 31.13 | 249.00 |
| | Total | 38 | | |
| RGEA diameters difference after/before | classical | 28 | 16.89 | 473.00 |
| | aberrant | 7 | 22.43 | 157.00 |
| | Total | 35 | | |

**Test Statistics**

| | Mann-Whitney U | Wilcoxon W | Z | Asymp. Sig. (2-tailed) | Exact Sig. [2*(1-tailed Sig.)] |
|---|---|---|---|---|---|
| GDA blood flow intencity alteration after/before | 111.000 | 576.000 | -.322 | .747 | .765 |
| PDA blood flow intencity alteration after/before | 113.000 | 149.000 | -.251 | .802 | .820 |
| PHA blood flow intencity alteration after/before | 20.000 | 56.000 | -3.581 | .000 | .000 |
| RGEA blood flow intencity alteration after/before | 48.000 | 454.000 | -2.062 | .039 | .039 |
| GDA diameters ratio after/before | 111.000 | 576.000 | -.322 | .747 | .765 |
| PDA diameters ratio after/before | 113.000 | 149.000 | -.251 | .802 | .820 |
| PHA diameters ratio after/before | 20.000 | 485.000 | -3.581 | .000 | .000 |

| | | | | | |
|---|---|---|---|---|---|
| RGEA diameters ratio after/before | 48.000 | 454.000 | -2.062 | .039 | .039 |
| GDA diameters difference after/before | 105.500 | 570.500 | -.519 | .604 | .610 |
| PDA diameters difference after/before | 107.500 | 143.500 | -.448 | .654 | .661 |
| PHA diameters difference after/before | 27.000 | 492.000 | -3.334 | .001 | .000 |
| RGEA diameters difference after/before | 67.000 | 473.000 | -1.279 | .201 | .214 |
