# Supplementary material for: Hemodynamic, Surgical and Oncological Outcomes of 40 Distal Pancreatectomies with Celiac and Left Gastric Arteries Resection (DP CAR) without Arterial Reconstructions and Preoperative Embolization
Source: Cancers (Basel). 2022 Feb 28;14(5):1254. doi: 10.3390/cancers14051254 (PMC8909059; doi:10.3390/cancers14051254)
Supplement: Supplementary file 1 [file cancers-14-01254-s001.zip › cancers-1582412-supplementary/Supplement/Table S5. Tumor size for PDAC.pdf]

Table S5. Tumor size in mm of the pancreatic ductal adenocarcinoma (PDAC) before chemotherapy, before surgery and after pathological examination

Patient	Tumor size, pathol, mm	Tumor size before NAChT on CT	Tumor size before surgery on CT
1	52	0	50
2	43	0	49
3	32	35	35
4	64	0	71
5	43	0	47
6	21	75	61
7	52	64	62
8	42	0	48
9	37	0	39
10	48	0	55
11	62	0	66
12	65	0	69
13	64	0	66
14	42	58	49
15	73	0	74
16	67	81	70
17	36	54	38
18	38	56	41
19	55	0	55
20	61	0	64
21	44	48	48
22	59	0	60
23	48	61	60
24	52	71	65
25	57	74	68
26	43	49	47
27	38	44	40
28	41	64	51
29	43	53	47
30	48	66	53
31	38	52	42
32	33	46	46
33	37	51	41
34	48	77	53
